# Structural modifications of abietic acid and a computational protocol to predict their compatibility in different environments

**DOI:** 10.1039/d6ra04565k

**Published:** 2026-08-06

**Authors:** Jéssica Macedo, Pedro Miranda, Américo Alves, Cláudia Alves, Nuno Alves, Dina Farinha, Susana M. M. Lopes, Catarina Gonçalves, Anna B. Brito, Vera A. Gamboa, Nelson A. M. Pereira, A. J. Lopes Jesus, Bernardo A. Nogueira, Licínia L. G. Justino, Cláudio M. Nunes, Dina Murtinho, Marta Pineiro, A. Mendes Ferreira, Rui Fausto, Teresa M. V. D. Pinho e Melo

**Affiliations:** a University of Coimbra, Coimbra Chemistry Centre – Institute of Molecular Sciences and Department of Chemistry 3004-535 Coimbra Portugal tmelo@ci.uc.pt; b United Resins, S.A Figueira da Foz Portugal

## Abstract

Abietic acid, a major diterpenoid of rosin, is a renewable feedstock with wide industrial relevance. However, its polarity limits compatibility with apolar environments. A computational protocol has been developed to predict how structural modifications of abietic acid influence molecular polarity and compatibility with different environments. A representative series of abietic acid derivatives was selected for the theoretical study, including non-carbonyl derivatives and two new long-chain esters, dodecyl and hexadecyl abietates. An extensive molecular mechanics conformational search, followed by DFT optimizations, enabled the estimation of population-weighted dipole moments and Gibbs free energies of solvation in solvents of different polarity (pentyl ethanoate and *n*-octane). The resulting descriptors were compared with experimental HPLC-DAD retention times and thin-layer chromatography *R*_f_ values, showing good agreement between computed and experimental trends. The calculations correctly capture subtle conformational effects, including the pseudo-amphiphilic character of dodecyl abietate arising from folded conformations. Overall, the computational framework provides a predictive tool for guiding the rational design of abietic acid derivatives with tailored polarity.

## Abstract 2

A computational framework predicts how abietic acid modifications influence polarity and compatibility, capturing subtle conformational effects such as pseudo‑amphiphilicity in long‑chain esters. Copilot assisted in creating the graphical abstract.

## Graphical abstract

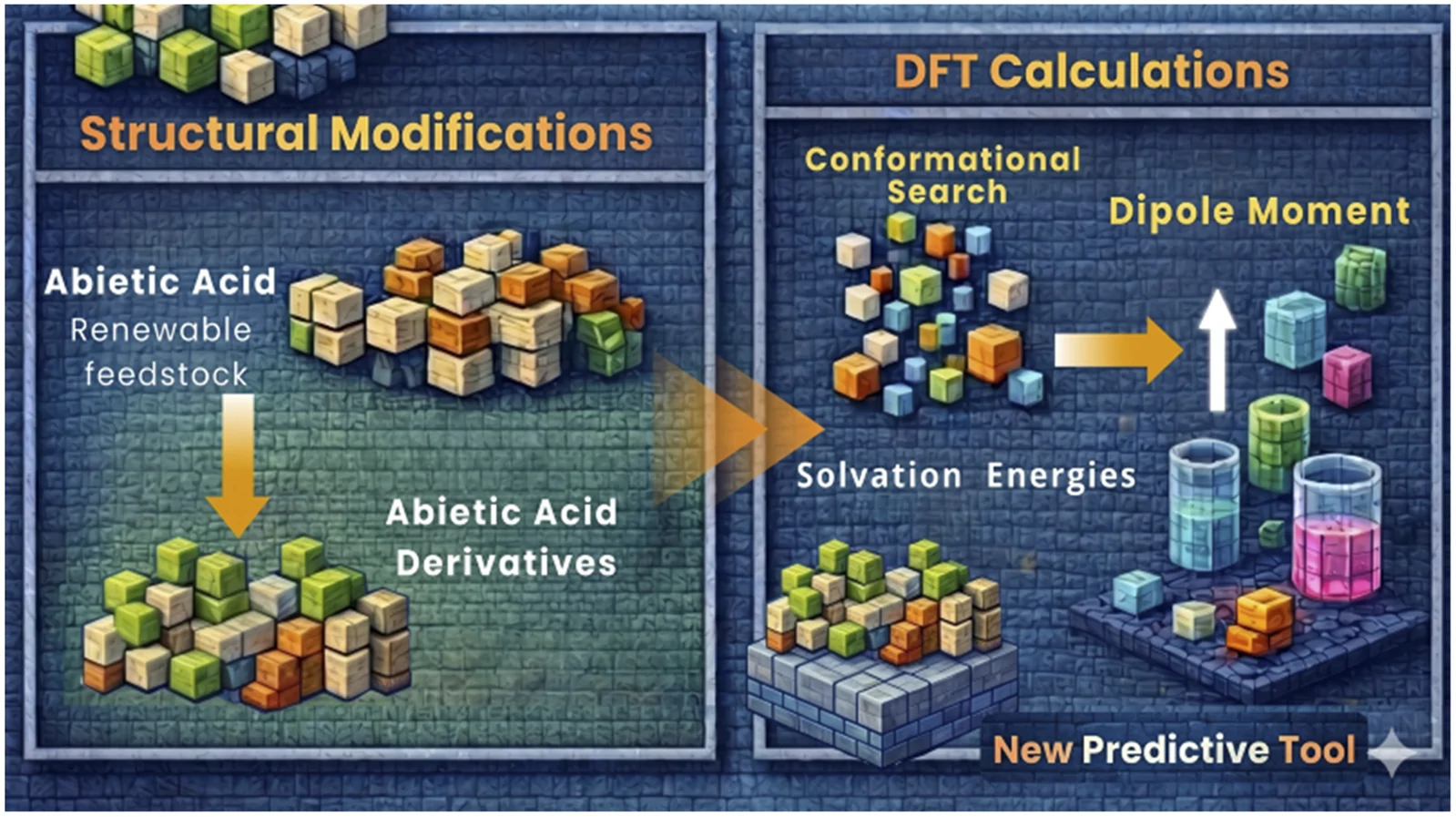

## Introduction

Abietic acid is one of the main constituents of rosin, a natural product resulting from the exudation of pine trees and other conifers. Rosin, and therefore abietic acid, is a raw material that is used industrially in the manufacture of adhesives, paints, varnishes, chewing gum, detergents, among other products.^1^ Rosins offer several advantages over petrochemical-based resins, particularly regarding sustainability. They originate from renewable natural resources, exhibit a low environmental footprint, while delivering performance that is comparable to that of petrochemical resins across many applications. In addition to abietic acid, rosin is composed of several other terpenic acids, including palustric, neoabietic, and levopimaric acids, among others.^2,3^

Rosin acids present some common structural features, namely a carboxylic acid group, which enables dipolar interactions, and a rigid hydrophobic tricyclic diterpenoid framework. These structural features are responsible for the amphiphilic character of these molecules.^4^

One practical consequence of the polarity of rosin is its excellent adhesion to polar substrates such as cardboard, wood, and paper, which underlies its widespread use as a tackifier in adhesive formulations. By contrast, this same polarity results in limited miscibility with more apolar materials, including aliphatic polymers such as polyethylene and polypropylene, thereby restricting the range of its applications, especially in hot-melt adhesives.^5^

The hydrophilic/hydrophobic balance of rosin can be tailored through the preparation of derivatives bearing different functional groups. For example, a recent study demonstrated that wood rosin modified with maleic anhydride can be used in the preparation of polypropylene composites reinforced with natural fibers.^6^ In this system, the modified rosin was used as a compatibilizing agent to bridge the hydrophobic polypropylene matrix and the hydrophilic natural fibers extracted from pine roots.^6^

When the aim is to reduce the polarity of rosin, one of the most employed strategies involves the conversion of carboxylic acid groups into esters, including mono-, di-, and triesters. For instance, Dal *et al.* reported that rosin chemically modified with fumaric acid, esterified with pentaerythritol, and emulsified with cationic starch can be employed as a sustainable hydrophobic additive to improve the water resistance of paper and packaging materials. Such formulations represent a viable bio-based alternative to conventional petroleum-derived additives.^7^

Beyond esterification, the incorporation of additional hydrophobic moieties into the rosin structure provides a further strategy for tailoring its amphiphilic behaviour. In this context, a bio-based anionic surfactant, sodium *N*-dodecyl-maleimidepimaric carboxylate, was successfully synthesized from rosin. By introducing a long hydrophobic alkyl chain into the rosin-based structure, the researchers obtained a surfactant capable of self-assembling into wormlike micelles in aqueous media, which exhibit considerable potential for applications in enhanced oil recovery, cosmetic formulations, and industrial cleaning processes.^8^

Rosin has also been used as a renewable feedstock for the development of hydrophobic thermosetting resins intended for electronic and microelectronic applications. A monomer derived from dehydroabietic acid was synthesised *via* bromination, coupling with benzocyclobutene, and subsequent functionalization with allyl groups. The resulting monomer was subsequently polymerized to yield a thermosetting resin exhibiting excellent hydrophobicity together with favorable dielectric properties.^9^

However, although these modifications lead to a decrease in polarity, they are generally insufficient to render the rosin compatible with more apolar environments.

Computational studies have been widely employed to predict the structural, electronic, and thermodynamic properties of natural products, including solvation effects, dipole moments, and Gibbs free energies. These approaches enable the characterization of the behavior of natural compounds in different environments and are commonly used to improve the understanding of the relationship between molecular structure and biological activity.^10–12^

In this context, the present work aimed at the development of a computational protocol capable of predicting how different structural modifications of abietic acid confer compatibility in different environments. A series of abietic acid derivatives was synthesized to serve as the foundation for the theoretical study. Comparison between computational predictions and experimental behavior was enabled by establishing a relative polarity scale based on HPLC-DAD retention time and thin-layer chromatography (TLC) *R*_f_ values, which corroborated the theoretical results.

## Results and discussion

### Synthesis

A series of modifications targeting the carboxylic acid moiety of abietic acid were carried out in order to obtain derivatives bearing functionalities with different polarities.

**Scheme 1:**
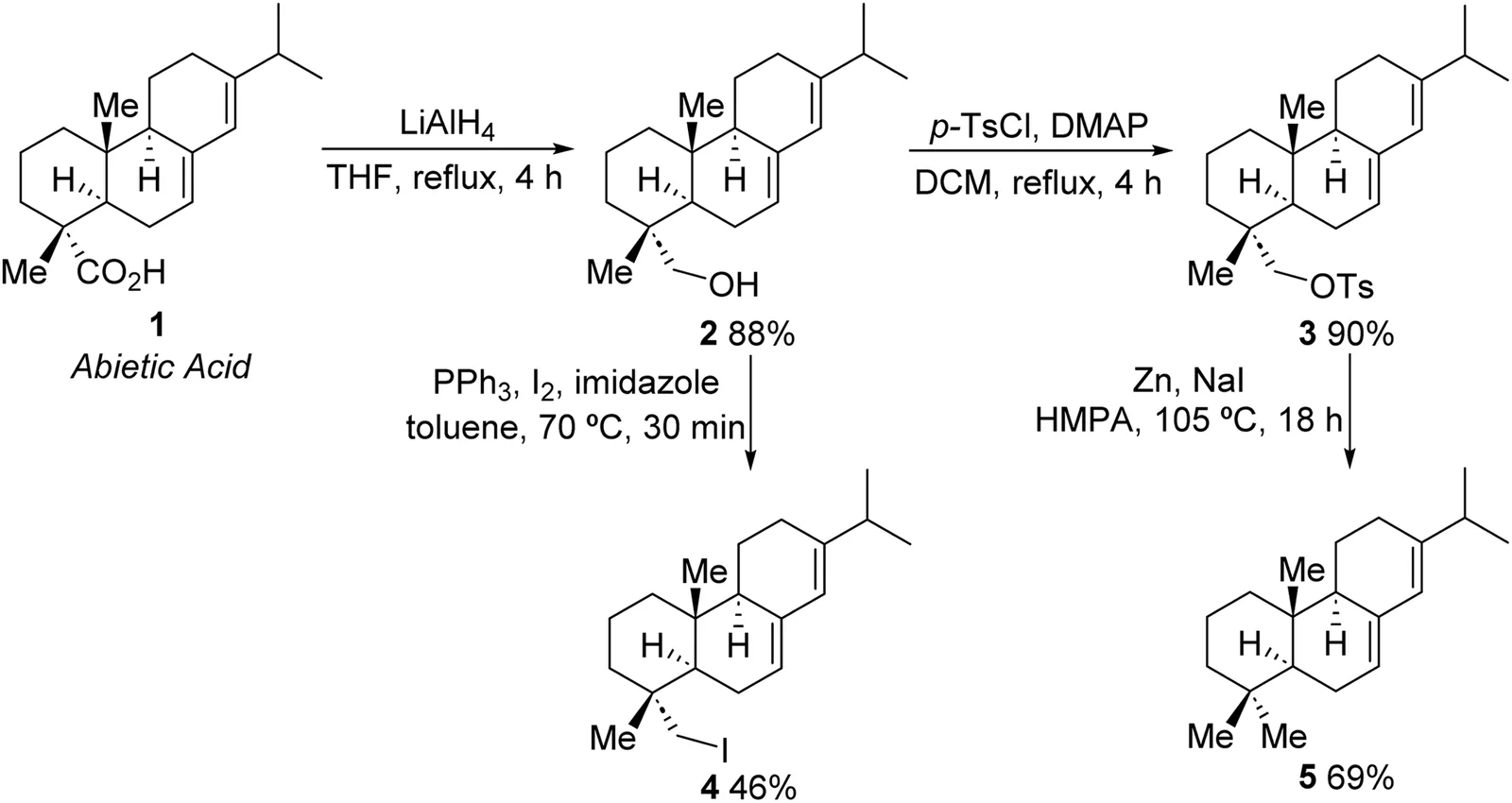
Synthesis of abietic acid derivatives.

The synthetic methodologies outlined in Scheme 1 are based on procedures already described in the literature.^13–15^ Reduction of the acid moiety of abietic acid was accomplished using lithium aluminium hydride in refluxing tetrahydrofuran (THF), yielding alcohol 2 in 88% yield. The tosyl derivative 3 was obtained in 90% yield through the reaction of alcohol 2 with tosyl chloride, in the presence of a catalytic amount of 4-dimethylaminopyridine (DMAP) in dichloromethane, at reflux. The iodo derivative 4 was obtained in 46% yield, by reacting the alcohol 2 with molecular iodine in the presence of triphenylphosphine and imidazole, in toluene at 70 °C for 30 min.^13^ The reaction of tosyl derivative 3 with zinc and sodium iodide in *N*,*N*,*N*,*N*-hexamethylphosphoramide (HMPA) at 105 °C for 18 h gave the derivative 5 in 69% yield (Scheme 1).^14,15^

**Scheme 2:**
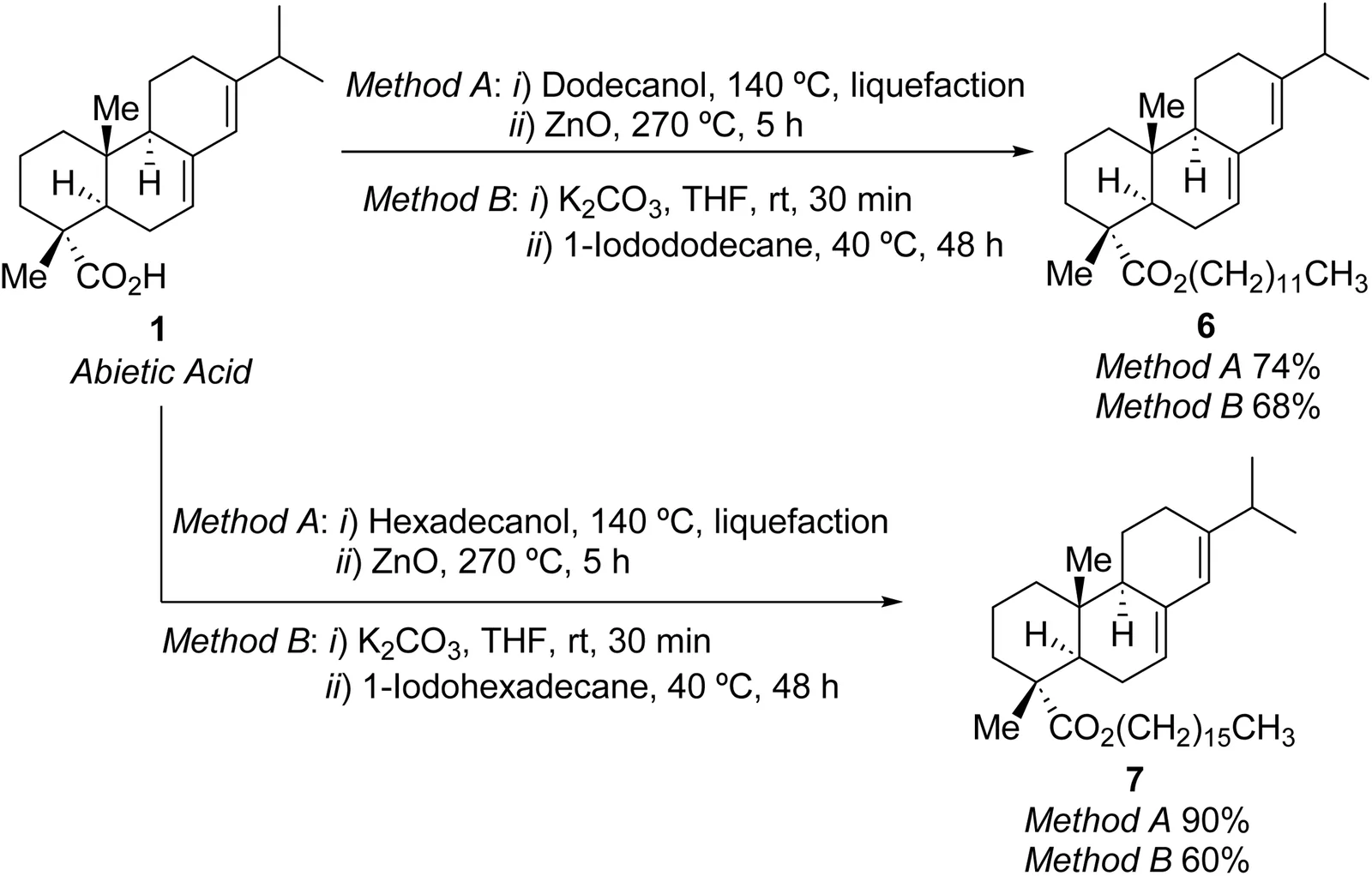
Synthesis of long alkyl chain abietates.

In addition to the above-described derivatives, two novel long-chain esters derived from abietic acid were synthesized. Esterification reactions were carried out using two different methods, method A and method B (Scheme 2). Method A is a solvent-free procedure involving the liquefaction of a mixture of abietic acid and dodecanol or hexadecanol, at 140 °C. Catalytic amounts of zinc oxide were added to the reaction mixture, which was then heated at 270 °C for 5 h. Following this procedure, new esters 6 and 7 were obtained in 74% and 90% yield, respectively (Scheme 2). Esters 6 and 7 were also obtained following method B in 68% and 60% yield, respectively. In this method, the solvent (THF) was pre-stirred with potassium carbonate for 30 min, after which abietic acid and 1-iodododecane or 1-iodohexadecane were added. The reaction mixture was then heated at 40 °C for 48 h (Scheme 2). Although method B takes longer reaction time (48 h *versus* 5 h), it proceeds under significantly milder conditions (40 °C) and does not require the harsh heating conditions (270 °C) associated with method A.

### HPLC-DAD analysis and *R*_f_ determination

Abietic acid (1), derivatives 2–5 and the new esters 6 and 7 were analyzed by HPLC-DAD using the chromatographic method described in the Experimental section. The *R*_f_ values for this set of compounds were calculated using *n*-hexane as eluent. Both abietic acid and alcohol 2 do not migrate under these conditions, but the other derivatives can be distinguished with this eluent. The retention time of each compound was determined by HPLC-DAD and the *R*_f_ values are collected in Table 1.

**Table 1:** Boltzmann-weighted gas-phase dipole moments (*µ*) and Gibbs solvation free energies (Δ*G*_solv_) for the title molecules in pentyl ethanoate (*ε* = 4.73 at 298 K) and *n*-octane (*ε* = 1.94 at 298 K), retention time in HPLC-DAD and *R*_f_ in silica gel using *n*-hexane as eluent

Molecule	*µ*/D	Δ*G*_solv_/kJ mol^−1^		*R*_s_	HPLC-DAD rt (min)	*R*_f_
		*ε* = 4.73	*ε* = 1.94			
1	1.83	−49.42	−42.54	−6.88	62.5	0.00
2	1.59	−43.81	−40.14	−3.67	57.6	0.00
3	5.42	−64.56	−55.73	−8.83	62.9	0.07
4	2.04	−38.55	−35.99	−2.56	54.1	0.85
5	0.47	−35.92	−37.28	1.36	56.1	0.78
6	2.32	−62.28	−60.70	−1.58	62.0	0.60
7	1.52	−78.39	−81.73	3.34	61.9	0.66

### Computational studies

Abietic acid derivatives 2–5 and new abietates 6 and 7, were used to develop a computational protocol for predicting the effect of structural modifications on the compatibility of abietic acid with environments of different polarity.

### Gas phase conformational behavior of the title molecules

**Fig. 1:**
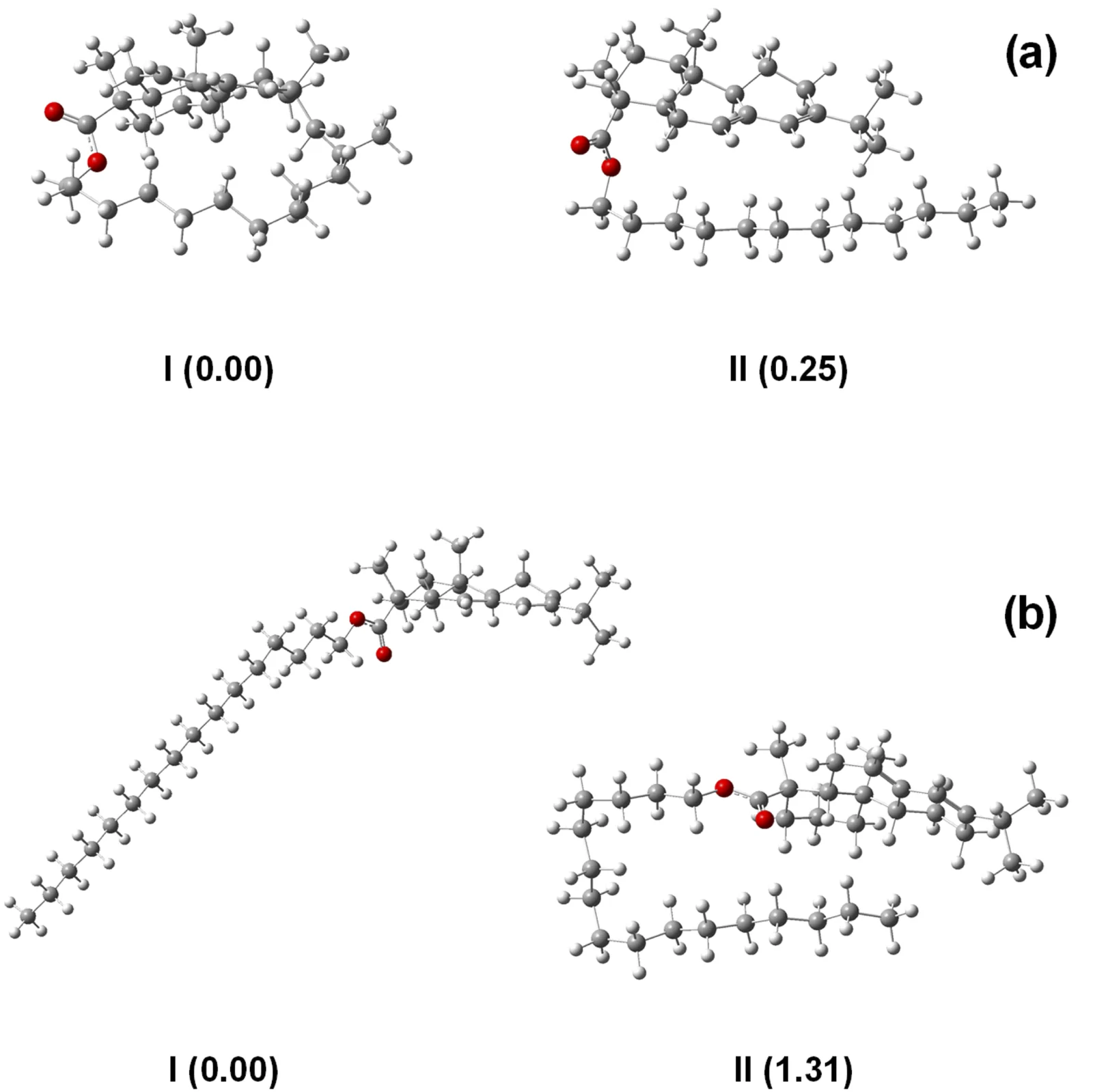
ωB97XD/def2-SVP optimized geometries of the two lowest-energy conformers of (a) dodecyl abietate (6) and (b) hexadecyl abietate (7) in the gas phase. Relative Gibbs energies (kJ mol^−1^) are given in parenthesis.

The lowest-energy conformations of the studied molecules generated through the molecular mechanics conformational search methodology described in the Experimental section were fully optimized using DFT at the ωB97XD/def2-SVP level of theory. Their relative Gibbs energies (*G*_gas,rel_), entropic terms (TS), Boltzmann populations at 298.15 K (*x*), dipole moments (*µ*) and polarizabilities (*α*) are presented in Tables S1–S7. The structures of the most stable conformers are displayed in Fig. 1 for molecules 6 and 7 and in Fig. S6–S10 for the remaining ones. Conformers are numbered with Roman numerals in order of increasing Gibbs energy.

The conformational behavior of the title molecules is determined primarily by the flexibility of the substituent attached to the abietadiene core and of the isopropyl fragment. Abietic acid (1) and abiet-7,13-dien-18-ol (2) exhibit relatively flexible conformational profiles, with nine and twelve low-energy conformers identified, respectively. The conformational space of 1 is dominated by conformers I and II, which have the carbonyl oxygen *cis* to the methyl group (O=C–C–CH_3_ ∼ 0°) and differ only in the orientation of the isopropyl fragment (see Fig. S6), with Δ*G* = 0.13 kJ mol^−1^ and Boltzmann populations of 21.8 and 20.7%, respectively (Table S1). For 2, conformer I is the most abundant species (Pop. = 26.1%) and features a *trans* arrangement of the hydroxyl oxygen relative to the CH_3_ group (O–C–C–CH_3_ ∼ 180°), with the OH moiety adopting a *gauche* orientation with respect to the adjacent C–C bond (H–O–C–C ∼77°). See Fig. S7 and Table S2 for details.

A different behavior is found for abiet-7,13-dien-18-yl *p*-toluenesulfonate (3), where only three relevant conformers are identified in the gas phase. The conformational equilibrium is clearly dominated by conformer I (Pop. = 62.4%), which is characterized by the *p*-toluenesulfonate group positioned alongside the abietadiene core (see Fig. S8). In contrast, conformations in which the sulfonate moiety is oriented outwards from the tricyclic core are highly destabilized (Δ*G* > 20 kJ mol^−1^) and were therefore excluded from further consideration. Iodide (4) occupies an intermediate position in the series, with six low-energy conformers identified. The most stable conformer represents almost 50% of the conformational mixture and is characterized by the iodine *trans* relative to the CH_3_ group (I–C–C–CH_3_ ∼ 180°) (see Fig. S9). Abieta-7,13-diene (5) is the simplest system in the series, both conformationally and in polarity, with three conformers arising from different orientations of the isopropyl fragment.

**Fig. 2:**
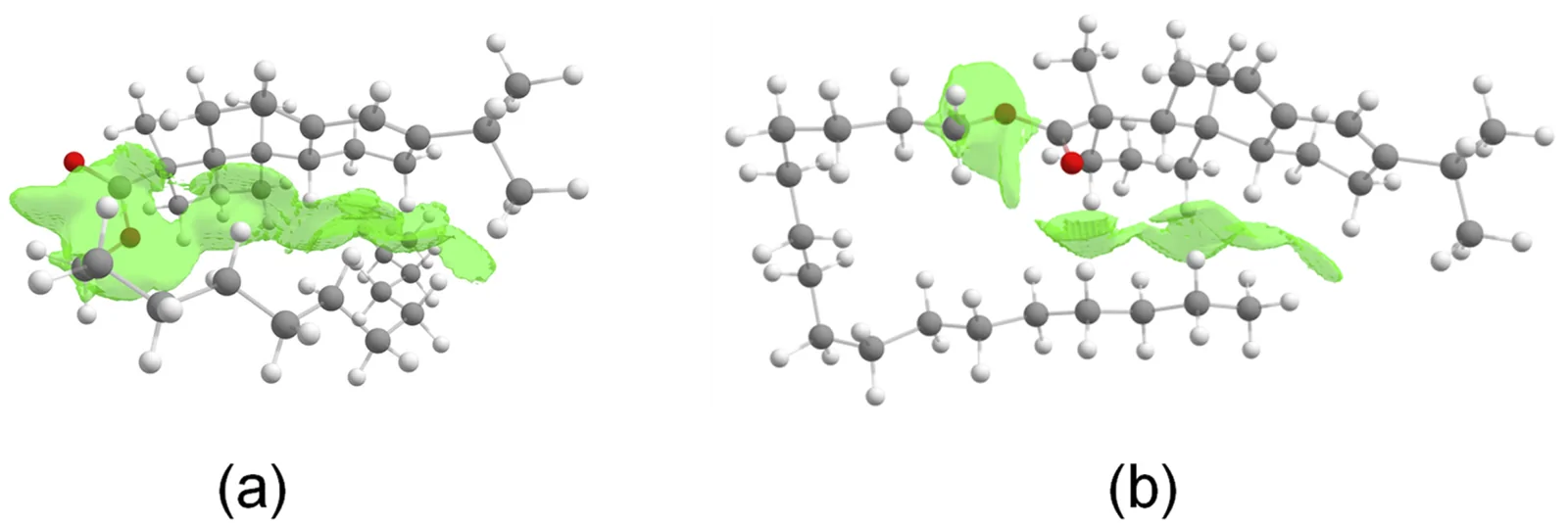
IGMH analysis of the most representative folded conformers of molecules 6 (conf. I, (a)) and 7 (conf. II, (b)), showing the δ*g*_inter isosurface (isovalue = 0.002 a.u.) describing the weak interfragment interactions between the abietadiene core (fragment 1) and the alkyl chain (fragment 2). The IGMH analysis was carried out using the Hirshfeld partition of the electron density.

As expected, the greatest conformational flexibility is observed for the long-chain esters dodecyl abietate (6) and hexadecyl abietate (7). Owing to the large number of conformers generated for these species in the molecular mechanics conformational search method, the subsequent DFT optimizations were restricted to the twenty lowest-energy structures, corresponding to energy windows of 5.0 kJ mol^−1^ for 6 and 4.2 kJ mol^−1^ for 7. Within the energy range examined, compound 6 populates exclusively folded conformations, *i.e.*, arrangements in which the alkyl chain bends back toward the abietadiene core (Fig. 1a). To better understand the origin of this preference, an Independent Gradient Model based on Hirshfeld partition (IGMH) analysis^16^ was performed for the most relevant folded conformer of 6, which corresponds to the global minimum (I). The generated map displayed in Fig. 2a reveals an extended green isosurface between the alkyl chain and the tricyclic framework, consistent with the existence of weak attractive intramolecular interactions. These results support the view that dispersion-type chain-core contacts contribute to the stabilization of the folded conformers of 6. To ensure that the absence of energetically relevant distended conformers for 6 was not an artifact of the conformational search protocol or of the relatively narrow energy window considered, representative distended conformations were optimized separately. In all cases, the resulting ωB97XD/def2-SVP optimized structures were found to lie by more than 16 kJ mol^−1^ above the global minimum, confirming that these conformers, in practical terms, do not contribute to the gas-phase population.

Regarding 7, the calculations reveal a markedly different conformational behavior, with distended conformers being more populated, although the folded ones also contribute appreciably (Table S7). For instance, the lowest-energy conformer (I) belongs to the distended family, whereas the second most stable conformer (II), only 1.3 kJ mol^−1^ higher in energy, adopts a folded geometry (Fig. 1b). The IGMH analysis of conformer II also shows a green isosurface between the alkyl chain and the abietadiene core, indicating the presence of weak attractive intramolecular through-space interactions (Fig. 2b). However, this interaction region is less extended than that observed for 6, which is consistent with the lower preference for the folded structures in this molecule.

The calculated Boltzmann-weighted dipole moments estimated for the seven molecules are given in Table 1. The tosylate derivative 3 exhibits the largest dipole moment (5.42 D), consistently with the high polarity of the sulfonyl group, whereas the hydrocarbon abieta-7,13-diene 5 shows the smallest value (0.47 D), in agreement with its apolar character. Intermediate dipole moments are estimated for abietic acid 1 (1.83 D), the corresponding alcohol 2 (1.59 D) and the iodide 4 (2.04 D), dodecyl abietate 6 (2.32 D), and hexadecyl abietate 7 (1.52 D). These results indicate that, for compounds 1–5 and 7, the molecular dipole is essentially governed by the intrinsic polarity of the substituent, although the iodide derivative 4 is predicted to be slightly more polar than the acid 1, alcohol 2 and ester 7. In contrast, the higher-than-expected dipole moment of compound 6 cannot be explained by the intrinsic polarity of the ester group alone; rather, it arises from the folded conformation of the abietane scaffold, which generates an anisotropic charge distribution and an atypically large molecular dipole.

### Solvent effect on the conformational behavior, Gibbs energies of solvation and relative solubility parameter

**Fig. 3:**
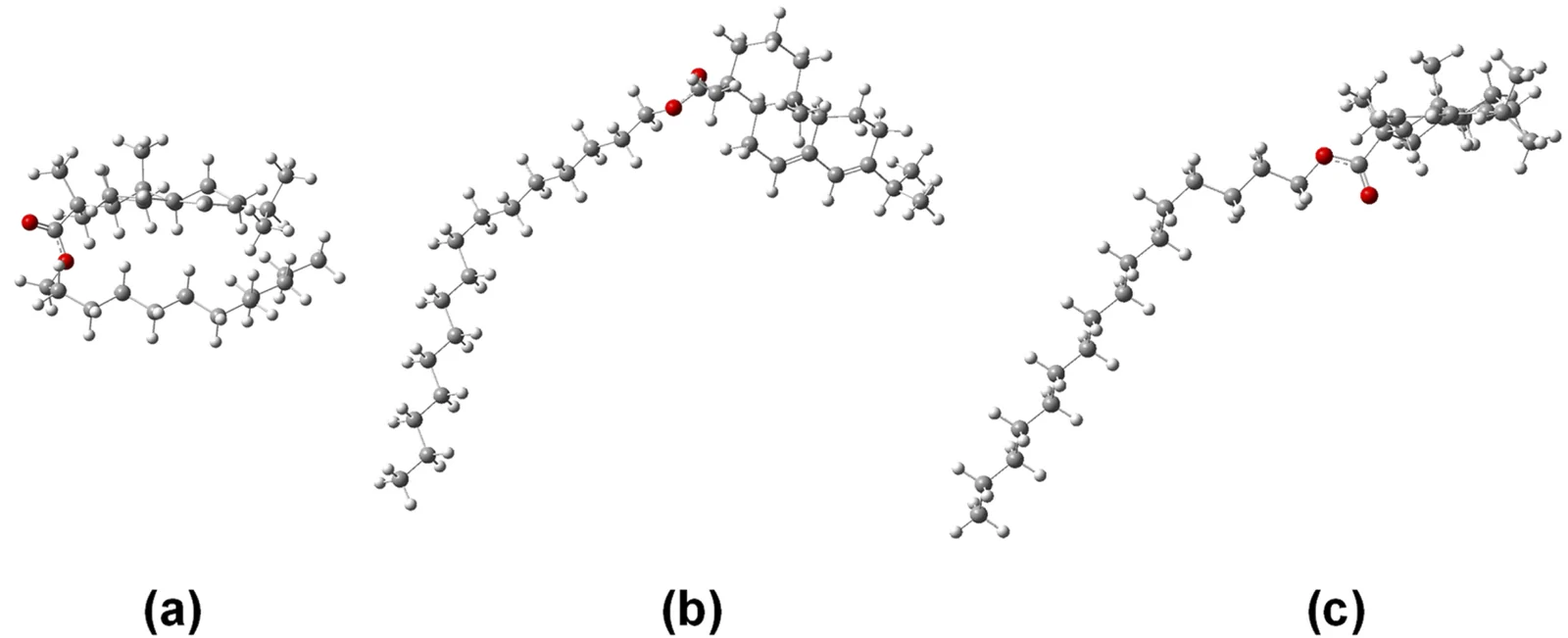
ωB97X-D/def2-SVP optimized geometries of the lowest-energy conformers of: (a) dodecyl abietate (6, conf. XV) in both *n*-octane and pentyl ethanoate; (b) hexadecyl abietate (7, conf. XV) in *n*-octane; and (c) hexadecyl abietate (7, conf. I) in pentyl ethanoate. Conformers are designated by Roman numerals according to their gas-phase Gibbs free energy stability ranking (see Tables S6 and S7 for details).

The conformers identified and optimized in the gas phase were also reoptimized in two dielectric media: pentyl ethanoate (*ε* = 4.73 at 298 K) and *n*-octane (*ε* = 1.94 at 298 K). The resulting relative energies are reported in Tables S1–S7, while the lowest-energy conformers in each medium are depicted in Fig. 3 and S6–S10. For compounds 2, 4, and 5, the gas-phase global minimum remains the most stable conformer in both media. By contrast, for compounds 1, 3, 6, and 7, the lowest-energy conformer changes upon solvation, showing that differences of Gibbs energy of solvation of the conformers are large enough to change the gas-phase stability order. For the flexible compounds 6 and 7, this effect is especially marked. Small differences in gas-phase Gibbs energy are counterbalanced by significantly varied solvation free energies, effectively reordering the conformers' stability order upon solvation.

The population-weighted average Gibbs free energies of solvation (Δ*G*_solv_) calculated for the different molecules in two solvents of different polarity (pentyl ethanoate and *n*-octane) are given in Table 1 (the values estimated for the individual conformers are provided in Tables S1–S7). Abietic acid (1) is more strongly stabilized in pentyl ethanoate than in *n*-octane by ∼7 kJ mol^−1^. This difference is consistent with the presence of the carboxylic acid group, which favors stabilization in the more polar medium. A comparable trend is observed for abiet-7,13-dien-18-ol (2) and 18-iodoabiet-7,13-diene (4), although to a lesser extent (*ca.* 3–4 kJ mol^−1^), while abiet-7,13-dien-18-yl *p*-toluenesulfonate (3) exhibits the largest stabilization difference (∼9 kJ mol^−1^), reflecting the pronounced polarity of the sulfonate moiety.

A distinct pattern is found for abieta-7,13-diene 5, dodecyl abietate 6, and hexadecyl abietate 7: compound 6 is only marginally more stabilized in pentyl ethanoate, whereas 5 and 7 are slightly more stabilized in *n*-octane. This behavior is consistent with expectations, as the removal of a polar functionality or the introduction of longer hydrophobic chains enhances compatibility with less polar media.

**Fig. 4:**
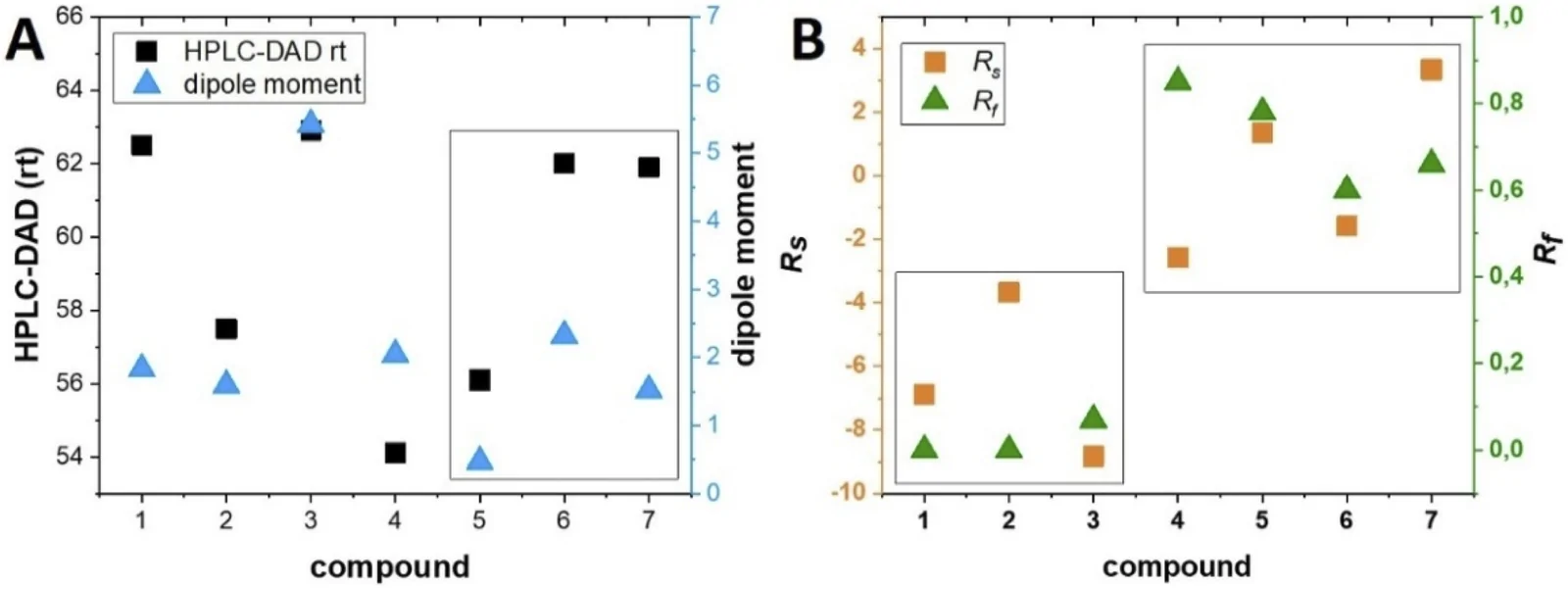
(A) HPLC-DAD retention time *vs.* calculated dipole moment and (B) *R*_s_*vs. R*_f_ determine in silica gel using *n*-hexane as eluent.

The calculated dipole moment values and the HPLC-DAD retention times are presented in Fig. 4, panel A. In gradient reverse-phase HPLC, elution order is not determined solely by analyte polarity but is also influenced by interaction kinetics, gradient profile, and secondary retention mechanisms. As a result, even relatively polar compounds may elute at later times. In the present method, the mobile phase is highly polar (water–methanol) only during the initial 4 minutes (Fig. S5). This short period is insufficient for the efficient elution of polar compounds, particularly if they engage in secondary interactions. As the gradient progresses (4–55 minutes), the increasing methanol content reduces the overall polarity of the mobile phase while enhancing its elution strength, promoting the desorption of more strongly retained (typically less polar) compounds. However, polar compounds that remain transiently adsorbed or exhibit slow migration may not elute during the early phase. Instead, they are carried off the column only at later stages of the gradient, when elution strength is sufficiently high to overcome these interactions (after 55 minutes).

The conversion of abietic acid into the corresponding hydroxymethyl derivative 2 leads to a decrease in the dipole moment, which in turn results in a shorter retention time under the chromatographic conditions employed. Tosylation of the hydroxyl group induces a pronounced change in the dipole moment: tosylate derivative 3 exhibits a higher dipole moment than the parent carboxylic acid, restoring the retention time close to that of the abietic acid. However, under the HPLC conditions employed, its longer retention time is unlikely to arise from polarity alone. Rather, the increased retention of compound 3 can be attributed to the introduction of the bulky *p*-toluenesulfonyl group, which increases molecular size, polarizability, aromatic character, and hydrophobic surface area, enhancing interactions with the C18 stationary phase.

A comparison of the carboxylic acid 1 with its ester derivatives highlights the influence of the alkyl chain length. Notably, the dodecyl ester 6 displays a calculated dipole moment higher than that of abietic acid, which was unexpected. However, this result correlates well with the retention times obtained by HPLC. Contrary to the anticipated decrease in polarity associated with conversion of a carboxylic acid into an ester, no such decrease was observed experimentally or predicted computationally. As mentioned above, within the examined energy range, the optimized geometries of dodecyl abietate (6) reveal an exclusive preference for folded conformations, characterized by the alkyl chain bending back toward the abietadiene core (Fig. 1). This folded geometry produces a differentiated polarity distribution, featuring a more polar region where the polar functional group is exposed and a contrasting nonpolar region dominated by the alkyl chain. As a result, dodecyl abietate 6 exhibits a pseudo-amphiphilic character.

Increasing the alkyl chain length leads to a clear decrease in polarity, as reflected in the calculated dipole moments (2.32 D for 6 and 1.52 D for 7). This trend is consistent with the aforementioned calculations, which reveal a markedly different conformational behavior between dodecyl abietate (6) and hexadecyl abietate (7) (Fig. 1), the latter exhibiting both folded and extended conformations with comparable stability.

The difference of Gibbs free energies of solvation (Δ*G*_solv_) in moderately polar (Δ*G*^P^) and nonpolar media (Δ*G*^A^) provides a simple measure of the balance of stabilization across these environments. More negative values indicate greater stability in polar media, whereas with less negative values correspond to increased stability in apolar environments.

A qualitative correlation is observed between Δ*G*^P^ − Δ*G*^A^ (*R*_s_) and experimental *R*_f_ values obtained from thin-layer chromatography (TLC) measurements (Fig. 4, panel B), which reflect the affinity of the compounds for the polar silica gel stationary phase. In general, the compounds with high negative *R*_s_ values interact more strongly with the polar stationary phase and thus show lower mobility, whereas compounds with less negative *R*_s_ values exhibit weaker retention and higher *R*_f_ values. TLC on silica gel using hexane as the eluent, does not effectively separate polar compounds (*e.g.*1, 2 and 3) which remain at the baseline (*R*_f_ ∼0), but allows differentiation of the more apolar compounds. The most positive *R*_s_ value obtained corresponds to the ester with the longest alkyl chain 7, which is consistent with its pronounced apolar character. This clearly distinguishes the hexadecyl abietate (7) (*R*_f_ = 0.66) from the dodecyl abietate (6) (*R*_f_ = 0.60), the latter being more stabilized in polar systems and therefore exhibiting a negative Δ*G*^P^ − Δ*G*^A^ value. This *R*_s_ descriptor provides a predictive tool for evaluating the compatibility of abietic acid derivatives in different environments.

## Conclusions

A series of chemical modifications of the carboxylic acid moiety of abietic acid was performed to obtain derivatives bearing functional groups with distinct polarities. In total, four non-carbonyl derivatives and two novel long alkyl chain ester derivatives were synthesized in good yields. Esterification with long-chain alcohols was successfully achieved using two complementary methodologies: a solvent-free protocol employing ZnO as a catalyst at 270 °C, and a milder approach using potassium carbonate at 40 °C in THF.

The hydrophilic–hydrophobic balance of the abietic acid derivatives was primarily investigated through computational approaches. Extensive molecular mechanics conformational searches were first performed, followed by full geometry optimizations at the DFT level of theory for abietic acid and its derivatives. Dipole moments (*µ*) relative Gibbs free energies (Δ*G*), among other molecular properties, were calculated in polar and apolar solvents providing a detailed and consistent description of the polarity profiles of the derivatives. The theoretical predictions were subsequently corroborated by experimental analyses using HPLC-DAD and thin-layer chromatography (TLC).

Interestingly, dodecyl abietate (6) revealed an unexpected behaviour. Contrary to the anticipated decrease in polarity associated with conversion of a carboxylic acid into an ester, no such reduction was observed, either experimentally or computationally. This abietate displays a calculated dipole moment higher than that of abietic acid, in agreement with its HPLC retention behaviour. Conformational analysis of dodecyl abietate revealed an exclusive preference for folded geometries, in which the alkyl chain bends back toward the abietadiene. IGMH analysis attributed this folding to weak, attractive intramolecular interactions, supporting the view that dispersion-type chain-core contacts stabilize these folded conformers. This folding induces an anisotropic polarity distribution, with a localized polar region associated with the ester functional group and a distinct nonpolar domain arising from the alkyl chain. Consequently, dodecyl abietate can be described as exhibiting a pseudo-amphiphilic character.

Overall, the combined experimental and computational approach provides a reliable and predictive framework for evaluating the compatibility of abietic acid derivatives in environments of differing polarity, facilitating the rational design of systems where fine tuning of hydrophilic *vs.* hydrophobic character is required.

## Experimental section

### Synthesis

#### General information

^1^H, ^13^C NMR spectra were recorded with a Bruker Avance III instrument operating at 400 and 100 MHz, respectively. The solvent was deuterochloroform (CDCl_3_). Chemical shifts for ^1^H and ^13^C NMR spectra are referenced relative to TMS (*δ* = 0 ppm) and are listed in parts per million (ppm). Coupling constants (*J*) are in Hz. Infrared spectra (IR) were recorded in a Fourier Transform spectrometer coupled with a diamond Attenuated Total Reflectance (ATR) sampling accessory. High-resolution mass spectra (HRMS; mass analyzer: quadrupole) were recorded on a Bruker microTOF-Focus mass spectrometer equipped with an electrospray ionization time-of-flight source. Melting points were recorded in a Falc apparatus with open glass capillaries and were uncorrected. Thin Layer Chromatography (TLC) was performed using precoated silica gel plates. Column chromatography was performed using silica gel 60 as a stationary phase. Preparative Layer Chromatography (PLC) was performed in precoated silica gel 60 F254 plates with 0.5 mm thickness. The HPLC-DAD analysis was carried out using the Hitachi LaChrom Elite HPLC system with an L-2455 DAD, a Merck Purospher® STAR RP-18 endcapped (5 µm) stationary-phase column, an L-23000 column oven, an L-2130 pump, and an L-2200 autosampler.

#### General procedure for the esterification of abietic acid

Method A: a mixture of abietic acid (1.00 g, 3.31 mmol) and dodecanol (0.62 g, 3.31 mmol) or hexadecanol (0.80 g, 3.31 mmol) was heated at 140 °C, until liquefaction of both reagents. Zinc oxide (0.06 g, 0.33 mmol) was added, and the mixture was heated at 270 °C, under stirring, for 5 h. Upon completion, monitored by TLC, the mixture was left to cool to room temperature. The product was purified by column chromatography on silica gel.

Method B: a suspension of K_2_CO_3_ (3.21 g, 23.2 mmol) in dry tetrahydrofuran (45 mL) was stirred at room temperature for 30 min. After which abietic acid (2.00 g, 6.62 mmol) and 1-iodododecane (4.09 mL, 16.6 mmol) or 1-iodohexadecane (5.22 mL, 16.6 mmol) were added. The reaction mixture was heated at 40 °C for 48 h. Upon completion, monitored by TLC, the solid in suspension was filtered, the filtrate was diluted with diethyl ether (150 mL), and washed with water (3 × 100 mL). The organic extracts were dried and the solvent evaporated off. The product was purified by column chromatography on silica gel.

##### Dodecyl abietate (6)

Purification of the crude product by column chromatography on silica gel [hexane (100%)] gave ester 6 (method A: 1.15 g, 74%; method B: 2.12 g, 68%) as a colorless oil. [*α*]^20^_D_ = −35° (*c* 1, CHCl_3_). IR (ATR): *ν* 1106, 1146, 1181, 1241, 1465, 1723, 2853, 2923 cm^−1^. ^1^H NMR (400 MHz, CDCl_3_): *δ* 0.82 (s, 3H), 0.88 (pseudo t, *J* = 6.75 Hz, 3H), 1.00 (d, *J* = 4.0 Hz, 3H), 1.02 (d, *J* = 4.0 Hz, 3H), 1.23–1.31 (m, 24H), 1.57–1.62 (m, 4H), 1.73–1.83 (m, 3H), 1.85–1.96 (m, 2H), 2.01 (m, 4H), 2.22 (septet, *J* = 6.75 Hz, 1H), 3.99–4.05 (m, 2H), 5.35–5.36 (m, 1H), 5.77 (s, 1H) ppm. ^13^C NMR (100 MHz, CDCl_3_): *δ* 14.2, 14.3, 17.2, 18.3, 21.0, 21.5, 22.6, 22.8, 25.8, 26.1, 27.6, 28.8, 29.4, 29.5, 29.7 (2C), 29.8 (2C), 32.1, 34.7, 35.0, 37.3, 38.5, 45.3, 46.7, 51.1, 64.8, 120.8, 122.6, 135.6, 145.3, 178.7 ppm. HRMS (ESI) *m*/*z*: [M–H]^+^ calcd for C_32_H_53_O_2_ 469.4040; found 469.4033.

##### Hexadecyl abietate (7)

Purification of the crude product by column chromatography on silica gel [hexane (100%)] gave ester 7 (method A: 1.57 g, 90%; method B: 2.09 g, 60%) as a colorless oil. [*α*]^20^_D_ = −35° (*c* 1, CHCl_3_). IR (ATR): *ν* 1106, 1181, 1242, 1465, 1724, 2853, 2922 cm^−1^. ^1^H NMR (400 MHz, CDCl_3_): *δ* 0.82 (s, 3H), 0.88 (pseudo t, *J* = 6.6 Hz, 3H), 1.00 (d, *J* = 3.2 Hz, 3H), 1.02 (d, *J* = 3.2 Hz, 3H), 1.23–1.31 (m, 31H), 1.57–1.62 (m, 4H), 1.73–1.83 (m, 4H), 1.86–1.96 (m, 2H), 2.01 (m, 4H), 2.22 (septet, *J* = 7.2 Hz, 1H), 3.98–4.05 (m, 2H), 5.35–5.36 (m, 1H), 5.77 (s, 1H) ppm. ^13^C NMR (100 MHz, CDCl_3_): *δ* 14.2, 14.3, 17.2, 18.3, 21.0, 21.6, 22.6, 22.8, 25.8, 26.1, 27.6, 28.8, 29.4, 29.5, 29.7 (2C), 29.8 (2C), 29.9 (4C), 32.1, 34.7, 35.0, 37.3, 38.5, 45.3, 46.7, 51.1, 64.8, 120.9, 122.6, 135.6, 145.4, 178.7 ppm. HRMS (ESI) *m*/*z*: [M–H]^+^ calcd for C_36_H_61_O_2_ 525.4666; found 525.4659.

### Computational methods

To identify the most stable conformers of abietic acid derivatives, initial conformational searches were performed using the Conformers plugin implemented in MarvinSketch 23.16 (ChemAxon).^17^ The conformer generation procedure is based on the Generate3D algorithm,^18^ and follows the approach described in ref. 19. The generated conformers are optimized and ranked using the MMFF94 force field.^20^ A total of 2000 conformers were initially generated. Additional tests with larger ensembles of 10 000 and 20 000 conformers confirmed that sampling with 2000 conformers was sufficient to identify all relevant low-energy conformations. From the resulting ensemble, conformers within a threshold of 15 kJ mol^−1^ from the global minimum were selected for further refinement. These structures were subsequently optimized in gas phase at the Density Functional Theory (DFT) level using the long-range corrected hybrid ωB97X-D functional with empirical dispersion corrections^21^ in combination with the def2-SVP split-valence polarization basis set.^22^ Harmonic vibrational frequency calculations were performed at the same level of theory to characterize the nature of each stationary point and to confirm that the selected optimized structures correspond to true minima on the potential energy surface (*i.e.*, no imaginary frequencies). From these calculations, the relative Gibbs free energies of the different conformers of each molecule (*G*_gas,rel_) at 298.15 K were obtained, and their populations (*x*_gas_) were estimated according to the Boltzmann distribution. Subsequently, the geometries of these conformers were re-optimized in solution to account for bulk solvent effects and the corresponding relative Gibbs free energies (*G*_sol,rel_) and Boltzmann population (*x*_sol_) were reassessed. Two solvents of different polarity were selected: pentyl ethanoate (*ε* = 4.7297 at 298 K) and *n*-octane (*ε* = 1.9406 at 298 K). Solvent effects were modeled using the integral equation formalism variant of the polarized continuum model (IEF-PCM) within the self-consistent reaction field (SCRF) framework, employing the radii and non-electrostatic terms for Truhlar and coworkers' solvation model based on density (SMD),^23^ which is recommended for computing free energies of solvation. All DFT calculations both in the gas phase and within the continuum dielectric medium were performed with the Gaussian16 software (version B.01).^24^ ChemCraft (version 1.6) was used to visualize the optimized structures.^25^

The Gibbs free energy of solvation for each molecule (Δ*G*_solv_) was then estimated as the difference between the population-weighted average Gibbs energies in solution and in the gas phase, according to the following expression:1
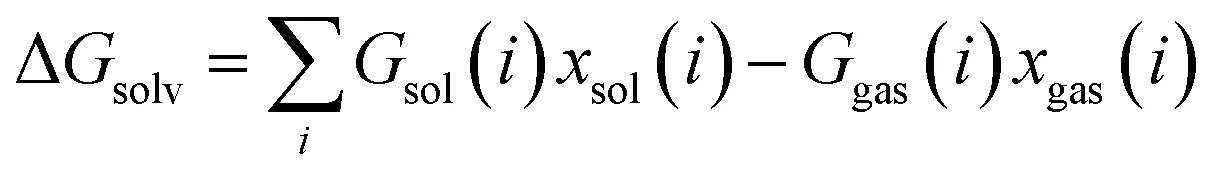
where the summation runs over all sampled conformers *i* of a given molecule. It is important to note that entropy contributions in condensed phase are not described rigorously by standard harmonic treatments combined with implicit-solvent models, and that such approaches may underestimate the entropy contribution in solution,^26^ thereby affecting the computed *G*_sol_ and consequently the Δ*G*_solv_ values estimated from eqn (1). Therefore, the latter are discussed mainly in terms of relative trends between related compounds and solvents rather than as absolute values. Additionally, continuum model does not account explicitly for specific solute–solvent interactions. Although explicit microsolvation may improve the description of such effects, its rigorous treatment in the present systems would require extensive sampling of solvent arrangements and, for the more flexible molecules 6 and 7, consideration of multiple relevant conformers, which is highly challenging. Therefore, a uniform IEF-PCM/SMD treatment was adopted here as a first-order approach for comparing relative solvation trends.

Based on these results, a Relative Solubility (*R*_s_) parameter was defined as the difference between the free energy of solvation in the more polar (P) pentyl ethanoate (Δ*G*^P^_solv_) and the more apolar (A) *n*-octane (Δ*G*^A^_solv_).

2 *R*_s_ = Δ*G*^P^_solv_ − Δ*G*^A^_solv_

In addition to the Gibbs free energies of solvation, other molecular properties, namely dipole moments (*µ*), polarizabilities (*α*), and entropic terms (TS), collectively denoted here as *X*, were estimated for both the gas phase and solution. For a given molecule, the macroscopic property was calculated as the Boltzmann-weighted average of the values computed for the individual conformers, according to the following expression:3
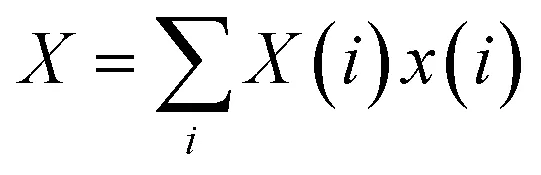
where *X*(*i*) represents the specific property of conformer *i*, and *x*(*i*) is its corresponding molar fraction (population) in the respective phase (gas or solution).

Independent Gradient Model based on Hirshfeld partition (IGMH) analyses^16^ were performed for selected folded conformers of molecules 6 and 7 using the Multiwfn program,^27^ on geometries fully optimized at ωB97XD/def2-SVP level.

### HPLC-DAD

High-performance liquid chromatography with diode-array detection (HPLC-DAD) was performed using a Purospher STARRP-18 endcapped (5 µm) column from Merck, an L-2130 Pump, L-2200 autosampler and L-2455 diode array detector. A chromatographic method based on a binary gradient elution system was used. The mobile phase consisted of solvent A (methanol/water, 55 : 45 v/v) and solvent B (methanol). The elution program was as follows: isocratic at 100% A from 0 to 4 min; linear gradient from 4 to 24 min to reach 60% A and 40% B (v/v); gradient from 24 to 53 min to 100% B, held isocratically until 55 min; followed by a gradient to 50% A and 50% B at 60 min, and finally returning to 100% A at 65 min. The column temperature was maintained at 40 °C, with a flow rate of 0.6 mL min^−1^ and an injection volume of 20 µL of an ethanolic solution. Chromatograms were recorded at 254 nm.

## Author contributions

Jéssica Macedo: investigation, formal analysis. Pedro Miranda: investigation, formal analysis. Américo Alves: methodology, investigation, formal analysis, validation. Cláudia Alves: investigation, formal analysis. Nuno Alves: investigation. Dina Farinha: investigation. Susana M. M. Lopes: writing – original draft, investigation, formal analysis. Catarina Gonçalves: investigation. Anna B. Brito: investigation, formal analysis. Vera A. Gamboa: investigation, formal analysis. Nelson A. M. Pereira: investigation, formal analysis, supervision. A. J. Lopes Jesus: writing – review and editing, writing – original draft, validation, methodology, investigation, formal analysis. Bernardo A. Nogueira: validation, methodology, investigation, formal analysis. Licínia L. G. Justino: writing – review and editing, writing – original draft, validation, methodology, investigation, formal analysis. Cláudio M. Nunes: writing – review and editing, validation, methodology, investigation, formal analysis. Dina Murtinho: writing – review and editing, writing – original draft, methodology, formal analysis, supervision. Marta Pineiro: writing – review and editing, writing – original draft, methodology, formal analysis, supervision. A. Mendes Ferreira: validation, formal analysis, funding acquisition, conceptualization, project administration. Rui Fausto: writing – review and editing, supervision, funding acquisition, conceptualization. Teresa M. V. D. Pinho e Melo: writing – review and editing, methodology, formal analysis, supervision, funding acquisition, conceptualization. All authors have given approval to the final version of the manuscript.

## Conflicts of interest

There are no conflicts to declare.

## Supplementary Material

RA-016-D6RA04565K-s001

## Data Availability

The data supporting this article have been included as part of the supplementary information (SI). Supplementary information: synthetic procedures for compounds 2–5. NMR spectra and HRMS of new compounds 6 and 7. HPLC-DAD solvent gradient. Relative Gibbs energy, calculated dipole moment and optimized geometries for compounds 1–7. See DOI: https://doi.org/10.1039/d6ra04565k.
