# Supplementary material for: Structural modifications of abietic acid and a computational protocol to predict their compatibility in different environments
Source: RSC Adv. 2026 Aug 6;16(41):48080–9. doi: 10.1039/d6ra04565k (PMC13446451; doi:10.1039/d6ra04565k)
Supplement: Supplementary file 1 [file RA-016-D6RA04565K-s001.pdf]

Supplementary Information (SI) for RSC Advances.
This journal is © The Royal Society of Chemistry 2026

Structural modifications of abietic acid and a computational protocol to predict their compatibility in different environments

Jéssica Macedo,[1#] Pedro Miranda,[1#] Américo Alves,[1] Cláudia Alves,[1] Nuno Alves,[1] Dina Farinha,[1] Susana M. M. Lopes,[1] C. Gonçalves,[1] Anna B. Brito,[1] Vera A. Gamboa,[2] Nelson A. M. Pereira,[2] A. J. Lopes Jesus,[1] Bernardo A. Nogueira,[1] Licínia L. G. Justino,[1] Cláudio M. Nunes,[1] Dina Murtinho,[1] Marta Pineiro,[1] A. Mendes Ferreira,[2] Rui Fausto,[1] Teresa M. V. D. Pinho e Melo[1]*

[1]CQC-IMS, University of Coimbra, Rua Larga, 3004-535 Coimbra, Portugal
[2]United Resins, S.A, Figueira da Foz, Portugal
*tmelo@ci.uc.pt

Supporting Information

Table of Contents

1. Synthetic Procedures for compounds **2-5** ……………………………………………...…. 2
2. NMR and HRMS of new compounds ………………………………………………….. 5
3. Computational Studies …………………………………………………………………. 9
4. References ……………………………………………………………………… ………125

1. Synthetic Procedures for compounds 2-5

General Information: ^{1}H, ^{13}C NMR spectra were recorded with a Bruker Avance III instrument operating at 500 and 125 MHz, respectively. The solvent was deuterochloroform ($CDCl_3$). Chemical shifts for ^{1}H and ^{13}C NMR spectra are referenced relative to TMS (δ = 0 ppm) and are listed in parts per million (ppm). Coupling constants (*J*) are in Hz. Infrared spectra (IR) were recorded in a Fourier Transform spectrometer. High-resolution mass spectra (HRMS; mass analyzer: quadrupole) were obtained on a spectrometer with electrospray ionization (ESI).

Abiet-7,13-dien-18-ol (**2**)

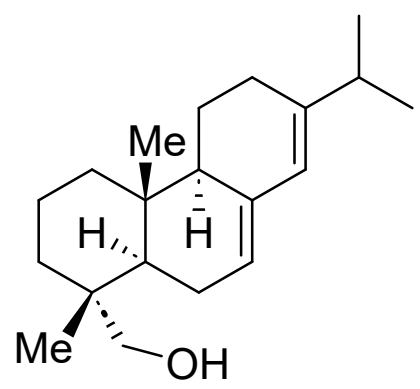

A solution of abietic acid (**1**) (4.53 g, 15 mmol, 80%) in dry tetrahydrofuran (40 mL) was added dropwise to a stirred and ice-cold suspension of $LiAlH_4$ (1.14 g, 30 mmol) in dry tetrahydrofuran (30 mL). Upon complete addition, the reaction mixture was refluxed for 4 h. After completion, monitored by TLC, the reaction mixture was cooled to 0 ºC and carefully quenched by sequential addition of H_2O (1.14 mL), aqueous solution NaOH 15 % (1.14 mL), and finally H_2O (3.42 mL). The solids were filtered over a Celite pad, and the Celite washed with THF (30 mL). The filtrate was dried over anhydrous Na_2SO_4 and the solvent evaporated off. Purification of the crude product by column chromatography on silica gel [ethyl acetate/hexane (1:4)] gave compound **2** (3.81 g, 88%) as a white solid. All the analytical data is in agreement with literature.[1] ^{1}H NMR (500 MHz, $CDCl_3$): δ 0.83 (s, 3H), 0.87 (s, 3H), 1.01 (dd, *J* = 7.00 and 5.00 Hz), 1.18-1.23 (m, 1H), 1.32-1.43 (m, 2H), 1.51-1.62 (m, 4H), 1.78-1.90 (m, 3H), 1.94-2.08 (m, 4H), 2.22 (septet, *J* = 7.00 Hz, 1H), 3.12 (d, *J* = 11.0 Hz, 1H), 3.35 (d, *J* = 11.0 Hz, 1H), 5.40 (s, 1H), 5.77 (s, 1H) ppm. ^{13}C NMR (125 MHz, $CDCl_3$): δ 14.4, 17.8, 18.3, 21.0, 21.5, 22.8, 23.9, 27.6, 34.8, 35.0, 35.8, 37.6, 39.0, 43.8, 50.9, 72.2, 121.0, 122.6, 135.7, 145.3 ppm.

Abiet-7,13-dien-18-yl *p*-toluenesulfonate (**3**)

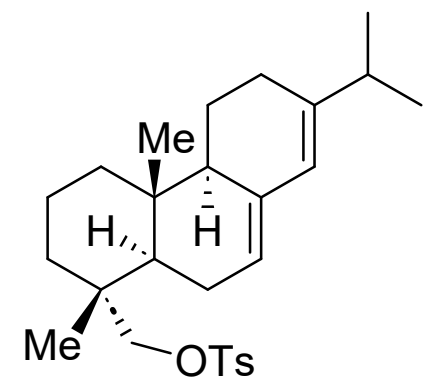

A solution of *p*-toluenesulfonyl chloride (0.41 g, 2.12 mmol) in dry dichloromethane (10 mL) was added to a stirred and ice-cold mixture of alcohol **2** (0.31 g, 1.06 mmol) with 4-dimethylaminopyridine (0.26 g, 2.12 mmol). Upon complete addition, the reaction mixture was refluxed for 4 h. Upon completion, monitored by TLC, the reaction mixture was left to cool to room temperature, ethyl acetate (15 mL) and saturated aqueous solution of $NaHCO_3$ (15 mL) were added. The reaction mixture was left, under stirring, for 30 min. The layers were separated and the organic extracts washed with saturated aqueous solution of $NaHCO_3$ (2 x 15 mL) and with brine (15 mL). The organic extracts were dried over anhydrous Na_2SO_4 and the solvent evaporated off. Purification of the crude product by column chromatography on silica gel [ethyl acetate/hexane (1:8)] gave compound **3** (0.42 g, 90%) as a gummy liquid. All the analytical data is in agreement with literature.[1] ^{1}H NMR (500 MHz, $CDCl_3$): δ 0.69 (s, 3H), 0.78 (s, 3H), 0.92 (d, *J* = 5.0 Hz, 3H), 0.93 (d, *J* = 5.0 Hz, 3H), 1.10-1.14 (m, 1H), 1.24-1.30 (m, 2H), 1.35-1.46 (m, 3H), 1.64-1.75 (m, 4H), 1.95-1.98 (m, 2H), 2.13 (septet, *J* = 7.0 Hz, 1H), 2.35 (s, 3H), 3.45 (d, *J* = 9.5 Hz, 1H), 3.59 (d, *J* = 9.5 Hz, 1H), 5.17 (s, 1H), 5.66 (s, 1H), 7.24 (d, *J* = 8.0 Hz, 2H), 7.67 (d, *J* = 8.0 Hz, 2H) ppm. ^{13}C NMR (125 MHz, $CDCl_3$): δ 14.1, 17.8, 20.9, 21.4, 22.6, 23.6, 27.4, 34.7, 34.9, 35.7, 36.8, 38.4, 43.6, 50.6, 78.0, 120.2, 122.4, 127.9, 129.8, 133.0, 135.4, 144.6, 145.1 ppm.

18-Iodo abiet-7,13-diene (**4**)

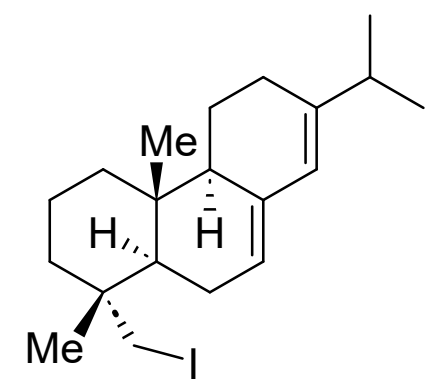

Triphenylphosphine (2.73 g, 10.4 mmol) and sublimated iodine (2.82 g, 11.1 mmol) were added to a solution of alcohol **2** (1.55 g, 3.47 mmol) in dry toluene (20 mL). The reaction mixture was stirred at room temperature for 10 min, after which imidazole (1.4 g, 20.6 mmol) was added, and heated at 70 ºC for 30 min. Upon completion, monitored by TLC, the solid in suspension was filtered, and the solvent of the filtrate evaporated off. Purification of the crude product by

column chromatography on silica gel [hexane (100%)] gave compound **4** (0.64 g, 46%) as a yellow oil. All the analytical data is in agreement with literature.[1] ^{1}H NMR (500 MHz, $CDCl_3$): δ 0.81 (s, 3H), 1.00 (d, *J* = 4.0 Hz, 3H), 1.02 (d, *J* = 4.0 Hz, 3H), 1.08 (s, 3H), 1.19-1.26 (m, 2H), 1.30-1.33 (m, 1H), 1.38-1.43 (m, 1H), 1.51-1.57 (m, 3H), 1.78-1.85 (m, 2H), 1.90-1.99 (m, 3H), 2.07-2.09 (m, 2H), 2.22 (septet, *J* = 7.0 Hz, 1H), 3.20 (s, 2H), 5.38-5.39 (m, 1H), 5.78 (s, 1H) ppm. ^{13}C NMR (125 MHz, $CDCl_3$): δ 13.7, 18.7, 19.0, 21.0, 21.5, 22.9, 23.6, 27.6, 28.7, 35.0, 35.1, 35.5, 38.9, 39.9, 47.1, 50.8, 120.5, 122.5, 135.7, 145.5 ppm.

Abieta-7,13-diene (**5**)

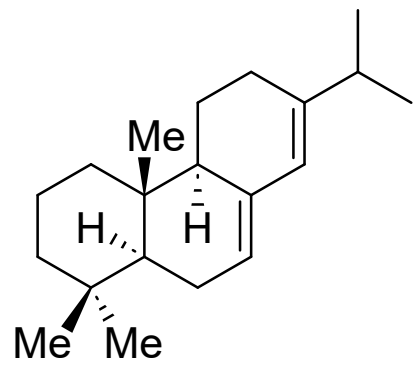

A solution of tosylate **3** (0.41 g, 0.93 mmol), sodium iodide (0.70 g, 4.63 mmol) and zinc dust (0.61 g, 9.30 mmol) in *N*,*N*,*N*,*N*-hexamethylphosphoramide (3 mL) was heated at 105 ºC for 18 h. Upon completion, monitored by TLC, the reaction mixture was dissolved in water (50 mL) and the aqueous layer extracted with ethyl acetate (3 x 50 mL). The combined organic extracts were washed with water (50 mL) and brine (2 x 50 mL). The organic extracts were dried over anhydrous Na_2SO_4 and the solvent evaporated off. Purification of the crude product by column chromatography on silica gel [ethyl acetate/hexane (1:10)] gave compound **5** (0.17 g, 69%) as a white oil. All the analytical data is in agreement with literature.[2,3] ^{1}H NMR (500 MHz, $CDCl_3$): δ 0.79 (s, 3H), 0.86 (s, 3H), 0.91 (s, 3H), 1.00 (d, *J* = 5.0 Hz, 3H), 1.02 (d, *J* = 5.0 Hz, 3H), 1.14-1.27 (m, 6H), 1.41-1.47 (m, 1H), 1.51-1.57 (m, 3H), 1.78-1.86 (m, 2H), 1.91-2.00 (m, 2H), 2.01-2.11 (m, 1H), 2.22 (septet, *J* = 8.0 Hz, 1H), 5.43-5.44 (m, 1H), 5.78 (s, 1H) ppm.

2. NMR and HRMS Spectra of compounds 6 and 7

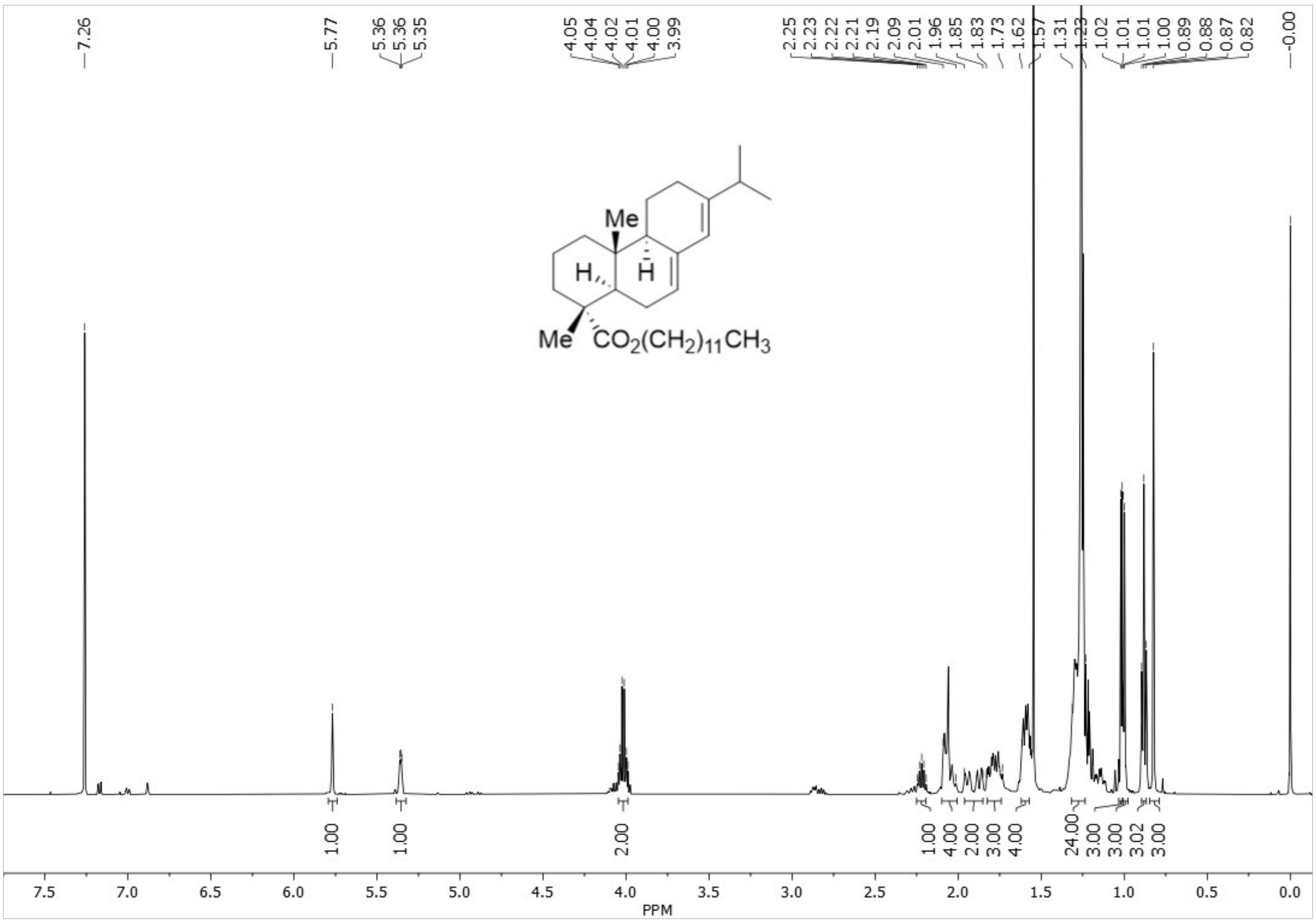

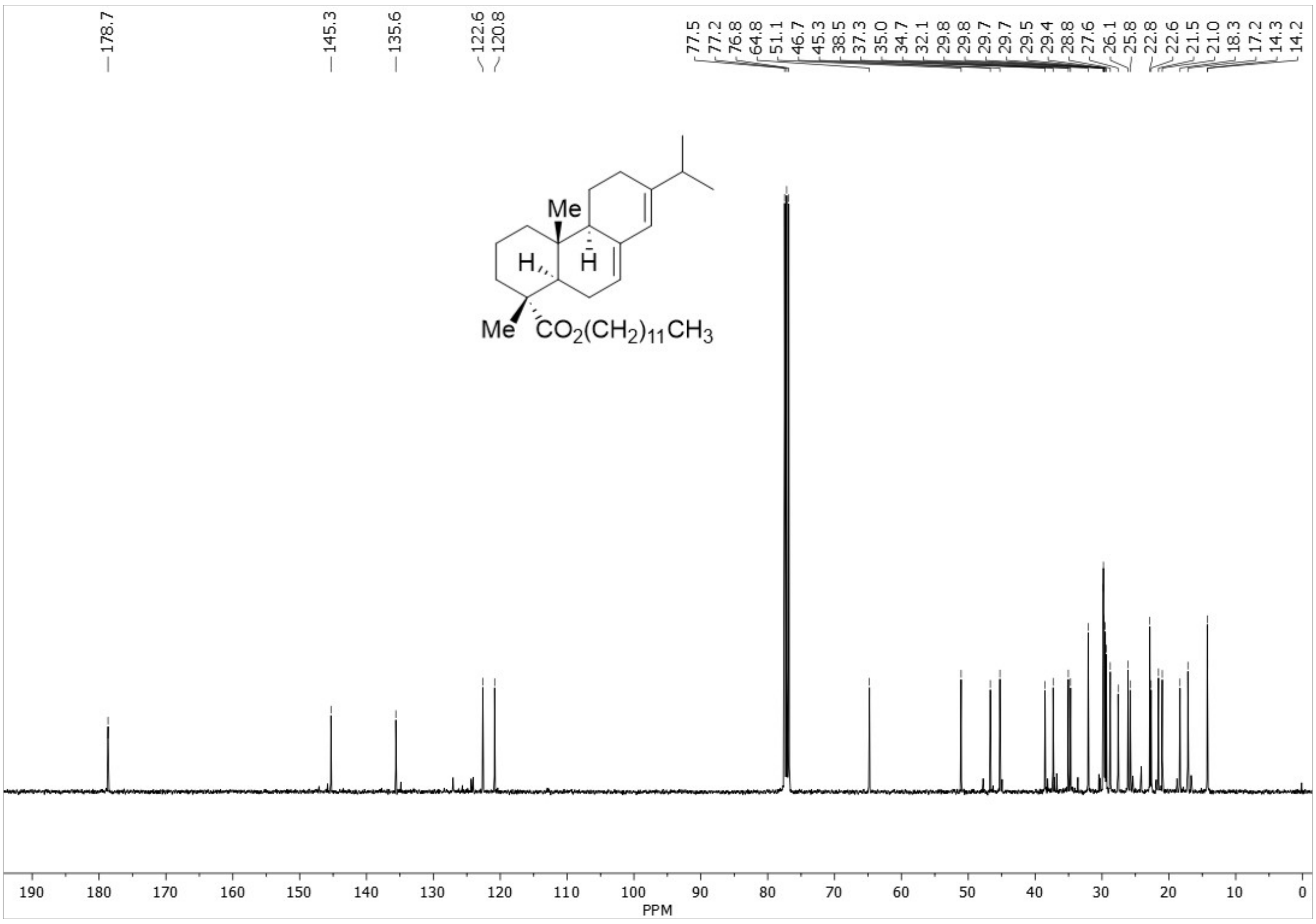

Figure S1. NMR spectra of abietate **6** ($CDCl_3$, ^1H at 400 MHz and ^{13}C at 100 MHz).

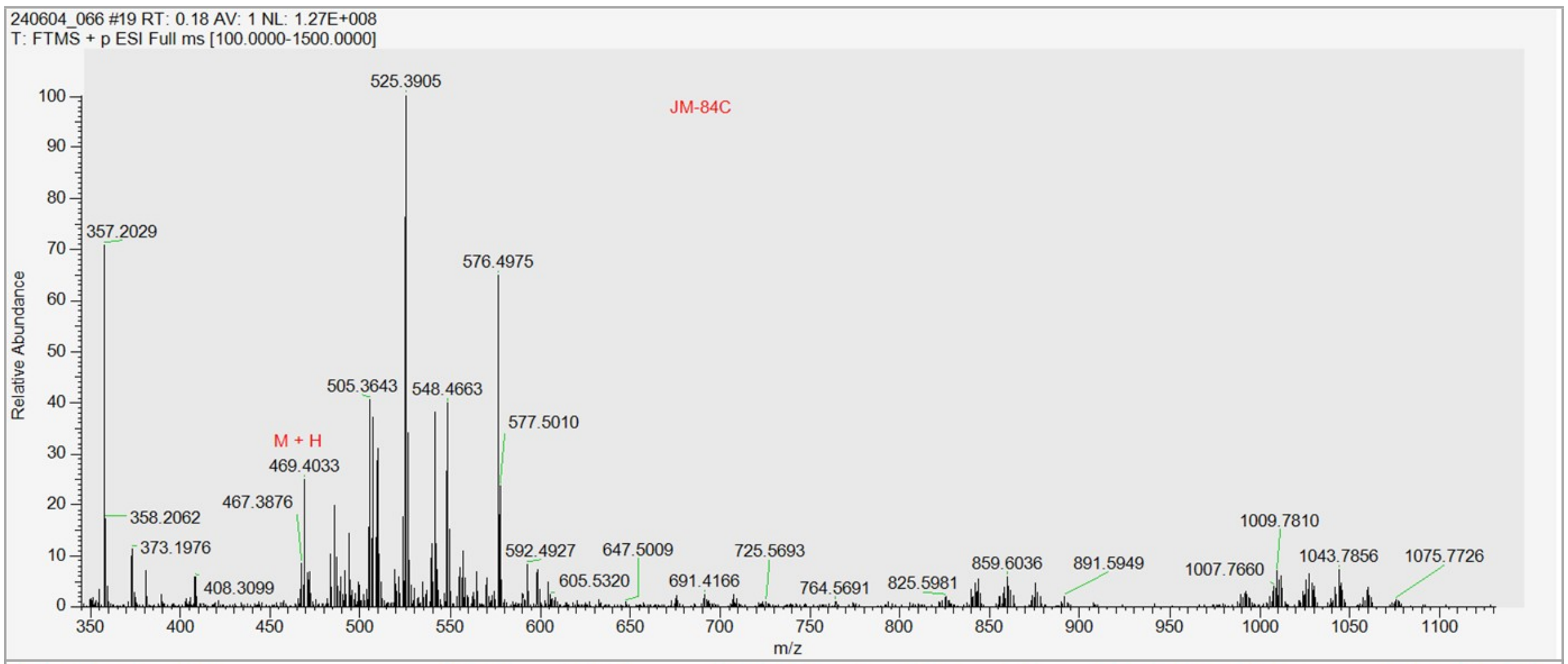

Display Formula	S Fit	RDB	Delta [ppm]	Theo. mass	Combined Score	# Matched Iso.
$C_{32}H_{53}O_2$	24,35	6,5	-1,46	469,40401	68,47	2

Figure S2. HRMS of abietate **6** (ESI).

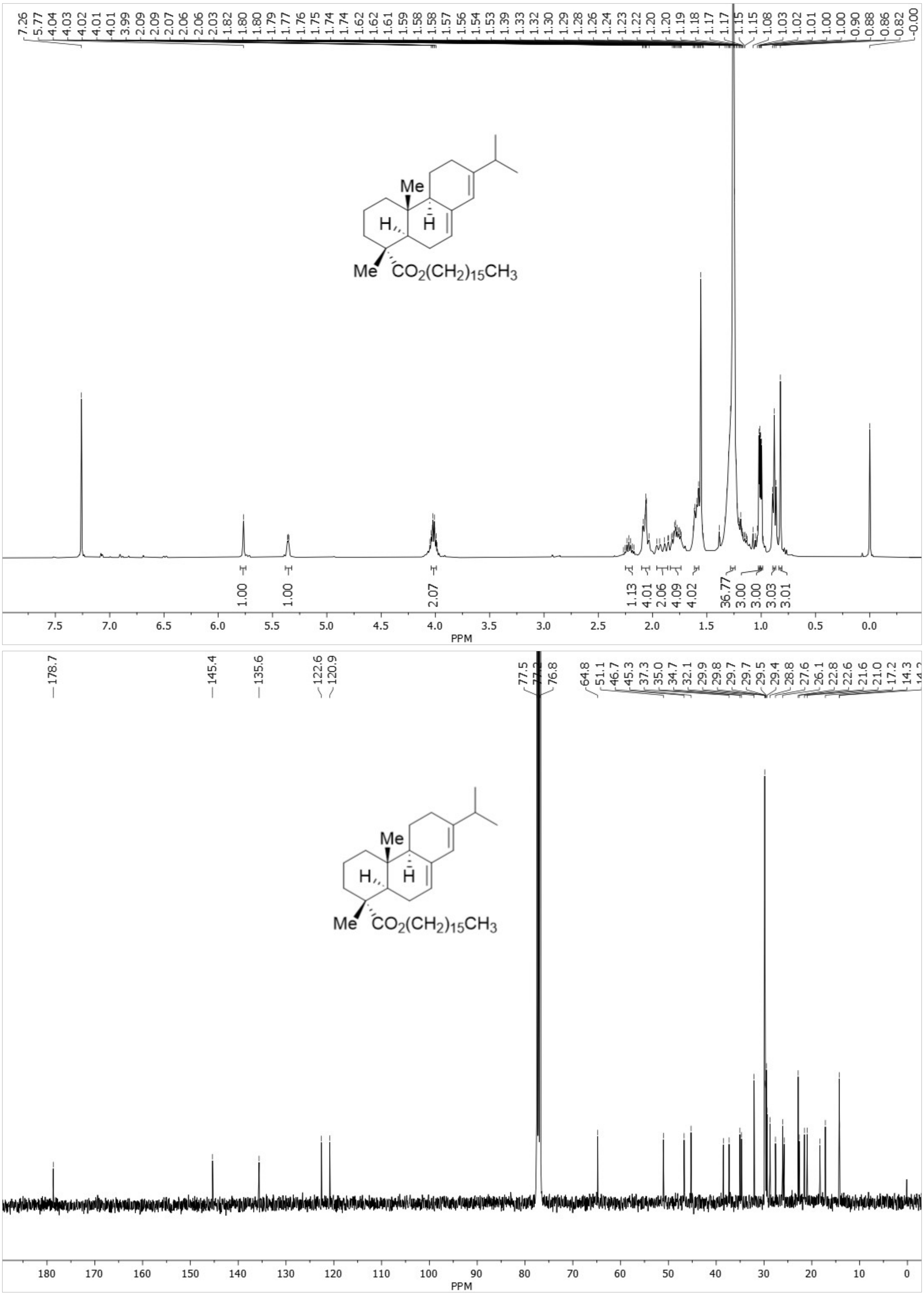

Figure S3. NMR spectra of abietate **7** ($CDCl_3$, ^{1}H at 400 MHz and ^{13}C at 100 MHz).

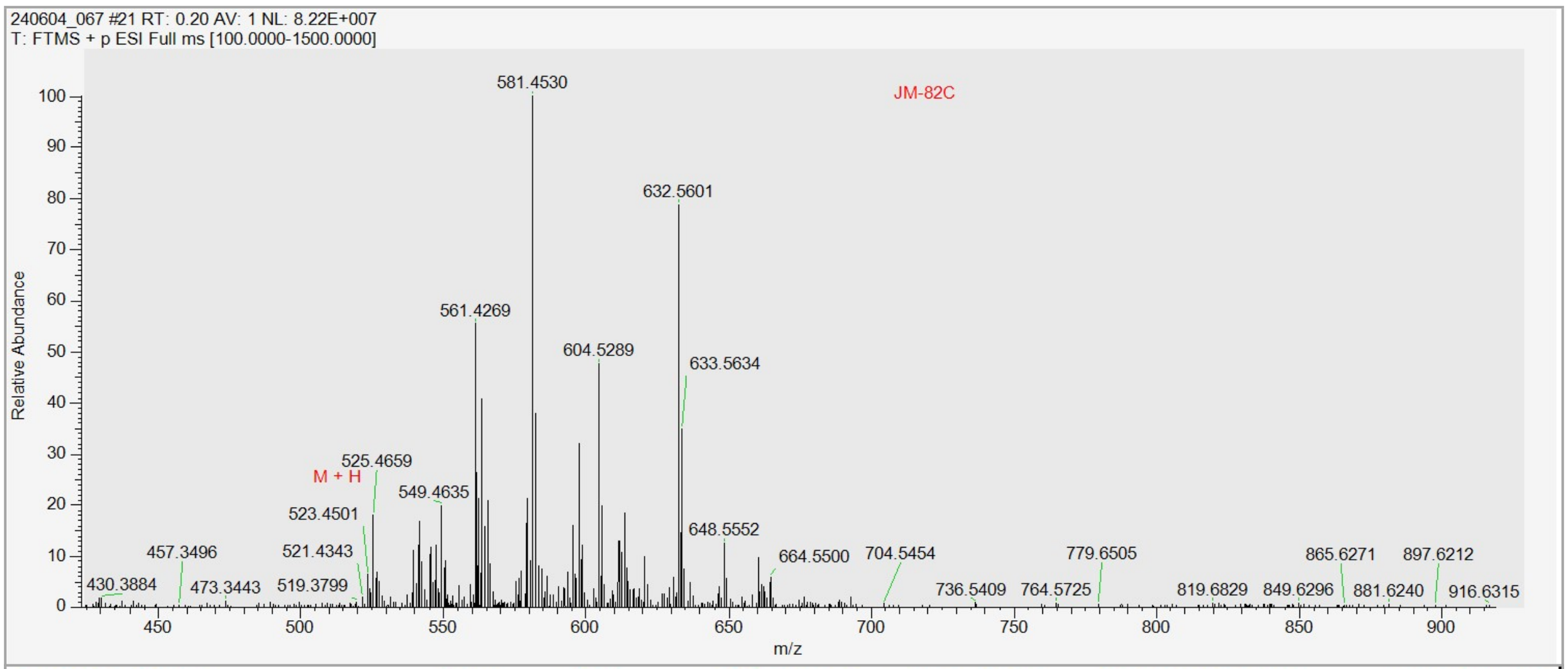

Display Formula	S Fit	RDB	Delta [ppm]	Theo. mass	Combined Score	# Matched Iso.
$C_{36}H_{61}O_2$	28,25	6,5	-1,27	525,46661	65,35	2

Figure S4. HRMS of abietate **7** (ESI).

3. HPLC-DAD solvent gradient

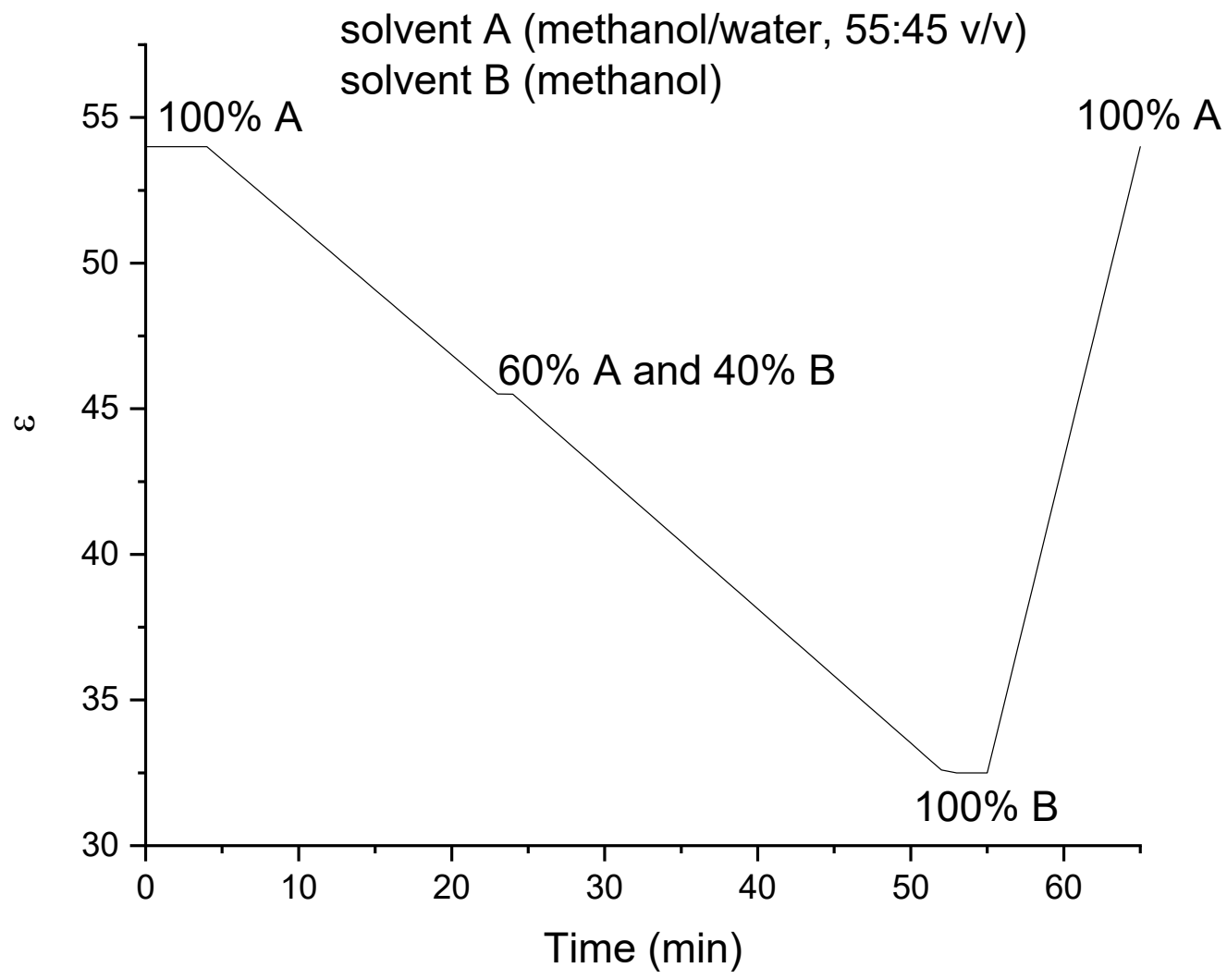

Figure S5. Binary gradient elution system used in HPLC-DAD. The mobile phase consisted of solvent A (methanol/water, 55:45 v/v) and solvent B (methanol).

3. Computational Studies

Table S1. ωB97XD/def2-SVP relative Gibbs energies in gas phase at 298.15 K ($G_{gas,rel}$), entropic terms (TS_{gas}) Boltzmann populations (x_{gas}), dipole moments (μ_{gas}) and polarizabilities (α_{gas}) for the sampled conformers of abietic acid (**1**), together with the corresponding solution-phase values $G_{sol,rel}$, TS_{sol}, x_{sol}, μ_{sol} and α_{sol} in *n*-octane (ε = 1.9406) and pentyl ethanoate (ε = 4.7297), calculated using the SMD continuum solvation model, and Gibbs free energies of solvation (ΔG_{solv}).[a]

Gas phase						
Conf.	$G_{gas,rel}$	TS_{gas}	x_{gas}	μ_{gas} / D	α_{gas} (a.u.)	
I	0.00	186.26	0.218	2.21	215.48	
II	0.13	186.15	0.207	2.16	215.96	
III	1.44	186.04	0.122	1.25	216.01	
IV	1.53	185.94	0.118	2.21	215.21	
V	1.97	186.42	0.099	1.55	215.99	
VI	2.19	186.93	0.090	1.56	215.28	
VII	3.08	185.20	0.063	1.25	215.55	
VIII	3.99	185.21	0.044	1.26	215.28	
IX	4.22	185.13	0.040	1.56	215.51	
		**(186.09)**[b]		**(1.83)**[b]	**(215.64)**[b]	
n-Octane						
Conf.	$G_{sol,rel}$	TS_{sol}	x_{sol}	μ_{sol} / D	α_{gas} (a.u.)	ΔG_{solv}
I	0.06	185.85	0.191	2.49	243.88	-42.34
II	0.00	186.27	0.195	2.45	244.28	-42.53
III	2.01	185.66	0.087	1.42	244.33	-41.83
IV	1.22	185.95	0.119	2.49	243.44	-42.71
V	1.65	187.02	0.100	1.74	244.13	-42.72
VI	1.74	187.50	0.097	1.76	243.41	-42.85
VII	2.65	185.32	0.067	1.42	243.90	-42.84
VIII	3.87	187.36	0.041	1.45	243.78	-42.53
IX	1.61	185.85	0.102	1.76	243.88	-45.01
		**(186.23)**[b]		**(2.05)**[b]	**(243.92)**[b]	**(-42.78)**[c]
Pentyl ethanoate						
Conf.	$G_{sol,rel}$	TS_{sol}	x_{sol}	μ_{sol} / D	α_{gas} (a.u.)	ΔG_{solv}
I	0.13	185.93	0.172	2.76	274.79	-48.99
II	0.71	185.85	0.136	2.72	275.08	-48.54
III	1.77	185.92	0.089	1.60	275.13	-48.79
IV	1.14	186.06	0.114	2.77	274.23	-49.51
V	0.00	188.39	0.181	1.96	274.80	-51.09
VI	1.30	188.14	0.107	1.97	274.19	-50.01
VII	2.50	185.58	0.066	1.60	274.85	-49.70
VIII	3.89	185.38	0.038	1.64	274.27	-49.23
IX	1.54	187.43	0.097	1.99	274.67	-51.80
		**(186.72)**[b]		**(2.23)**[b]	**(274.71)**[b]	**(-49.79)**[c]

[a] All energies are expressed in kJ mol^{-1}. [b] Entropic terms, dipole moments and polarizabilities of the molecule, calculated as Boltzmann-weighted averages of the corresponding values obtained for the individual conformers.[c] Gibbs energies of solvation estimated for the molecule obtained as the difference between the Boltzmann-weighted sum of the Gibbs energies of the conformers in solution and the Boltzmann-weighted sum of the Gibbs energies of the same conformers in the gas phase (for details see the Computational Methods section). Absolute values of the Gibbs energy (*G*) for conf. I: G_{gas} = -929.333627 Hartee; G_{sol} (*n*-octane) = -929.349754 Hartee; G_{sol} (pentyl ethanoate) = -929.352288 Hartee.

Table S2. ωB97XD/def2-SVP relative Gibbs energies in gas phase at 298.15 K ($G_{gas,rel}$), entropic terms (TS_{gas}) Boltzmann populations (x_{gas}), dipole moments (μ_{gas}) and polarizabilities (α_{gas}) for the sampled conformers of Abiet-7,13-dien-18-ol (**2**), together with the corresponding solution-phase values $G_{sol,rel}$, TS_{sol}, x_{sol}, μ_{sol} and α_{sol} in *n*-octane (ε = 1.9406) and pentyl ethanoate (ε = 4.7297), calculated using the SMD continuum solvation model, and Gibbs free energies of solvation (ΔG_{solv}).[a]

Gas phase						
Conf.	$G_{gas,rel}$	TS_{gas}	x_{gas}	μ_{gas} / D	α_{gas} (a.u.)	
I	0.00	182.60	0.261	1.48	216.12	
II	1.80	182.19	0.126	1.74	216.32	
III	1.97	181.72	0.118	1.51	215.62	
IV	2.27	182.19	0.104	1.77	215.81	
V	2.97	182.37	0.079	1.77	215.57	
VI	2.99	183.30	0.078	1.36	216.88	
VII	3.67	181.20	0.06	1.52	215.38	
VIII	4.70	182.69	0.039	1.32	216.36	
IX	4.72	183.11	0.039	1.30	216.11	
X	4.98	181.16	0.035	1.98	216.19	
XI	5.15	181.51	0.033	1.73	216.45	
XII	5.59	181.61	0.027	2.03	215.45	
		**(182.26)**[b]		**(1.59)**[b]	**(216.03)**[b]	
n-Octane						
Conf.	$G_{sol,rel}$	TS_{sol}	x_{sol}	μ_{sol} / D	α_{sol} (a.u.)	ΔG_{solv}
I	0.00	182.91	0.307	1.59	245.45	-40.36
II	2.37	182.05	0.118	1.98	245.66	-39.78
III	2.16	181.81	0.128	1.61	245.01	-40.16
IV	3.28	181.76	0.082	2.00	245.19	-39.35
V	3.45	182.31	0.076	2.00	244.82	-39.88
VI	4.06	183.12	0.060	1.39	246.18	-39.29
VII	3.44	181.55	0.077	1.64	244.66	-40.58
VIII	5.95	182.33	0.028	1.35	245.70	-39.11
IX	5.26	183.26	0.037	1.33	245.33	-39.81
X	5.69	181.08	0.031	2.19	245.29	-39.64
XI	5.71	181.48	0.031	1.97	245.70	-39.80
XII	6.13	181.61	0.026	2.24	244.42	-39.81
		**(182.30)**[b]		**(1.73)**[b]	**(245.31)**[b]	**(-40.14)**[c]
Pentyl ethanoate						
Conf.	$G_{sol,rel}$	TS_{sol}	x_{sol}	μ_{sol} / D	α_{sol} (a.u.)	ΔG_{solv}
I	0.00	182.58	0.258	1.67	277.47	-43.56
II	1.59	182.02	0.136	2.25	277.64	-43.77
III	0.79	182.47	0.187	1.70	277.17	-44.74
IV	2.89	181.52	0.080	2.28	277.34	-42.94
V	2.94	182.20	0.079	2.28	276.85	-43.59
VI	3.93	183.03	0.053	1.42	278.03	-42.62
VII	2.96	181.46	0.078	1.72	276.73	-44.27
VIII	6.44	181.87	0.019	1.39	277.70	-41.83
IX	5.50	183.05	0.028	1.35	277.23	-42.79
X	5.31	181.09	0.030	2.42	277.16	-43.22
XI	5.23	181.47	0.031	2.24	277.62	-43.48
XII	6.27	181.08	0.021	2.49	276.40	-42.88
		**(182.19)**[b]		**(1.89)**[b]	**(277.32)**[b]	**(-43.81)**[c]

[a] All energies are expressed in kJ mol^{-1}. [b] Entropic terms, dipole moments and polarizabilities of the molecule, calculated as Boltzmann-weighted averages of the corresponding values obtained for the individual conformers.[c] Gibbs energies of solvation estimated for the molecule obtained as the difference between the Boltzmann-weighted sum of the Gibbs energies of the conformers in solution and the Boltzmann-weighted sum of the Gibbs energies of the same conformers in the gas phase (for details see the Computational Methods section). Absolute values of the Gibbs energy (G) for conf. I: G_{gas} = -855.347720 Hartee; G_{sol} (*n*-octane) = -855.363091 Hartee; G_{sol} (pentyl ethanoate) = -855.364312 Hartee.

Table S3. ωB97XD/def2-SVP relative Gibbs energies in gas phase at 298.15 K ($G_{gas,rel}$), entropic terms (TS_{gas}) Boltzmann populations (x_{gas}), dipole moments (μ_{gas}) and polarizabilities (α_{gas}) for the sampled conformers of Abiet-7,13-dien-18-yl *p*-toluenesulfonate (**3**), together with the corresponding solution-phase values $G_{sol,rel}$, TS_{sol}, x_{sol}, μ_{sol} and α_{sol} in *n*-octane (ε = 1.9406) and pentyl ethanoate (ε = 4.7297), calculated using the SMD continuum solvation model, and Gibbs free energies of solvation (ΔG_{solv}).[a]

Gas phase						
Conf.	$G_{gas,rel}$	TS_{gas}	x_{gas}	μ_{gas} / D	α_{gas} (a.u.)	
I	0.00	237.96	0.624	5.38	299.39	
II	1.95	234.55	0.284	5.49	298.19	
III	4.73	233.72	0.093	5.44	298.68	
		**(236.60)**[b]		**(5.42)**[b]	**(298.98)**[b]	
n-Octane						
Conf.	$G_{sol,rel}$	TS_{sol}	x_{sol}	μ_{sol} / D	α_{sol} (a.u.)	ΔG_{solv}
I	0.00	238.98	0.805	6.05	342.10	-55.68
II	4.17	234.72	0.150	6.16	340.96	-53.47
III	7.16	232.78	0.045	6.12	341.65	-53.24
		**(238.06)**[b]		**(6.07)**[b]	**(341.91)**[b]	**(-55.73)**[c]
Pentyl ethanoate						
Conf.	$G_{sol,rel}$	TS_{sol}	x_{sol}	μ_{sol} / D	α_{sol} (a.u.)	ΔG_{solv}
I	0.36	237.38	0.424	6.63	388.57	-63.73
II	0.00	237.00	0.491	6.72	387.52	-66.04
III	4.34	234.14	0.085	6.71	388.14	-64.47
		**(236.91)**[b]		**(6.68)**[b]	**(388.02)**[b]	**(-64.56)**[c]

[a] All energies are expressed in kJ mol^{-1}. [b] Entropic terms, dipole moments and polarizabilities of the molecule, calculated as Boltzmann-weighted averages of the corresponding values obtained for the individual conformers.[c] Gibbs energies of solvation estimated for the molecule obtained as the difference between the Boltzmann-weighted sum of the Gibbs energies of the conformers in solution and the Boltzmann-weighted sum of the Gibbs energies of the same conformers in the gas phase (for details see the Computational Methods section). Absolute values of the Gibbs energy (G) for conf. I: G_{gas} = -1673.621003 Hartee; G_{sol} (*n*-octane) = -1673.642211 Hartee; G_{sol} (pentyl ethanoate) = -1673.645275 Hartee.

Table S4. ωB97XD/def2-SVP relative Gibbs energies in gas phase at 298.15 K ($G_{gas,rel}$), entropic terms (TS_{gas}) Boltzmann populations (x_{gas}), dipole moments (μ_{gas}) and polarizabilities (α_{gas}) for the sampled conformers of 18-Iodo abiet-7,13-diene (**4**), together with the corresponding solution-phase values $G_{sol,rel}$, TS_{sol}, x_{sol}, μ_{sol} and α_{sol} in *n*-octane (ε = 1.9406) and pentyl ethanoate (ε = 4.7297), calculated using the SMD continuum solvation model, and Gibbs free energies of solvation (ΔG_{solv}).[a]

Gas phase						
Conf.	$G_{gas,rel}$	TS_{gas}	x_{gas}	μ_{gas} / D	α_{gas} (a.u.)	
I	0.00	189.62	0.496	1.90	238.11	
II	2.75	188.24	0.164	1.90	237.50	
III	2.87	190.57	0.156	2.50	241.75	
IV	3.74	188.39	0.110	1.90	237.41	
V	6.08	188.76	0.043	2.50	241.23	
VI	6.86	188.98	0.031	2.60	241.04	
		**(189.35)**[b]		**(2.04)**[b]	**(238.73)**[b]	
n-Octane						
Conf.	$G_{sol,rel}$	TS_{sol}	x_{sol}	μ_{sol} / D	α_{sol} (a.u.)	ΔG_{solv}
I	0.00	189.48	0.505	2.13	272.28	-36.00
II	2.40	188.35	0.192	2.15	271.72	-36.34
III	3.77	189.70	0.110	2.71	275.36	-35.10
IV	3.69	188.39	0.114	2.16	271.49	-36.04
V	5.86	188.54	0.048	2.74	274.84	-36.22
VI	6.88	188.76	0.032	2.77	274.51	-35.98
		**(189.10)**[b]		**(2.250)**[b]	**(272.62)**[b]	**(-35.99)**[c]
Pentyl ethanoate						
Conf.	$G_{sol,rel}$	TS_{sol}	x_{sol}	μ_{sol} / D	α_{sol} (a.u.)	ΔG_{solv}
I	0.00	189.51	0.510	2.41	309.80	-38.54
II	2.37	188.50	0.196	2.43	309.41	-38.92
III	4.08	189.61	0.098	2.91	311.97	-37.33
IV	3.48	188.62	0.125	2.45	309.07	-38.80
V	6.41	188.32	0.038	2.94	311.59	-38.21
VI	6.76	188.94	0.033	2.98	311.14	-38.65
				**(2.51)**[b]	**(309.96)**[b]	**(-38.55)**[c]

[a] All energies are expressed in kJ mol^{-1}. [b] Entropic terms, dipole moments and polarizabilities of the molecule, calculated as Boltzmann-weighted averages of the corresponding values obtained for the individual conformers.[c] Gibbs energies of solvation estimated for the molecule obtained as the difference between the Boltzmann-weighted sum of the Gibbs energies of the conformers in solution and the Boltzmann-weighted sum of the Gibbs energies of the same conformers in the gas phase (for details see the Computational Methods section). Absolute values of the Gibbs energy (G) for conf. I: G_{gas} = -1077.405527 Hartee; G_{sol} (*n*-octane) = -1077.419234 Hartee; G_{sol} (pentyl ethanoate) = -1077.420211 Hartee.

Table S5. ωB97XD/def2-SVP relative Gibbs energies in gas phase at 298.15 K ($G_{gas,rel}$), entropic terms (TS_{gas}) Boltzmann populations (x_{gas}), dipole moments (μ_{gas}) and polarizabilities (α_{gas}) for the sampled conformers of Abieta-7,13-diene (**5**), together with the corresponding solution-phase values $G_{sol,rel}$, TS_{sol}, x_{sol}, μ_{sol} and α_{sol} in *n*-octane (ε = 1.9406) and pentyl ethanoate (ε = 4.7297), calculated using the SMD continuum solvation model, and Gibbs free energies of solvation (ΔG_{solv}).[a]

Gas phase						
Conf.	$G_{gas,rel}$	TS_{gas}	x_{gas}	μ_{gas} / D	α_{gas} (a.u.)	
I	0.00	174.86	0.459	0.44	213.94	
II	0.47	174.21	0.380	0.49	213.22	
III	2.59	174.19	0.161	0.49	213.22	
		**(174.51)**[b]		**(0.47)**[b]	**(213.55)**[b]	
n-Octane						
Conf.	$G_{sol,rel}$	TS_{sol}	x_{sol}	μ_{sol} / D	α_{sol} (a.u.)	ΔG_{solv}
I	0.00	175.81	0.805	0.55	243.65	-37.50
II	10.03	170.84	0.014	0.60	242.94	-27.93
III	3.70	174.37	0.181	0.61	242.85	-36.38
		**(175.48)**[b]		**(0.564)**[b]	**(243.50)**[b]	**(-37.28)**[c]
Pentyl ethanoate						
Conf.	$G_{sol,rel}$	TS_{sol}	x_{sol}	μ_{sol} / D	α_{sol} (a.u.)	ΔG_{solv}
I	0.00	175.26	0.759	0.70	276.12	-36.15
II	9.66	170.87	0.015	0.74	275.37	-26.96
III	3.01	174.31	0.225	0.75	275.35	-35.73
		**(174.80)**[b]		**(0.708)**[b]	**(275.66)**[b]	**(-35.92)**[c]

[a] All energies are expressed in kJ mol^{-1}. [b] Entropic terms, dipole moments and polarizabilities of the molecule, calculated as Boltzmann-weighted averages of the corresponding values obtained for the individual conformers.[c] Gibbs energies of solvation estimated for the molecule obtained as the difference between the Boltzmann-weighted sum of the Gibbs energies of the conformers in solution and the Boltzmann-weighted sum of the Gibbs energies of the same conformers in the gas phase (for details see the Computational Methods section). Absolute values of the Gibbs energy (G) for conf. I: G_{gas} = -780.216841 Hartee; G_{sol} (*n*-octane) = -780.231041 Hartee; G_{sol} (pentyl ethanoate) = -780.230521 Hartee.

Table S6. ωB97XD/def2-SVP relative Gibbs energies in gas phase at 298.15 K ($G_{gas,rel}$), entropic terms (TS_{gas}) Boltzmann populations (x_{gas}), dipole moments (μ_{gas}) and polarizabilities (α_{gas}) for the sampled conformers of Dodecyl abietate (**6**), together with the corresponding solution-phase values $G_{sol,rel}$, TS_{sol}, x_{sol}, μ_{sol} and α_{sol} in *n*-octane (ε = 1.9406) and pentyl ethanoate (ε = 4.7297), calculated using the SMD continuum solvation model, and Gibbs free energies of solvation (ΔG_{solv}).[a]

Gas phase						
Conf.	$G_{gas,rel}$	TS_{gas}	x_{gas}	μ_{gas} / D	α_{gas} (a.u.)	
I	0.00	280.23	0.118	2.27	345.82	
II	0.25	278.52	0.106	2.40	347.03	
III	0.73	276.65	0.088	2.41	346.97	
IV	1.22	278.27	0.072	2.10	346.96	
V	1.27	279.09	0.071	2.39	346.81	
VI	1.51	277.83	0.064	2.44	342.76	
VII	2.24	274.72	0.048	2.38	346.45	
VIII	2.53	274.93	0.042	2.25	343.19	
IX	2.55	275.73	0.042	2.27	345.41	
X	2.61	275.69	0.041	2.27	345.43	
XI	2.78	275.56	0.038	2.27	345.43	
XII	2.92	273.40	0.036	2.42	343.05	
XIII	3.10	279.96	0.034	2.45	346.84	
XIV	3.13	280.88	0.033	2.18	347.60	
XV	3.27	276.35	0.031	2.17	345.48	
XVI	3.34	276.11	0.031	2.43	343.93	
XVII	3.34	281.07	0.031	2.19	347.30	
XVIII	3.42	277.38	0.030	2.37	346.74	
XIX	3.61	274.49	0.027	2.45	341.99	
XX	4.72	280.30	0.018	2.04	344.73	
		**(277.62)**[b]		**(2.32)**[b]	**(345.75)**[b]	

n-Octane						
Conf.	$G_{gas,rel}$	TS_{gas}	x_{gas}	μ_{gas} / D	α_{gas} (a.u.)	ΔG_{solv}
I	1.13	280.41	0.112	2.53	392.05	-59.60
II	3.51	275.99	0.043	2.70	391.06	-57.48
III	1.18	277.84	0.110	2.70	391.15	-60.29
IV	1.96	279.09	0.080	2.38	391.65	-60.00
V	4.05	277.38	0.035	2.70	390.90	-57.95
VI	5.45	276.63	0.020	2.74	390.19	-56.79
VII	0.25	278.70	0.160	2.66	391.02	-62.72
VIII	–	–	–	–	–	–
IX	2.31	278.15	0.070	2.57	391.93	-60.98
X	–	–	–	–	–	–
XI	–	–	–	–	–	–
XII	5.94	274.68	0.016	2.71	390.52	-57.72
XIII	6.10	278.10	0.015	2.75	391.12	-57.73
XIV	2.43	282.03	0.067	2.45	392.28	-61.44
XV	0.00	280.30	0.177	2.45	391.15	-64.00
XVI	7.02	275.62	0.010	2.74	390.94	-57.05
XVII	3.37	281.60	0.045	2.47	392.16	-60.70
XVIII	5.82	276.53	0.017	2.66	390.85	-58.33
XIX	7.97	274.29	0.007	2.75	389.76	-56.38
XX	6.00	280.72	0.016	2.41	390.92	-59.45
		**(279.08)**[b]		**(2.57)**[b]	**(391.38)**[b]	**(-60.71)**[c]

Pentyl ethanoate						
Conf.	$G_{gas,rel}$	TS_{gas}	x_{gas}	μ_{gas} / D	α_{gas} (a.u.)	ΔG_{solv}
I	1.36	279.29	0.078	2.81	441.63	-61.07
II	2.36	275.61	0.052	2.98	438.91	-60.33
III	2.71	274.82	0.045	2.98	439.20	-60.45
IV	1.27	278.76	0.081	2.67	440.25	-62.39
V	3.98	275.84	0.027	2.98	438.79	-59.72
VI	5.18	275.61	0.017	3.05	441.12	-58.76
VII	0.53	276.67	0.109	2.94	439.12	-64.14
VIII	1.44	276.16	0.076	2.83	440.90	-63.52
IX	2.94	276.26	0.041	2.83	441.74	-62.05
X	3.00	276.22	0.040	2.83	441.74	-62.05
XI	2.95	276.25	0.041	2.83	441.75	-62.26
XII	4.61	274.02	0.021	3.00	441.14	-60.75
XIII	4.69	277.98	0.020	3.02	439.15	-60.84
XIV	1.86	281.33	0.064	2.73	440.80	-63.70
XV	0.00	279.33	0.135	2.74	440.24	-65.71
XVI	1.80	278.53	0.065	3.04	441.10	-63.98
XVII	2.72	280.91	0.045	2.75	440.81	-63.06
XVIII	4.51	276.20	0.022	2.93	438.72	-61.35
XIX	7.02	273.51	0.008	3.06	440.99	-59.02
XX	6.04	279.64	0.012	2.69	440.49	-61.11
		**(277.71)**[b]		**(2.85)**[b]	**(440.41)**[b]	**(-62.29)**[c]

[a] All energies are expressed in kJ mol^{-1}. In *n*-octane conformation VIII did not converge while conformations X and XI converged to IX. [b] Entropic terms, dipole moments and polarizabilities of the molecule, calculated as Boltzmann-weighted averages of the corresponding values obtained for the individual conformers.[c] Gibbs energies of solvation estimated for the molecule obtained as the difference between the Boltzmann-weighted sum of the Gibbs energies of the conformers in solution and the Boltzmann-weighted sum of the Gibbs energies of the same conformers in the gas phase (for details see the Computational Methods section). Absolute values of the Gibbs energy (G) for conf. I: G_{gas} = -1400.317827 Hartee; G_{sol} (*n*-octane) = -1400.340946 Hartee; G_{sol} (pentyl ethanoate) = -1400.341549 Hartee.

Table S7. ωB97XD/def2-SVP relative Gibbs energies in gas phase at 298.15 K ($G_{gas,rel}$), entropic terms (TS_{gas}) Boltzmann populations (x_{gas}), dipole moments (μ_{gas}) and polarizabilities (α_{gas}) for the sampled conformers of Hexadodecyl abietate (**7**), together with the corresponding solution-phase values $G_{sol,rel}$, TS_{sol}, x_{sol}, μ_{sol} and α_{sol} in *n*-octane (ε = 1.9406) and pentyl ethanoate (ε = 4.7297), calculated using the SMD continuum solvation model, and Gibbs free energies of solvation (ΔG_{solv}).[a]

Gas phase						
Conf.	$G_{gas,rel}$	TS_{gas}	x_{gas}	μ_{gas} / D	α_{gas} (a.u.)	
I (D)	0.00	340.68	0.142	1.43	400.09	
II (F)	1.31	312.03	0.084	1.79	394.01	
III (D/F)	1.33	339.04	0.083	1.48	399.72	
IV (D)	1.44	337.42	0.080	2.06	397.44	
V (D)	2.08	338.20	0.062	1.53	399.26	
VI (F)	2.32	313.02	0.056	1.21	395.08	
VII (D)	2.84	335.16	0.045	1.12	398.65	
VIII (D)	2.87	339.94	0.045	1.46	399.42	
IX (D)	2.95	333.88	0.043	1.12	399.18	
X (D)	2.97	336.36	0.043	1.45	400.80	
XI (D)	3.01	336.73	0.042	2.45	396.94	
XII (D)	3.08	336.26	0.041	1.45	400.80	
XIII (F)	3.47	336.34	0.035	1.50	398.01	
XIV (D/F)	3.62	336.76	0.033	1.50	398.69	
XV (f)	3.81	337.91	0.031	1.40	398.71	
XVI (D)	3.94	337.95	0.029	1.39	399.75	
XVII (D)	4.03	334.13	0.028	1.12	398.59	
XVIII (F)	4.17	334.90	0.027	1.19	396.61	
XIX (D/F)	4.23	334.98	0.026	2.07	397.35	
XX (D)	4.23	333.53	0.026	1.11	398.97	
		**(333.96)**[b]		**(1.52)**[b]	**(398.39)**[b]	

n-Octane						
Conf.	$G_{gas,rel}$	TS_{gas}	x_{gas}	μ_{gas} / D	α_{gas} (a.u.)	ΔG_{solv}
I (D)	12.99	328.04	0.003	1.54	444.10	-69.40
II (F)	15.01	310.63	0.001	2.01	442.32	-68.69
III (D/F)	0.00	343.83	0.618	1.58	444.10	-83.72
IV (D)	5.48	337.74	0.068	2.37	442.31	-78.35
V (D)	14.02	327.22	0.002	1.61	444.04	-70.45
VI (F)	7.42	316.47	0.031	1.31	444.43	-77.29
VII (D)	15.42	324.94	0.001	1.23	443.75	-69.81
VIII (D)	8.72	337.33	0.018	1.56	443.86	-76.54
IX (D)	7.15	333.79	0.034	1.24	444.09	-78.18
X (D)	7.25	335.13	0.033	1.54	444.44	-78.11
XI (D)	7.07	335.93	0.036	2.73	442.16	-78.32
XII (D)	7.24	335.13	0.033	1.54	444.44	-78.22
XIII (F)	10.22	334.15	0.010	1.59	443.25	-75.64
XIV (D/F)	9.23	334.80	0.015	1.59	443.57	-76.77
XV (f)	10.96	334.89	0.007	1.52	443.75	-75.24
XVI (D)	7.75	337.96	0.027	1.51	443.90	-78.58
XVII (D)	8.59	334.70	0.019	1.23	443.79	-77.84
XVIII (F)	8.70	335.33	0.018	1.28	443.08	-77.85
XIX (D/F)	10.72	333.38	0.008	2.40	442.38	-75.90
XX (D)	9.33	333.15	0.014	1.23	443.84	-77.29
		**(340.06)**[b]		**(1.64)**[b]	**(443.87)**[b]	**(-81.73)**[c]

Pentyl ethanoate						
Conf.	$G_{gas,rel}$	TS_{gas}	x_{gas}	μ_{gas} / D	α_{gas} (a.u.)	ΔG_{solv}
I (D)	0.00	338.23	0.189	1.63	494.13	-78.44
II (F)	7.27	311.51	0.010	2.24	495.21	-72.48
III (D/F)	10.96	324.73	0.002	1.68	494.58	-68.82
IV (D)	8.78	326.35	0.005	2.72	493.14	-71.10
V (D)	7.94	327.36	0.008	1.69	494.49	-72.58
VI (F)	2.68	314.70	0.064	1.43	497.37	-78.09
VII (D)	9.88	324.87	0.004	1.37	494.33	-71.40
VIII (D)	2.99	337.05	0.057	1.66	494.23	-78.32
IX (D)	3.96	332.06	0.038	1.39	494.67	-77.42
X (D)	1.32	335.12	0.111	1.64	494.35	-80.09
XI (D)	1.58	335.50	0.100	3.00	493.26	-79.87
XII (D)	1.32	335.12	0.111	1.64	494.35	-80.19
XIII (F)	3.83	334.37	0.040	1.68	494.06	-78.08
XIV (D/F)	4.55	333.68	0.030	1.69	494.21	-77.50
XV (F)	5.11	334.96	0.024	1.63	494.40	-77.14
XVI (D)	2.68	336.85	0.064	1.62	494.18	-79.70
XVII (D)	2.76	334.84	0.062	1.37	494.35	-79.71
XVIII (F)	4.62	333.85	0.029	1.42	494.43	-77.99
XIX (D/F)	5.97	332.79	0.017	2.73	493.36	-76.70
XX (D)	4.22	332.88	0.034	1.38	494.40	-78.45
		**(333.88)**[b]		**(1.75)**[b]	**(494.36)**[b]	**(-78.39)**[c]

[a] All energies are expressed in kJ mol^{-1}. Conformers are characterized as distended (D), folded (F), or partially folded / partially distended (F/D) character. [b] Entropic terms, dipole moments and polarizabilities of the molecule, calculated as Boltzmann-weighted averages of the corresponding values obtained for the individual conformers.[c] Gibbs energies of solvation estimated for the molecule obtained as the difference between the Boltzmann-weighted sum of the Gibbs energies of the conformers in solution and the Boltzmann-weighted sum of the Gibbs energies of the same conformers in the gas phase (for details see the Computational Methods section). Absolute values of the Gibbs energy (G) for conf. I: G_{gas} = -1557.306794 Hartee; G_{sol} (*n*-octane) = -1557.337922 Hartee; G_{sol} (pentyl ethanoate) = -1557.336650 Hartee.

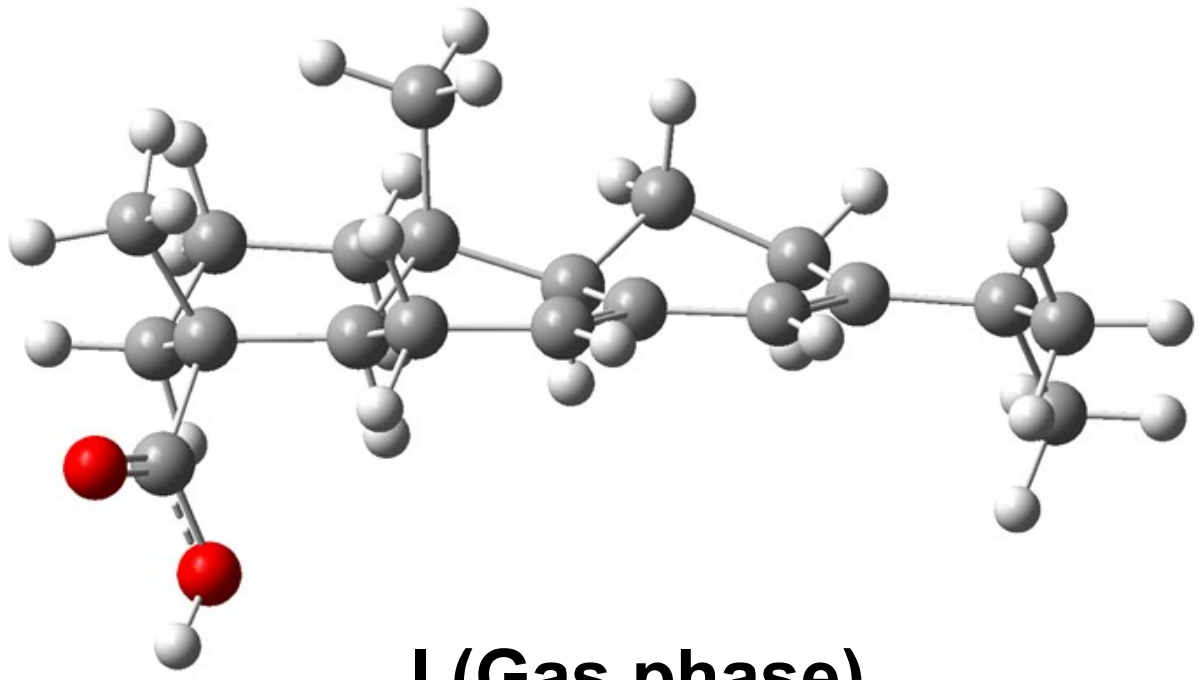

I (Gas phase)

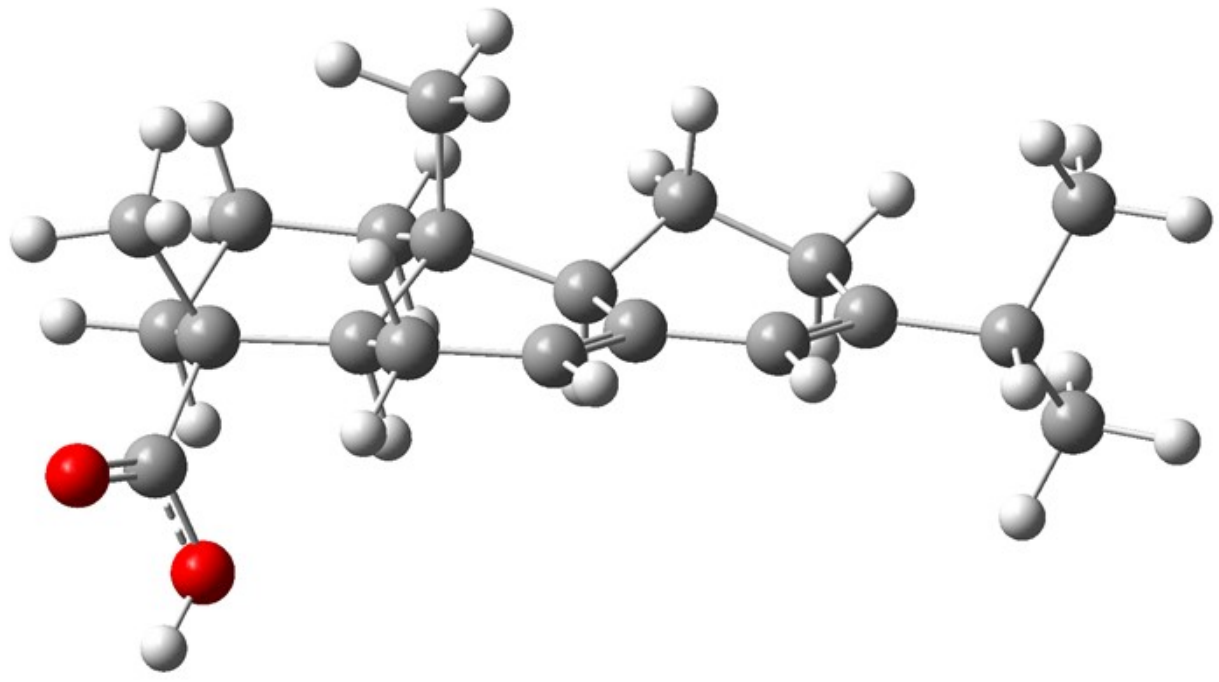

II (*n*-Octane)

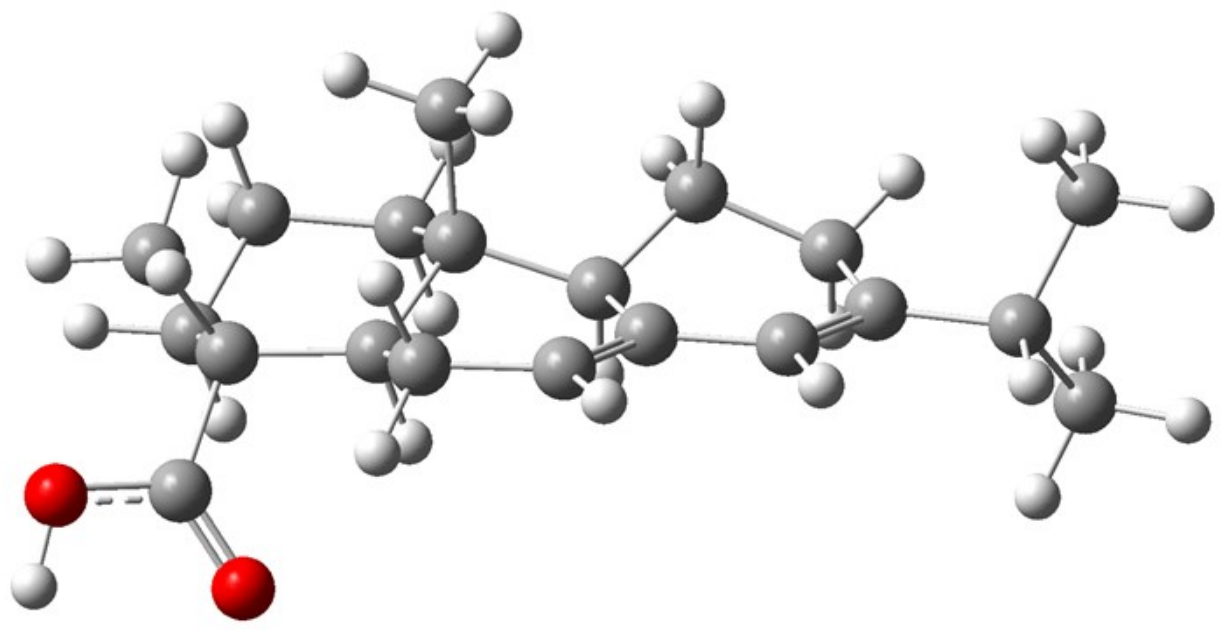

V (Pentyl ethanoate)

Figure S6. ωB97XD/def2-SVP optimized geometries of the lowest-energy conformers of abietic acid (**1**) in the gas phase, *n*-octane, and pentyl ethanoate.

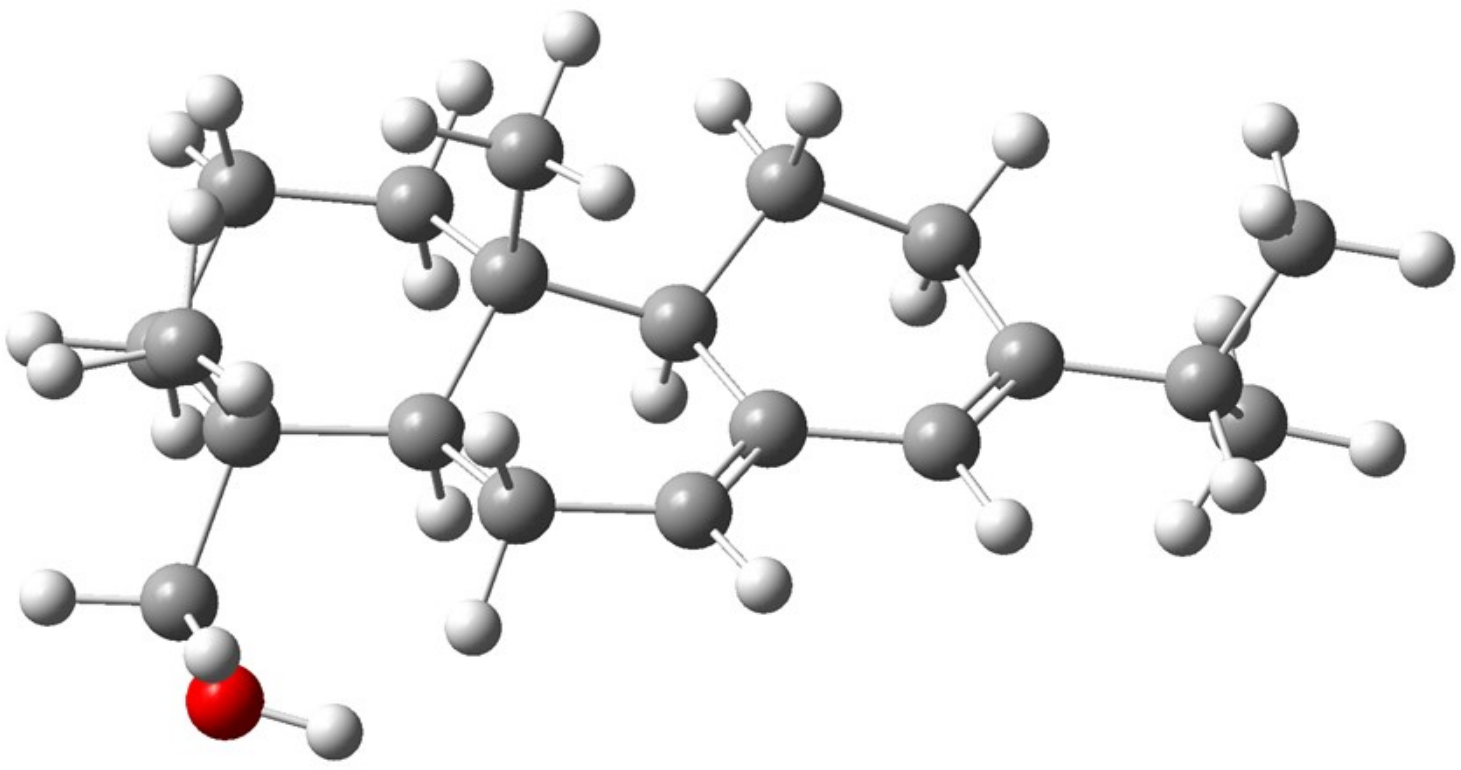

I (Gas phase)

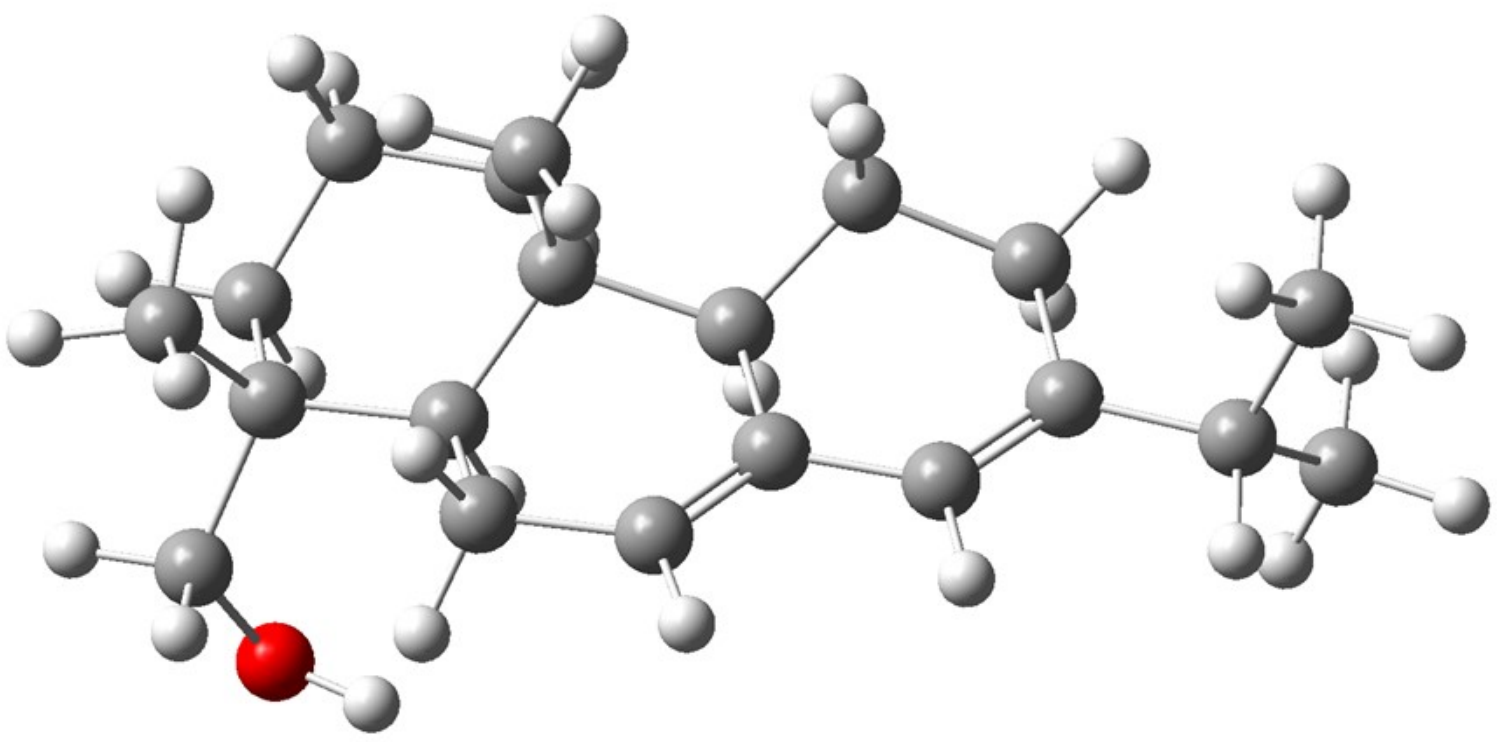

I (*n*-Octane)

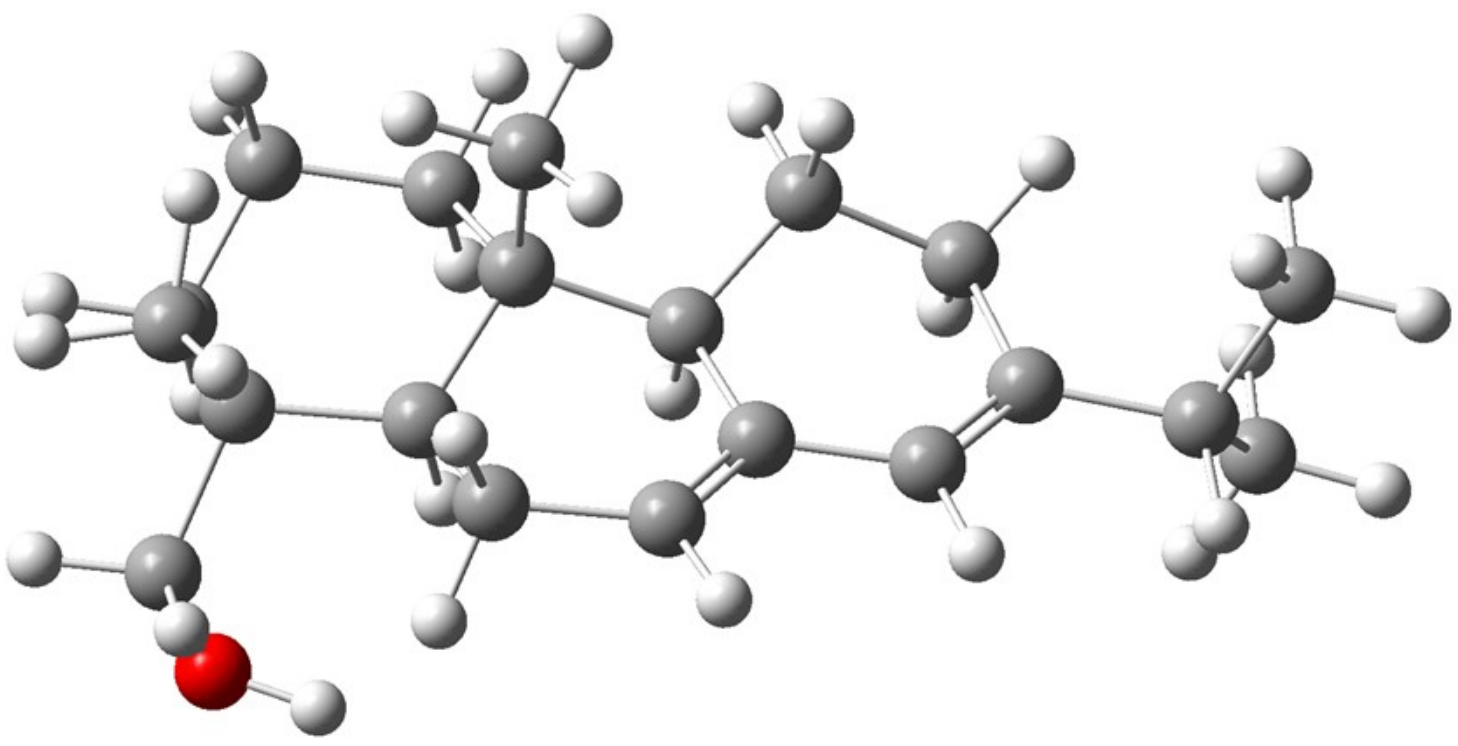

I (Pentyl ethanoate)

Figure S7. ωB97XD/def2-SVP optimized geometries of the lowest-energy conformers of Abiet-7,13-dien-18-ol (**2**) in the gas phase, *n*-octane, and pentyl ethanoate.

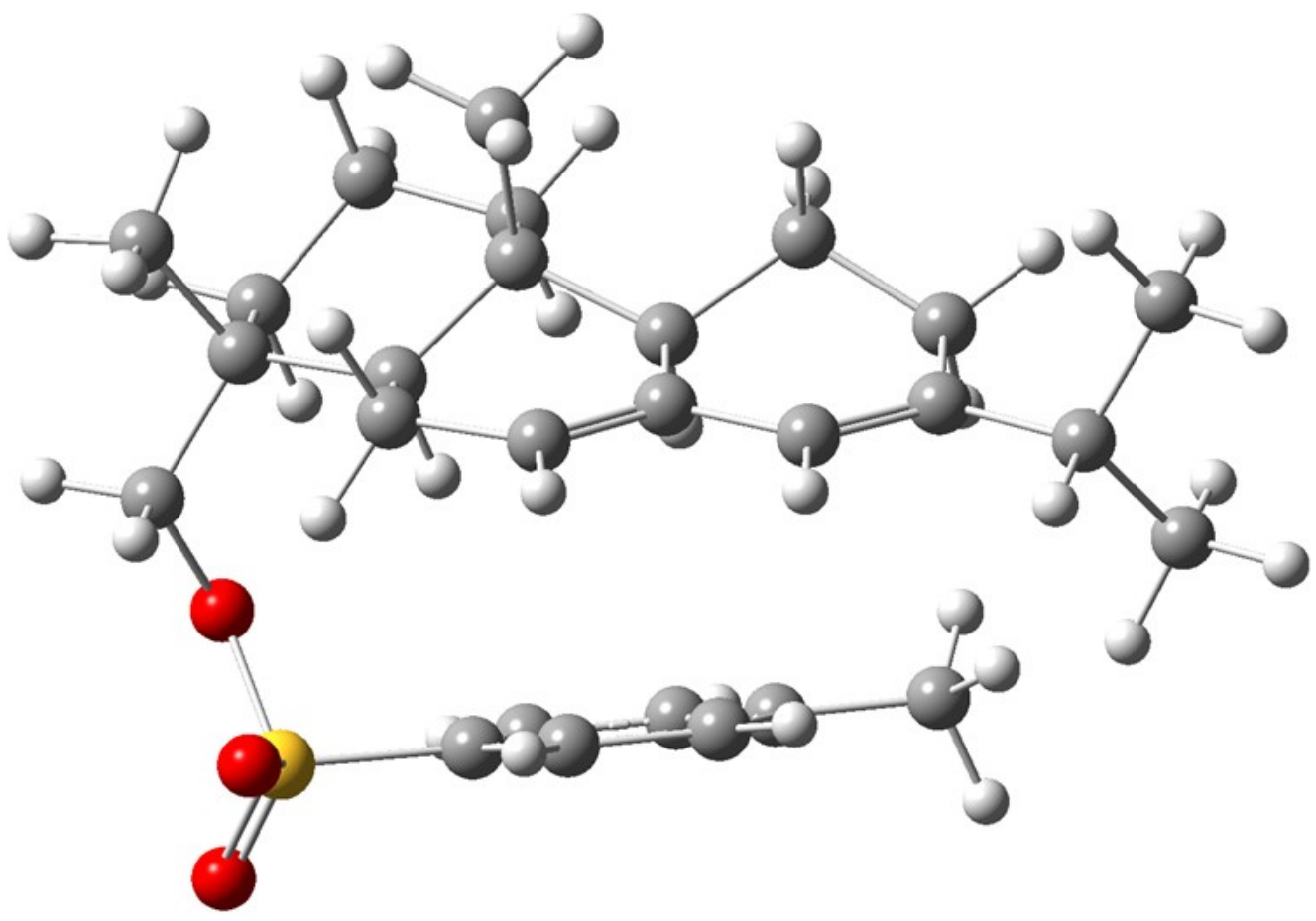

I (Gas phase)

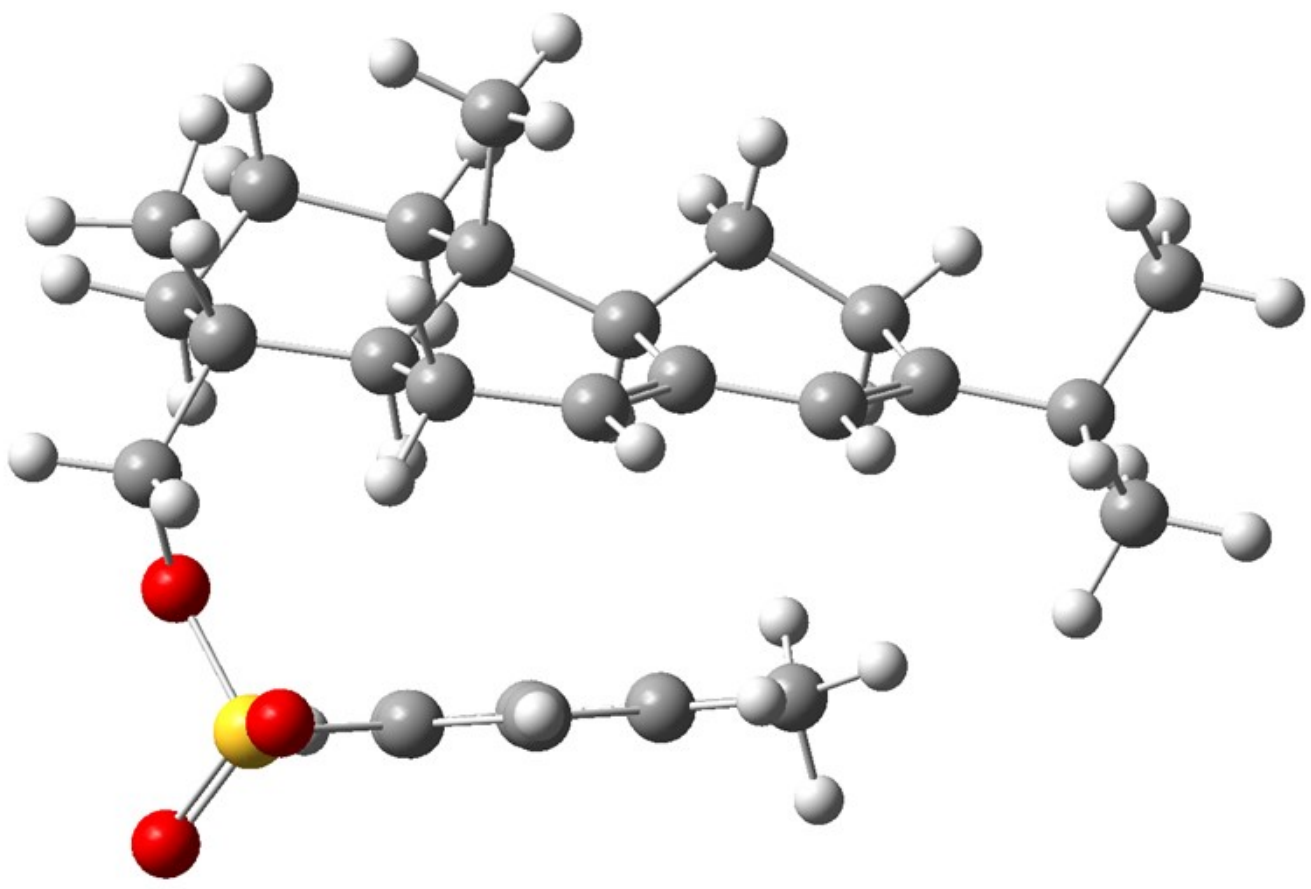

I (*n*-Octane)

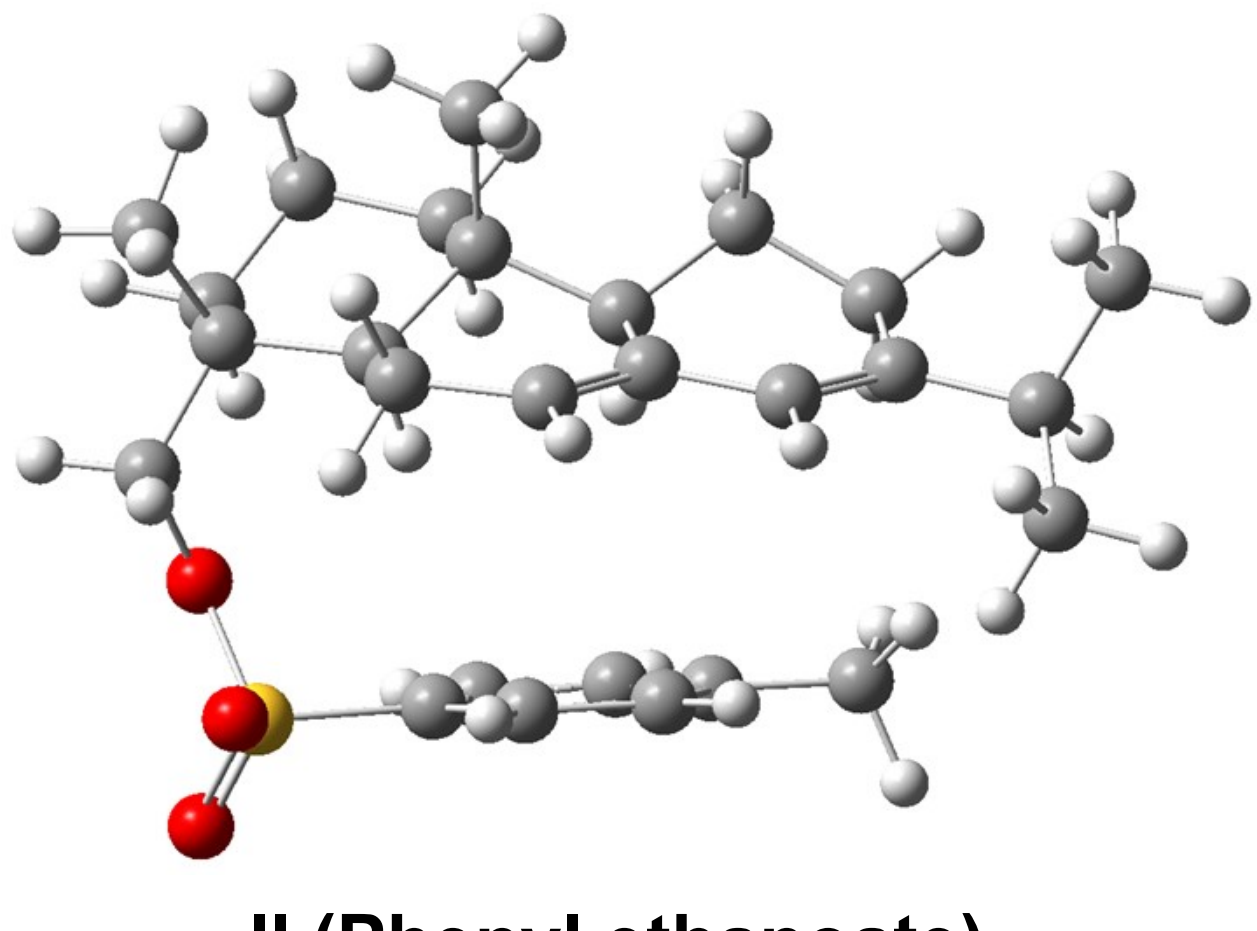

II (Phenyl ethanoate)

Figure S8. ωB97XD/def2-SVP optimized geometries of the lowest-energy conformers of Abiet-7,13-dien-18-yl *p*-toluenesulfonate (**3**) in the gas phase, *n*-octane, and pentyl ethanoate.

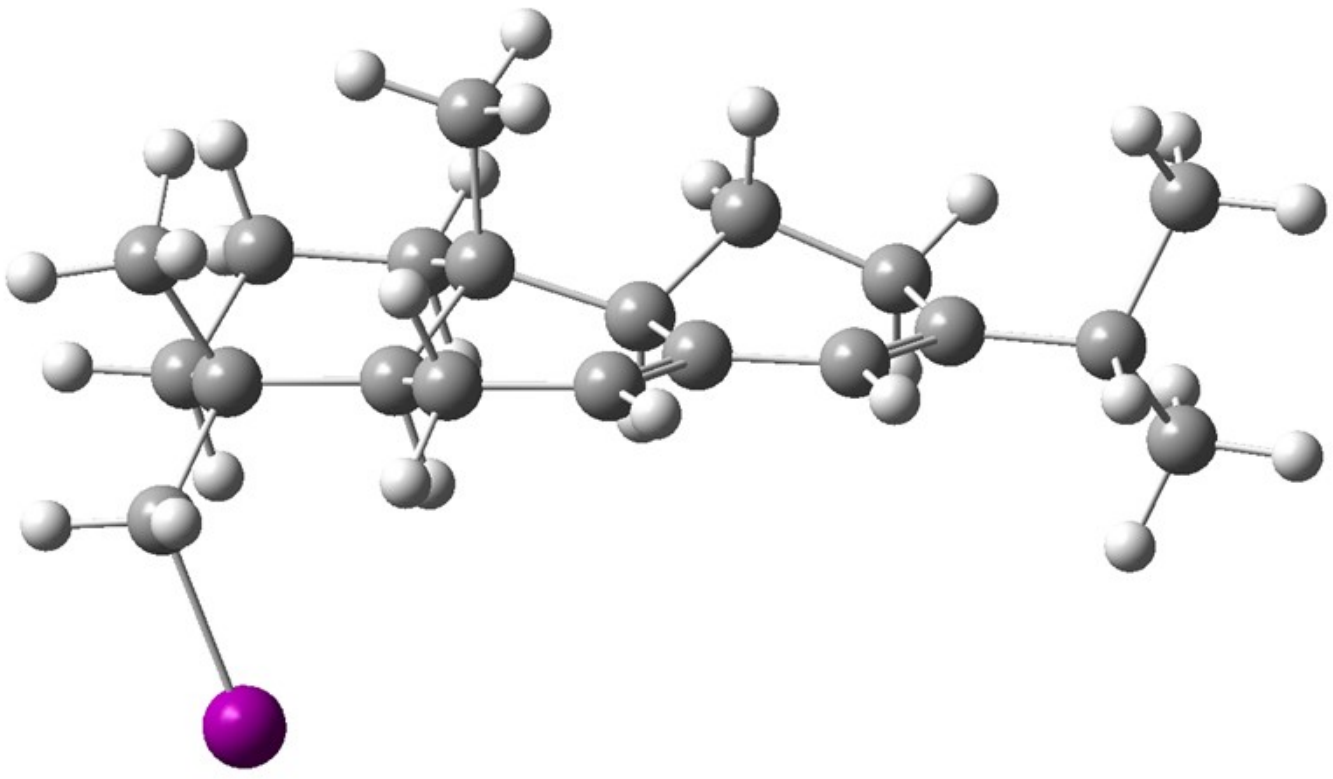

I (Gas phase)

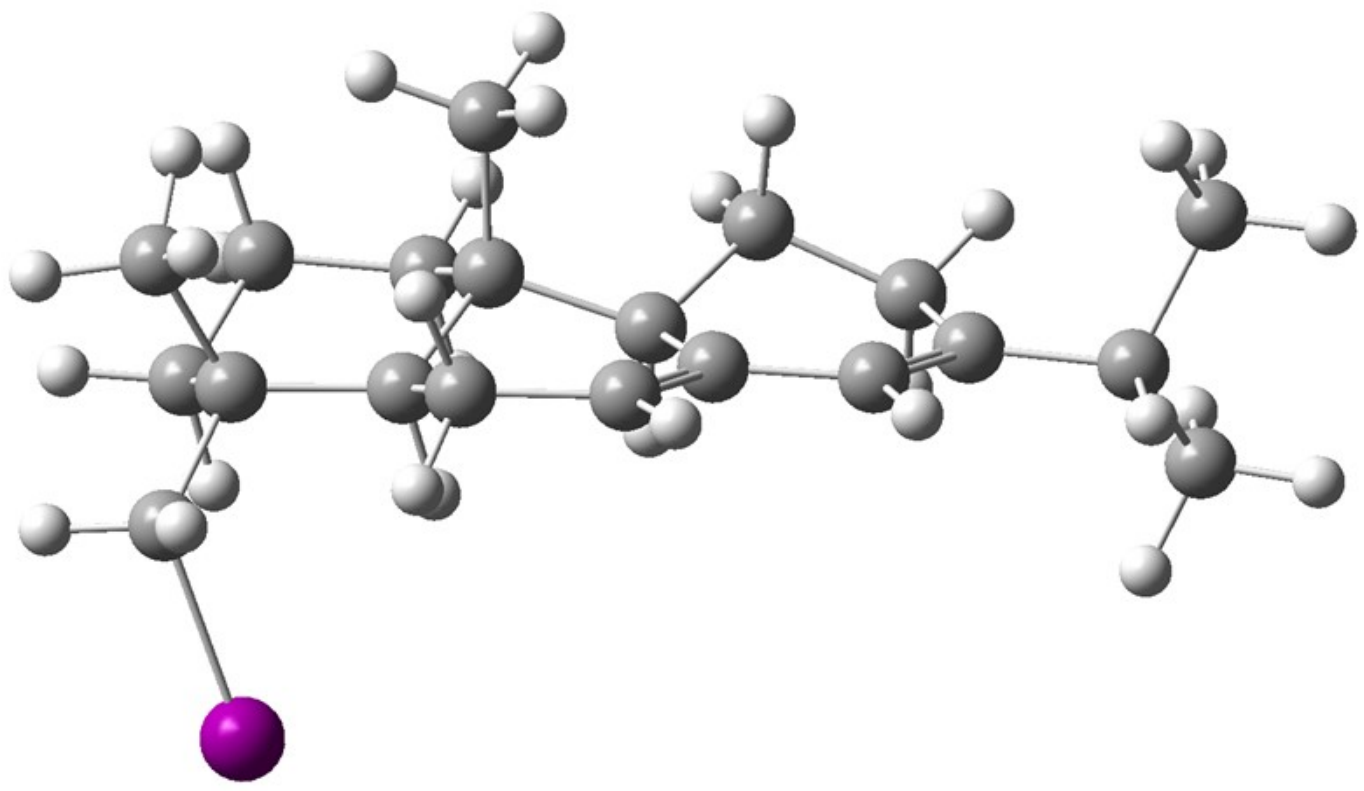

I (*n*-Octane)

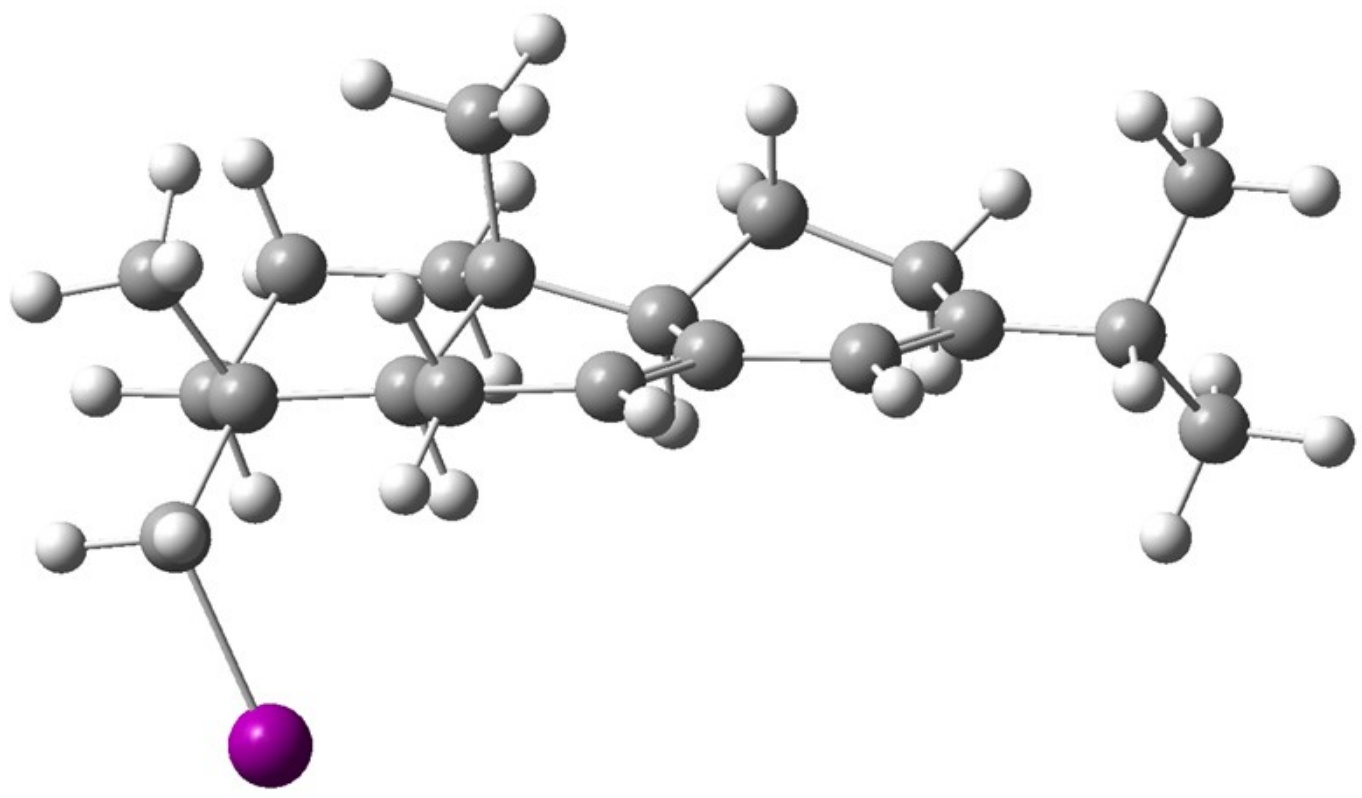

I (Pentyl ethanoate)

Figure S9. ωB97XD/def2-SVP optimized geometries of the lowest-energy conformers of 18-Iodo abiet-7,13-diene (**4**) in the gas phase, *n*-octane, and pentyl ethanoate.

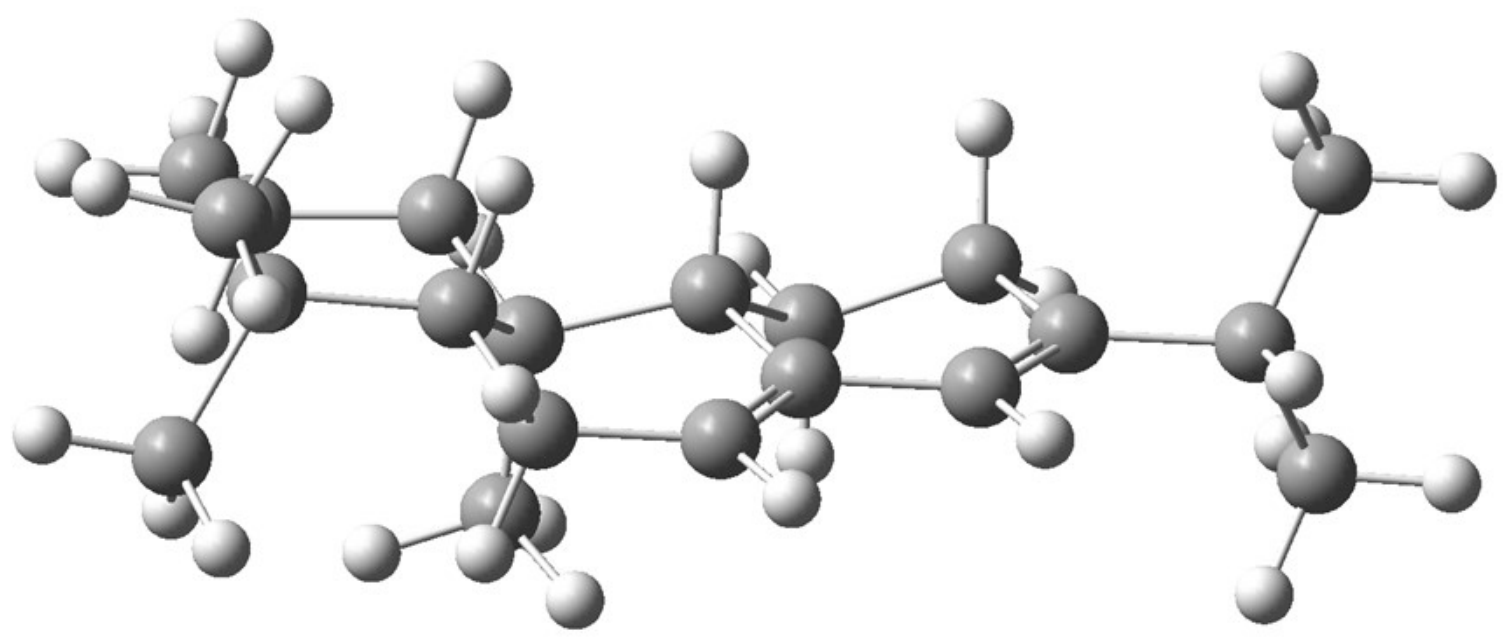

I (Gas phase)

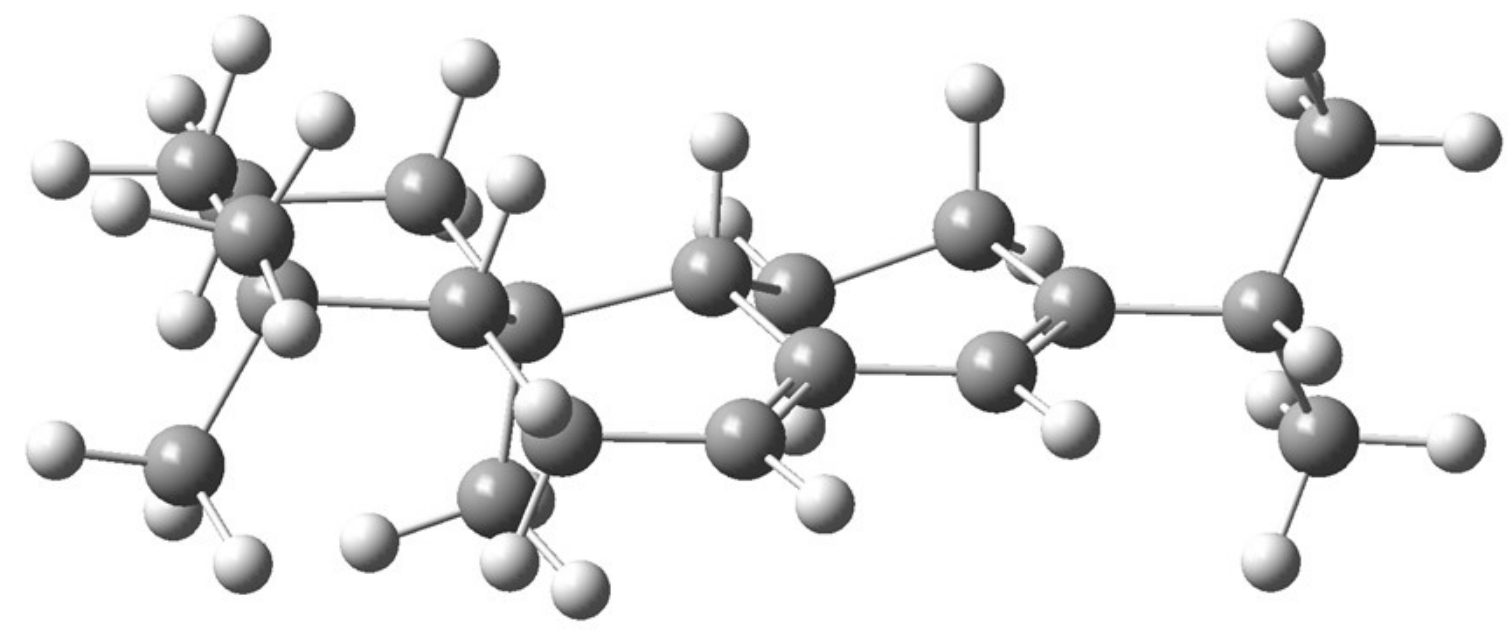

I (*n*-Octane)

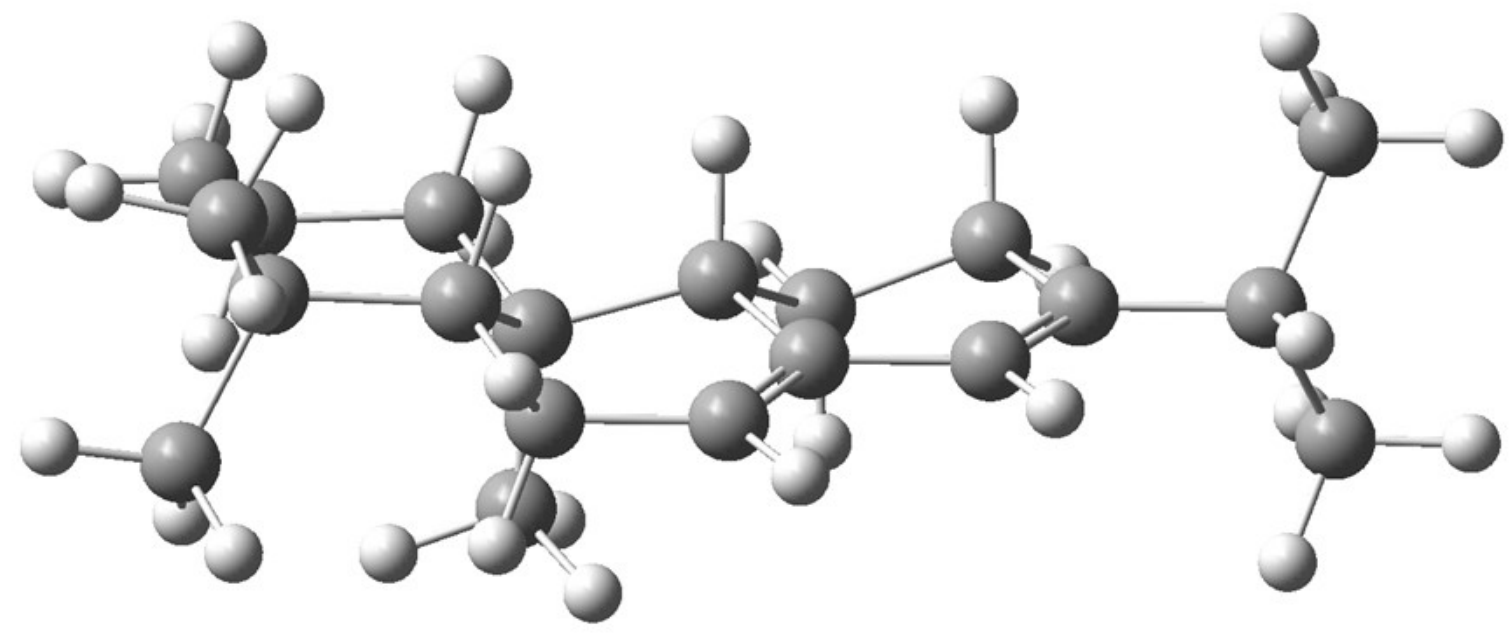

I (Pentyl ethanoate)

Figure S10. ωB97XD/def2-SVP optimized geometries of the lowest-energy conformers of Abieta-7,13-diene (**5**) in the gas phase, *n*-octane, and pentyl ethanoate.

Molecule 1

I

C	-1.25301700	2.16282300	-0.50820200
C	-2.76236900	2.35845100	-0.41773200
C	-3.49561800	1.09728800	-0.85934000
C	-3.11925200	-0.12962100	0.00455000
C	-1.56205400	-0.28783500	0.02201600
C	-1.09779400	-1.47786900	0.86295400
C	0.39157900	-1.62704500	0.85011500
C	1.23172400	-0.67291400	0.41675600
C	0.73038800	0.65915100	-0.11284000
C	-0.72379500	0.98700500	0.33830300
C	1.76571400	1.74713500	0.19383500
C	3.12093800	1.39176200	-0.40870200
C	3.58452700	0.01420700	0.00428800
C	2.68129600	-0.89958300	0.40141400
C	5.07678800	-0.24446400	-0.06116600
C	5.51564900	-1.58243300	0.52737500
C	5.59123800	-0.09273200	-1.50076700
C	-3.68340300	-1.38102200	-0.67449500
C	-3.79344300	-0.03159200	1.37852200
C	-0.73057500	1.36134600	1.82919300
O	-4.45603400	-2.15950400	-0.17899800
O	-3.22842200	-1.55261500	-1.92699700
H	-0.73765400	3.09215900	-0.21925400
H	-0.99235500	1.97767100	-1.56574300
H	-3.05403400	2.63967300	0.60719200
H	-3.06503700	3.20079500	-1.05923100
H	-3.23553600	0.88932000	-1.90902600
H	-4.58730500	1.24522500	-0.82371600
H	-1.30324200	-0.52780100	-1.02294500
H	-1.44510000	-1.38209300	1.90728900
H	-1.56309300	-2.40746900	0.49191800
H	0.80622000	-2.57030800	1.22171800
H	0.70057800	0.56281800	-1.21610600
H	1.88260200	1.85363200	1.28446500
H	1.43009500	2.72063000	-0.19338300
H	3.87647300	2.13861800	-0.11107900
H	3.06317900	1.44899300	-1.51135500
H	3.00904500	-1.89180000	0.72208400
H	5.55343000	0.55469800	0.53859200
H	6.61276100	-1.66485200	0.51021300
H	5.11149500	-2.42743200	-0.05263900
H	5.18517200	-1.69601400	1.57043200
H	6.68206400	-0.23447700	-1.54183900
H	5.37028100	0.90122500	-1.91648200
H	5.12421600	-0.84532000	-2.15621700
H	-4.88440200	-0.07744800	1.25773400
H	-3.55141000	0.90970700	1.88433900
H	-3.51227100	-0.86203400	2.03850400
H	-0.23226100	0.59568000	2.44178100
H	-0.19720600	2.30965300	1.98969100
H	-1.74427800	1.49520500	2.22343800
H	-3.63791200	-2.36404800	-2.25887300

II

C	1.13660400	2.10119900	0.64214600
C	2.62579800	2.41014200	0.53536800
C	3.45610900	1.18182200	0.88875200
C	3.14914500	-0.01724200	-0.03924300
C	1.60700400	-0.28612200	-0.03915900
C	1.21011000	-1.45443000	-0.94291000
C	-0.26382600	-1.71397600	-0.90829200
C	-1.15948600	-0.85000000	-0.40315400
C	-0.74367700	0.48343100	0.19471000
C	0.67313900	0.94120000	-0.26280300
C	-1.85917600	1.51168400	-0.02761800
C	-3.17749900	1.03128500	0.57126200
C	-3.54376300	-0.35300900	0.09365300
C	-2.58794100	-1.17906000	-0.36621700
C	-4.99926300	-0.76990100	0.13746300
C	-5.82976400	-0.03470300	-0.92269200
C	-5.60124000	-0.58899300	1.53526700
C	3.81480300	-1.26179300	0.55522100
C	3.78460400	0.20765900	-1.41671900
C	0.61911600	1.40145600	-1.72856700
O	4.62928800	-1.95339900	0.00130300
O	3.40064500	-1.53659900	1.80350500
H	0.55012900	3.00602700	0.41845000
H	0.91393500	1.83682000	1.69149900
H	2.88242900	3.23405400	1.21945400
H	2.87366800	2.76963300	-0.47653900
H	4.53353400	1.40980800	0.84292800
H	3.23420400	0.89565800	1.92875600
H	1.38878300	-0.60582100	0.99361200
H	1.52337800	-1.26959500	-1.98607600
H	1.75097300	-2.36771100	-0.64028900
H	-0.61847800	-2.66298100	-1.32456700
H	-0.68244200	0.32545400	1.28954600
H	-2.00556000	1.67385100	-1.10777500
H	-1.58264300	2.48180600	0.41134100
H	-3.98225600	1.74269500	0.32406800
H	-3.11217600	1.03061200	1.67507100
H	-2.86856200	-2.17663500	-0.72208200
H	-5.03343700	-1.84524800	-0.10548200
H	-6.87124700	-0.39231800	-0.91756700
H	-5.85472500	1.05100300	-0.73691200
H	-5.41653100	-0.19627200	-1.92923000
H	-6.63221500	-0.97367700	1.56764200
H	-5.63774900	0.47389600	1.82329700
H	-5.01270700	-1.12572800	2.29467600
H	4.87832100	0.23784800	-1.31762700
H	3.46212600	1.15463800	-1.86358100
H	3.55203400	-0.60374200	-2.11801600
H	0.16218100	0.63899300	-2.37639600
H	0.01650100	2.31666500	-1.82194100
H	1.61126500	1.63088800	-2.13344300
H	3.87303100	-2.33453600	2.07991300

III

C	-1.11594700	2.13967400	-0.57180500
C	-2.60290400	2.45336100	-0.45488800
C	-3.43909800	1.24763300	-0.86371100
C	-3.14772300	0.00425300	0.00473000
C	-1.60397200	-0.27306200	0.00053000
C	-1.21479700	-1.48281500	0.85270000
C	0.25841700	-1.74592500	0.81682600
C	1.16060200	-0.86503500	0.35498900
C	0.75280400	0.49379300	-0.18773300
C	-0.66430100	0.93768600	0.28224900
C	1.87099900	1.50731600	0.08385000
C	3.19104100	1.04701400	-0.52704400
C	3.54948000	-0.35748700	-0.10613100
C	2.58782400	-1.19836800	0.31273900
C	5.00338700	-0.77860400	-0.16028000
C	5.61280200	-0.54258500	-1.54662800
C	5.83210200	-0.09202500	0.93332400
C	-3.75521200	-1.19762300	-0.72791800
C	-3.79185200	0.16544400	1.38845200
C	-0.61832600	1.33001000	1.76809700
O	-3.60785000	-1.42533600	-1.90117100
O	-4.46488000	-2.02011400	0.05867100
H	-0.52354500	3.02919400	-0.30627700
H	-0.89250600	1.91984300	-1.63100500
H	-2.85249800	3.30767100	-1.10324200
H	-2.85004500	2.77186700	0.57130500
H	-4.51523600	1.48193600	-0.81359200
H	-3.21751800	0.99512300	-1.91213800
H	-1.39323300	-0.54697500	-1.04805300
H	-1.53413700	-1.34410600	1.90139800
H	-1.75477300	-2.38005100	0.50589700
H	0.60633800	-2.71384200	1.19332900
H	0.69588300	0.38247000	-1.28836100
H	2.01138300	1.62386700	1.17074700
H	1.60117700	2.49591600	-0.31612000
H	3.99698200	1.74430500	-0.24582800
H	3.13185500	1.09268400	-1.63020800
H	2.86221600	-2.21150400	0.62702000
H	5.03168800	-1.86326800	0.03786600
H	6.64207900	-0.93070700	-1.59031300
H	5.65573100	0.53118200	-1.78992800
H	5.02528100	-1.04427000	-2.33035000
H	6.87183500	-0.45445000	0.91835000
H	5.86335400	1.00028600	0.79274600
H	5.41313200	-0.29295100	1.93038300
H	-4.88638800	0.19637600	1.29087800
H	-3.48088800	1.10151700	1.86669500
H	-3.55042600	-0.66233000	2.06674200
H	-0.16342600	0.53917000	2.38257300
H	-0.01805600	2.24099600	1.90703000
H	-1.61330500	1.53899400	2.17763200
H	-4.77306900	-2.74127200	-0.50927100

IV

C	-1.26544200	2.12320000	-0.67164600
C	-2.76277400	2.35216900	-0.49783500
C	-3.54103100	1.07990600	-0.81199900
C	-3.12866700	-0.09752600	0.10266700
C	-1.57570600	-0.28295800	0.03389400
C	-1.07624100	-1.42427300	0.92093600
C	0.40636700	-1.60450300	0.81981900
C	1.23135500	-0.69603600	0.27378700
C	0.71745400	0.61044900	-0.30653800
C	-0.70016400	0.99343500	0.21333000
C	1.78446900	1.69848000	-0.13710800
C	3.09878400	1.28206000	-0.78798500
C	3.56122600	-0.07873100	-0.32740600
C	2.67230300	-0.95363000	0.17382000
C	5.04574300	-0.36180700	-0.44989600
C	5.82322200	0.33442200	0.67765100
C	5.40217400	-1.84583900	-0.51039800
C	-3.75161000	-1.37840800	-0.46006500
C	-3.71352000	0.09751000	1.50685100
C	-0.60615500	1.46160700	1.67454300
O	-4.50107800	-2.11176100	0.13074800
O	-3.38009000	-1.63418900	-1.72569300
H	-0.71898800	3.05907800	-0.47610000
H	-1.07645000	1.86699800	-1.72959300
H	-3.09401800	3.15885200	-1.17029100
H	-2.98350100	2.70115300	0.52400500
H	-4.62597900	1.25004600	-0.71783300
H	-3.35116100	0.80224300	-1.86061100
H	-1.38714600	-0.59475600	-1.00709800
H	-1.35139900	-1.25196400	1.97689200
H	-1.58036600	-2.36654400	0.64487600
H	0.82937700	-2.53186300	1.22082500
H	0.61551100	0.44478200	-1.39719500
H	1.96807300	1.87584500	0.93498900
H	1.43842300	2.64944400	-0.56863900
H	3.87761000	2.03384800	-0.57941700
H	2.99133800	1.26711200	-1.88904500
H	3.00630000	-1.93737500	0.51562200
H	5.36891200	0.09746900	-1.40296900
H	6.90900300	0.21802900	0.53676200
H	5.55466600	-0.10432200	1.65153000
H	5.60648800	1.41202100	0.72479000
H	6.47611000	-1.97445600	-0.71366200
H	5.19011400	-2.35053700	0.44546900
H	4.83803900	-2.36408200	-1.29981400
H	-4.81059900	0.06882800	1.45642800
H	-3.42280600	1.06176500	1.93833600
H	-3.40692900	-0.69810900	2.19764500
H	-0.08046000	0.72734200	2.30250700
H	-0.05070300	2.40850400	1.73999400
H	-1.59052700	1.63861400	2.12242600
H	-3.82031100	-2.45753600	-1.97979900

V

C	-1.12529200	2.07301200	-0.67407700
C	-2.61326100	2.37704900	-0.54785400
C	-3.43891000	1.14314400	-0.88582900
C	-3.11545500	-0.05962900	0.03984700
C	-1.57895500	-0.30851700	0.06079400
C	-1.17215200	-1.43801100	1.00926700
C	0.30369400	-1.68798400	0.97884100
C	1.19176500	-0.83940000	0.43596900
C	0.76435400	0.46926700	-0.20687300
C	-0.65126800	0.93247700	0.24798500
C	1.87697400	1.50996100	-0.03232600
C	3.19238200	1.01321500	-0.62460000
C	3.56958600	-0.35024500	-0.09786700
C	2.62148500	-1.16266600	0.40047700
C	5.02672400	-0.76174000	-0.13859100
C	5.86354800	0.01857800	0.88364200
C	5.61493900	-0.63378100	-1.54807000
C	-3.77148600	-1.25975400	-0.65245500
C	-3.76391900	0.14510400	1.41886700
C	-0.58617200	1.43333900	1.70077800
O	-3.21850200	-2.05104700	-1.37224400
O	-5.09447700	-1.32506400	-0.43644600
H	-0.53873100	2.98343600	-0.47500600
H	-0.91727500	1.79026400	-1.72163800
H	-2.85140000	2.73482300	0.46715200
H	-2.88653300	3.19722500	-1.23001100
H	-3.21808500	0.85375700	-1.92801300
H	-4.51744400	1.35879900	-0.83707600
H	-1.35738600	-0.67488100	-0.95523400
H	-1.48068400	-1.21548600	2.04715400
H	-1.70320900	-2.36209300	0.72963600
H	0.66629800	-2.61990600	1.42581900
H	0.69283600	0.27169200	-1.29451300
H	2.03283500	1.71283700	1.03965300
H	1.59282200	2.46211600	-0.50467200
H	3.99580200	1.73725200	-0.41166600
H	3.11704800	0.97043200	-1.72692800
H	2.90964800	-2.14509700	0.79079000
H	5.06840900	-1.82646300	0.14636400
H	6.90683300	-0.33391900	0.88277100
H	5.88113800	1.09622300	0.65493300
H	5.46048800	-0.10499400	1.89967200
H	6.64767200	-1.01426800	-1.57498900
H	5.64291800	0.41698700	-1.87849800
H	5.02205700	-1.20324200	-2.27971400
H	-4.84558500	0.29711200	1.31273600
H	-3.35143300	1.02253400	1.92921300
H	-3.61492600	-0.72392100	2.07496900
H	-0.13613900	0.68579700	2.36998300
H	0.02718600	2.34387100	1.76515600
H	-1.57344500	1.68998800	2.10258100
H	-5.41935000	-2.07249600	-0.95865700

VI

C	-1.24817200	2.10372500	-0.68608300
C	-2.74239000	2.33109800	-0.49079800
C	-3.51953700	1.05908100	-0.80140800
C	-3.09427600	-0.13102000	0.09904200
C	-1.54790100	-0.30212000	0.04800000
C	-1.04178100	-1.41554800	0.96714800
C	0.44179400	-1.59018100	0.86768700
C	1.26111800	-0.69457500	0.29293600
C	0.73880200	0.59411300	-0.31908400
C	-0.67601100	0.98263200	0.20357400
C	1.80404900	1.68934400	-0.18852400
C	3.11459000	1.25769300	-0.83741500
C	3.58526500	-0.08709700	-0.33955600
C	2.70232700	-0.94992200	0.19212600
C	5.07005300	-0.36835300	-0.46338500
C	5.43189900	-1.85220600	-0.47887400
C	5.85299700	0.36649000	0.63550000
C	-3.72015700	-1.35898600	-0.57196900
C	-3.68870800	0.03304100	1.50753400
C	-0.56967500	1.47610600	1.65648700
O	-3.16106000	-2.11872500	-1.32049100
O	-5.02750300	-1.49077400	-0.29866000
H	-0.69989200	3.04145100	-0.50544000
H	-1.07367200	1.83882000	-1.74437600
H	-2.95232600	2.67052300	0.53666500
H	-3.08706900	3.14037800	-1.15335600
H	-4.60419500	1.21975700	-0.70229700
H	-3.33202200	0.78732400	-1.85481400
H	-1.35531600	-0.65058300	-0.98018000
H	-1.31342400	-1.21561200	2.01982800
H	-1.53800300	-2.36339300	0.70346200
H	0.87034300	-2.50560900	1.28976600
H	0.62696700	0.39888000	-1.40373900
H	1.99606900	1.89792800	0.87652200
H	1.45284200	2.62693400	-0.64461000
H	3.89231500	2.01819400	-0.65812000
H	2.99782700	1.20916400	-1.93655200
H	3.04150300	-1.92253300	0.55970300
H	5.38480500	0.06116500	-1.43305300
H	6.50494700	-1.98340300	-0.68538700
H	5.22819900	-2.32675300	0.49412800
H	4.86421300	-2.39747800	-1.24721700
H	6.93827500	0.24973300	0.49066500
H	5.59306900	-0.04174400	1.62489300
H	5.63225000	1.44424400	0.64962200
H	-4.78034700	0.13082300	1.45142400
H	-3.29800800	0.92730000	2.00565000
H	-3.46671400	-0.83099800	2.14937800
H	-0.05268300	0.74718900	2.29728400
H	-0.00031000	2.41587900	1.70162200
H	-1.54912300	1.67961500	2.10516700
H	-5.33773500	-2.25110800	-0.81106300

VII

C	-1.23203800	2.19318800	-0.45757900
C	-2.73948100	2.39668400	-0.36152800
C	-3.48011300	1.15696000	-0.84612500
C	-3.11907300	-0.10408800	-0.03111400
C	-1.55973200	-0.27244900	-0.01192300
C	-1.10290400	-1.49279900	0.79001500
C	0.38590900	-1.64817300	0.78064900
C	1.23297100	-0.68485000	0.38325700
C	0.73991700	0.66551300	-0.10517200
C	-0.71463700	0.98565200	0.34995400
C	1.77839500	1.73835100	0.24201200
C	3.13518200	1.39645300	-0.36480800
C	3.59067300	0.00418900	0.00540600
C	2.68143200	-0.91798700	0.36786800
C	5.08198400	-0.25906300	-0.06128600
C	5.51174900	-1.61786600	0.48476400
C	5.60440300	-0.06236200	-1.49254200
C	-3.62433000	-1.30845300	-0.83391600
C	-3.80077500	-0.05645800	1.34318000
C	-0.72855600	1.30778800	1.85315600
O	-3.44188300	-1.46526900	-2.01384700
O	-4.28298800	-2.21968600	-0.10267300
H	-0.70928800	3.10778600	-0.13671100
H	-0.97197200	2.04077700	-1.52024800
H	-3.03553600	3.26195000	-0.97485000
H	-3.02930600	2.64719000	0.67231600
H	-4.57089200	1.31308600	-0.80993300
H	-3.22062000	0.97173900	-1.89988200
H	-1.30862700	-0.47844200	-1.06717600
H	-1.45520100	-1.43068400	1.83549400
H	-1.56834700	-2.40760500	0.38582600
H	0.79362700	-2.60568300	1.12224300
H	0.71454700	0.60484900	-1.21094000
H	1.88984700	1.80908600	1.33617400
H	1.44952900	2.72536600	-0.11538800
H	3.89244100	2.12985400	-0.03938000
H	3.08307200	1.48953100	-1.46523100
H	3.00295300	-1.92185500	0.65701500
H	5.55912800	0.51758900	0.56701000
H	6.60854300	-1.70490500	0.47009700
H	5.10653200	-2.44121200	-0.12488100
H	5.17563500	-1.76433900	1.52192000
H	6.69478000	-0.20774000	-1.53276300
H	5.39005700	0.94576700	-1.87629900
H	5.13733200	-0.79063800	-2.17482100
H	-4.89247800	-0.10032200	1.22177100
H	-3.56925700	0.87583600	1.87131600
H	-3.51229600	-0.89599400	1.98779900
H	-0.23452500	0.52069800	2.44160800
H	-0.19493500	2.24920000	2.04951100
H	-1.74446500	1.42956500	2.24615500
H	-4.52716700	-2.93219200	-0.71121300

VIII

C	-1.24483000	2.15714200	-0.62323400
C	-2.74029300	2.39104700	-0.44477300
C	-3.52578600	1.13604500	-0.80286000
C	-3.12928600	-0.07772900	0.06575300
C	-1.57415900	-0.27021500	-0.00223900
C	-1.08228200	-1.44463700	0.84617600
C	0.40033100	-1.62699200	0.74873200
C	1.23252500	-0.70462900	0.23870300
C	0.72668400	0.62183900	-0.30080100
C	-0.69173200	0.99379500	0.22453400
C	1.79671700	1.69965100	-0.08988200
C	3.11335400	1.29954300	-0.74621600
C	3.56805300	-0.07740100	-0.32800900
C	2.67294600	-0.96491000	0.13881000
C	5.05205500	-0.36211300	-0.45229300
C	5.82685400	0.29764300	0.69875800
C	5.40369600	-1.84489600	-0.55539300
C	-3.69985700	-1.32091800	-0.62650800
C	-3.72177800	0.06616000	1.47425300
C	-0.60503000	1.40921800	1.70224400
O	-3.59175800	-1.55521900	-1.80274500
O	-4.32663300	-2.17157700	0.19956800
H	-0.69110300	3.08114800	-0.39449100
H	-1.05555600	1.93468800	-1.68852300
H	-2.95991200	2.70957500	0.58764000
H	-3.06432300	3.22151800	-1.09116200
H	-3.33613700	0.88175800	-1.85709100
H	-4.61001400	1.31201700	-0.70817200
H	-1.39350900	-0.54670200	-1.05577200
H	-1.36326300	-1.30874000	1.90615800
H	-1.58621400	-2.37490600	0.53391000
H	0.81655900	-2.56965300	1.12007300
H	0.62930900	0.49232900	-1.39660600
H	1.97438100	1.84166500	0.98851600
H	1.45698800	2.66527800	-0.49271900
H	3.89375800	2.04096200	-0.50838800
H	3.01235500	1.32100500	-1.84772900
H	3.00090800	-1.96101700	0.44918000
H	5.38093700	0.12419300	-1.38988000
H	6.91286000	0.18180200	0.55913700
H	5.55260600	-0.16885600	1.65806600
H	5.61339900	1.37411000	0.77677700
H	6.47815900	-1.97116800	-0.75736000
H	5.18526100	-2.37705500	0.38402400
H	4.84164700	-2.33734600	-1.36258700
H	-4.81949100	0.03467800	1.42402100
H	-3.44386900	1.02448700	1.92817400
H	-3.40521900	-0.73759100	2.15049600
H	-0.08363300	0.65242200	2.30664800
H	-0.04868900	2.35237700	1.80446200
H	-1.59191300	1.57165300	2.15062400
H	-4.61825500	-2.91611400	-0.34654000

IX

C	-1.23880000	2.14211400	-0.53398400
C	-2.74656200	2.33237900	-0.42038300
C	-3.47670900	1.06696900	-0.84949400
C	-3.08306000	-0.16516200	0.00787500
C	-1.53329400	-0.30669200	0.04336500
C	-1.06102600	-1.46281800	0.92719200
C	0.42928800	-1.60436800	0.91285500
C	1.26302700	-0.66329600	0.44073200
C	0.75204200	0.64826900	-0.12945200
C	-0.69941700	0.98192900	0.32542200
C	1.78648400	1.74949200	0.12979300
C	3.13718600	1.37728000	-0.47308100
C	3.60962200	0.01677100	-0.01574000
C	2.71314800	-0.88539800	0.42108600
C	5.10211300	-0.23898000	-0.08616100
C	5.60243600	-0.13710600	-1.53512100
C	5.55123700	-1.55389700	0.54510100
C	-3.64239600	-1.36314600	-0.76801000
C	-3.76530200	-0.09052500	1.38377600
C	-0.69189200	1.39366400	1.80721600
O	-3.02450400	-2.06843400	-1.52352300
O	-4.96060100	-1.53324500	-0.58167400
H	-0.72204100	3.07716900	-0.26709900
H	-0.99408200	1.94121700	-1.59245300
H	-3.02629800	2.60933900	0.60917400
H	-3.06637900	3.17202600	-1.05710900
H	-3.21939600	0.85896000	-1.90267800
H	-4.56850000	1.20310300	-0.80938700
H	-1.27049500	-0.59085000	-0.98897300
H	-1.51884300	-2.40263400	0.57894700
H	-1.40159900	-1.33009400	1.97040200
H	0.85039300	-2.53364700	1.31153300
H	0.71073200	0.51476600	-1.22834600
H	1.91411200	1.89424900	1.21484600
H	1.44451300	2.70816900	-0.28789000
H	3.89285600	2.13681200	-0.20957000
H	3.06820500	1.39520900	-1.57640300
H	3.04700900	-1.86502700	0.77268800
H	5.58189700	0.58239600	0.48027300
H	6.69342600	-0.27622000	-1.58167800
H	5.13200300	-0.91421900	-2.15868600
H	5.37346000	0.84053600	-1.98393900
H	6.64840100	-1.63391500	0.51948200
H	5.14362200	-2.42007000	-0.00018000
H	5.23178200	-1.63109500	1.59490300
H	-4.85352000	-0.01214100	1.26539900
H	-3.42675500	0.78330800	1.95154400
H	-3.56230500	-0.98349900	1.99136400
H	-0.20347900	0.63698600	2.43812600
H	-0.14182700	2.33672800	1.93974700
H	-1.70062200	1.55890100	2.20376900
H	-5.22419400	-2.26789600	-1.15414900

Molecule 2

I

C	-1.39942800	1.95570100	-0.66284100
C	-2.89712200	2.21263600	-0.55363200
C	-3.68152700	0.94775200	-0.87640500
C	-3.33079700	-0.23764600	0.04092700
C	-1.78386900	-0.43903700	0.04843400
C	-1.31917700	-1.58508300	0.95303400
C	0.16513900	-1.77798000	0.91963300
C	1.02121600	-0.87913300	0.40784300
C	0.54415900	0.42914300	-0.19698800
C	-0.89273800	0.82470400	0.25467400
C	1.61169100	1.50826900	0.02136700
C	2.95195200	1.08629600	-0.57277600
C	3.38058300	-0.27774800	-0.08951100
C	2.46297800	-1.14439200	0.37340100
C	4.85353500	-0.62814700	-0.13046800
C	5.44804300	-0.42555800	-1.52849700
C	5.64851800	0.14856900	0.92732200
C	-4.00212100	-1.50296100	-0.55169800
C	-3.97293300	-0.03650300	1.42156200
C	-0.86645200	1.29794900	1.71706700
O	-3.51851400	-1.90197700	-1.80633000
H	-1.17231100	1.68377800	-1.70939200
H	-0.84235800	2.88268400	-0.45453600
H	-3.18529700	3.01863000	-1.24679000
H	-3.14966900	2.57867600	0.45563200
H	-3.47135200	0.64736000	-1.91664300
H	-4.76656900	1.13877800	-0.81837000
H	-1.55464000	-0.74424300	-0.98915200
H	-1.63979600	-1.41534400	1.99680100
H	-1.80870500	-2.52972200	0.65956500
H	0.56194700	-2.70704200	1.34338800
H	0.49196300	0.26244500	-1.29111400
H	1.74757300	1.68251600	1.10113900
H	1.29225900	2.46234300	-0.42301700
H	3.72311300	1.83453500	-0.32711400
H	2.88905000	1.07821400	-1.67669200
H	2.78815900	-2.12656300	0.73428500
H	4.93653000	-1.69983200	0.11697500
H	6.49542000	-0.76323500	-1.55838000
H	5.43662000	0.63673200	-1.82068700
H	4.88532200	-0.99136100	-2.28636200
H	6.70508900	-0.16164500	0.92478500
H	5.62452900	1.23341600	0.73668200
H	5.24180600	-0.02705900	1.93416400
H	-5.06940800	-0.10429400	1.33231000
H	-3.75232400	0.94728200	1.85446500
H	-3.65785800	-0.79959700	2.14807800
H	-0.36880700	0.56647900	2.37108700
H	-0.31723000	2.24669600	1.80690700
H	-1.87255100	1.47123100	2.11618100
H	-2.66958100	-2.33578600	-1.68692800
H	-3.95051300	-2.33127300	0.18114200
H	-5.07220000	-1.27997500	-0.69245100

II

C	-1.39567900	1.95981400	-0.64499800
C	-2.89231700	2.22022000	-0.52853000
C	-3.68084000	0.95928300	-0.85822100
C	-3.32706200	-0.23690600	0.04657700
C	-1.78126000	-0.44505800	0.03732600
C	-1.31829700	-1.60789700	0.91959900
C	0.16764600	-1.78705300	0.90187100
C	1.02466900	-0.88387900	0.40032300
C	0.54583600	0.42401900	-0.20348000
C	-0.88950500	0.81550900	0.25625300
C	1.61278600	1.50476400	0.01064800
C	2.95385000	1.08213700	-0.58158500
C	3.38481500	-0.27831500	-0.08985400
C	2.46727400	-1.14467000	0.37343800
C	4.85878600	-0.62521600	-0.12489500
C	5.65004200	0.16008800	0.92934200
C	5.45620400	-0.43072700	-1.52286700
C	-4.00860400	-1.50115400	-0.53592500
C	-3.96268200	-0.03049800	1.43011100
C	-0.85837800	1.27262400	1.72390500
O	-3.46585600	-1.98093800	-1.73575200
H	-0.83801000	2.88395200	-0.42653100
H	-1.17030700	1.70225800	-1.69590500
H	-3.18336400	3.03388300	-1.21150800
H	-3.14227800	2.57354300	0.48530200
H	-4.76619700	1.15010800	-0.80277000
H	-3.46251700	0.69389300	-1.90976300
H	-1.57073500	-0.75945200	-0.99969600
H	-1.65261200	-1.47502500	1.96494300
H	-1.78844600	-2.54326900	0.57596200
H	0.56506100	-2.71757200	1.32191400
H	0.48773200	0.25692100	-1.29717000
H	1.74913900	1.68301800	1.08972100
H	1.29344600	2.45752400	-0.43705900
H	3.72315400	1.83378800	-0.34011900
H	2.89054100	1.06788800	-1.68547700
H	2.79309200	-2.12507900	0.73855600
H	4.94366900	-1.69507900	0.12971500
H	6.70756300	-0.14715200	0.93097600
H	5.62343500	1.24371600	0.73188800
H	5.24165300	-0.01023500	1.93643500
H	6.50474700	-0.76537100	-1.54827900
H	5.44208800	0.62951700	-1.82252900
H	4.89662400	-1.00350400	-2.27781800
H	-5.05926300	-0.11123200	1.35103200
H	-3.74936400	0.95770200	1.85598300
H	-3.63161100	-0.78590500	2.15688500
H	-0.36912800	0.52726100	2.36816200
H	-0.29914500	2.21441400	1.82587900
H	-1.86228800	1.45136900	2.12624300
H	-3.61265500	-1.32629100	-2.42395900
H	-5.09018700	-1.28221800	-0.64523100
H	-3.93068100	-2.32836900	0.18600100

III

C	1.51993100	2.00947700	0.49919800
C	3.03481200	2.14210900	0.40611400
C	3.71290800	0.85200200	0.84781500
C	3.28773400	-0.37443400	0.02024900
C	1.73017100	-0.45736600	-0.00923500
C	1.19442400	-1.63465000	-0.83037300
C	-0.30082400	-1.70959900	-0.82044000
C	-1.09421000	-0.70999800	-0.40285500
C	-0.52734000	0.60182700	0.10811700
C	0.94394000	0.84941000	-0.33768900
C	-1.50550500	1.73519700	-0.22219500
C	-2.88008300	1.45920800	0.37791100
C	-3.41101500	0.09993800	-0.01381700
C	-2.55324200	-0.86479100	-0.39072400
C	-4.91461800	-0.08207200	0.04720500
C	-5.42822300	0.12087200	1.48077700
C	-5.41767200	-1.40630200	-0.52076600
C	3.84723400	-1.63435500	0.72895900
C	3.96875700	-0.33662700	-1.35592300
C	0.98200600	1.20328500	-1.83316400
O	3.31012400	-1.89142300	1.99878400
H	1.04066900	2.95621000	0.20421900
H	1.25344000	1.84151200	1.55821700
H	3.33282000	2.40432200	-0.62286500
H	3.37217900	2.97780800	1.03929500
H	3.46163700	0.65438200	1.90343500
H	4.81034000	0.95484400	0.80069800
H	1.45883700	-0.65842300	1.04355600
H	1.54763300	-1.57622500	-1.87579700
H	1.60246800	-2.58677300	-0.44942800
H	-0.76035600	-2.63631600	-1.18133000
H	-0.50856700	0.52161200	1.21303400
H	-1.61063300	1.82913300	-1.31526600
H	-1.12372700	2.69689700	0.15078200
H	-3.59554700	2.23786000	0.06324800
H	-2.82504100	1.53250500	1.47971700
H	-2.92892000	-1.84461500	-0.69684100
H	-5.34695200	0.72941800	-0.56930700
H	-6.52498100	0.03491100	1.51756000
H	-5.00321200	-0.64241300	2.15222300
H	-5.15963400	1.10968600	1.88026500
H	-6.51763400	-1.43236100	-0.50951500
H	-5.06080800	-2.26044100	0.07664100
H	-5.08753800	-1.55511700	-1.55949100
H	5.05502900	-0.47826100	-1.23446600
H	3.83164000	0.62146900	-1.87272600
H	3.60984500	-1.13174800	-2.02554800
H	0.44297700	0.46049900	-2.43986800
H	0.50877000	2.17996100	-2.01183500
H	2.00593700	1.26882900	-2.21887200
H	2.42932900	-2.26043000	1.89269900
H	3.74540100	-2.51349000	0.06369400
H	4.92849600	-1.48265400	0.87860700

IV

C	1.51691400	2.01374500	0.47656000
C	3.03110500	2.14923300	0.37549200
C	3.71396300	0.86436400	0.82619500
C	3.28455000	-0.37274900	0.01387200
C	1.72732900	-0.46195700	0.00408000
C	1.19105700	-1.65700800	-0.78891400
C	-0.30441900	-1.71927000	-0.79540200
C	-1.09813200	-0.71501500	-0.39096300
C	-0.52931100	0.59761100	0.11528000
C	0.93992000	0.83947600	-0.33978000
C	-1.50693900	1.73202900	-0.21352900
C	-2.88219000	1.45679900	0.38567300
C	-3.41514500	0.09971200	-0.01127000
C	-2.55750600	-0.86578200	-0.38618300
C	-4.91943600	-0.07882000	0.04421800
C	-5.43749900	0.12023700	1.47670000
C	-5.42342400	-1.40014400	-0.52969800
C	3.85640800	-1.63209500	0.71295900
C	3.95752000	-0.32963300	-1.36684500
C	0.97109000	1.17310000	-1.84032800
O	3.25771200	-1.96804600	1.93482800
H	1.03696700	2.95603900	0.16918500
H	1.25326900	1.86312600	1.53924000
H	3.37213900	2.99294400	0.99601000
H	3.32528400	2.39638300	-0.65771100
H	4.81160800	0.96738900	0.78099200
H	3.45753000	0.70478500	1.89043600
H	1.47543300	-0.67058100	1.05844000
H	1.55504800	-1.64079300	-1.83269100
H	1.57935800	-2.59276000	-0.35565200
H	-0.76485300	-2.64802100	-1.14995300
H	-0.50405000	0.51945500	1.22013900
H	-1.61255800	1.82726200	-1.30645900
H	-1.12532200	2.69366500	0.16029900
H	-3.59575300	2.23817800	0.07307500
H	-2.82710500	1.52674200	1.48776900
H	-2.93358200	-1.84489100	-0.69417300
H	-5.34849300	0.73553200	-0.57098600
H	-6.53472400	0.03770200	1.50980500
H	-5.01682800	-0.64685500	2.14655300
H	-5.16684700	1.10668800	1.88078400
H	-6.52353200	-1.42412600	-0.52242700
H	-5.07012600	-2.25684400	0.06613000
H	-5.08954800	-1.54627800	-1.56760900
H	5.04227900	-0.49233600	-1.25696300
H	3.83334600	0.63581600	-1.87277600
H	3.57673700	-1.11094900	-2.03987200
H	0.43913800	0.41474400	-2.43352100
H	0.48696600	2.14185300	-2.03326000
H	1.99303100	1.24314200	-2.23059100
H	3.44141400	-1.27045700	2.56996400
H	4.94962900	-1.48713300	0.82943100
H	3.72731600	-2.50804600	0.05905700

V

C	-1.52911100	1.95887000	-0.68304800
C	-3.03109600	2.13520200	-0.49725500
C	-3.76074200	0.82690200	-0.77353500
C	-3.29627500	-0.33358000	0.12760100
C	-1.74324100	-0.45365700	0.04627000
C	-1.17288400	-1.57450600	0.91968800
C	0.31822400	-1.66984500	0.83106800
C	1.09793900	-0.72764000	0.27755800
C	0.51625700	0.54169800	-0.31791100
C	-0.91565100	0.85841400	0.20618600
C	1.52836100	1.68436700	-0.16975700
C	2.86202500	1.32431200	-0.81501500
C	3.39445200	-0.00208000	-0.33011000
C	2.55039000	-0.91245600	0.18499300
C	4.89184600	-0.20985000	-0.44686900
C	5.63151300	0.55045800	0.66457200
C	5.32537500	-1.67419200	-0.47519900
C	-3.93058000	-1.64308300	-0.40604100
C	-3.87597500	-0.14288200	1.53767300
C	-0.84067300	1.33831900	1.66500300
O	-3.41960300	-2.10765900	-1.62562100
H	-1.01617900	2.91650700	-0.50261800
H	-1.33962200	1.69945700	-1.74055200
H	-3.25216400	2.48811900	0.52340500
H	-3.40076100	2.92115500	-1.17462700
H	-3.57752000	0.55880500	-1.83106000
H	-4.85140200	0.95666500	-0.66814400
H	-1.56452900	-0.77143300	-0.99567800
H	-1.46334700	-1.44428100	1.97838900
H	-1.60504000	-2.54017300	0.61177500
H	0.78738400	-2.57041900	1.24232200
H	0.41532300	0.35504000	-1.40529900
H	1.70312300	1.88934700	0.89901300
H	1.13595400	2.60966100	-0.61698400
H	3.60109300	2.11921700	-0.62164900
H	2.75438800	1.28363100	-1.91546400
H	2.93291900	-1.87222100	0.54373700
H	5.19142100	0.24468800	-1.40996400
H	6.72206500	0.48868300	0.52547800
H	5.38607800	0.11940800	1.64797500
H	5.35773500	1.61581100	0.68839200
H	6.40493900	-1.75114500	-0.67505100
H	5.13797900	-2.16851000	0.49121300
H	4.79011300	-2.23799300	-1.25340500
H	-4.96848400	-0.28805500	1.51324700
H	-3.70055500	0.86255500	1.93989500
H	-3.46660900	-0.86650000	2.25685200
H	-0.27986100	0.63121300	2.29387600
H	-0.33140700	2.31132900	1.72836100
H	-1.83284000	1.46675300	2.11310800
H	-3.63761300	-1.47227900	-2.31303300
H	-5.02710800	-1.48840800	-0.46442700
H	-3.76958500	-2.45398300	0.32064200

VI

C	-1.39673900	1.95381200	-0.66053700
C	-2.89309000	2.21583800	-0.54377900
C	-3.68478300	0.95479500	-0.86424400
C	-3.32917600	-0.23430100	0.04938500
C	-1.78128500	-0.44432900	0.04004100
C	-1.31809000	-1.60078800	0.93085500
C	0.16778000	-1.78142400	0.91377400
C	1.02454200	-0.88125700	0.40629600
C	0.54523400	0.42240900	-0.20616000
C	-0.89012500	0.81799800	0.25109000
C	1.61288000	1.50417500	-0.00075000
C	2.95332600	1.07678300	-0.59089200
C	3.38467200	-0.27957000	-0.08830900
C	2.46718500	-1.14230200	0.38185000
C	4.85867400	-0.62670900	-0.12028400
C	5.64974700	0.16688600	0.92786000
C	5.45634500	-0.44303800	-1.51960900
C	-4.00539200	-1.49168000	-0.52914900
C	-3.95904600	-0.02511000	1.43600600
C	-0.85720200	1.28748800	1.71479200
O	-3.42922600	-1.84288200	-1.76079600
H	-1.17476200	1.68418100	-1.70866300
H	-0.83719400	2.87927000	-0.45143400
H	-3.18259800	3.02350500	-1.23464100
H	-3.13909600	2.58218800	0.46696100
H	-4.76883200	1.15018400	-0.79558700
H	-3.48230100	0.65893000	-1.90673700
H	-1.57168500	-0.76087400	-0.99530800
H	-1.78930900	-2.53896900	0.59488300
H	-1.65123100	-1.46160600	1.97583700
H	0.56580600	-2.70860100	1.34100400
H	0.48623000	0.24757100	-1.29839600
H	1.75047400	1.69070000	1.07679700
H	1.29296100	2.45334700	-0.45538100
H	3.72310600	1.83028800	-0.35664800
H	2.88871500	1.05318700	-1.69454000
H	2.79322800	-2.11969300	0.75502900
H	4.94366400	-1.69456500	0.14276900
H	6.70736400	-0.14009400	0.93201900
H	5.62279400	1.24891000	0.72194900
H	5.24117800	0.00440200	1.93617700
H	6.50508800	-0.77735400	-1.54227600
H	5.44160600	0.61479700	-1.82755700
H	4.89700400	-1.02182400	-2.27013900
H	-5.05643400	-0.09660900	1.36077400
H	-3.73646900	0.96322700	1.85626600
H	-3.63069200	-0.77961300	2.16523000
H	-0.37137900	0.54599000	2.36632400
H	-0.29431200	2.22797500	1.80865100
H	-1.86030400	1.47488900	2.11497000
H	-3.89919200	-2.59916000	-2.11833700
H	-5.08519400	-1.26843900	-0.64421600
H	-3.92716900	-2.32112000	0.20116000

VII

C	-1.53417700	1.95297100	-0.70438200
C	-3.03747000	2.12540300	-0.52639700
C	-3.76188600	0.81265400	-0.79343800
C	-3.30019000	-0.33529900	0.12231900
C	-1.74553200	-0.44743900	0.05860100
C	-1.17321000	-1.54905500	0.95640300
C	0.31638000	-1.65811400	0.85302000
C	1.09472200	-0.72125500	0.28774200
C	0.51439700	0.54705500	-0.31172200
C	-0.91995300	0.86824600	0.20331300
C	1.52695700	1.68894800	-0.16186600
C	2.85989800	1.32841400	-0.80802900
C	3.39016300	-0.00044300	-0.32806300
C	2.54629500	-0.91030700	0.18851200
C	4.88668900	-0.21135100	-0.44925700
C	5.62978200	0.54191200	0.66472900
C	5.31692600	-1.67646100	-0.48594900
C	-3.92364200	-1.64680200	-0.42010100
C	-3.88720000	-0.14879500	1.52922100
C	-0.85150000	1.36714600	1.65581000
O	-3.47655900	-2.03822400	-1.69048200
H	-1.02297800	2.91384600	-0.53594300
H	-1.34156600	1.67729000	-1.75684200
H	-3.26252100	2.49292500	0.48882000
H	-3.40445200	2.90210500	-1.21574500
H	-3.58408800	0.50754200	-1.83833400
H	-4.85217900	0.94246100	-0.68587000
H	-1.54813000	-0.75702300	-0.98424700
H	-1.45247500	-1.37920300	2.01196300
H	-1.62155200	-2.52479100	0.70127500
H	0.78525000	-2.55622400	1.26988100
H	0.41957600	0.35864200	-1.39943400
H	1.70140400	1.89243000	0.90721600
H	1.13421100	2.61441700	-0.60799600
H	3.60093100	2.12079800	-0.61239700
H	2.75243600	1.29113100	-1.90855600
H	2.92853300	-1.87087300	0.54539400
H	5.18495900	0.24764700	-1.41052500
H	6.71981000	0.47842200	0.52296600
H	5.38552700	0.10641000	1.64643400
H	5.35850000	1.60771000	0.69442700
H	6.39567700	-1.75449900	-0.68938300
H	4.77855000	-2.23536300	-1.26556500
H	5.13169100	-2.17520600	0.47859600
H	-4.98106300	-0.27917300	1.49330500
H	-3.70250100	0.85230100	1.93856200
H	-3.49560700	-0.88181900	2.24943900
H	-0.28290900	0.67595000	2.29564800
H	-0.35325600	2.34649000	1.70542100
H	-1.84585200	1.49111200	2.10019200
H	-2.59793700	-2.41768400	-1.60560700
H	-3.78993300	-2.45842200	0.32099100
H	-5.01028300	-1.48812000	-0.51302500

VIII

C	-1.51790700	2.00972000	-0.49016000
C	-3.03187900	2.14654800	-0.38716500
C	-3.71822000	0.86110900	-0.83011700
C	-3.28685200	-0.37084100	-0.01076200
C	-1.72741300	-0.46138500	-0.00194300
C	-1.19107000	-1.65153600	0.79868600
C	0.30444900	-1.71533900	0.80451200
C	1.09791900	-0.71298200	0.39482100
C	0.52876500	0.59678800	-0.11823700
C	-0.94067600	0.84216100	0.33547600
C	1.50720400	1.73258900	0.20341100
C	2.88195600	1.45347600	-0.39512500
C	3.41501100	0.09895400	0.01025700
C	2.55731300	-0.86415400	0.39117900
C	4.91933200	-0.07986000	-0.04377700
C	5.43793700	0.11150000	-1.47708100
C	5.42337900	-1.39794200	0.53752500
C	-3.85418800	-1.62226600	-0.70694800
C	-3.95357200	-0.32769500	1.37382300
C	-0.97040700	1.18692700	1.83348200
O	-3.23175700	-1.82379100	-1.94961400
H	-1.03585000	2.95395700	-0.19128700
H	-1.25765000	1.84781200	-1.55155000
H	-3.32223200	2.40569400	0.64474600
H	-3.37140000	2.98591600	-1.01454600
H	-4.81511400	0.96740400	-0.77034300
H	-3.47616000	0.67104800	-1.88871100
H	-1.47670600	-0.67118300	-1.05517200
H	-1.58070400	-2.59048700	0.37242500
H	-1.55359600	-1.62955700	1.84295200
H	0.76534700	-2.64188900	1.16470600
H	0.50287600	0.51239200	-1.22243200
H	1.61394600	1.83444200	1.29565200
H	1.12521900	2.69180800	-0.17592300
H	3.59604400	2.23671800	-0.08830000
H	2.82585700	1.51600300	-1.49761100
H	2.93338200	-1.84137300	0.70537300
H	5.34809000	0.73787900	0.56713800
H	6.53522700	0.02912500	-1.50939400
H	5.01762800	-0.65927200	-2.14294400
H	5.16693500	1.09560400	-1.88655100
H	6.52351800	-1.42199900	0.53057400
H	5.07003900	-2.25790600	-0.05360700
H	5.08928300	-1.53835100	1.57616800
H	-5.04022800	-0.47983700	1.26861100
H	-3.81852200	0.63727800	1.87740400
H	-3.57633100	-1.11112900	2.04671000
H	-0.44415400	0.42970600	2.43346300
H	-0.47995700	2.15392900	2.01927500
H	-1.99209200	1.26789600	2.22211800
H	-3.63447100	-2.58172900	-2.37859900
H	-4.94587200	-1.47216700	-0.82843100
H	-3.72477100	-2.50204200	-0.04613600

IX

C	-1.53010300	1.95448200	-0.69339100
C	-3.03165900	2.13280500	-0.50581400
C	-3.76505500	0.82546500	-0.77540500
C	-3.29848500	-0.33069800	0.13047300
C	-1.74337800	-0.45284300	0.04772000
C	-1.17278900	-1.56970500	0.92636000
C	0.31825400	-1.66705700	0.83730500
C	1.09780100	-0.72685000	0.28007500
C	0.51573800	0.53994900	-0.32039600
C	-0.91615900	0.86003500	0.20280800
C	1.52867400	1.68274800	-0.17871000
C	2.86167100	1.31888900	-0.82324500
C	3.39431000	-0.00461700	-0.33093200
C	2.55029300	-0.91231300	0.18898300
C	4.89188300	-0.21250300	-0.44548700
C	5.63003800	0.55291800	0.66345000
C	5.32606800	-1.67675300	-0.46714100
C	-3.92863000	-1.63110000	-0.40317500
C	-3.87138000	-0.14148900	1.54432500
C	-0.83903700	1.34850800	1.65862100
O	-3.39400700	-1.96276300	-1.65869300
H	-1.34402500	1.68549400	-1.74863500
H	-1.01524000	2.91271000	-0.52028300
H	-3.24879000	2.49687100	0.51232600
H	-3.39984100	2.91452100	-1.18904300
H	-3.59686600	0.52906400	-1.82382100
H	-4.85382800	0.96038700	-0.65538000
H	-1.56604600	-0.77010400	-0.99342600
H	-1.46171200	-1.43546900	1.98506700
H	-1.60625800	-2.53709800	0.62408700
H	0.78788900	-2.56552500	1.25303800
H	0.41371500	0.34835700	-1.40662000
H	1.70473700	1.89341100	0.88875800
H	1.13581500	2.60554400	-0.63042500
H	3.60132800	2.11467300	-0.63554000
H	2.75273500	1.27144100	-1.92327400
H	2.93303100	-1.86996400	0.55332400
H	5.19225600	0.23796600	-1.41021100
H	6.72081300	0.49168900	0.52563200
H	5.38403000	0.12568000	1.64837500
H	5.35505900	1.61803400	0.68268700
H	6.40585500	-1.75421200	-0.66577400
H	5.13808800	-2.16709000	0.50120400
H	4.79154700	-2.24397200	-1.24339300
H	-4.96530900	-0.27536600	1.52368100
H	-3.68493600	0.86240900	1.94484000
H	-3.46562300	-0.86819500	2.26273800
H	-0.28249100	0.64264200	2.29292600
H	-0.32470400	2.31925600	1.71590300
H	-1.83057400	1.48631100	2.10511400
H	-3.83593100	-2.74911800	-1.98582600
H	-5.02384100	-1.47121700	-0.46660600
H	-3.76716600	-2.44626600	0.32955600

X

C	-1.28743700	2.13209100	-0.43349500
C	-2.77953900	2.41038900	-0.31203900
C	-3.58330500	1.25302900	-0.88778300
C	-3.29796800	-0.09951000	-0.20516400
C	-1.75355400	-0.34689100	-0.17106100
C	-1.34950200	-1.63432400	0.55530300
C	0.12909900	-1.86058600	0.53177300
C	1.02467600	-0.91741800	0.19885900
C	0.60043100	0.48793300	-0.18864000
C	-0.84223100	0.84083300	0.27982400
C	1.68476400	1.48144100	0.24739000
C	3.03465600	1.12990500	-0.37114700
C	3.41323300	-0.31040000	-0.12412600
C	2.45957500	-1.21573600	0.15543000
C	4.87744700	-0.69299300	-0.18872800
C	5.66156100	-0.13672300	1.00704100
C	5.51841400	-0.26973100	-1.51504900
C	-3.98116500	-1.16290400	-1.10169600
C	-3.99222000	-0.13437200	1.16586700
C	-0.85329200	1.05290400	1.80311100
O	-4.06026100	-2.46349900	-0.57902400
H	-0.71302300	2.98976200	-0.04990400
H	-1.03768400	2.04521900	-1.50648900
H	-3.03106300	3.34026400	-0.84598100
H	-3.05311900	2.58380200	0.74193300
H	-4.66404600	1.46714700	-0.83279800
H	-3.34047000	1.16333700	-1.96262400
H	-1.48777400	-0.49850100	-1.23461000
H	-1.69184800	-1.61019900	1.60671000
H	-1.87195700	-2.49044400	0.10622900
H	0.48855100	-2.85966700	0.80190100
H	0.58458200	0.51429700	-1.29626000
H	1.78712900	1.46047900	1.34463700
H	1.40531000	2.50804500	-0.03217600
H	3.81424300	1.79998500	0.02696500
H	3.01028300	1.31948100	-1.46029500
H	2.74804100	-2.25534500	0.34707500
H	4.92519300	-1.79340200	-0.12994500
H	6.70953400	-0.47370800	0.97614900
H	5.67104300	0.96503200	1.00823000
H	5.22115800	-0.47215400	1.95759700
H	6.55719400	-0.62900100	-1.57786700
H	5.54302400	0.82704100	-1.61801100
H	4.96344100	-0.67804000	-2.37321800
H	-5.08705700	-0.10308800	1.03140700
H	-3.73027700	0.72422300	1.79655100
H	-3.74663000	-1.04086200	1.73805500
H	-0.38322600	0.21136000	2.33265900
H	-0.29569900	1.96263700	2.07055500
H	-1.86775700	1.17172600	2.20184900
H	-4.62769700	-2.44837800	0.19649600
H	-4.99311900	-0.78721100	-1.35716300
H	-3.42604500	-1.24745900	-2.04947400

XI

C	-1.28847700	2.01658300	-0.39901700
C	-2.78344100	2.26370500	-0.24844100
C	-3.57710500	1.07519800	-0.77463500
C	-3.23666700	-0.25029700	-0.06444200
C	-1.68966500	-0.46324400	-0.09737700
C	-1.23156700	-1.74260500	0.61217700
C	0.25292100	-1.92873400	0.56284000
C	1.11582100	-0.96335200	0.20914000
C	0.64615000	0.42674900	-0.17905700
C	-0.79400800	0.74556500	0.31957700
C	1.71228100	1.45257400	0.22551900
C	3.05897800	1.13083600	-0.41532900
C	3.48074300	-0.29600200	-0.16137000
C	2.55727500	-1.22408100	0.14369500
C	4.95342000	-0.63926100	-0.24808100
C	5.55951300	-0.21097700	-1.58909600
C	5.74159700	-0.04999000	0.92906500
C	-3.88264300	-1.40204600	-0.87625800
C	-3.88524000	-0.26243700	1.32898000
C	-0.78005900	0.96877000	1.84092700
O	-5.26565500	-1.26031900	-1.06882800
H	-1.05975800	1.92103900	-1.47607600
H	-0.72613600	2.89246100	-0.03949700
H	-3.06774900	3.17551900	-0.79700800
H	-3.03613500	2.45460700	0.80731100
H	-3.36070600	0.96689700	-1.85504900
H	-4.65773800	1.27332800	-0.68151000
H	-1.45884000	-0.60991600	-1.17057700
H	-1.56204400	-1.74471000	1.66657100
H	-1.71198200	-2.62420200	0.15864400
H	0.64363900	-2.91575000	0.83359200
H	0.60530800	0.44259600	-1.28627800
H	1.83550800	1.44588500	1.32073700
H	1.39987500	2.46822500	-0.05872100
H	3.82780300	1.82538500	-0.03908500
H	3.00915400	1.30762600	-1.50575700
H	2.87709100	-2.25361200	0.33973900
H	5.03249500	-1.73732600	-0.18011100
H	6.60653500	-0.54227300	-1.66646400
H	5.55219100	0.88502700	-1.70225000
H	5.00165800	-0.64251400	-2.43395600
H	6.79774600	-0.35839200	0.88327900
H	5.72063200	1.05155500	0.91984500
H	5.32682400	-0.38819200	1.89011200
H	-4.97267000	-0.37141100	1.20557700
H	-3.70731700	0.66099000	1.89539400
H	-3.53629600	-1.10529500	1.94248700
H	-0.27649000	0.14332400	2.36550800
H	-0.24220500	1.89495800	2.09173800
H	-1.78994300	1.05961000	2.25701800
H	-5.41670700	-0.51999400	-1.66235900
H	-3.35138700	-1.50029000	-1.84468700
H	-3.75608200	-2.35431700	-0.34043700

XII

C	-1.41182500	2.01005800	-0.54246500
C	-2.91274500	2.17998800	-0.35027300
C	-3.65225400	0.91712700	-0.77181100
C	-3.20920400	-0.33858400	0.00540600
C	-1.65439800	-0.46463500	-0.07537600
C	-1.09573500	-1.66869800	0.69142900
C	0.39463700	-1.77170200	0.59600000
C	1.18625500	-0.78275200	0.15179800
C	0.62030400	0.55043000	-0.30174500
C	-0.81776600	0.81698900	0.23235400
C	1.63624500	1.66012000	-0.00391700
C	2.97671200	1.37030800	-0.67008600
C	3.49342300	-0.01022200	-0.34613000
C	2.63777600	-0.96898900	0.04803400
C	4.99008500	-0.21593400	-0.47474600
C	5.72490000	0.39555600	0.72798600
C	5.41198300	-1.66973300	-0.67857200
C	-3.81393900	-1.57547000	-0.70666500
C	-3.80598000	-0.29737200	1.42124400
C	-0.76414000	1.13590600	1.73577700
O	-5.20889800	-1.52604500	-0.85351800
H	-0.89078800	2.93759000	-0.25821500
H	-1.21504800	1.86062600	-1.61968700
H	-3.13954600	2.42266800	0.70068600
H	-3.26959800	3.03750300	-0.94227000
H	-4.73881400	1.05911400	-0.64983900
H	-3.46735500	0.75262400	-1.85093700
H	-1.45396900	-0.66481300	-1.14602500
H	-1.53782800	-2.60296800	0.31011900
H	-1.38844600	-1.62485600	1.75601000
H	0.85228800	-2.71598800	0.91075400
H	0.53899900	0.49328800	-1.40534400
H	1.79597500	1.73410300	1.08410600
H	1.25551900	2.63500800	-0.34232500
H	3.71852600	2.12855500	-0.36977300
H	2.88530600	1.46684300	-1.76850000
H	3.00970700	-1.96809900	0.29220200
H	5.30245900	0.35054200	-1.37217100
H	6.81613200	0.34045700	0.59186800
H	5.46606500	-0.15008500	1.64905000
H	5.46041000	1.45266100	0.87956000
H	6.49258600	-1.73134500	-0.87787300
H	4.87932400	-2.12941800	-1.52413800
H	5.21222300	-2.27670700	0.21869200
H	-4.88841100	-0.47777600	1.34781700
H	-3.66504200	0.67018500	1.92033600
H	-3.38479200	-1.07610900	2.07294300
H	-0.18964200	0.37860800	2.28937200
H	-0.27956700	2.10812500	1.90895400
H	-1.76239700	1.18872100	2.18513000
H	-5.42484700	-0.83705600	-1.48743100
H	-3.31247000	-1.70600600	-1.68703700
H	-3.61178500	-2.48197400	-0.11734600

Molecule 3

I

C	-0.51376500	-2.12047300	1.97833800
C	-1.90556400	-2.69043400	2.22221800
C	-2.95285200	-1.84303800	1.51401400
C	-2.73591200	-1.74804600	-0.00885200
C	-1.26197800	-1.33237000	-0.30740300
C	-0.93038100	-1.28225700	-1.80278900
C	0.48692900	-0.87957100	-2.06310900
C	1.43381100	-0.81740500	-1.11358700
C	1.13450800	-1.16072000	0.33383600
C	-0.12979600	-2.05706800	0.48767600
C	2.40334000	-1.69426600	1.00850300
C	3.54914400	-0.69239600	0.89259700
C	3.78889200	-0.27391300	-0.53832900
C	2.79079300	-0.36748300	-1.43474000
C	5.16610000	0.21697900	-0.93490200
C	6.20994600	-0.90532500	-0.86490000
C	5.60310600	1.43007700	-0.10690400
C	-3.69178400	-0.66965600	-0.55157500
C	-3.18538600	-3.05287700	-0.68616900
C	0.16621200	-3.47160900	-0.03652200
O	-3.50499300	0.59522400	0.09059300
H	-0.47959200	-1.09629300	2.39271600
H	0.23505400	-2.70789700	2.53238300
H	-1.95662800	-3.73877100	1.88428700
H	-2.11508300	-2.71151700	3.30320100
H	-2.91741000	-0.82438900	1.93332700
H	-3.96805600	-2.22714500	1.71080300
H	-1.20206700	-0.29547000	0.05970600
H	-1.59377100	-0.56713300	-2.31500300
H	-1.11859500	-2.25955100	-2.28313200
H	0.75034600	-0.61806800	-3.09400400
H	0.89783600	-0.20194900	0.83537500
H	2.71486600	-2.63783000	0.53157200
H	2.21193500	-1.91479300	2.06881200
H	4.46814600	-1.12228300	1.32218700
H	3.32591700	0.20093800	1.50374500
H	2.97634500	-0.07843200	-2.47552500
H	5.10161900	0.53834300	-1.98810000
H	7.18250000	-0.55399000	-1.24295000
H	5.89975500	-1.77027400	-1.46958400
H	6.36646300	-1.25238500	0.16874300
H	6.59894900	1.77938800	-0.42009400
H	5.66072800	1.18773500	0.96652400
H	4.90035600	2.26817300	-0.23033300
H	-3.05660700	-3.02094800	-1.77774100
H	-2.63749000	-3.92603800	-0.31265500
H	-4.25329800	-3.23405100	-0.48604800
H	-0.72202300	-4.11449700	-0.02730200
H	0.92170400	-3.96524300	0.59241500
H	0.55480500	-3.45265400	-1.06495400
H	-4.73135100	-0.96116400	-0.33710000
H	-3.59199800	-0.54342500	-1.63890200
S	-3.05716000	1.93034700	-0.70815300
O	-2.89209800	1.60501500	-2.11485800
O	-3.96654500	2.96618800	-0.28141300
C	-1.47008800	2.23350200	0.01947000

C	-0.33009400	2.20513300	-0.77348600
C	-1.39521800	2.48142700	1.39167200
C	0.91173800	2.42084600	-0.17790900
H	-0.41291700	1.99469200	-1.84032700
C	-0.14947500	2.68230800	1.96866500
H	-2.30400900	2.50922000	1.99535500
C	1.02215700	2.65594200	1.19482600
H	1.81344100	2.37278500	-0.79236600
H	-0.08019900	2.86634000	3.04374300
C	2.36095800	2.89443800	1.83829700
H	2.49409500	3.96220400	2.07463300
H	2.45449400	2.33670200	2.78195100
H	3.18336100	2.59222600	1.17689700

II

C	-0.69013200	-2.42129900	1.77928000
C	-2.10860000	-2.97025400	1.86356100
C	-3.08922900	-1.98682500	1.24142200
C	-2.78613700	-1.67249900	-0.23678400
C	-1.28687100	-1.27178400	-0.39582800
C	-0.86996600	-1.01326300	-1.84802300
C	0.56932700	-0.62012100	-1.96856700
C	1.45886800	-0.72775700	-0.96950100
C	1.07102000	-1.28477300	0.38630200
C	-0.22300300	-2.14775000	0.33677200
C	2.28714900	-1.96651600	1.02279500
C	3.45349100	-0.99279900	1.14380900
C	3.76596100	-0.30151300	-0.16034600
C	2.82963400	-0.23190700	-1.12302700
C	5.16540100	0.26484500	-0.30280200
C	6.17283000	-0.85103300	-0.61838000
C	5.28566700	1.40011700	-1.31780600
C	-3.67719100	-0.48915100	-0.65399100
C	-3.23853900	-2.84325000	-1.12436800
C	0.06236900	-3.47717400	-0.38081200
O	-3.45483600	0.66420800	0.16319400
H	-0.65004800	-1.47352800	2.34646300
H	0.01050900	-3.11189600	2.27412500
H	-2.17124300	-3.95257800	1.36654700
H	-2.37792700	-3.14704900	2.91666200
H	-3.04728500	-1.04494900	1.81234200
H	-4.12470600	-2.35762400	1.32585200
H	-1.21630600	-0.30250200	0.12356400
H	-1.48702100	-0.20915900	-2.28199800
H	-1.05688400	-1.90105100	-2.47881600
H	0.89480200	-0.20131000	-2.92739500
H	0.83721400	-0.40692400	1.02106600
H	2.60498000	-2.81970100	0.40151500
H	2.03233500	-2.36541700	2.01546400
H	4.35018200	-1.51820100	1.51188800
H	3.22789200	-0.22524200	1.90774500
H	3.05940200	0.25337700	-2.07631800
H	5.43345600	0.67526400	0.68953200
H	7.20314500	-0.46298900	-0.61913200
H	5.96624100	-1.27937500	-1.61166800
H	6.12384500	-1.66883700	0.11571200
H	6.29498600	1.83740600	-1.28846400
H	5.11585700	1.04273000	-2.34552300

H	4.56039100	2.20316800	-1.11600900
H	-3.04353200	-2.65771500	-2.19043400
H	-2.74526100	-3.78472600	-0.85478100
H	-4.32229900	-3.00513900	-1.01189800
H	-0.84421400	-4.07756700	-0.52286600
H	0.76089900	-4.09032200	0.20758100
H	0.51632000	-3.31814000	-1.36958900
H	-4.73500100	-0.76034700	-0.51377600
H	-3.53453600	-0.21924500	-1.70988500
S	-3.00469900	2.09361000	-0.44824100
O	-2.93983400	1.98541600	-1.89612800
O	-3.84776800	3.07220200	0.19447100
C	-1.36146400	2.24874800	0.19776400
C	-0.29113300	2.36265300	-0.68126300
C	-1.16904700	2.26382900	1.58009000
C	0.99564000	2.48752200	-0.16512100
H	-0.46803700	2.33916300	-1.75721300
C	0.12395500	2.37307800	2.07588300
H	-2.02270700	2.18412000	2.25540900
C	1.22520500	2.48885500	1.21339300
H	1.84215800	2.55962200	-0.85102800
H	0.28456300	2.37249300	3.15679900
C	2.62142700	2.63990200	1.75283100
H	2.89443900	3.70492100	1.82624300
H	2.71670300	2.20421200	2.75743200
H	3.35159400	2.15341800	1.09071000

III

C	-0.71686400	-2.18499200	1.95478000
C	-2.15461200	-2.65895700	2.12747500
C	-3.10908400	-1.72137000	1.40177300
C	-2.82222600	-1.60452200	-0.10794400
C	-1.31171300	-1.28592100	-0.33449400
C	-0.91475100	-1.21828500	-1.81310700
C	0.53719100	-0.91215400	-2.00438000
C	1.44591100	-0.94427600	-1.01677300
C	1.06255100	-1.30530400	0.40549900
C	-0.26788400	-2.11104400	0.48282800
C	2.26199200	-1.94968900	1.10902500
C	3.47600300	-1.02619000	1.06825100
C	3.80892500	-0.58716800	-0.33903100
C	2.84402500	-0.58716500	-1.27620700
C	5.24752800	-0.19077000	-0.60668800
C	5.57964100	0.04176700	-2.07801000
C	5.64965500	1.02983100	0.23359200
C	-3.67762400	-0.44922000	-0.65978900
C	-3.33180900	-2.85766800	-0.83825200
C	-0.05217000	-3.52895500	-0.07049300
O	-3.42971000	0.78411200	0.02151900
H	-0.62724900	-1.17688900	2.39912900
H	-0.03499600	-2.83868800	2.52099500
H	-2.26589800	-3.69234100	1.75926300
H	-2.40974200	-2.69139700	3.19833300
H	-3.01974900	-0.71850100	1.85012500
H	-4.15611400	-2.03685000	1.54708700
H	-1.19507500	-0.26635200	0.06612400
H	-1.50400100	-0.44355600	-2.32933400
H	-1.15129600	-2.16579400	-2.33041100

H	0.86115700	-0.64322900	-3.01598300
H	0.87570500	-0.34652600	0.92778700
H	2.52521700	-2.89774100	0.61246200
H	2.01417500	-2.18837000	2.15361800
H	4.35099200	-1.52857200	1.51414700
H	3.28574400	-0.13984700	1.70046200
H	3.07629100	-0.29567500	-2.30408400
H	5.86589100	-1.03959900	-0.25707600
H	6.65296800	0.25180000	-2.19840300
H	5.02753800	0.90514600	-2.48272900
H	5.33627000	-0.83665100	-2.69349800
H	6.72047600	1.25331200	0.11212300
H	5.08317300	1.91920700	-0.08782300
H	5.46226500	0.87495900	1.30648700
H	-3.14975800	-2.81091300	-1.92167000
H	-2.86663000	-3.77667100	-0.46263600
H	-4.41822700	-2.96474700	-0.69144300
H	-0.98356500	-4.10582500	-0.11465800
H	0.63993500	-4.09248000	0.57246000
H	0.37837700	-3.51061400	-1.08210600
H	-4.74289600	-0.67174200	-0.49429000
H	-3.52432400	-0.30642100	-1.73875200
S	-2.86376200	2.10469700	-0.72507500
O	-2.66770600	1.80298900	-2.13305600
O	-3.71744000	3.18917800	-0.30425600
C	-1.28889200	2.28089200	0.06760500
C	-1.25049600	2.49449700	1.44716700
C	-0.12373400	2.18729700	-0.68237900
C	-0.01729900	2.59340000	2.07510800
H	-2.17807800	2.57521000	2.01652700
C	1.10603200	2.30070300	-0.03555500
H	-0.17947900	2.00584600	-1.75629000
C	1.17894500	2.49854500	1.34540700
H	0.02282900	2.75002700	3.15600200
H	2.02559600	2.20082700	-0.61638000
C	2.50523200	2.62505400	2.04402800
H	2.52815200	2.02507200	2.96603100
H	2.69584800	3.67164400	2.33030900
H	3.33068400	2.29754200	1.39902100

Molecule 4

I

C	-0.20072700	2.03965800	-1.53763600
C	-1.58255800	2.68175000	-1.54582400
C	-2.62959200	1.70094400	-1.03417300
C	-2.33948300	1.19925000	0.39257300
C	-0.87912700	0.65538700	0.46899400
C	-0.48315600	0.15306400	1.85993500
C	0.91554900	-0.37948600	1.89678700
C	1.81410800	-0.18663200	0.91836400
C	1.47882200	0.60513100	-0.33287100
C	0.24775100	1.54195800	-0.14871500
C	2.74576000	1.29359000	-0.85509400
C	3.85203400	0.27855400	-1.12618000
C	4.11101900	-0.60729600	0.06813500
C	3.15387700	-0.77712900	0.99669700
C	5.46297000	-1.27929600	0.18905200
C	6.56937000	-0.26945300	0.52091500
C	5.81019700	-2.08776800	-1.06594200
C	-3.35875000	0.10301800	0.76407100
C	-2.65565000	2.32808400	1.39715200
C	0.63564300	2.73402700	0.74151900
H	0.54265600	2.74609500	-1.93881200
H	-0.21724700	1.17800300	-2.22883500
H	-1.83957600	2.99521700	-2.56967300
H	-1.58206400	3.60322500	-0.94033800
H	-3.63572900	2.15251900	-1.06232800
H	-2.65726000	0.83432900	-1.71651300
H	-0.89338800	-0.23720200	-0.18087700
H	-0.58708100	0.95149100	2.61729400
H	-1.17035900	-0.64771700	2.18054500
H	1.20649300	-0.96309000	2.77683700
H	1.19174200	-0.13882700	-1.10182400
H	3.11178400	2.01632900	-0.10787000
H	2.52746700	1.85886100	-1.77317000
H	4.77707200	0.80233100	-1.41747100
H	3.58199400	-0.35027300	-1.99465300
H	3.35717500	-1.41341200	1.86520600
H	5.39710700	-1.98472200	1.03439200
H	7.53320200	-0.78215100	0.66516800
H	6.70590700	0.46360100	-0.29020600
H	6.33445700	0.28467400	1.44168100
H	6.75553500	-2.63466600	-0.92728900
H	5.93428100	-1.43406200	-1.94418500
H	5.02258200	-2.82060600	-1.29767700
H	-3.73345300	2.55385000	1.38433200
H	-2.13376000	3.25977000	1.14919200
H	-2.38669100	2.05711500	2.42773500
H	1.07980200	2.40290800	1.69174900
H	1.37786800	3.36772400	0.23453600
H	-0.21930600	3.37713800	0.97969400
H	-4.37946700	0.46513600	0.58529500
H	-3.27866500	-0.18811700	1.81842200
I	-3.27795100	-1.77621900	-0.31635700

II

C	-0.32773500	2.19977200	-1.43708800
C	-1.74643400	2.75279000	-1.37770800
C	-2.71885200	1.67910100	-0.90683200
C	-2.36405400	1.10997000	0.47935100
C	-0.87188800	0.65425700	0.49084400
C	-0.41246100	0.09142100	1.83837000
C	1.01771200	-0.35029400	1.81461400
C	1.87920600	-0.04183500	0.83238700
C	1.46462700	0.80165900	-0.35903300
C	0.18337500	1.64641300	-0.09150900
C	2.67559100	1.59659100	-0.86128600
C	3.82733200	0.66315700	-1.21941800
C	4.17752500	-0.27045600	-0.08419900
C	3.25578700	-0.54880400	0.85450300
C	5.58151000	-0.84180500	-0.08700300
C	5.94545400	-1.63725700	1.16358000
C	5.82312300	-1.68153000	-1.35054600
C	-3.30577300	-0.06811000	0.80215400
C	-2.72368400	2.15285700	1.55940300
C	0.51824600	2.80290900	0.86448200
H	-0.30735900	1.38303100	-2.18071200
H	0.36085000	2.97513400	-1.80766300
H	-2.04654600	3.11074400	-2.37490500
H	-1.78813000	3.63440000	-0.71687700
H	-3.75102300	2.06796400	-0.88608200
H	-2.71031900	0.85565700	-1.64116400
H	-0.84795000	-0.19491400	-0.21459500
H	-1.03946100	-0.77114800	2.11960300
H	-0.54940000	0.83181100	2.64765600
H	1.36547000	-0.96500000	2.65186600
H	1.20490900	0.09022200	-1.16747200
H	3.01793800	2.29104200	-0.07701900
H	2.40402000	2.20412900	-1.73715000
H	4.71802500	1.25012200	-1.50113800
H	3.56211000	0.07392300	-2.11653200
H	3.50483700	-1.20751800	1.69045300
H	6.26625600	0.02653700	-0.13956200
H	6.99794700	-1.95527500	1.12019200
H	5.32896800	-2.54582700	1.25419200
H	5.81056200	-1.04131600	2.07833500
H	6.85734800	-2.05754500	-1.37683500
H	5.65763300	-1.10175600	-2.27035300
H	5.14372100	-2.54874600	-1.37029300
H	-3.81332000	2.31063900	1.58362800
H	-2.26653600	3.12916200	1.36011300
H	-2.41289900	1.83636700	2.56494500
H	0.99746400	2.44119800	1.78610300
H	1.21377600	3.50817500	0.38669300
H	-0.36773900	3.37973500	1.15343300
H	-4.35049100	0.24028300	0.66641300
H	-3.18342900	-0.41700600	1.83470900
I	-3.13677100	-1.86979400	-0.39378100

III

C	0.26070100	2.20032100	-0.73893700
C	-1.17725900	2.66646900	-0.56089300
C	-2.13757500	1.49402000	-0.69839900
C	-1.89111600	0.37404600	0.33108500
C	-0.37069200	-0.03363100	0.28656900
C	0.01746700	-1.09158400	1.32611100
C	1.45920400	-1.48429400	1.22948100
C	2.37679100	-0.79014500	0.53802300
C	2.01892400	0.47867900	-0.21436900
C	0.68608400	1.12070000	0.27406400
C	3.22820400	1.42209000	-0.22142100
C	4.44965700	0.74601200	-0.83685700
C	4.74258500	-0.58767700	-0.19376800
C	3.76534400	-1.25198300	0.44733500
C	6.15091400	-1.13794000	-0.28194800
C	6.64570300	-1.20552500	-1.73094800
C	7.12408500	-0.34390300	0.59930200
C	-2.65566500	-0.89374700	-0.09840500
C	-2.38559400	0.81190300	1.71718700
C	0.89806100	1.75682900	1.65821800
H	0.94675700	3.05934700	-0.67780400
H	0.36739900	1.78864600	-1.75888700
H	-1.30545100	3.15960500	0.41674400
H	-1.41811700	3.42980700	-1.31703100
H	-2.02892600	1.07087300	-1.71351900
H	-3.18041600	1.83761100	-0.61861200
H	-0.24815200	-0.51287400	-0.70289600
H	-0.19442600	-0.73021900	2.34854400
H	-0.60010200	-1.99583600	1.20617100
H	1.76746800	-2.39341200	1.75697300
H	1.85465300	0.18024600	-1.26869200
H	3.47598500	1.71663000	0.81122100
H	2.99616600	2.34386000	-0.77481500
H	5.32633800	1.40850200	-0.75133200
H	4.29363200	0.60093200	-1.92186700
H	3.99289100	-2.21278800	0.92224800
H	6.11875700	-2.16859400	0.10955700
H	7.63559900	-1.68447700	-1.78186100
H	6.74422000	-0.19981100	-2.17006800
H	5.95384800	-1.78430900	-2.36142300
H	8.12894900	-0.79333200	0.57097200
H	6.78514700	-0.32737700	1.64565200
H	7.22146000	0.69911500	0.25800000
H	-3.47042100	0.99231400	1.68807200
H	-1.91406900	1.74213000	2.05472800
H	-2.20088100	0.04441400	2.48261100
H	1.34585200	1.04746000	2.36930800
H	1.57681600	2.61876500	1.58306200
H	-0.03552700	2.12558000	2.09800600
H	-2.57567000	-1.69196700	0.64781300
H	-2.28542900	-1.27107300	-1.06053100
I	-4.79828800	-0.70950200	-0.39771500

IV

C	-0.29983900	2.23206500	-1.35869900
C	-1.68531400	2.84920100	-1.21198100
C	-2.69283600	1.79810600	-0.76459500
C	-2.31679600	1.13262300	0.57224700
C	-0.84966800	0.60636600	0.50039000
C	-0.36977100	-0.05294600	1.79603700
C	1.03374200	-0.56309200	1.69014100
C	1.87370400	-0.23996400	0.69403100
C	1.45934300	0.69076600	-0.43130600
C	0.23193000	1.57854100	-0.06717500
C	2.68735700	1.45760600	-0.93690000
C	3.78656200	0.49942100	-1.38202500
C	4.12263900	-0.52040300	-0.32161900
C	3.22062600	-0.81785000	0.62946100
C	5.50330100	-1.14218200	-0.39901500
C	6.56548100	-0.16750300	0.13232100
C	5.62444800	-2.49792900	0.29395400
C	-3.30358800	-0.01619000	0.86420800
C	-2.58516600	2.12678300	1.72250000
C	0.65811600	2.66109000	0.93810400
H	0.41323700	2.99270900	-1.71307000
H	-0.34878500	1.45915500	-2.14653000
H	-1.65779900	3.69134600	-0.50084500
H	-2.00338700	3.27917500	-2.17454400
H	-2.75261200	1.01967300	-1.54420100
H	-3.70264700	2.23438000	-0.68121300
H	-0.89348600	-0.20166500	-0.25089100
H	-0.43806600	0.64704100	2.64881600
H	-1.02947700	-0.89780700	2.05539500
H	1.37944100	-1.24366900	2.47570000
H	1.13555700	0.03989500	-1.26716900
H	3.08683600	2.09439200	-0.13089300
H	2.41254400	2.12216300	-1.76927400
H	4.69096400	1.06589300	-1.65859700
H	3.47924300	-0.03260700	-2.30210000
H	3.46272000	-1.55162000	1.40342400
H	5.71038600	-1.29816500	-1.47443700
H	7.57904700	-0.57161700	-0.01506800
H	6.41815400	0.00452200	1.21018800
H	6.51899000	0.80919400	-0.37202700
H	6.61349600	-2.94043000	0.10107300
H	4.85729600	-3.20191100	-0.06078600
H	5.51879500	-2.40294800	1.38630600
H	-3.66397300	2.33512700	1.79715400
H	-2.08700900	3.08992800	1.56100100
H	-2.25540300	1.73791100	2.69607800
H	1.16014500	2.22554600	1.81452400
H	1.36253800	3.36481000	0.47119600
H	-0.18848700	3.25531600	1.30045000
H	-4.33586900	0.34924200	0.78796400
H	-3.16099100	-0.43108800	1.86935300
I	-3.26918900	-1.74964000	-0.43919700

V

C	0.19307600	2.33318100	-0.63040900
C	-1.26542400	2.72559800	-0.44176100
C	-2.17112000	1.51813200	-0.63570500
C	-1.87881000	0.36737300	0.34666000
C	-0.34156400	0.03068200	0.29349300
C	0.08927800	-1.05322300	1.28835000
C	1.54850400	-1.37344800	1.18805300
C	2.43733700	-0.61019400	0.53261900
C	2.02408600	0.66990200	-0.16969000
C	0.66216100	1.23062700	0.33770300
C	3.19103100	1.66387400	-0.13251800
C	4.43760600	1.06130000	-0.77229700
C	4.79636500	-0.27814500	-0.17226800
C	3.84702400	-1.00603200	0.44193800
C	6.24111300	-0.71548800	-0.30973400
C	6.62835100	-0.85154500	-1.79015500
C	6.59535300	-1.98663300	0.45697600
C	-2.58392900	-0.91402300	-0.14012800
C	-2.39765000	0.72322600	1.74724000
C	0.83941200	1.81517100	1.74914800
H	0.83900400	3.21889800	-0.52735600
H	0.32318900	1.97170400	-1.66654100
H	-1.41995900	3.16941100	0.55548400
H	-1.53736800	3.50949200	-1.16563700
H	-2.03884700	1.14420000	-1.66722000
H	-3.22880700	1.81070000	-0.54814300
H	-0.19356900	-0.39871300	-0.71519000
H	-0.14640100	-0.74800600	2.32379600
H	-0.48423000	-1.97911100	1.12306500
H	1.89548000	-2.28731600	1.68229500
H	1.87654200	0.40804400	-1.23612800
H	3.42415900	1.92650700	0.91202800
H	2.92250000	2.59652500	-0.64994000
H	5.29069200	1.75253600	-0.66507100
H	4.27925100	0.94993300	-1.86084800
H	4.10019700	-1.96657900	0.89797900
H	6.85168500	0.10696500	0.11018500
H	7.69017100	-1.12320000	-1.89169200
H	6.02696900	-1.63830800	-2.27327500
H	6.47090400	0.08392300	-2.34661700
H	7.67161300	-2.19758400	0.36842900
H	6.05352100	-2.85930400	0.05867900
H	6.35641300	-1.89627800	1.52694200
H	-3.48974300	0.85306900	1.72012400
H	-1.97186400	1.65997100	2.12513000
H	-2.17881100	-0.06587700	2.48107500
H	1.31004200	1.09447100	2.43358700
H	1.48420100	2.70538300	1.71583000
H	-0.11100800	2.12829100	2.19606400
H	-2.47176100	-1.73831900	0.57289400
H	-2.19284300	-1.23402500	-1.11469600
I	-4.73121800	-0.81244200	-0.44528900

VI

C	0.18470100	2.30892300	-0.69477600
C	-1.25886800	2.72120400	-0.44409900
C	-2.19015200	1.52983900	-0.61496500
C	-1.87730400	0.36420000	0.34318800
C	-0.34864200	0.00484800	0.22579700
C	0.10463100	-1.09603800	1.19154900
C	1.55242600	-1.44001000	1.02528500
C	2.42667300	-0.68272100	0.34383300
C	2.00725600	0.61386600	-0.32474300
C	0.67481000	1.18876400	0.24226200
C	3.18866800	1.59194100	-0.32087100
C	4.40658700	0.98174400	-1.00573800
C	4.75975600	-0.37551600	-0.44853600
C	3.82309200	-1.10297300	0.18408800
C	6.19584000	-0.82552600	-0.63226000
C	7.11638400	-0.11800800	0.37404000
C	6.39597200	-2.33853300	-0.57096700
C	-2.62074100	-0.90112100	-0.12854600
C	-2.33531100	0.71301900	1.76661500
C	0.91651900	1.75467700	1.65184400
H	0.26798900	1.95705700	-1.73900100
H	0.84761900	3.18376300	-0.60830900
H	-1.54695700	3.51739500	-1.14803300
H	-1.36686900	3.15582300	0.56327300
H	-3.23901300	1.83711000	-0.48280000
H	-2.10414100	1.16537200	-1.65472600
H	-0.24730500	-0.41596500	-0.79221600
H	-0.08160400	-0.79721100	2.23884600
H	-0.49138300	-2.01031900	1.04214400
H	1.90335300	-2.36736700	1.49079700
H	1.81451800	0.36794500	-1.38776900
H	3.46158200	1.84148800	0.71732200
H	2.91384800	2.53306100	-0.81931800
H	5.26980500	1.66118300	-0.91429200
H	4.22357900	0.88305700	-2.09240600
H	4.07716800	-2.08339300	0.59671700
H	6.49604900	-0.49039500	-1.64284900
H	8.17226500	-0.36240700	0.18007300
H	6.87358800	-0.43600200	1.40019100
H	7.01366200	0.97646600	0.32944000
H	7.43430500	-2.59861200	-0.82660100
H	6.20314000	-2.73005100	0.44042900
H	5.72798600	-2.86239400	-1.27051300
H	-3.42564800	0.85832200	1.78433600
H	-1.88189200	1.64020300	2.13587100
H	-2.09875100	-0.08613600	2.48385100
H	1.40923200	1.02173700	2.30715200
H	1.56690900	2.64001400	1.60224600
H	-0.01141000	2.06995700	2.14236400
H	-2.49392700	-1.73449700	0.57134000
H	-2.27264700	-1.21675000	-1.12069700
I	-4.77639300	-0.76365900	-0.34914800

Molecule 5

I

C	-0.86918300	-1.00296800	0.08865300
C	-2.32106200	-1.20443000	0.05146800
C	-3.21730700	-0.21939400	-0.13223600
C	-2.74856800	1.20902100	-0.26786200
C	-1.37230200	1.42056900	0.35588500
C	-0.35869700	0.40164700	-0.17966700
H	-2.67426700	-2.23578800	0.16219700
H	-2.72338600	1.48445200	-1.33850300
H	-3.47880100	1.89271500	0.19515600
H	-1.02940900	2.44701400	0.15894000
H	-0.35069300	0.52378100	-1.28086000
H	-1.46464100	1.31336900	1.44887200
C	-4.70420300	-0.50056600	-0.19700300
H	-4.82347700	-1.59673400	-0.22558300
C	-5.43097600	0.00632100	1.05554600
H	-6.49919800	-0.25883200	1.01966600
H	-5.36766400	1.10263000	1.14484900
H	-4.99892100	-0.43268400	1.96693800
C	-5.33593900	0.06979000	-1.47170800
H	-6.39708500	-0.21506600	-1.54144800
H	-5.28933200	1.17054400	-1.48607600
H	-4.82263000	-0.30264600	-2.37112400
C	-0.03339800	-2.02923700	0.31423800
H	-0.45389100	-3.02335400	0.50180100
C	1.10850100	0.61766200	0.29658200
C	1.93636900	-0.57610000	-0.27791100
H	1.65241200	-0.60799000	-1.34707000
C	1.45810900	-1.89987400	0.32603400
H	1.82277500	-2.01173100	1.36347200
H	1.89427800	-2.74742800	-0.22633700
C	1.63070200	1.93809000	-0.30449400
H	1.35373400	1.96950900	-1.37390200
H	1.12388000	2.79197900	0.17198100
C	3.49354800	-0.43107500	-0.31017000
C	3.85292100	0.95043400	-0.89171000
H	4.94704000	1.08546400	-0.85249400
H	3.57784000	0.96270900	-1.96191100
C	3.14159700	2.10532300	-0.19938300
H	3.44798600	2.16989600	0.85780900
H	3.44212200	3.06013400	-0.65913100
C	4.06904700	-1.49847700	-1.25761800
H	5.14165600	-1.31615300	-1.43001000
H	3.97386800	-2.51439600	-0.84592300
H	3.56443600	-1.47957900	-2.23671100
C	4.18937400	-0.60965800	1.05009700
H	5.28090400	-0.62963000	0.90336700
H	3.90975600	-1.55612100	1.53539400
H	3.97636600	0.20301300	1.75605200
C	1.14711000	0.68785300	1.83232200
H	0.63478700	1.59319500	2.18977600
H	0.64538000	-0.17687300	2.29107600
H	2.17030900	0.72501100	2.22335800

II

C	-0.94480300	-0.82924100	0.15387800
C	-2.40831000	-0.93120700	0.17364600
C	-3.23965700	0.11913000	0.05495900
C	-2.66866900	1.51386700	-0.05446100
C	-1.26310700	1.60799900	0.53023000
C	-0.34403800	0.54439800	-0.08210600
H	-2.81195500	-1.94202700	0.27489900
H	-2.65058100	1.82565800	-1.11511000
H	-3.34003500	2.22683900	0.45380300
H	-0.85673300	2.61584000	0.35960500
H	-0.36695100	0.71024200	-1.17736600
H	-1.32531700	1.46310800	1.62098100
C	-4.75075500	0.00294600	0.02306500
H	-5.12707600	0.67154600	0.82118100
C	-5.30837000	0.53327100	-1.30657000
H	-6.40842500	0.49307600	-1.31189900
H	-4.94027900	-0.07656300	-2.14724000
H	-5.01378100	1.57661300	-1.49160900
C	-5.28590400	-1.39750400	0.30873200
H	-6.38576600	-1.38634200	0.34061100
H	-4.98490300	-2.10941300	-0.47636700
H	-4.92440900	-1.78197600	1.27393000
C	-0.17678000	-1.91989500	0.30696700
H	-0.65998100	-2.88856500	0.47598100
C	1.15090200	0.63741000	0.34462500
C	1.86983500	-0.58653900	-0.30753200
H	1.54635600	-0.55362900	-1.36537500
C	1.31951700	-1.89746800	0.26201500
H	1.67157800	-2.74940500	-0.34137400
H	1.71415100	-2.07952200	1.27834000
C	1.74380100	1.94175600	-0.22503300
H	1.31654500	2.80860000	0.30327500
H	1.43130700	2.03637800	-1.28083900
C	3.43129100	-0.54965300	-0.39426400
C	3.86719000	0.82606600	-0.93600800
H	4.96886700	0.88257500	-0.93416700
H	3.55534100	0.90135300	-1.99346700
C	3.26558700	1.99865200	-0.17281300
H	3.61419400	1.99848600	0.87321200
H	3.61654100	2.94808800	-0.60726100
C	4.16115200	-0.83179600	0.93015300
H	4.03310500	-0.03534800	1.67408600
H	3.83197500	-1.77506700	1.38981100
H	5.24237400	-0.92278200	0.74025300
C	3.89459300	-1.61521000	-1.40325400
H	4.97044900	-1.50110300	-1.61058200
H	3.35719000	-1.52179300	-2.36037600
H	3.74264200	-2.63782100	-1.02663700
C	1.24942700	0.64150700	1.87932000
H	0.81823200	1.56600300	2.29075700
H	0.70209200	-0.20270500	2.32387800
H	2.28583400	0.58872200	2.23176800

III

C	-0.94624900	-0.81846200	0.07723900
C	-2.40736200	-0.92829100	0.00204100
C	-3.22476000	0.09493400	-0.30073700
C	-2.64968400	1.46910800	-0.54309900
C	-1.28170400	1.64844600	0.10593200
C	-0.32774900	0.51684100	-0.29602000
H	-2.82181300	-1.92353000	0.18644500
H	-2.57895500	1.63746000	-1.63437000
H	-3.34685400	2.23844800	-0.17225100
H	-0.86164000	2.62595200	-0.17347100
H	-0.27559600	0.53575500	-1.40260600
H	-1.41326600	1.65240000	1.20019900
C	-4.73244400	-0.03079300	-0.40149200
H	-5.03863000	0.61080000	-1.24907000
C	-5.40725100	0.52928200	0.86002300
H	-6.50291500	0.53495600	0.75088700
H	-5.08795700	1.55972700	1.07595800
H	-5.15269300	-0.09054400	1.73414700
C	-5.22972900	-1.44532700	-0.69276000
H	-6.31657600	-1.44198100	-0.86613500
H	-5.03837200	-2.12228900	0.15489400
H	-4.74066300	-1.86941700	-1.58213500
C	-0.19291300	-1.87391800	0.42560200
H	-0.68880100	-2.81591500	0.68465100
C	1.13515400	0.67532500	0.21509300
C	1.89384400	-0.61969100	-0.21765100
H	1.64293800	-0.73050400	-1.28980400
C	1.30286900	-1.84638200	0.48352000
H	1.62512300	-1.88723600	1.54006500
H	1.69467200	-2.76894400	0.02622800
C	1.76918600	1.89615800	-0.48127200
H	1.52986400	1.84773700	-1.55910500
H	1.30929100	2.82277900	-0.10320000
C	3.45771800	-0.58466300	-0.20302000
C	3.93303500	0.70979000	-0.89191000
H	5.03218600	0.77308700	-0.82307300
H	3.69388600	0.64230800	-1.96866900
C	3.28399400	1.96912800	-0.33336800
H	3.55995700	2.10932400	0.72489000
H	3.66646600	2.85506900	-0.86456500
C	4.09581100	-0.68378600	1.19323200
H	3.73357100	-1.55922100	1.75164000
H	5.18706100	-0.79293600	1.09013200
H	3.92058300	0.20409400	1.81385500
C	3.98532800	-1.77167500	-1.02809500
H	5.07311600	-1.67950400	-1.17539700
H	3.51445000	-1.80912200	-2.02333100
H	3.80503900	-2.73631400	-0.53043900
C	1.12877000	0.88373900	1.73877000
H	0.67438700	1.85256500	1.99337100
H	0.54859600	0.10331300	2.25285300
H	2.13835200	0.88311300	2.16524500

Molecule 6

I

C	-0.57844500	-2.44536000	0.59450300
C	-1.83144700	-3.17057600	1.07332100
C	-3.00463900	-2.20337500	1.19292400
C	-3.33290400	-1.51617900	-0.15005700
C	-2.03113800	-0.85184800	-0.71218800
C	-2.25951500	-0.12081900	-2.03548600
C	-1.01733800	0.56192200	-2.51303200
C	0.20778800	0.28972000	-2.03469700
C	0.44213800	-0.74663100	-0.95157100
C	-0.74778300	-1.73239300	-0.76333700
C	1.80650300	-1.41048300	-1.17357700
C	2.92538400	-0.37406700	-1.18152000
C	2.64371600	0.77080200	-2.12157300
C	1.38195600	1.02957200	-2.50718300
C	3.80799200	1.60931400	-2.60607000
C	4.59919700	2.20550200	-1.43653800
C	4.72446800	0.82322800	-3.55218100
C	-4.31245400	-0.35846600	0.08543700
C	-4.03598500	-2.50257300	-1.09121400
C	-0.76263400	-2.75363300	-1.91109700
O	-5.32006100	-0.17146100	-0.54690400
O	-3.90542000	0.46305500	1.06379300
H	-0.30732700	-1.68798300	1.35257100
H	0.26745300	-3.14931400	0.54525500
H	-2.07868500	-4.00051400	0.39144600
H	-1.63744100	-3.63372100	2.05343600
H	-2.75041100	-1.43128100	1.93511100
H	-3.90250700	-2.72216500	1.56686800
H	-1.78841600	-0.07414200	0.02867600
H	-3.06550800	0.62395500	-1.92397800
H	-2.62310700	-0.81494400	-2.81433800
H	-1.12537100	1.32303100	-3.29330700
H	0.52583000	-0.18012100	-0.00656300
H	1.81015900	-1.94313700	-2.13827400
H	2.00329600	-2.15812300	-0.39025600
H	3.88215800	-0.85516000	-1.44192600
H	3.06453900	0.02816400	-0.16446000
H	1.19276100	1.85550700	-3.20181700
H	3.38181300	2.44763200	-3.18236500
H	5.39757600	2.86897300	-1.80297900
H	5.07715900	1.41883500	-0.83039800
H	3.94515800	2.79259500	-0.77382700
H	5.51884700	1.47298900	-3.95113000
H	5.21650300	-0.01676700	-3.03658700
H	4.15763900	0.41426700	-4.40162800
H	-4.20706500	-2.07635700	-2.08760700
H	-3.46758000	-3.43284500	-1.20414700
H	-5.02270300	-2.76221900	-0.68275400
H	-1.64904200	-3.39752400	-1.88866000
H	0.11534100	-3.41342000	-1.85246500
H	-0.73623000	-2.25649700	-2.89237100
C	-4.64760900	1.66385800	1.28316700
H	-4.58989000	1.84971900	2.36584200
H	-5.69450900	1.48337500	1.00465100
C	-4.08347500	2.84282800	0.49876500
H	-3.96481800	2.53743800	-0.55473600
H	-4.85786400	3.62747600	0.49869900

C	-2.78007100	3.43417500	1.04069500
H	-2.94130700	3.74423300	2.08947800
H	-2.55530200	4.36136200	0.48625100
C	-1.57260600	2.50381000	0.97410200
H	-1.81842200	1.55756300	1.48130300
H	-1.36933600	2.23535700	-0.07856100
C	-0.30610500	3.06827900	1.60964000
H	-0.51133100	3.30999000	2.66844600
H	-0.02524200	4.02225000	1.12905200
C	0.85532600	2.08127700	1.53400700
H	0.47179500	1.08802400	1.82331600
H	1.18703700	1.98212000	0.48399800
C	2.04785700	2.42891900	2.42035300
H	2.38940900	3.45303100	2.19098300
H	1.71662000	2.44883500	3.47439400
C	3.22770500	1.46561800	2.27672600
H	4.03682800	1.77718500	2.95909300
H	3.64451600	1.55753300	1.25872000
C	2.88831100	-0.00023900	2.54106300
H	2.44320900	-0.10220200	3.54731500
H	2.10828200	-0.33610300	1.83582800
C	4.08405600	-0.93830400	2.41139000
H	4.56724000	-0.77619200	1.43047300
H	4.84655000	-0.67702600	3.16693400
C	3.71286800	-2.41281200	2.54580600
H	2.94199700	-2.65657900	1.79329800
H	3.23563400	-2.57918100	3.52716800
C	4.89998300	-3.35496200	2.38424100
H	4.59844600	-4.40823500	2.48482700
H	5.67327100	-3.15458400	3.14278200
H	5.36961000	-3.23448300	1.39489600

II

C	3.19164500	-2.62171800	-1.06187300
C	4.70600300	-2.55426700	-0.89517000
C	5.17272200	-1.10855800	-0.76666100
C	4.52789300	-0.39375600	0.44065800
C	2.96999400	-0.54270600	0.34961000
C	2.23839100	0.15000700	1.50063700
C	0.75258000	0.01431300	1.39115000
C	0.14243100	-0.84797100	0.56189500
C	0.92657400	-1.76708700	-0.35921700
C	2.40420800	-1.97011000	0.09378400
C	0.13684400	-3.06273400	-0.57976400
C	-1.25393000	-2.77585600	-1.13722300
C	-2.00025500	-1.76553900	-0.30100400
C	-1.32016800	-0.90981200	0.48201800
C	-3.51382400	-1.76423800	-0.34961700
C	-4.04093800	-1.70260800	-1.78652400
C	-4.10354200	-2.96268400	0.40479700
C	4.80055100	1.11294900	0.34680000
C	5.16824300	-0.87991000	1.74653400
C	2.44742500	-2.86716700	1.34090200
O	5.25911100	1.78848900	1.23210500
O	4.42784500	1.62109200	-0.83559000
H	2.92528400	-2.10423900	-2.00102100
H	2.87596500	-3.66970400	-1.18425300
H	5.02285200	-3.14502200	-0.02029700
H	5.19327900	-3.02071900	-1.76566000

H	4.90357600	-0.56723200	-1.68660200
H	6.27006300	-1.05820200	-0.67589100
H	2.70707400	0.01169800	-0.56628700
H	2.50527700	1.22017900	1.52971300
H	2.56938200	-0.25000100	2.47558800
H	0.13911800	0.65492700	2.03422200
H	0.96795500	-1.25721800	-1.34216700
H	0.02421800	-3.59579000	0.37816300
H	0.67988600	-3.73343500	-1.26188600
H	-1.83130700	-3.71262000	-1.20236700
H	-1.17594500	-2.40345700	-2.17543600
H	-1.87474600	-0.19115200	1.09530800
H	-3.85104700	-0.85359100	0.17155600
H	-5.14004800	-1.63614100	-1.79445900
H	-3.76290800	-2.60246000	-2.35796000
H	-3.64430800	-0.82555800	-2.32075200
H	-5.20378700	-2.91248700	0.41092400
H	-3.81944300	-3.91809000	-0.06492500
H	-3.75517500	-2.98143300	1.44788800
H	4.68726000	-0.44676700	2.63242500
H	5.13763800	-1.97204000	1.83381200
H	6.22260000	-0.57145100	1.77576200
H	3.45606200	-2.94716700	1.76190000
H	2.12106800	-3.88798600	1.09398100
H	1.78391800	-2.49132600	2.13369000
C	4.38093600	3.04252300	-0.96291700
H	4.41602300	3.23015900	-2.04519800
H	5.27004000	3.48190800	-0.48917700
C	3.10390100	3.59506300	-0.34726000
H	3.13162000	3.39819200	0.73744300
H	3.11292200	4.69262500	-0.45641500
C	1.83431200	3.00611500	-0.95751600
H	1.94679400	1.91260300	-1.03486800
H	1.71617300	3.37132700	-1.99330200
C	0.56936000	3.29494000	-0.15813300
H	0.70235400	2.90523500	0.86692000
H	0.42444300	4.38500800	-0.05151000
C	-0.67458400	2.66165600	-0.77244200
H	-0.46300600	1.59986700	-0.99141400
H	-0.89092500	3.13898900	-1.74513700
C	-1.90817000	2.73141600	0.11981200
H	-1.69440600	2.19019700	1.05929000
H	-2.10441500	3.77781500	0.41495100
C	-3.15386800	2.13819400	-0.53036600
H	-3.46620100	2.77439000	-1.37772100
H	-2.89450800	1.15746900	-0.96724200
C	-4.32273900	1.95218500	0.43108100
H	-4.54200600	2.90385800	0.94717200
H	-4.01875800	1.24691700	1.22702300
C	-5.58766500	1.43206900	-0.24610000
H	-5.99107600	2.21068400	-0.91763000
H	-5.32370900	0.58445700	-0.90136100
C	-6.67066000	0.98390200	0.72945600
H	-6.25564200	0.20475000	1.39508100
H	-6.94659400	1.82616400	1.38940200
C	-7.92299800	0.44162400	0.04565900
H	-7.63670100	-0.39508900	-0.61589200
H	-8.33950100	1.21928200	-0.61793100
C	-8.99444700	-0.02469900	1.02471300
H	-9.88189700	-0.41064000	0.50127800

H	-9.32313800	0.80030200	1.67656300
H	-8.61466100	-0.82784600	1.67627300

III

C	3.09642100	-2.64620200	-1.08479900
C	4.61588200	-2.59392500	-0.96544700
C	5.10156600	-1.15239000	-0.86306200
C	4.50205800	-0.42124200	0.35773100
C	2.94073600	-0.55489300	0.31740700
C	2.25359000	0.15277000	1.48659500
C	0.76371500	0.03196100	1.42551900
C	0.11861800	-0.82865900	0.62137200
C	0.86371300	-1.76123000	-0.31849800
C	2.35230000	-1.97788500	0.08974000
C	0.05342000	-3.04897800	-0.50784900
C	-1.35003900	-2.74829300	-1.02464000
C	-2.05947400	-1.72612500	-0.17079200
C	-1.34641800	-0.87488000	0.58739100
C	-3.57360400	-1.70738300	-0.17693600
C	-4.15654400	-2.90240000	0.58791600
C	-4.13867500	-1.63424800	-1.59878000
C	4.78637200	1.08180300	0.24226700
C	5.17769800	-0.90336300	1.64715600
C	2.42434900	-2.86668800	1.34142100
O	5.27996600	1.76010400	1.10636800
O	4.38046100	1.58379900	-0.93183900
H	2.80661400	-2.13265100	-2.01916200
H	2.76595600	-3.69154700	-1.18944900
H	4.95337100	-3.18126600	-0.09599900
H	5.07102700	-3.07229400	-1.84679400
H	4.80973900	-0.61548900	-1.77866400
H	6.20166300	-1.11309800	-0.80664600
H	2.65428300	-0.00396400	-0.59343900
H	2.53228900	1.22024300	1.50009000
H	2.61188600	-0.24471700	2.45290400
H	0.17771500	0.68300200	2.08357200
H	0.88015800	-1.25688500	-1.30502700
H	-0.03602600	-3.57601500	0.45584300
H	0.56784800	-3.72913500	-1.20268000
H	-1.93985500	-3.67845600	-1.06880200
H	-1.29862300	-2.38066100	-2.06617600
H	-1.87393000	-0.14719800	1.21377200
H	-3.88664700	-0.79527400	0.35697000
H	-5.25549800	-2.83912500	0.62310800
H	-3.89705200	-3.85886600	0.10639800
H	-3.78106000	-2.93028400	1.62135400
H	-5.23653400	-1.56094600	-1.57505000
H	-3.88221400	-2.53301400	-2.18186100
H	-3.75001200	-0.75724600	-2.13893200
H	4.72796000	-0.45861500	2.54369400
H	5.13900400	-1.99448000	1.74395400
H	6.23540500	-0.60520400	1.64172600
H	3.44456000	-2.95483600	1.73187800
H	2.07965100	-3.88557400	1.11203900
H	1.78955600	-2.47819500	2.15143000
C	4.34169500	3.00448900	-1.06927000
H	4.34437500	3.18338400	-2.15359600
H	5.24903400	3.43952800	-0.62708800
C	3.08954800	3.57317700	-0.41819000

H	3.15001800	3.38471900	0.66668400
H	3.10463400	4.66971000	-0.53659700
C	1.79572300	2.99112500	-0.98267100
H	1.89522300	1.89597300	-1.05373100
H	1.64788400	3.34858900	-2.01733700
C	0.56013700	3.29932000	-0.14548300
H	0.72320300	2.91818900	0.87846100
H	0.42889600	4.39170400	-0.04490800
C	-0.70971100	2.67240500	-0.71162100
H	-0.51668700	1.60598900	-0.92530100
H	-0.95351000	3.14152200	-1.68180900
C	-1.91131500	2.76594400	0.22119200
H	-1.67141300	2.23239500	1.15877000
H	-2.08499600	3.81771600	0.51137700
C	-3.18640800	2.18120200	-0.37773400
H	-3.52107200	2.81189000	-1.22067600
H	-2.95532700	1.19229600	-0.81179000
C	-4.32122600	2.02292100	0.62833500
H	-4.50577700	2.98326300	1.14210600
H	-3.99858400	1.32109800	1.42006200
C	-5.62018500	1.51577000	0.00780400
H	-6.03695000	2.29159200	-0.65951000
H	-5.39016700	0.65571300	-0.64227400
C	-6.66779300	1.11148800	1.04173900
H	-6.83746800	1.95655000	1.73117500
H	-6.25788800	0.29351400	1.66284100
C	-8.00915500	0.66965000	0.45449200
H	-8.45064400	1.50700400	-0.11381300
H	-8.70728500	0.46656900	1.28334700
C	-7.92623700	-0.56258600	-0.44259800
H	-8.92634000	-0.89755900	-0.75558100
H	-7.44147600	-1.40241800	0.08208500
H	-7.34688000	-0.36428400	-1.35740200

IV

C	1.94002500	-2.10748500	-2.04989200
C	3.39435500	-2.16126000	-2.50494700
C	4.15806700	-0.93509300	-2.01742600
C	4.13913300	-0.81150000	-0.47750800
C	2.65524600	-0.85442000	0.01897300
C	2.53296700	-0.72054100	1.53728200
C	1.10517800	-0.72726500	1.98635900
C	0.08439400	-1.13142200	1.21234700
C	0.29268600	-1.60936200	-0.21279900
C	1.76520100	-2.01308600	-0.52008300
C	-0.74954900	-2.68599300	-0.54073700
C	-2.16592800	-2.15823500	-0.34745800
C	-2.36169600	-1.50484100	0.99772300
C	-1.29909800	-1.06982000	1.69722200
C	-3.79196000	-1.39776200	1.48939300
C	-4.03898500	-0.24023200	2.45463800
C	-4.25752500	-2.72872300	2.09734500
C	4.68741100	0.56532800	-0.08084300
C	5.08145000	-1.85124600	0.14329400
C	2.07586300	-3.36650200	0.13937600
O	5.58751300	0.74854800	0.69867200
O	4.04553000	1.57040200	-0.69179200
H	1.46846800	-1.21997400	-2.50875900
H	1.39481500	-2.98339600	-2.43530700

H	3.87475400	-3.08674100	-2.14798300
H	3.43534800	-2.20535700	-3.60456700
H	3.69616100	-0.03542100	-2.45221200
H	5.20287600	-0.95949200	-2.36812200
H	2.20228200	0.05592600	-0.40784400
H	3.01862300	0.21131500	1.87501400
H	3.08276700	-1.52876800	2.05182300
H	0.89451800	-0.38509300	3.00541400
H	0.06121600	-0.74136500	-0.86141800
H	-0.60668800	-3.55296000	0.12412000
H	-0.62317300	-3.04525500	-1.57276600
H	-2.89522600	-2.97580600	-0.47310400
H	-2.41101100	-1.42300300	-1.13693400
H	-1.44284400	-0.60757100	2.67813800
H	-4.41113700	-1.21577100	0.59171700
H	-5.11488500	-0.13570700	2.66201800
H	-3.67446900	0.71108000	2.03926300
H	-3.53525400	-0.40316600	3.42050000
H	-5.32670200	-2.68966000	2.35755700
H	-3.68857700	-2.94909200	3.01418100
H	-4.11215300	-3.56957800	1.40270700
H	5.03033100	-1.85866900	1.23937700
H	4.86893600	-2.86263400	-0.22079200
H	6.11978500	-1.61163900	-0.12390800
H	3.13275700	-3.64172000	0.05483500
H	1.49648800	-4.17046200	-0.33740500
H	1.81701400	-3.36326200	1.20872000
C	4.44548200	2.90003500	-0.35413700
H	4.16984000	3.51180600	-1.22550200
H	5.53767400	2.91588700	-0.23538700
C	3.76923100	3.41173200	0.91109900
H	3.87158700	2.64201700	1.69368500
H	4.35505900	4.27544700	1.26613700
C	2.31012800	3.83853500	0.75337400
H	2.25933800	4.68834800	0.04836200
H	1.95879200	4.23487400	1.72167200
C	1.34721700	2.74968900	0.28658300
H	1.63396700	2.40565100	-0.72141500
H	1.43780700	1.86501900	0.94028200
C	-0.10755500	3.20733500	0.26896000
H	-0.19391600	4.14237700	-0.31416100
H	-0.42062500	3.46450100	1.29702300
C	-1.06131000	2.16426300	-0.30218600
H	-0.78694300	1.95764200	-1.35285800
H	-0.92604200	1.21342700	0.24016800
C	-2.53041100	2.56784900	-0.23161300
H	-2.81416900	2.70814600	0.82746300
H	-2.67315900	3.55211100	-0.71317700
C	-3.46279300	1.54491200	-0.87074000
H	-3.24297000	0.55466600	-0.44008900
H	-3.23264200	1.46309600	-1.94838400
C	-4.94547900	1.84957800	-0.68772300
H	-5.17367000	1.89818500	0.39228500
H	-5.16980700	2.85455600	-1.08827300
C	-5.86276000	0.82479300	-1.34805700
H	-5.65789400	0.79545100	-2.43348600
H	-5.61233500	-0.18335600	-0.97034600
C	-7.34850500	1.08905700	-1.11985800
H	-7.59868800	2.09672600	-1.49492500
H	-7.54701500	1.11502700	-0.03411500

C	-8.25472500	0.05656700	-1.78087000
H	-9.31768700	0.27281800	-1.59697800
H	-8.04843500	-0.95582500	-1.39822200
H	-8.10195400	0.03374500	-2.87163800

V

C	3.25091600	-2.54538500	-1.12531700
C	4.76656400	-2.41975500	-1.01312800
C	5.18146900	-0.95737600	-0.89583700
C	4.55411000	-0.27139600	0.33713800
C	3.00109200	-0.48112400	0.30330300
C	2.28692300	0.17763600	1.48482100
C	0.80449700	-0.01571400	1.43028100
C	0.19763400	-0.89707900	0.61886400
C	0.98189300	-1.77975500	-0.33716400
C	2.48155500	-1.92836800	0.06098600
C	0.23481400	-3.10328500	-0.53936000
C	-1.18557300	-2.86603200	-1.04293600
C	-1.93874300	-1.89206000	-0.17042500
C	-1.26337800	-1.01570500	0.59360500
C	-3.45189500	-1.94987300	-0.16206300
C	-4.03515100	-1.89310000	-1.57737000
C	-3.96533900	-3.17910800	0.59863600
C	4.76299800	1.24517500	0.23896700
C	5.26051000	-0.73516400	1.61673500
C	2.60344100	-2.82712400	1.30163500
O	5.22906200	1.93628300	1.10817900
O	4.32321300	1.74118400	-0.92546400
H	2.93088600	-2.03576700	-2.05193700
H	2.97185900	-3.60446600	-1.24085400
H	5.13742800	-2.99996300	-0.15253500
H	5.23962600	-2.86475900	-1.90248300
H	4.85815700	-0.42457300	-1.80322500
H	6.27862600	-0.86474600	-0.84488700
H	2.68300200	0.06628700	-0.59912900
H	2.51282400	1.25728200	1.51029600
H	2.66997800	-0.21355300	2.44418000
H	0.19113000	0.59747300	2.09967500
H	0.96817800	-1.26241500	-1.31698600
H	0.17843700	-3.64715600	0.41744100
H	0.77751100	-3.74751400	-1.24680100
H	-1.72840900	-3.82389800	-1.09628800
H	-1.16001700	-2.48223100	-2.07960200
H	-1.82202500	-0.32335700	1.23296900
H	-3.80419600	-1.05895000	0.38314800
H	-5.13558000	-1.87183000	-1.54356400
H	-3.74248500	-2.77409400	-2.17044500
H	-3.69511800	-0.99429500	-2.11441300
H	-5.06554000	-3.17140600	0.64839400
H	-3.66403500	-4.11718100	0.10534000
H	-3.57562000	-3.19695900	1.62700400
H	4.79514800	-0.32389700	2.52125000
H	5.27619300	-1.82791300	1.70031200
H	6.30223800	-0.38528800	1.60904800
H	3.62830200	-2.86820900	1.68771600
H	2.30972600	-3.85938800	1.06140300
H	1.95275400	-2.48020900	2.11794200
C	4.21148900	3.15983300	-1.04295400
H	4.19839900	3.35356200	-2.12464400

H	5.09817800	3.63424900	-0.59938500
C	2.93595900	3.65460000	-0.37704100
H	3.01274400	3.45453300	0.70475700
H	2.89410900	4.75199200	-0.48009600
C	1.67027800	3.01480200	-0.94273500
H	1.82395500	1.92676600	-1.02611600
H	1.50054400	3.37552100	-1.97291300
C	0.42414100	3.25225800	-0.09814000
H	0.60901700	2.86791000	0.92086200
H	0.23977400	4.33566300	0.01541400
C	-0.81517100	2.57046400	-0.66843100
H	-0.57125600	1.51697000	-0.89358300
H	-1.08300600	3.03716400	-1.63343500
C	-2.01870300	2.59677300	0.26629000
H	-1.75285500	2.06638000	1.19867800
H	-2.24255100	3.63622800	0.56639200
C	-3.26438900	1.95771200	-0.33882400
H	-3.62010500	2.57358800	-1.18412200
H	-2.98864300	0.97967200	-0.77132700
C	-4.39863700	1.75231500	0.65958100
H	-4.63172300	2.70661300	1.16490800
H	-4.05112200	1.07089500	1.45851700
C	-5.66716600	1.18539200	0.02857900
H	-6.07593500	1.92648600	-0.67933200
H	-5.40495400	0.30373100	-0.58162600
C	-6.73119300	0.78378300	1.04692600
H	-7.04811800	1.67546200	1.61788000
H	-6.27268000	0.10151100	1.78404500
C	-7.96075700	0.10072300	0.44521500
H	-8.61530300	-0.23582200	1.26622500
H	-7.64022600	-0.81666900	-0.08014200
C	-8.76754800	0.97609500	-0.50965900
H	-9.67564100	0.45861600	-0.85353700
H	-8.18784000	1.24983800	-1.40437000
H	-9.08177700	1.91144900	-0.01882100

VI

C	-1.12149900	-0.01198000	2.45444000
C	-2.58364000	0.22565100	2.81224700
C	-3.42643000	0.36994800	1.55016700
C	-3.35378200	-0.88280400	0.65206100
C	-1.85034900	-1.22857600	0.36706000
C	-1.67054200	-2.47208300	-0.50570200
C	-0.23062900	-2.69205300	-0.84931700
C	0.78195400	-2.11080000	-0.18509700
C	0.55131600	-1.17531000	0.98639000
C	-0.88183500	-1.26178400	1.58502800
C	1.69017900	-1.34911800	1.99748000
C	3.03955100	-1.05395800	1.35108300
C	3.23396000	-1.79620200	0.05255200
C	2.17379900	-2.29731300	-0.60603800
C	4.64052600	-1.93659400	-0.49107600
C	5.30797100	-0.56787400	-0.67168600
C	5.50372000	-2.86560400	0.37128600
C	-3.93673000	-0.57718800	-0.73208400
C	-4.18815900	-2.02003400	1.25242700
C	-1.02106400	-2.53746200	2.43013700
O	-4.68205500	-1.30190700	-1.33929300
O	-3.46108100	0.57077800	-1.23662300

H	-0.75833400	0.86857100	1.89593900
H	-0.51229000	-0.06859100	3.37022700
H	-2.96358000	-0.59220000	3.44624100
H	-2.66982000	1.14227700	3.41692400
H	-3.06049100	1.23884000	0.98238700
H	-4.47938100	0.57530800	1.80301900
H	-1.49142900	-0.37598400	-0.23416700
H	-2.26119800	-2.38195100	-1.43158800
H	-2.07528000	-3.36802500	-0.00187500
H	-0.00311300	-3.35596900	-1.69028600
H	0.65389000	-0.15247500	0.57700800
H	1.69465200	-2.38378900	2.37699600
H	1.54340900	-0.68623000	2.86339700
H	3.85515900	-1.29805000	2.05100600
H	3.12831400	0.02989200	1.16168200
H	2.33433600	-2.84923100	-1.53882800
H	4.55338800	-2.39913100	-1.48852200
H	6.28832000	-0.67350800	-1.16107900
H	5.47640500	-0.07181200	0.29779700
H	4.68767100	0.09978000	-1.28938600
H	6.49619500	-3.00631800	-0.08427400
H	5.66193600	-2.45255200	1.38017900
H	5.03340300	-3.85414800	0.47956700
H	-4.10483700	-2.94599800	0.67012600
H	-3.89994900	-2.23252200	2.28873000
H	-5.25078800	-1.73785700	1.25305400
H	-2.05191800	-2.70754000	2.76259500
H	-0.39747100	-2.47075600	3.33369500
H	-0.69806700	-3.42954900	1.87318600
C	-3.56336800	0.77962400	-2.64465700
H	-3.70042700	1.86231300	-2.77815100
H	-4.44730600	0.25213300	-3.02732900
C	-2.28600700	0.29841100	-3.31668600
H	-2.16197400	-0.77616200	-3.09810600
H	-2.41054900	0.37563900	-4.40995600
C	-1.04826700	1.07059900	-2.86668500
H	-1.06869700	1.15319800	-1.76830300
H	-1.11142600	2.10582500	-3.24853400
C	0.27234300	0.44429200	-3.31113400
H	0.23117300	0.24903700	-4.39659500
H	0.38823800	-0.53943000	-2.82145700
C	1.49068000	1.31765300	-3.00566800
H	1.33167700	2.31633800	-3.45220700
H	2.37959800	0.90108900	-3.50911800
C	1.79321300	1.47637300	-1.51621200
H	0.85432200	1.66951400	-0.97148000
H	2.17813200	0.51997100	-1.12249400
C	2.77725400	2.60450600	-1.21890000
H	3.68894200	2.46443300	-1.82579200
H	2.33727400	3.55607200	-1.56611200
C	3.18295200	2.73157800	0.25224400
H	3.80307100	3.63657500	0.37087000
H	3.83886800	1.88510600	0.51634700
C	2.02473100	2.78486900	1.25209100
H	1.49352200	1.81653800	1.26102200
H	2.44067600	2.89869400	2.26804800
C	1.01497500	3.90162700	0.99870000
H	1.54366400	4.87126300	0.95333400
H	0.55028000	3.76735100	0.00594200
C	-0.08899200	3.97378600	2.05091000

H	0.36500600	4.13343400	3.04437000
H	-0.59610400	2.99531800	2.10728200
C	-1.11912600	5.06256200	1.77419200
H	-1.89815100	5.08980300	2.55066400
H	-1.61841500	4.89847000	0.80588300
H	-0.64766800	6.05759600	1.73812000

VII

C	-3.09595500	-2.62250800	1.08643500
C	-4.60501300	-2.59292800	0.86836500
C	-5.09828900	-1.16152000	0.68950700
C	-4.42911500	-0.46065100	-0.51266100
C	-2.87217600	-0.57337100	-0.36769300
C	-2.11800600	0.10810000	-1.51095300
C	-0.63381800	0.00295600	-1.35508200
C	-0.03236900	-0.82583600	-0.48623500
C	-0.82766000	-1.73381500	0.43678400
C	-2.28488800	-1.98136000	-0.05863500
C	-0.02097900	-3.00541600	0.72422300
C	1.34725700	-2.67333200	1.31135400
C	2.10079700	-1.68159600	0.45948100
C	1.42845400	-0.86146300	-0.36695200
C	3.61311200	-1.66787100	0.53502800
C	4.11861700	-1.58059700	1.97792000
C	4.21521100	-2.87790900	-0.19067900
C	-4.73476400	1.04176900	-0.46314100
C	-5.01506900	-0.98993500	-1.82719300
C	-2.26845800	-2.90933100	-1.28357600
O	-5.17135000	1.68867800	-1.38033800
O	-4.41738300	1.58254500	0.72097200
H	-2.87215400	-2.07706600	2.02086700
H	-2.76214000	-3.66000800	1.24469400
H	-4.87962800	-3.21095300	-0.00191800
H	-5.11092600	-3.04956500	1.73340800
H	-4.87194600	-0.59307100	1.60453700
H	-6.19281700	-1.13788800	0.56143200
H	-2.65160400	0.00796400	0.54258100
H	-2.40334800	1.17216100	-1.57141500
H	-2.41212400	-0.31867400	-2.48638600
H	-0.01369300	0.63734700	-1.99795800
H	-0.91283600	-1.19557000	1.40166000
H	0.13021900	-3.56844200	-0.21096400
H	-0.57176000	-3.66293900	1.41289600
H	1.93754800	-3.59677200	1.42863300
H	1.23232200	-2.26397300	2.33207600
H	1.98832300	-0.15607100	-0.99087700
H	3.95681000	-0.76468800	0.00501900
H	5.21678600	-1.50166300	1.99896000
H	3.84304600	-2.47515700	2.55876800
H	3.70567900	-0.70041000	2.49430800
H	5.31542600	-2.83679900	-0.16963000
H	3.91181200	-3.82596600	0.28176500
H	3.89405600	-2.90528900	-1.24227300
H	-4.51272100	-0.56746000	-2.70636300
H	-4.95972100	-2.08290300	-1.88742400
H	-6.07379100	-0.70420200	-1.89913800
H	-3.26186000	-3.02480800	-1.73175600
H	-1.92554400	-3.91552100	-1.00148500
H	-1.59083400	-2.53697300	-2.06590100

C	-4.39647200	3.00694700	0.81748100
H	-4.47997300	3.21835400	1.89271500
H	-5.27081500	3.42217500	0.29686400
C	-3.10205700	3.56400600	0.24355600
H	-3.08161500	3.34376300	-0.83681700
H	-3.12950200	4.66340000	0.32823500
C	-1.85194300	3.00510600	0.91899900
H	-1.95517700	1.91249300	1.01729100
H	-1.78098700	3.39530400	1.94995100
C	-0.55839300	3.29118100	0.16600100
H	-0.64496100	2.87955500	-0.85539200
H	-0.42018600	4.38047300	0.04341700
C	0.66596300	2.68477600	0.84332100
H	0.46282700	1.62167500	1.06381300
H	0.82908100	3.17593100	1.81953100
C	1.93766400	2.76984600	0.00792600
H	1.78164100	2.20994100	-0.93190900
H	2.12210400	3.81665100	-0.29362500
C	3.16643200	2.22080100	0.72490300
H	3.40373700	2.86516800	1.59044300
H	2.92301200	1.22805900	1.14325300
C	4.39499000	2.09164100	-0.16902700
H	4.58401800	3.04901700	-0.68688500
H	4.17362000	1.36122100	-0.96767800
C	5.65286700	1.67567700	0.59182900
H	5.93467700	2.49368300	1.27622500
H	5.41741100	0.81291200	1.23921700
C	6.84570900	1.31985300	-0.29649300
H	7.00777000	2.12517700	-1.03583400
H	7.76066600	1.28836100	0.31979900
C	6.70564200	-0.01765700	-1.02283800
H	5.79118400	-0.01776100	-1.63997000
H	6.55900800	-0.81340300	-0.27179400
C	7.90429100	-0.35797500	-1.90076500
H	7.77818000	-1.33165200	-2.39744000
H	8.83205200	-0.40171400	-1.30822900
H	8.05002200	0.40125900	-2.68551200

VIII

C	-1.09124300	-2.10936900	1.77920400
C	-2.49105800	-2.33702500	2.33895800
C	-3.43078800	-1.21275400	1.91629700
C	-3.53741400	-1.08802100	0.38165400
C	-2.09353300	-0.95185800	-0.22146500
C	-2.09243000	-0.79633500	-1.74325400
C	-0.70926900	-0.64452600	-2.29253000
C	0.40326800	-0.93839100	-1.60049800
C	0.35442200	-1.45115100	-0.17400900
C	-1.04219500	-2.00357700	0.24114100
C	1.51519800	-2.43030400	0.04513300
C	2.86205700	-1.77864200	-0.24670700
C	2.88556300	-1.08599600	-1.58675700
C	1.73303300	-0.73427100	-2.18411300
C	4.22618400	-0.84208200	-2.24950700
C	5.19929500	-0.09390600	-1.33302700
C	4.84238500	-2.15248100	-2.75793200
C	-4.25364400	0.21501300	0.00915200
C	-4.39348900	-2.23121100	-0.18111700
C	-1.25812100	-3.38317600	-0.40209600

O	-5.12916100	0.30490000	-0.81325100
O	-3.76528900	1.27487900	0.66701600
H	-0.70044700	-1.16815000	2.20459500
H	-0.41409300	-2.90734400	2.12232700
H	-2.88076700	-3.31538900	2.01369700
H	-2.44516100	-2.38170200	3.43832800
H	-3.05246100	-0.26396900	2.32576000
H	-4.43708400	-1.36393300	2.34010300
H	-1.71956400	0.00282000	0.18829000
H	-2.69856900	0.07614200	-2.03759500
H	-2.58591000	-1.65784800	-2.22752800
H	-0.61258600	-0.27805100	-3.32038600
H	0.55393300	-0.57262400	0.47172700
H	1.39531800	-3.29590000	-0.62562700
H	1.50573700	-2.81509500	1.07528800
H	3.66138800	-2.53588400	-0.19865000
H	3.10495700	-1.04957100	0.54600400
H	1.76587400	-0.25137000	-3.16716600
H	4.03851700	-0.20439100	-3.12971700
H	6.15048500	0.09858600	-1.85260200
H	5.43097300	-0.67670700	-0.42689200
H	4.78382500	0.87267300	-1.01446000
H	5.77978900	-1.95533500	-3.30091200
H	5.08162700	-2.83655800	-1.92816400
H	4.15419300	-2.67414700	-3.43928400
H	-4.39255800	-2.25367700	-1.27794500
H	-4.06308900	-3.20880400	0.18758900
H	-5.43774400	-2.09488500	0.13289500
H	-2.27600500	-3.75944900	-0.25617400
H	-0.57595200	-4.12568600	0.03656000
H	-1.06545600	-3.35674900	-1.48512100
C	-4.16242000	2.56969200	0.21217100
H	-4.03538700	3.23108500	1.08078500
H	-5.22209100	2.54583200	-0.07606400
C	-3.28166000	3.00599300	-0.94795900
H	-3.41180800	2.28152400	-1.76931600
H	-3.65452700	3.96979200	-1.33304900
C	-1.80855400	3.11560300	-0.56225000
H	-1.54894600	2.25500500	0.07412400
H	-1.66138900	4.01322500	0.06576200
C	-0.85752000	3.15703600	-1.75501300
H	-1.21821500	3.89727200	-2.49007400
H	-0.88724200	2.17645800	-2.26236200
C	0.58708600	3.48887500	-1.37957200
H	0.63711000	4.53185800	-1.01667300
H	1.21245300	3.45479300	-2.28829000
C	1.19114700	2.56856800	-0.32154300
H	0.60408200	2.64921300	0.60776400
H	1.10149800	1.51970700	-0.65425200
C	2.65450400	2.87861400	-0.02160500
H	3.24025200	2.75562700	-0.94938900
H	2.75355800	3.94297000	0.25997100
C	3.26823000	2.00764900	1.07349400
H	4.35337800	2.20553900	1.12770000
H	3.17037300	0.95138600	0.77385600
C	2.66389300	2.20518800	2.46286600
H	2.64465700	3.28397100	2.69613200
H	1.60874200	1.88350200	2.46198800
C	3.41943100	1.47743500	3.57786300
H	2.92138400	1.68831600	4.54025300

H	4.43369600	1.90512600	3.66046600
C	3.54013300	-0.03956100	3.40596000
H	4.10533300	-0.44485300	4.26128700
H	4.15152900	-0.26577000	2.51509500
C	2.20162700	-0.76175900	3.29767300
H	2.33688800	-1.85238900	3.24183500
H	1.64745100	-0.45677200	2.39694800
H	1.55894100	-0.54728000	4.16675400

IX

C	-0.71515000	-2.27325300	1.19578400
C	-2.02108700	-2.78053700	1.79806300
C	-3.12683700	-1.74020500	1.65133200
C	-3.38797100	-1.37203200	0.17467700
C	-2.03594200	-0.94086300	-0.48693400
C	-2.19631800	-0.52307000	-1.94887200
C	-0.90211600	-0.06715300	-2.54418000
C	0.29564300	-0.30349300	-1.98435100
C	0.44217000	-1.06114300	-0.67803700
C	-0.81814800	-1.89138200	-0.29555500
C	1.75448000	-1.85323200	-0.69913200
C	2.94741800	-0.92956300	-0.92272700
C	2.76418100	-0.05409300	-2.13844800
C	1.52728900	0.19600700	-2.60338500
C	3.99329400	0.50583500	-2.82401700
C	4.85689600	1.33285500	-1.86635800
C	4.81676300	-0.59855600	-3.49941900
C	-4.29349600	-0.13549200	0.10202200
C	-4.14127800	-2.50874600	-0.52810700
C	-0.89321200	-3.15196300	-1.17075900
O	-5.28503400	-0.04861800	-0.57599900
O	-3.84203100	0.87439800	0.85923500
H	-0.40047900	-1.37699200	1.76135300
H	0.07962600	-3.02278800	1.33634300
H	-2.31639500	-3.73202500	1.32667900
H	-1.87084600	-3.00732800	2.86519700
H	-2.83093700	-0.83175800	2.19800400
H	-4.06328900	-2.09795600	2.10983100
H	-1.74957800	-0.03005800	0.06202100
H	-2.94137800	0.28627100	-2.03215100
H	-2.60942000	-1.34956700	-2.55458500
H	-0.94659200	0.48469200	-3.48933500
H	0.55823400	-0.29249200	0.10861200
H	1.72858300	-2.59662400	-1.51217500
H	1.88384500	-2.40512800	0.24239000
H	3.87112900	-1.52334100	-1.01583100
H	3.09525500	-0.29073600	-0.03461900
H	1.40708000	0.81862400	-3.49691700
H	3.63686400	1.18180600	-3.61936100
H	5.72261600	1.76225100	-2.39371600
H	5.24655800	0.71690300	-1.03962800
H	4.28017700	2.16152400	-1.42874300
H	5.65838200	-0.16533100	-4.06186100
H	5.24126900	-1.29796500	-2.76174300
H	4.19891000	-1.17912400	-4.20037600
H	-4.26083600	-2.32600700	-1.60336300
H	-3.63991500	-3.47391500	-0.39370800
H	-5.15220600	-2.59653800	-0.10628200
H	-1.82041600	-3.71515400	-1.01714200

H	-0.06148300	-3.83300900	-0.93867200
H	-0.82720700	-2.90351800	-2.24061800
C	-4.51956200	2.12876200	0.76579300
H	-4.41268200	2.59151800	1.75787200
H	-5.58332400	1.93734900	0.57038300
C	-3.93257200	3.02401300	-0.31908100
H	-3.86108600	2.44045100	-1.25240100
H	-4.67293600	3.81630200	-0.51772800
C	-2.59022600	3.67597500	0.01828200
H	-2.71233000	4.28316800	0.93399900
H	-2.33499100	4.39148000	-0.78212200
C	-1.42695400	2.70780300	0.21432600
H	-1.69558700	1.97037900	0.98749500
H	-1.26563500	2.12705900	-0.71158200
C	-0.12274800	3.38203200	0.62610700
H	-0.29314100	3.95796100	1.55406500
H	0.18311000	4.12045300	-0.13637600
C	1.00413000	2.37976400	0.85923500
H	0.60901800	1.56007100	1.48413900
H	1.29266500	1.91654300	-0.10195300
C	2.23651400	2.97629000	1.53304900
H	2.59400000	3.83559000	0.93963500
H	1.94331300	3.38878700	2.51554000
C	3.38907700	1.99033200	1.72392100
H	4.23426400	2.51329400	2.20342600
H	3.75886600	1.66964000	0.73546300
C	3.04203800	0.75086000	2.54610100
H	2.60981000	1.05904300	3.51569400
H	2.25069300	0.17827700	2.03300400
C	4.24425500	-0.15744600	2.79373300
H	5.03031700	0.42471800	3.30451000
H	4.67682000	-0.45813900	1.82136500
C	3.93519000	-1.40987900	3.61595600
H	3.48873000	-1.10778500	4.57950000
H	4.88352200	-1.91271100	3.86764500
C	3.01533800	-2.40786900	2.91828300
H	2.85283200	-3.30293100	3.53714900
H	3.45019200	-2.73857200	1.96103500
H	2.02617600	-1.97624600	2.70094900

X

C	-0.71542100	-2.27404200	1.19396100
C	-2.02130500	-2.78176300	1.79596400
C	-3.12706400	-1.74130900	1.65012200
C	-3.38827700	-1.37190500	0.17377100
C	-2.03629800	-0.94028800	-0.48760900
C	-2.19678400	-0.52133300	-1.94921100
C	-0.90260100	-0.06510700	-2.54432100
C	0.29518800	-0.30192000	-1.98475900
C	0.44180000	-1.06053800	-0.67902200
C	-0.81854300	-1.89101600	-0.29707100
C	1.75413400	-1.85258700	-0.70077400
C	2.94701900	-0.92869400	-0.92369200
C	2.76370900	-0.05228600	-2.13873800
C	1.52679300	0.19811400	-2.60344600
C	3.99276100	0.50826100	-2.82392800
C	4.81597700	-0.59549700	-3.50068400
C	4.85661200	1.33419900	-1.86557500

C	-4.29369500	-0.13522500	0.10228100
C	-4.14174800	-2.50795200	-0.52988900
C	-0.89382500	-3.15093600	-1.17319100
O	-5.28565400	-0.04787700	-0.57505300
O	-3.84151600	0.87424000	0.85966000
H	-0.40065300	-1.37824400	1.76020600
H	0.07932500	-3.02373200	1.33384300
H	-2.31667000	-3.73289200	1.32390100
H	-1.87093600	-3.00936800	2.86291000
H	-2.83112900	-0.83328700	2.19748000
H	-4.06349600	-2.09941700	2.10837700
H	-1.74981900	-0.02992100	0.06199900
H	-2.94173300	0.28818400	-2.03176500
H	-2.61008800	-1.34730700	-2.55549300
H	-0.94710600	0.48747900	-3.48904100
H	0.55793800	-0.29246400	0.10818000
H	1.72825200	-2.59532100	-1.51441500
H	1.88354300	-2.40524600	0.24030000
H	3.87076200	-1.52235400	-1.01726600
H	3.09483800	-0.29055700	-0.03508900
H	1.40653400	0.82139600	-3.49650600
H	3.63624800	1.18510700	-3.61849300
H	5.65775700	-0.16181300	-4.06252800
H	5.24023200	-1.29596200	-2.76386800
H	4.19799400	-1.17500900	-4.20239700
H	5.72211200	1.76427900	-2.39273200
H	5.24660100	0.71731100	-1.03969700
H	4.27998800	2.16231500	-1.42679400
H	-4.26150700	-2.32418900	-1.60494800
H	-3.64035800	-3.47324400	-0.39650800
H	-5.15259000	-2.59612200	-0.10794100
H	-1.82108700	-3.71413400	-1.01990300
H	-0.06217600	-3.83225600	-0.94164400
H	-0.82786500	-2.90172200	-2.24287500
C	-4.51888900	2.12877200	0.76746900
H	-4.41205200	2.59050400	1.76003000
H	-5.58266000	1.93777100	0.57172500
C	-3.93164700	3.02500800	-0.31645400
H	-3.86033100	2.44241800	-1.25040400
H	-4.67180000	3.81771800	-0.51423200
C	-2.58914600	3.67629600	0.02155900
H	-2.71107000	4.28245400	0.93799300
H	-2.33388400	4.39269100	-0.77803300
C	-1.42597500	2.70774500	0.21640100
H	-1.69463300	1.96952100	0.98879600
H	-1.26480500	2.12798100	-0.71015000
C	-0.12166700	3.38139400	0.62881900
H	-0.29198900	3.95632100	1.55741200
H	0.18418700	4.12063400	-0.13286900
C	1.00514800	2.37879600	0.86083400
H	0.60994600	1.55841500	1.48477100
H	1.29371300	1.91665600	-0.10086900
C	2.23752800	2.97446100	1.53541600
H	2.59526100	3.83427500	0.94289900
H	1.94424500	3.38606300	2.51825800
C	3.38988500	1.98812000	1.72556600
H	4.23507500	2.51047400	2.20572200
H	3.75982200	1.66828400	0.73688800
C	3.04247800	0.74797700	2.54656900
H	2.60996900	1.05538100	3.51628700

H	2.25124300	0.17592700	2.03271000
C	4.24451800	-0.16068200	2.79380800
H	5.03056200	0.42097300	3.30519200
H	4.67725400	-0.46072600	1.82132000
C	3.93504200	-1.41365800	3.61503900
H	3.48847600	-1.11216400	4.57872200
H	4.88320800	-1.91689300	3.86653700
C	3.01505000	-2.41089200	2.91647100
H	2.85211000	-3.30632500	3.53468700
H	3.44997100	-2.74107400	1.95907600
H	2.02607600	-1.97878100	2.69926400

XI

C	-0.71611700	2.27567300	-1.18944200
C	-2.02184300	2.78416500	-1.79112200
C	-3.12745400	1.74321000	-1.64762300
C	-3.38914000	1.37108600	-0.17201500
C	-2.03734800	0.93874200	0.48922000
C	-2.19841900	0.51721400	1.95000100
C	-0.90443300	0.05988400	2.54471200
C	0.29353400	0.29778500	1.98597800
C	0.44054500	1.05887500	0.68174500
C	-0.81968700	1.89000000	0.30086100
C	1.75284000	1.85092200	0.70532000
C	2.94581000	0.92687400	0.92714800
C	2.76203200	0.04785800	2.14020600
C	1.52497200	-0.20351000	2.60400900
C	3.99086100	-0.51435200	2.82439700
C	4.81429100	0.58785800	3.50342000
C	4.85458000	-1.33859600	1.86447400
C	-4.29427800	0.13401000	-0.10316000
C	-4.14340100	2.50562400	0.53323900
C	-0.89529600	3.14831800	1.17926000
O	-5.28620400	0.04489700	0.57393500
O	-3.84166800	-0.87373100	-0.86261500
H	-0.40096800	1.38088000	-1.75708300
H	0.07853600	3.02576700	-1.32775000
H	-2.31762300	3.73444300	-1.31757400
H	-1.87106400	3.01358500	-2.85763100
H	-2.83111800	0.83631500	-2.19662300
H	-4.06380900	2.10202700	-2.10549700
H	-1.75051900	0.02939000	-0.06185800
H	-2.94341100	-0.29244200	2.03085000
H	-2.61194200	1.34213300	2.55759500
H	-0.94922700	-0.49450400	3.48837700
H	0.55696500	0.29229200	-0.10684900
H	1.72659600	2.59241800	1.52007200
H	1.88253800	2.40500700	-0.23486800
H	3.86928600	1.52066400	1.02264900
H	3.09459100	0.29062300	0.03734900
H	1.40444400	-0.82885800	3.49560000
H	3.63406000	-1.19268600	3.61757000
H	5.65583700	0.15286800	4.06461900
H	5.23889800	1.28957300	2.76798400
H	4.19633200	1.16624400	4.20609300
H	5.71952700	-1.77056800	2.39100800
H	5.24541600	-0.71999300	1.04026800
H	4.27755500	-2.16524100	1.42341800
H	-4.26411600	2.31970200	1.60782300

H	-3.64206000	3.47127000	0.40220900
H	-5.15388000	2.59446900	0.11055000
H	-1.82257000	3.71172200	1.02682500
H	-0.06360800	3.83009200	0.94921400
H	-0.82959900	2.89707200	2.24849400
C	-4.51695500	-2.12942500	-0.77136600
H	-4.40994700	-2.59005800	-1.76444700
H	-5.58093000	-1.94003400	-0.57515200
C	-3.92796800	-3.02602300	0.31130200
H	-3.85602400	-2.44415500	1.24565600
H	-4.66748900	-3.81930300	0.50916700
C	-2.58547200	-3.67635400	-0.02852000
H	-2.70768400	-4.28089700	-0.94597800
H	-2.32962800	-4.39413600	0.76966000
C	-1.42265100	-2.70715700	-0.22206900
H	-1.69195200	-1.96739600	-0.99280300
H	-1.26112600	-2.12925200	0.70559500
C	-0.11838400	-3.37969300	-0.63647100
H	-0.28889400	-3.95267500	-1.56623000
H	0.18789500	-4.12048800	0.12354300
C	1.00815500	-2.37636900	-0.86668400
H	0.61252100	-1.55466300	-1.48861800
H	1.29694900	-1.91635200	0.09597000
C	2.24053000	-2.97006200	-1.54298600
H	2.59876000	-3.83107900	-0.95250600
H	1.94716100	-3.37953100	-2.52670000
C	3.39236300	-1.98270800	-1.73115500
H	4.23761700	-2.50336000	-2.21305100
H	3.76256000	-1.66528300	-0.74178800
C	3.04398200	-0.74057400	-2.54872200
H	2.61157900	-1.04569200	-3.51921400
H	2.25235100	-0.17046000	-2.03328500
C	4.24533700	0.16961600	-2.79361700
H	5.03171700	-0.41006500	-3.30673000
H	4.67798300	0.46721600	-1.82032000
C	3.93490200	1.42466300	-3.61134200
H	3.48867900	1.12547700	-4.57591400
H	4.88266400	1.92940300	-3.86135500
C	3.01395200	2.41917000	-2.91015400
H	2.85038700	3.31622600	-3.52586100
H	3.44841200	2.74701300	-1.95172800
H	2.02530300	1.98559800	-2.69436900

XII

C	-0.97272400	-2.37352400	-0.49752800
C	-2.35291300	-2.98926200	-0.30038600
C	-3.32385000	-1.96135200	0.27001200
C	-3.47792400	-0.73073600	-0.64803400
C	-2.05831300	-0.14321300	-0.96598600
C	-2.10344300	1.08080000	-1.88242200
C	-0.74267000	1.67568300	-2.06502800
C	0.39312100	1.01692200	-1.78198700
C	0.39566200	-0.40298800	-1.24863900
C	-0.96027200	-1.14454200	-1.42899400
C	1.61822900	-1.14252200	-1.80417700
C	2.91066600	-0.43364400	-1.41318800
C	2.87638600	1.04255600	-1.72071000
C	1.70080200	1.66609100	-1.91542700
C	4.18934900	1.79465900	-1.78632300

C	4.98460600	1.64833400	-0.48365900
C	5.03140200	1.38122500	-2.99962600
C	-4.19439000	0.40100600	0.09706600
C	-4.33689100	-1.08001000	-1.86902700
C	-1.12558700	-1.58093300	-2.89317800
O	-5.07466800	1.07918900	-0.36622600
O	-3.67734500	0.60744900	1.31697200
H	-0.58871900	-2.06547800	0.49161300
H	-0.27301900	-3.13544600	-0.87545300
H	-2.73100700	-3.39884100	-1.25141400
H	-2.27743600	-3.84488500	0.38887000
H	-2.94929700	-1.63051200	1.25041400
H	-4.31461800	-2.41225100	0.44304500
H	-1.69457800	0.21721200	0.01134800
H	-2.78746400	1.84238400	-1.47459100
H	-2.53106000	0.81957000	-2.86695300
H	-0.68173500	2.70119800	-2.44501600
H	0.55603800	-0.31083100	-0.15807200
H	1.55528500	-1.18838000	-2.90335300
H	1.64252500	-2.18000300	-1.43852600
H	3.76741500	-0.90564100	-1.92101900
H	3.09634300	-0.57119800	-0.33434000
H	1.69493400	2.73487700	-2.15658000
H	3.94053700	2.86240300	-1.90658800
H	5.88855000	2.27615600	-0.50589900
H	5.31114500	0.60757100	-0.32667600
H	4.38156000	1.94825400	0.38720400
H	5.94385000	1.99401500	-3.06502500
H	5.34817100	0.32816800	-2.93464900
H	4.46598000	1.50818600	-3.93468200
H	-4.40257200	-0.24821200	-2.58122400
H	-3.95457600	-1.96182900	-2.39656100
H	-5.36329900	-1.30469400	-1.54552800
H	-2.12397900	-1.98399000	-3.09907600
H	-0.40133000	-2.36955400	-3.14452600
H	-0.95408000	-0.74397900	-3.58639400
C	-3.93491300	1.85795400	1.95464700
H	-3.99859500	1.63938400	3.03032000
H	-4.89935400	2.25143000	1.60680100
C	-2.79344700	2.81790400	1.65420200
H	-2.73327600	2.95609600	0.56093500
H	-3.04331900	3.80666100	2.07461000
C	-1.45087200	2.33375100	2.19567400
H	-1.32505000	1.27458800	1.92012600
H	-1.47938600	2.35712100	3.30019400
C	-0.25102100	3.13992900	1.70213600
H	-0.43980400	4.21257200	1.88049000
H	-0.15825000	3.01736700	0.60772300
C	1.06374400	2.74297800	2.37654600
H	0.93155100	2.79778500	3.47267500
H	1.84380200	3.48335200	2.13053900
C	1.56746300	1.35045500	1.99956300
H	0.72816600	0.63645400	2.03052900
H	1.91347000	1.36381700	0.95073400
C	2.67974800	0.84254600	2.91497600
H	3.46218600	1.61654300	3.00387000
H	2.26790800	0.71474700	3.93238100
C	3.32889900	-0.46604300	2.46226900
H	4.05154000	-0.79521300	3.22850400
H	3.92673200	-0.27611000	1.55384100

C	2.34709000	-1.60082300	2.17944600
H	1.68830500	-1.73915100	3.05449700
H	1.68149900	-1.31633700	1.34623400
C	3.03230800	-2.91783300	1.82148900
H	3.55556600	-3.31229600	2.71118500
H	3.81992200	-2.71653200	1.07394600
C	2.09057500	-3.98489100	1.26080200
H	2.67713600	-4.88674500	1.01977000
H	1.68033000	-3.62594200	0.30144200
C	0.93645500	-4.36404400	2.18326400
H	0.33475400	-5.17833700	1.75217100
H	0.25854100	-3.51435600	2.35825700
H	1.30451900	-4.70288000	3.16527800

XIII

C	-3.29434700	-2.54989300	1.09854500
C	-4.80578100	-2.35097000	1.05904200
C	-5.15342000	-0.86694100	1.02597900
C	-4.55070300	-0.15556600	-0.20525400
C	-3.01258100	-0.44256200	-0.26188300
C	-2.32830100	0.22363000	-1.45656900
C	-0.85171500	-0.01576300	-1.45902300
C	-0.24982100	-0.94233400	-0.69596400
C	-1.03067500	-1.84211600	0.24624700
C	-2.54866200	-1.91840900	-0.09534800
C	-0.32102300	-3.19626400	0.35735100
C	1.11511800	-3.02281400	0.84199000
C	1.87496100	-2.01462500	0.01513500
C	1.20851700	-1.09065300	-0.69918100
C	3.38669900	-2.08261000	0.00367400
C	3.89913800	-3.29696300	-0.78028000
C	3.96883600	-2.05326800	1.42019500
C	-4.67454300	1.36261400	-0.02234400
C	-5.33946500	-0.52126400	-1.46760400
C	-2.75839800	-2.76416900	-1.36095600
O	-5.18285100	2.11665200	-0.81171100
O	-4.10606800	1.77865500	1.11791000
H	-2.91232700	-2.09453200	2.02984600
H	-3.05951900	-3.62425200	1.15908500
H	-5.24007100	-2.87162400	0.19001800
H	-5.26192300	-2.81497400	1.94751300
H	-4.76205500	-0.39247600	1.93915700
H	-6.24574900	-0.71992700	1.03223800
H	-2.62217000	0.05721400	0.63923200
H	-2.52060300	1.31014100	-1.44692400
H	-2.76091500	-0.13201800	-2.40864800
H	-0.23797700	0.60667600	-2.11937000
H	-0.96031300	-1.37288300	1.24757300
H	-0.30160100	-3.68741200	-0.62910700
H	-0.86675500	-3.86337700	1.04098000
H	1.63744600	-3.99339900	0.82576800
H	1.11902900	-2.70243600	1.90026800
H	1.77271900	-0.37463000	-1.30741500
H	3.74247400	-1.18206900	-0.52194600
H	4.99890600	-3.28164300	-0.83859300
H	3.50149300	-3.30114800	-1.80585000
H	3.60840700	-4.24473800	-0.29942700
H	5.06922300	-2.04548600	1.38760200
H	3.66370900	-2.93838100	2.00096200

H	3.63747300	-1.15732100	1.96783600
H	-4.90902200	-0.06989400	-2.37037500
H	-5.39256000	-1.60643700	-1.61385000
H	-6.36767000	-0.14266400	-1.38295800
H	-3.80056400	-2.75253500	-1.70136600
H	-2.49381900	-3.81479500	-1.17121500
H	-2.13110500	-2.41158000	-2.19294600
C	-3.90493000	3.18129400	1.28901800
H	-3.77998000	3.31883800	2.37219900
H	-4.80160400	3.72275200	0.95576600
C	-2.67328800	3.64733600	0.52699900
H	-2.85959600	3.50222200	-0.55026200
H	-2.57118400	4.73622000	0.67147500
C	-1.38622800	2.93553100	0.93912800
H	-1.56362000	1.84830300	0.97119500
H	-1.11421600	3.22880700	1.96880700
C	-0.21692900	3.20201800	-0.00162500
H	-0.49575000	2.86471500	-1.01600500
H	-0.03953300	4.28960900	-0.08423400
C	1.07262900	2.50377800	0.41951100
H	0.86486600	1.43476000	0.60121500
H	1.41595700	2.91900700	1.38411000
C	2.18801600	2.62569800	-0.61318800
H	1.87092300	2.12300700	-1.54556400
H	2.32755200	3.68859900	-0.88160500
C	3.52336800	2.04635000	-0.15592200
H	3.86430500	2.58850600	0.74488200
H	3.36982800	1.00037500	0.16177100
C	4.60787900	2.12168300	-1.23065300
H	4.62078800	3.14397200	-1.64643200
H	4.33176600	1.45918500	-2.07152200
C	6.01620800	1.77244900	-0.74760400
H	6.73401700	1.95513600	-1.56629800
H	6.29490800	2.46948100	0.06186800
C	6.17212600	0.33185500	-0.26601400
H	5.83128700	-0.34623600	-1.06821500
H	5.49678800	0.15533800	0.58794700
C	7.59284000	-0.06100300	0.14167300
H	8.27278900	0.08937100	-0.71527100
H	7.61040800	-1.14350500	0.35329400
C	8.12817600	0.69156300	1.35657800
H	9.12292900	0.32273900	1.64819100
H	7.45847800	0.56854900	2.22322900
H	8.22286000	1.77073000	1.16185500

XIV

C	-1.66302800	-1.60639100	2.32642000
C	-3.02009500	-1.50124400	3.01410300
C	-3.84758000	-0.37313700	2.40761900
C	-4.09085700	-0.57781900	0.89643800
C	-2.71495300	-0.79441200	0.18333900
C	-2.85524700	-0.98936800	-1.32649000
C	-1.52611000	-1.14375000	-1.99488600
C	-0.38765800	-1.40195100	-1.33057500
C	-0.35009700	-1.55667800	0.17915700
C	-1.74750800	-1.84405500	0.80439000
C	0.74847500	-2.55789500	0.55698200
C	2.10631700	-2.11277000	0.02116400
C	2.06162800	-1.80946800	-1.45684300

C	0.88965700	-1.50587300	-2.04235400
C	3.34803500	-1.86833700	-2.25500900
C	4.39754200	-0.88994800	-1.71757300
C	3.90756500	-3.29432000	-2.33500600
C	-4.67025600	0.70928600	0.29550900
C	-5.13600900	-1.67790600	0.67368100
C	-2.16063100	-3.29447500	0.51001500
O	-5.67248700	0.76808900	-0.36978800
O	-3.91560600	1.78397700	0.56584900
H	-1.11553600	-0.66260000	2.50058500
H	-1.06571800	-2.40338100	2.79667200
H	-3.56021900	-2.45974600	2.94789100
H	-2.87444100	-1.31162500	4.08911500
H	-3.31168000	0.57694200	2.55631700
H	-4.81700000	-0.27755400	2.92356200
H	-2.19080800	0.16428700	0.32243600
H	-3.38989600	-0.13248400	-1.77095700
H	-3.48678600	-1.86619100	-1.55627200
H	-1.49269800	-1.04086000	-3.08487800
H	-0.02712000	-0.57640600	0.57949200
H	0.51181900	-3.54749400	0.13369700
H	0.80213100	-2.67708100	1.64930600
H	2.86330400	-2.88604900	0.22789500
H	2.44477300	-1.21436900	0.56797500
H	0.86581100	-1.30433500	-3.11902100
H	3.09938500	-1.55582800	-3.28312000
H	5.30684400	-0.91205800	-2.33787500
H	4.69530600	-1.14185700	-0.68680200
H	4.01129200	0.14000600	-1.71459100
H	4.78642900	-3.32793400	-2.99735000
H	4.22853000	-3.66227400	-1.34760200
H	3.15493800	-3.99311500	-2.72940000
H	-5.28078200	-1.90376000	-0.39026300
H	-4.86754500	-2.60560400	1.19170700
H	-6.10865700	-1.34660900	1.06313600
H	-3.18549100	-3.51124900	0.83199800
H	-1.50239500	-3.99882100	1.03936900
H	-2.09298500	-3.52351800	-0.56395000
C	-4.28240400	3.01825100	-0.05235200
H	-3.92758000	3.80229700	0.63251000
H	-5.37793700	3.06562400	-0.11624700
C	-3.66187100	3.17704900	-1.43521500
H	-3.83844100	2.24860100	-2.00354600
H	-4.23341600	3.95993300	-1.96021100
C	-2.18021500	3.55543600	-1.45551700
H	-2.05792900	4.53304300	-0.95414000
H	-1.87686800	3.71953300	-2.50387600
C	-1.22687100	2.54913700	-0.81521600
H	-1.50444400	2.39929500	0.24149100
H	-1.34232300	1.56383700	-1.29976500
C	0.23518500	2.97875600	-0.88872200
H	0.33522700	3.99905600	-0.47537400
H	0.54849700	3.05008400	-1.94577600
C	1.17940000	2.04413900	-0.13965600
H	0.84475600	1.96407500	0.91101200
H	1.10202200	1.02769100	-0.56396500
C	2.63982900	2.48572000	-0.16893200
H	3.01719300	2.42292900	-1.20580100
H	2.71246500	3.55182100	0.11199300
C	3.53525600	1.66075500	0.74957800

H	3.37346500	0.59054000	0.53992300
H	3.22027500	1.81128000	1.79771300
C	5.02221300	1.96815100	0.61429500
H	5.32833300	1.81984800	-0.43699700
H	5.20091500	3.03657800	0.83158700
C	5.90319500	1.10989300	1.51673200
H	5.61114400	1.26669700	2.57087800
H	5.70490100	0.04283400	1.30690100
C	7.39675000	1.38239500	1.35934200
H	7.59373000	2.44883700	1.56572700
H	7.68270000	1.22257300	0.30506400
C	8.26808600	0.51666700	2.26242600
H	9.33755300	0.73497200	2.12434100
H	8.11579100	-0.55425500	2.05326100
H	8.02746000	0.68228500	3.32466200

XV

C	1.07248400	-2.40469500	-1.52557600
C	2.43523600	-2.79412200	-2.08784200
C	3.44723200	-1.67340100	-1.87686600
C	3.62364200	-1.32077700	-0.38343300
C	2.21910600	-1.01210000	0.23622300
C	2.29332000	-0.62798700	1.71431200
C	0.94491900	-0.30644600	2.27825800
C	-0.21056100	-0.62783700	1.67332700
C	-0.24823200	-1.34444300	0.33622900
C	1.09105500	-2.05342700	-0.02345300
C	-1.48536300	-2.24958700	0.28337800
C	-2.76016500	-1.44720900	0.50780200
C	-2.69252700	-0.58305400	1.74124900
C	-1.49944800	-0.24663600	2.26175100
C	-4.02077800	-0.15797300	2.33563600
C	-4.63296200	-1.29910000	3.16154400
C	-3.95983100	1.13117500	3.15233000
C	4.43556300	-0.02478200	-0.26445900
C	4.43992500	-2.41230600	0.32138700
C	1.24235800	-3.32682000	0.82426000
O	5.42198400	0.10890200	0.41403300
O	3.91889700	0.97420000	-0.99289100
H	0.70887200	-1.52305300	-2.08355600
H	0.34639300	-3.21153500	-1.71210600
H	2.78814000	-3.73039400	-1.62573500
H	2.34279000	-3.00616700	-3.16455800
H	3.09974700	-0.77816000	-2.41494800
H	4.42603700	-1.94414800	-2.30566100
H	1.88140500	-0.10955500	-0.29936000
H	2.96106000	0.24079200	1.84581100
H	2.75737700	-1.43403700	2.31033700
H	0.91194300	0.21616600	3.24041500
H	-0.40416100	-0.55573100	-0.42618900
H	-1.41372800	-3.02065000	1.06718700
H	-1.54057500	-2.77324500	-0.68262500
H	-3.62963300	-2.12251500	0.56987800
H	-2.95469400	-0.79772900	-0.36313300
H	-1.45036900	0.37170000	3.16258700
H	-4.69662800	0.02287600	1.47850700
H	-5.64425700	-1.03533000	3.50787900
H	-4.01001600	-1.50598300	4.04584900
H	-4.71022100	-2.23050000	2.58094800

H	-4.97134100	1.44440100	3.45199800
H	-3.37395200	0.99720100	4.07527300
H	-3.50250300	1.95031200	2.57706100
H	4.50777700	-2.24836500	1.40421000
H	4.02290200	-3.41042800	0.14709400
H	5.46846000	-2.40873300	-0.06488900
H	2.22208300	-3.80045300	0.69913500
H	0.48690400	-4.07321800	0.53878900
H	1.10888400	-3.11560900	1.89578600
C	4.55765200	2.24881600	-0.89929900
H	4.32627800	2.75582900	-1.84743200
H	5.64287000	2.09139900	-0.83172800
C	4.06609800	3.06288500	0.29051500
H	4.10125100	2.42292400	1.18764400
H	4.80671400	3.86052900	0.46523700
C	2.68637700	3.70028600	0.12781700
H	2.71514900	4.39859200	-0.72865300
H	2.48569700	4.32615700	1.01442000
C	1.52436100	2.72878900	-0.06426600
H	1.69121900	2.12532400	-0.97198400
H	1.49581900	2.00910000	0.77258100
C	0.17446200	3.42980600	-0.17051500
H	0.21232200	4.17103200	-0.98979200
H	-0.01220900	4.01069200	0.75080400
C	-0.99019100	2.47512400	-0.41032000
H	-0.77475300	1.87439900	-1.31144300
H	-1.05626700	1.75666900	0.42531700
C	-2.33040100	3.18943500	-0.56972600
H	-2.48949100	3.84244800	0.30598800
H	-2.28323000	3.86417100	-1.44386900
C	-3.53233300	2.25717400	-0.71082200
H	-4.45729100	2.85897600	-0.72912700
H	-3.59986500	1.62353000	0.18994300
C	-3.50446800	1.35599200	-1.94230500
H	-3.37315800	1.97025700	-2.85130500
H	-2.61968700	0.69648500	-1.89979800
C	-4.75716100	0.49569700	-2.08417900
H	-4.95856900	-0.01413400	-1.12421400
H	-5.63168200	1.14661900	-2.26267500
C	-4.66160100	-0.55026100	-3.19108200
H	-3.79279100	-1.20267200	-2.99201500
H	-4.44556100	-0.04693700	-4.14950800
C	-5.91795900	-1.40242000	-3.32853800
H	-5.81571300	-2.14982900	-4.12939200
H	-6.79707100	-0.78074500	-3.56132000
H	-6.13458600	-1.94231400	-2.39282500

XVI

C	-0.87412800	-2.52188200	0.35514200
C	-2.16856800	-3.19891200	0.79131500
C	-3.24450600	-2.15869000	1.08312500
C	-3.55972600	-1.28030200	-0.14563500
C	-2.22582900	-0.67293600	-0.70599600
C	-2.43519400	0.19683000	-1.94681900
C	-1.15937900	0.84997700	-2.37609200
C	0.05222600	0.43669300	-1.96962000
C	0.23514700	-0.74324100	-1.03349000
C	-1.02619000	-1.64462600	-0.90424500
C	1.52297000	-1.48220900	-1.41367400

C	2.72756400	-0.55312700	-1.31958800
C	2.51494800	0.74394100	-2.05835600
C	1.27037800	1.14973000	-2.36646000
C	3.73005100	1.57326700	-2.41703700
C	4.56542400	1.90573200	-1.17504900
C	4.58633700	0.90341400	-3.49871500
C	-4.37936100	-0.05544200	0.27582700
C	-4.40427100	-2.06226300	-1.15834500
C	-1.16813200	-2.52483800	-2.15601100
O	-5.34081200	0.36485100	-0.31444300
O	-3.85732800	0.55338400	1.35066200
H	-0.52331100	-1.88234900	1.18535800
H	-0.08842700	-3.27664200	0.19251100
H	-2.50964500	-3.90946600	0.02066200
H	-1.98499400	-3.79957800	1.69583200
H	-2.89488700	-1.51280800	1.90249700
H	-4.17205700	-2.64145200	1.43160100
H	-1.88627300	0.00726800	0.09331200
H	-3.19796500	0.96796700	-1.75269600
H	-2.84677400	-0.40008000	-2.78033200
H	-1.22886300	1.70920400	-3.05185900
H	0.41104200	-0.30823300	-0.03105800
H	1.44369000	-1.86201500	-2.44502700
H	1.67554700	-2.35438500	-0.75992400
H	3.62960900	-1.06285500	-1.69582100
H	2.93851800	-0.33138400	-0.25970100
H	1.13288700	2.08644700	-2.91792800
H	3.35767200	2.52439600	-2.83313600
H	5.39002900	2.58866500	-1.43062400
H	5.01333700	0.99877000	-0.73748000
H	3.95082800	2.38685100	-0.39871000
H	5.42321500	1.55667600	-3.79064500
H	5.01942900	-0.04525000	-3.14381100
H	3.98992200	0.68842300	-4.39769400
H	-4.58849300	-1.48821300	-2.07480000
H	-3.93380900	-3.01426600	-1.43156100
H	-5.38713400	-2.28974800	-0.72132100
H	-2.11668900	-3.07399300	-2.17609800
H	-0.36204600	-3.27203100	-2.19576100
H	-1.10882700	-1.92799600	-3.07837300
C	-4.23162800	1.90660400	1.60568000
H	-4.22251600	2.01895000	2.69933800
H	-5.24970900	2.07965800	1.23210500
C	-3.22506400	2.83842600	0.94737300
H	-3.22543100	2.63936000	-0.13810800
H	-3.57451700	3.87776500	1.06757100
C	-1.81308100	2.68515900	1.50760000
H	-1.57267800	1.61144800	1.56251600
H	-1.79806100	3.05179500	2.55007200
C	-0.74138900	3.41198200	0.69721200
H	-1.05934300	4.45644400	0.53547700
H	-0.67026200	2.94939600	-0.30378100
C	0.63524100	3.40756100	1.36434200
H	0.53794300	3.82363800	2.38390100
H	1.30843500	4.09363900	0.82257200
C	1.29490400	2.03170200	1.44434700
H	0.56248000	1.29202100	1.80757100
H	1.57485600	1.70810000	0.42660400
C	2.51883400	2.00582800	2.35715300
H	3.20567400	2.82083300	2.06824900

H	2.19563500	2.23813900	3.38815800
C	3.29064000	0.68558900	2.35705100
H	4.11447900	0.75351000	3.08859700
H	3.77530700	0.55752000	1.37519300
C	2.44085000	-0.54390300	2.68106900
H	1.83978800	-0.33210500	3.58266600
H	1.71290800	-0.71994300	1.86894100
C	3.24287400	-1.82553000	2.90913200
H	3.96596900	-1.66229100	3.72900000
H	2.55951000	-2.61688100	3.26317000
C	3.98739100	-2.34033900	1.67826900
H	4.68813900	-1.57006900	1.31419400
H	3.26167300	-2.49718600	0.86189700
C	4.75063200	-3.63427600	1.93724200
H	5.27082500	-3.98546700	1.03357500
H	4.07188100	-4.43731400	2.26616100
H	5.50682600	-3.49827900	2.72670200

XVII

C	-1.77731900	-1.68422600	2.26420800
C	-3.17403700	-1.60305100	2.87096000
C	-3.96712100	-0.45716000	2.25190100
C	-4.11711400	-0.61434700	0.72296300
C	-2.69848600	-0.80246700	0.08920100
C	-2.74386300	-0.94416100	-1.43241400
C	-1.37582800	-1.07768700	-2.02216000
C	-0.28036200	-1.36147700	-1.29862800
C	-0.33524900	-1.56701700	0.20438100
C	-1.76837700	-1.87221600	0.73290600
C	0.73658800	-2.58462900	0.61420400
C	2.12577700	-2.12921500	0.17637000
C	2.17134800	-1.77606100	-1.29054400
C	1.03768000	-1.44698200	-1.93485300
C	3.50366000	-1.81327700	-2.01111100
C	4.51183000	-0.83966400	-1.39218100
C	4.07701300	-3.23389600	-2.08562700
C	-4.66830100	0.68809300	0.12872600
C	-5.14072200	-1.71128700	0.40386600
C	-2.16108400	-3.31197000	0.36656400
O	-5.62741300	0.76195800	-0.59607400
O	-3.94262300	1.75872300	0.48125100
H	-1.24417600	-0.74610800	2.50186200
H	-1.20729700	-2.49554600	2.74374900
H	-3.70584600	-2.55955500	2.74027100
H	-3.09488100	-1.44858700	3.95847300
H	-3.44475600	0.48859400	2.46307300
H	-4.96645800	-0.37993300	2.71066200
H	-2.18778600	0.15188900	0.29418200
H	-3.25040600	-0.07140700	-1.87893300
H	-3.35907800	-1.81135800	-1.73214300
H	-1.27557200	-0.93832000	-3.10397400
H	-0.03622000	-0.60190800	0.65687000
H	0.52314400	-3.55858600	0.14483000
H	0.72359300	-2.74012700	1.70321200
H	2.86586600	-2.91362200	0.40132500
H	2.43458900	-1.25229900	0.77322600
H	1.07939400	-1.20975700	-3.00368600
H	3.31509900	-1.48122100	-3.04585700
H	5.45884700	-0.84483100	-1.95376000

H	4.74648300	-1.10716000	-0.34912200
H	4.11867200	0.18755800	-1.39545500
H	4.99181100	-3.24936400	-2.69805100
H	4.34505300	-3.61807000	-1.08867500
H	3.35302800	-3.93020100	-2.53441600
H	-5.21508600	-1.91088500	-0.67251100
H	-4.90333900	-2.65096800	0.91539800
H	-6.13804200	-1.39221200	0.73700500
H	-3.20327800	-3.53856100	0.61817800
H	-1.53542900	-4.03370500	0.91173000
H	-2.02766000	-3.50561900	-0.70823000
C	-4.28774900	3.01107000	-0.11302600
H	-3.97680900	3.77316400	0.61649000
H	-5.37883500	3.05224100	-0.23308800
C	-3.59723000	3.22350300	-1.45485900
H	-3.73655400	2.31511800	-2.06442100
H	-4.14684600	4.02132200	-1.98078200
C	-2.11954600	3.61088900	-1.38313000
H	-2.03114600	4.57212900	-0.84439100
H	-1.76116000	3.81055100	-2.40767700
C	-1.19658000	2.58848000	-0.72454100
H	-1.52953600	2.40471300	0.31053500
H	-1.28157200	1.61830800	-1.24465000
C	0.26579000	3.02243700	-0.70572200
H	0.34099300	4.03087900	-0.25937200
H	0.63698000	3.12279600	-1.74146800
C	1.16694600	2.06634300	0.06842300
H	0.77938100	1.96467900	1.09883400
H	1.10513400	1.06018400	-0.38216500
C	2.63003100	2.49611200	0.12232900
H	3.05829400	2.45163400	-0.89554400
H	2.69795200	3.55499200	0.43023300
C	3.46899500	1.63982000	1.06513000
H	3.29028500	0.57678100	0.83291200
H	3.11561800	1.78299200	2.10202300
C	4.96765300	1.90796900	0.99073800
H	5.29996000	1.76039300	-0.05099000
H	5.17063300	2.96854500	1.22511300
C	5.78263300	1.01600200	1.92442200
H	5.52651500	-0.04118100	1.72613000
H	5.46987000	1.20978700	2.96487100
C	7.29844500	1.18968700	1.81331000
H	7.78067200	0.61671500	2.62250200
H	7.55754400	2.24724500	1.99656900
C	7.88199100	0.74494900	0.47451400
H	8.97880700	0.83189700	0.46849800
H	7.50102400	1.34985600	-0.36247300
H	7.62834200	-0.30668800	0.26375100

XVIII

C	3.38925900	2.53823200	0.86775900
C	4.88489900	2.35483200	0.63291200
C	5.24323500	0.87490500	0.55593300
C	4.48976000	0.15137900	-0.58123900
C	2.95279500	0.41672600	-0.42575000
C	2.11830900	-0.27396000	-1.50583400
C	0.65281000	-0.02735000	-1.33371800
C	0.14646100	0.91368500	-0.52038600
C	1.03792200	1.81502100	0.31688800

C	2.50102400	1.89145000	-0.21522800
C	0.35174200	3.17197600	0.51299800
C	-1.02485900	3.00891800	1.14997200
C	-1.88089400	2.01830800	0.39957200
C	-1.30237000	1.08322800	-0.37419900
C	-3.38636500	2.12686900	0.52162200
C	-3.84079700	2.17018800	1.98377000
C	-3.92923600	3.32850800	-0.26239100
C	4.65998500	-1.36491500	-0.42233400
C	5.09522100	0.52639300	-1.93924900
C	2.54316200	2.72456700	-1.50593600
O	5.02590500	-2.11398700	-1.29144800
O	4.31004700	-1.78704000	0.80023000
H	3.13596600	2.08545900	1.84325100
H	3.15338700	3.61066200	0.95234500
H	5.19691800	2.87940400	-0.28483700
H	5.44695200	2.82571900	1.45456900
H	4.98474800	0.39876400	1.51415800
H	6.32837800	0.74178000	0.41564000
H	2.69764600	-0.07409800	0.52774900
H	2.30871000	-1.36066400	-1.49482600
H	2.42720200	0.05654300	-2.51358600
H	-0.03519600	-0.65154200	-1.91454200
H	1.09432000	1.34767500	1.32004400
H	0.22725100	3.66886700	-0.46273400
H	0.97389700	3.83174200	1.13561200
H	-1.53254900	3.98612500	1.19905600
H	-0.91890100	2.67749200	2.19953100
H	-1.93578000	0.37979000	-0.92562100
H	-3.81303700	1.21938600	0.06410100
H	-4.93988600	2.18144700	2.04841500
H	-3.47294400	3.07430600	2.49446000
H	-3.47653800	1.29394900	2.54193400
H	-5.02897200	3.35864500	-0.21213500
H	-3.55403800	4.28144300	0.14416500
H	-3.63542200	3.27311900	-1.32086400
H	4.53943900	0.08801000	-2.77757200
H	5.13734500	1.61249000	-2.08054400
H	6.12220200	0.14056000	-2.00322000
H	3.53291800	2.71409200	-1.97642800
H	2.29981400	3.77615500	-1.29502700
H	1.81741300	2.36047500	-2.24795400
C	4.16564500	-3.19328000	1.00127200
H	4.24353600	-3.33267700	2.08860200
H	4.99401500	-3.72011200	0.50704300
C	2.82097100	-3.67523300	0.47699000
H	2.80645800	-3.53203600	-0.61636900
H	2.75501200	-4.76402400	0.64079700
C	1.63117600	-2.96416200	1.11736600
H	1.82580300	-1.87964500	1.13197500
H	1.54188000	-3.26903800	2.17531000
C	0.30825900	-3.19609900	0.39717700
H	0.41481600	-2.86776500	-0.65217600
H	0.07954200	-4.27618600	0.35536200
C	-0.85281500	-2.44243100	1.03736000
H	-0.55588900	-1.38886600	1.18525700
H	-1.04849200	-2.85119400	2.04503500
C	-2.13507400	-2.47014700	0.21410500
H	-1.93630200	-1.99678100	-0.76452100
H	-2.41852200	-3.51414500	-0.01042800

C	-3.30051200	-1.75641300	0.89125900
H	-3.60405000	-2.31977500	1.79179600
H	-2.95492500	-0.77153700	1.25218100
C	-4.50915500	-1.55267400	-0.01630700
H	-4.79867600	-2.51819500	-0.46584700
H	-4.21435400	-0.90604900	-0.86451300
C	-5.70410500	-0.92834600	0.70339300
H	-6.13443600	-1.66841900	1.40162500
H	-5.34381000	-0.09901200	1.33422400
C	-6.79919400	-0.39876900	-0.22134500
H	-7.58138000	0.08533800	0.38913400
H	-6.37741000	0.40045900	-0.85896100
C	-7.45068900	-1.45614900	-1.11098000
H	-7.82454100	-2.27943900	-0.47680300
H	-6.68994700	-1.90516300	-1.77129200
C	-8.59027200	-0.90366600	-1.96005000
H	-9.03893800	-1.68331100	-2.59359500
H	-8.23615200	-0.09775200	-2.62256900
H	-9.38990400	-0.48380100	-1.32908800

XIX

C	-1.12878000	-0.01335600	2.45484600
C	-2.58589600	0.26127700	2.80655600
C	-3.41284000	0.47132300	1.54299900
C	-3.37528500	-0.75744000	0.61064400
C	-1.88146000	-1.13243100	0.31798300
C	-1.73031800	-2.34873300	-0.59770300
C	-0.29505100	-2.59624300	-0.94251400
C	0.72935000	-2.06213100	-0.25712700
C	0.51618000	-1.15717200	0.94130800
C	-0.91849300	-1.23385600	1.53675000
C	1.65137200	-1.37775900	1.94663000
C	3.00483300	-1.08851600	1.30653800
C	3.18659000	-1.80329500	-0.00921400
C	2.11765200	-2.26773800	-0.68047300
C	4.59058500	-1.95527800	-0.55605200
C	5.27799000	-0.59311000	-0.70943800
C	5.43994700	-2.91446800	0.28667500
C	-3.95120500	-0.39802400	-0.76357900
C	-4.23884800	-1.88905000	1.17940400
C	-1.08838800	-2.53695700	2.33283900
O	-4.72239900	-1.08182500	-1.38574200
O	-3.43997200	0.74636800	-1.24089800
H	-0.72875700	0.87659900	1.94051300
H	-0.53524900	-0.12989900	3.37532300
H	-2.99920600	-0.56178400	3.41229700
H	-2.64573000	1.16165200	3.43795600
H	-3.01235100	1.34186400	1.00123800
H	-4.45990300	0.70656400	1.79421400
H	-1.49875500	-0.26860100	-0.25188800
H	-2.31209200	-2.20891900	-1.52306800
H	-2.16338300	-3.25054300	-0.12914200
H	-0.08136900	-3.23901400	-1.80331200
H	0.63640600	-0.12533500	0.56018700
H	1.63804200	-2.42191700	2.29909600
H	1.51630200	-0.73507200	2.82948100
H	3.81709700	-1.36106800	1.99973500
H	3.10915700	-0.00189200	1.14219800
H	2.26837800	-2.79920400	-1.62666900

H	4.49607000	-2.39612500	-1.56260600
H	6.25695400	-0.70294100	-1.20068200
H	5.45279000	-0.11850900	0.26962300
H	4.66712800	0.09495200	-1.31390600
H	6.42943100	-3.06194300	-0.17321500
H	5.60619600	-2.52385300	1.30318000
H	4.95420900	-3.89741300	0.37638200
H	-4.18112700	-2.79907700	0.56943100
H	-3.95436800	-2.14009600	2.20800600
H	-5.29356500	-1.57888600	1.19102400
H	-2.12199900	-2.69077400	2.66491500
H	-0.45899500	-2.52098400	3.23469200
H	-0.79238600	-3.41608000	1.74149600
C	-3.55190900	1.00025400	-2.64103000
H	-3.65490200	2.09035900	-2.74047200
H	-4.45728800	0.51332800	-3.02721000
C	-2.29967800	0.49858000	-3.34495700
H	-2.20805400	-0.58553200	-3.16040200
H	-2.43593500	0.61301600	-4.43354700
C	-1.03263000	1.21691500	-2.88772500
H	-1.04231500	1.26941400	-1.78746800
H	-1.06487500	2.26397200	-3.24013700
C	0.26379000	0.56038100	-3.35991300
H	0.20865600	0.39375200	-4.44951200
H	0.35134900	-0.43835800	-2.89535600
C	1.51250900	1.38590800	-3.04156000
H	1.39319300	2.39339900	-3.48061600
H	2.38860300	0.94015200	-3.54242600
C	1.80319800	1.52059300	-1.54808000
H	0.86459200	1.75195500	-1.01915100
H	2.13761200	0.54489100	-1.15483300
C	2.82821100	2.60172600	-1.21683200
H	3.75779300	2.41709400	-1.78295300
H	2.44966800	3.57053300	-1.58733000
C	3.17227500	2.71251500	0.27327000
H	3.78786800	3.61431400	0.43244500
H	3.81476000	1.86099800	0.55343800
C	1.96925200	2.74540400	1.22065200
H	1.41326400	1.79477900	1.14028100
H	2.33988900	2.77729300	2.25846900
C	1.00614400	3.90976100	0.98299100
H	0.81334700	4.01622300	-0.09726000
H	1.49402700	4.85107500	1.29423000
C	-0.34326400	3.76805600	1.69052000
H	-0.88891900	2.91551200	1.24780900
H	-0.95519800	4.65777300	1.46864800
C	-0.25528300	3.57886300	3.20128800
H	-1.25633900	3.55505600	3.65771800
H	0.30858700	4.39957000	3.67360100
H	0.24736700	2.63539600	3.46450200

XX

C	-1.12223800	-1.94945700	2.08357000
C	-2.50478500	-2.10647500	2.70862400
C	-3.46463100	-1.03704200	2.19617400
C	-3.61109300	-1.07765200	0.66066600
C	-2.18002600	-0.97604200	0.01816400
C	-2.21521900	-0.92942900	-1.51019100
C	-0.84774400	-0.86867000	-2.11202500

C	0.28127800	-1.13442400	-1.43561600
C	0.27493900	-1.50449900	0.03504100
C	-1.11897100	-1.99080200	0.54111900
C	1.42083400	-2.49026100	0.31097100
C	2.77657700	-1.93466100	-0.11412000
C	2.75537800	-1.38827300	-1.52015000
C	1.58839800	-1.04404300	-2.09308500
C	4.06231000	-1.28485900	-2.28039300
C	5.13829700	-0.52801700	-1.49680800
C	4.56533700	-2.67058800	-2.70711600
C	-4.37497000	0.15626100	0.16620900
C	-4.45475000	-2.29334400	0.24610900
C	-1.36980300	-3.42098100	0.03473000
O	-5.25668300	0.12965200	-0.65439700
O	-3.93280400	1.29426600	0.71513600
H	-0.70701400	-0.97675800	2.40328600
H	-0.44435200	-2.72146700	2.48042700
H	-2.90230200	-3.11459100	2.50744900
H	-2.42322700	-2.02972100	3.80418100
H	-3.08559300	-0.04700700	2.49159300
H	-4.45740300	-1.14988500	2.66191000
H	-1.80298600	0.00805900	0.34990500
H	-2.79850100	-0.05709800	-1.84873400
H	-2.75379900	-1.80311400	-1.91899400
H	-0.77864000	-0.60166700	-3.17229100
H	0.51430100	-0.57339300	0.58665400
H	1.24214900	-3.42070200	-0.25046500
H	1.44952400	-2.75828600	1.37766300
H	3.54207400	-2.72328600	-0.02853200
H	3.09826600	-1.14162500	0.58434400
H	1.59043400	-0.66844100	-3.12254300
H	3.85380700	-0.71314700	-3.20023000
H	6.04768200	-0.40698000	-2.10504300
H	5.42596400	-1.07137700	-0.58248800
H	4.79176400	0.47330100	-1.20120100
H	5.48288100	-2.58284700	-3.30958900
H	4.80382900	-3.29956100	-1.83448900
H	3.80900500	-3.19729800	-3.30741200
H	-4.43665900	-2.46859000	-0.83681200
H	-4.12973600	-3.20904300	0.75197300
H	-5.50392700	-2.11991100	0.52199600
H	-2.38338600	-3.77197100	0.24983400
H	-0.67854200	-4.12892800	0.51379100
H	-1.21710000	-3.49291200	-1.05273500
C	-4.43112400	2.51895100	0.17191000
H	-4.30803900	3.25740300	0.97662500
H	-5.49899000	2.40658700	-0.05979100
C	-3.63515000	2.91517200	-1.06208000
H	-3.78288100	2.14308400	-1.83557900
H	-4.06325700	3.84537200	-1.47206700
C	-2.15181000	3.09034900	-0.75688700
H	-1.82291600	2.22334800	-0.16433100
H	-2.01629200	3.97209200	-0.10397800
C	-1.25736400	3.22466400	-1.98549500
H	-1.65930900	4.00558600	-2.65380500
H	-1.29176400	2.28006800	-2.55789700
C	0.19523000	3.55663600	-1.63615400
H	0.23124500	4.55009200	-1.15277000
H	0.78323200	3.64989200	-2.56513000
C	0.85578600	2.53004800	-0.72108900

H	0.25988600	2.42074100	0.20085500
H	0.83510300	1.53923500	-1.20718300
C	2.28486300	2.86664400	-0.31208800
H	2.92722700	2.94503800	-1.20766900
H	2.30782300	3.86171100	0.16841700
C	2.85648200	1.82247700	0.63964000
H	2.88506100	0.85483500	0.11275500
H	2.14797600	1.68563300	1.47566800
C	4.23965400	2.14887700	1.19199200
H	4.92202400	2.37784700	0.35485500
H	4.18265900	3.07761200	1.78667100
C	4.85711000	1.02622700	2.03123500
H	5.83291200	1.36730700	2.41833700
H	5.07654100	0.17040700	1.37111500
C	4.00326800	0.53083000	3.20210100
H	4.55935800	-0.26495500	3.72485000
H	3.08868700	0.04659900	2.81755500
C	3.61926800	1.61747000	4.20142300
H	3.07431400	1.19693400	5.05975000
H	2.97221500	2.38212000	3.74391500
H	4.51257600	2.13178400	4.59117300

Molecule 7

I

C	-6.63778400	2.15422400	1.73855400
C	-5.76072500	3.39946100	1.79930600
C	-4.28547900	3.02065300	1.76387700
C	-3.90689600	2.23341200	0.49180900
C	-4.87301700	1.00618800	0.34301100
C	-4.57972500	0.16464500	-0.90121200
C	-5.50179400	-1.00823700	-1.02069800
C	-6.62925100	-1.14570900	-0.30447500
C	-7.06719900	-0.10556700	0.71182800
C	-6.39916800	1.28360900	0.48829800
C	-8.59853600	-0.08098000	0.78828000
C	-9.15488300	-1.45802700	1.13740200
C	-8.62508200	-2.53150700	0.21803900
C	-7.47005100	-2.33894100	-0.44235700
C	-9.42753800	-3.80616400	0.05870500
C	-9.72405200	-4.46431000	1.41076100
C	-10.71828400	-3.56665900	-0.73548500
C	-2.53136800	1.60158400	0.74528000
C	-3.84333500	3.18269400	-0.71287400
C	-7.00620000	1.95633600	-0.75362100
O	-2.21916000	1.04513100	1.76741700
O	-1.70784800	1.68649500	-0.30386100
H	-6.42986400	1.54261200	2.63461000
H	-7.69957000	2.44086300	1.79623500
H	-6.00906200	4.08508800	0.97227300
H	-5.97364300	3.95594700	2.72537900
H	-4.05260100	2.39411000	2.63859100
H	-3.64929000	3.91804000	1.83846400
H	-4.62451900	0.37439800	1.21402600
H	-3.53647900	-0.19257400	-0.87871200
H	-4.65129700	0.77843100	-1.81722600
H	-5.23037100	-1.79065900	-1.73766100
H	-6.71302900	-0.46750700	1.69701700
H	-9.01421200	0.22690900	-0.18491000
H	-8.93583400	0.65568700	1.53233500
H	-10.25628800	-1.43708300	1.10046900
H	-8.89871200	-1.71409800	2.18210700
H	-7.10186700	-3.12063000	-1.11614100
H	-8.80692200	-4.50611500	-0.52567900
H	-10.23455300	-5.42950300	1.27010000
H	-10.38027400	-3.83244900	2.03055200
H	-8.79768900	-4.64724800	1.97598200
H	-11.25804100	-4.51319500	-0.89476200
H	-11.40112600	-2.88510400	-0.20341000
H	-10.49995500	-3.12645200	-1.71965900
H	-3.67437100	2.65585600	-1.65990100
H	-4.76372500	3.77120700	-0.80667300
H	-3.01357800	3.89248300	-0.58505200
H	-6.48764100	2.88347300	-1.02319400
H	-8.05906800	2.21876500	-0.57403400
H	-6.97703900	1.29038100	-1.62875400
C	-0.43826300	1.05237000	-0.17978600
H	0.11347700	1.50348100	0.66087600
H	-0.58840900	-0.01055700	0.07102900
C	0.30255500	1.21783500	-1.48983900
H	-0.31238800	0.79563700	-2.30215300

H	0.41039700	2.29358700	-1.70729100
C	1.67391100	0.54899000	-1.47140100
H	1.55553800	-0.52899900	-1.25931700
H	2.26513900	0.95952800	-0.63505700
C	2.43850700	0.72097600	-2.78269500
H	1.79640200	0.38207400	-3.61360700
H	2.61731500	1.79691400	-2.95876500
C	3.76796000	-0.03113600	-2.84771900
H	4.18700100	0.07775700	-3.86271200
H	3.58191800	-1.11211200	-2.71366200
C	4.81393200	0.42692700	-1.83326000
H	4.96106200	1.51813400	-1.92943100
H	4.44246600	0.26167700	-0.80711700
C	6.15721500	-0.28023500	-1.98982700
H	6.00548800	-1.37146400	-1.90607400
H	6.54375200	-0.10899000	-3.01064200
C	7.20550300	0.16175700	-0.97304600
H	7.35792600	1.25288300	-1.05680000
H	6.81881000	-0.00954900	0.04770300
C	8.54710200	-0.54826700	-1.12891900
H	8.93394300	-0.37742700	-2.14969100
H	8.39384900	-1.63928000	-1.04523800
C	9.59626900	-0.10778900	-0.11229200
H	9.20846200	-0.27648200	0.90846000
H	9.75167600	0.98280500	-0.19746400
C	10.93613700	-0.82148400	-0.26641400
H	10.78022600	-1.91198400	-0.18086900
H	11.32371600	-0.65339200	-1.28737800
C	11.98602400	-0.38165800	0.74977300
H	12.14322200	0.70858000	0.66320500
H	11.59770500	-0.54837300	1.77066900
C	13.32483000	-1.09766400	0.59681200
H	13.71273800	-0.93124100	-0.42433400
H	13.16706900	-2.18783800	0.68352700
C	14.37527000	-0.65836900	1.61259400
H	14.53351000	0.43189800	1.52563200
H	13.98699500	-0.82408000	2.63389700
C	15.71462700	-1.37527700	1.46066400
H	15.55353200	-2.46403400	1.54703200
H	16.09974900	-1.20897000	0.43947000
C	16.75812200	-0.93047700	2.47937800
H	17.71044800	-1.46445600	2.34351100
H	16.41368600	-1.11781500	3.50892300
H	16.96425600	0.14823100	2.39215700

II

C	1.96344200	-0.04418000	2.30046100
C	0.60784100	-0.45144800	2.86513700
C	-0.40944700	-0.61317900	1.74394000
C	0.01772000	-1.67772800	0.70218700
C	1.45540200	-1.36171100	0.19931200
C	1.97534600	-2.38973300	-0.80711100
C	3.33774800	-2.03137600	-1.31479900
C	4.12951200	-1.11492900	-0.73461100
C	3.70334000	-0.35360900	0.50743500
C	2.53832800	-1.04245300	1.27682400
C	4.93875100	-0.02243900	1.35257400
C	5.95303200	0.78331200	0.54707500
C	6.28257300	0.12561600	-0.77076300

C	5.42946600	-0.76096800	-1.31298100
C	7.58666600	0.48917300	-1.45009900
C	7.71881300	2.00347800	-1.64782200
C	8.79771000	-0.07894300	-0.69888700
C	-0.94727400	-1.46737500	-0.47402100
C	-0.17628400	-3.09129000	1.27342800
C	3.06850600	-2.28859300	2.00477800
O	-0.72745100	-0.79428600	-1.45031100
O	-2.12133100	-2.06950500	-0.26718000
H	1.84721100	0.93329300	1.80313100
H	2.68155100	0.10740400	3.12140500
H	0.69201400	-1.38345100	3.44776400
H	0.25381400	0.31675500	3.57069900
H	-0.51212000	0.35507900	1.22601300
H	-1.40415600	-0.87004700	2.14045600
H	1.33600000	-0.42208500	-0.36542600
H	1.27791400	-2.45649700	-1.65761100
H	2.00555600	-3.40121800	-0.36220700
H	3.69294900	-2.54555500	-2.21438200
H	3.30891100	0.61528600	0.15087400
H	5.42106500	-0.95379600	1.69124400
H	4.65070700	0.53987700	2.25352800
H	6.87201200	0.92713500	1.13857500
H	5.55970700	1.79896500	0.35998900
H	5.68842200	-1.24042400	-2.26359500
H	7.57018100	0.02148100	-2.44889500
H	8.62831700	2.24400500	-2.21968000
H	7.78788200	2.53073000	-0.68277900
H	6.85427300	2.40978500	-2.19448000
H	9.73034500	0.14188400	-1.24122900
H	8.89229200	0.35855300	0.30783200
H	8.71604600	-1.17025200	-0.58728400
H	0.04051300	-3.86742700	0.52588300
H	0.47477200	-3.26892300	2.13617100
H	-1.21406700	-3.22961400	1.60102500
H	2.27666000	-2.84465500	2.52010700
H	3.80037100	-1.99923700	2.77284100
H	3.57240600	-2.98146700	1.31525400
C	-3.13838700	-1.92390900	-1.25369400
H	-3.12885000	-0.89422300	-1.64312300
H	-2.91056400	-2.59488800	-2.09898800
C	-4.46587900	-2.26474700	-0.60912800
H	-4.39004500	-3.24945000	-0.11874100
H	-4.64900400	-1.53083800	0.19242600
C	-5.61797900	-2.26689000	-1.61069400
H	-5.45850300	-3.07376100	-2.34566900
H	-5.59657000	-1.32997200	-2.19343600
C	-7.00099400	-2.44308400	-0.97460600
H	-7.07023800	-3.45026500	-0.52953000
H	-7.75691700	-2.41284500	-1.77755300
C	-7.36155800	-1.40363600	0.09275700
H	-6.74112400	-1.56054400	0.99259600
H	-8.40104100	-1.57436800	0.42018100
C	-7.20071200	0.03986000	-0.37277100
H	-6.16774800	0.18431300	-0.72823300
H	-7.84683000	0.22133900	-1.25147200
C	-7.48983500	1.08060900	0.70418500
H	-8.51134300	0.93640900	1.09552200
H	-6.81712900	0.90066100	1.56116400
C	-7.34259700	2.52774600	0.21979700

H	-8.17048500	2.75773400	-0.47278200
H	-7.46985200	3.20667400	1.08037400
C	-6.02181500	2.84621200	-0.48695700
H	-6.03967600	3.89932100	-0.81532700
H	-5.94901300	2.25539700	-1.41653300
C	-4.76488000	2.61481800	0.35021700
H	-4.79140200	1.61307200	0.81405400
H	-4.75014000	3.33214700	1.19041000
C	-3.47747000	2.74054900	-0.46068300
H	-3.41771400	1.89993000	-1.17568300
H	-3.51986300	3.65467900	-1.07978400
C	-2.20781900	2.77761600	0.38463600
H	-2.21359500	3.69614600	0.99893400
H	-2.21578400	1.93781400	1.10313900
C	-0.92830400	2.72291200	-0.44706000
H	-1.02137500	3.41651800	-1.30199200
H	-0.82039200	1.71627500	-0.88863300
C	0.33303500	3.07865200	0.33442300
H	0.26230100	4.12371900	0.68671200
H	0.38951900	2.46241400	1.25037800
C	1.61275700	2.90011800	-0.47769500
H	1.70851000	1.83817800	-0.76480500
H	1.51612600	3.45346100	-1.42744200
C	2.87264000	3.35738700	0.24819100
H	3.76953200	3.20331200	-0.37097900
H	3.01955900	2.80337800	1.18964900
H	2.81999200	4.42799000	0.50201000

III

C	-7.17619200	-1.58510200	1.18342100
C	-6.60689800	-2.85772200	1.79936300
C	-5.37708600	-3.32344300	1.03032600
C	-4.25606700	-2.26338800	1.00693700
C	-4.84535000	-0.90608500	0.48513500
C	-3.80555100	0.21582900	0.43588600
C	-4.38152200	1.49787800	-0.07879300
C	-5.69909700	1.72816600	-0.19685800
C	-6.73455500	0.68376100	0.18214800
C	-6.17380100	-0.41414500	1.13412600
C	-8.00450100	1.38378900	0.68086900
C	-8.55352400	2.34343200	-0.37047900
C	-7.50024700	3.30614600	-0.86237600
C	-6.19809200	2.99349500	-0.74441800
C	-7.94958000	4.61742300	-1.47290800
C	-8.94624000	4.40040800	-2.61687100
C	-8.52153900	5.56804900	-0.41305200
C	-3.25988100	-2.68633300	-0.08136000
C	-3.55924300	-2.20614100	2.37354900
C	-5.97238600	0.17254400	2.54078900
O	-3.57777400	-3.08487700	-1.17312400
O	-1.98245100	-2.53136500	0.27987800
H	-7.49556200	-1.81506200	0.15129100
H	-8.08215400	-1.27763300	1.72894500
H	-6.36596800	-2.69578500	2.86305700
H	-7.37129300	-3.65019900	1.78170400
H	-5.66390500	-3.54401500	-0.00933600
H	-4.98162200	-4.26078400	1.45533800
H	-5.11701300	-1.12746000	-0.56218000
H	-2.95580400	-0.08363400	-0.20015500

H	-3.36670400	0.38897600	1.43509800
H	-3.67744500	2.28462500	-0.37066600
H	-7.01285300	0.16898300	-0.75839000
H	-7.77771200	1.95842700	1.59358400
H	-8.77409600	0.64404200	0.94647700
H	-9.41176700	2.89895400	0.04153800
H	-8.95473100	1.77286800	-1.22843200
H	-5.44269500	3.70603800	-1.09404500
H	-7.05248100	5.09905500	-1.89699900
H	-9.19619300	5.35661000	-3.10184500
H	-9.88827000	3.96072100	-2.25181600
H	-8.53259400	3.72701500	-3.38272900
H	-8.78719800	6.53742100	-0.86293900
H	-9.43480100	5.15881300	0.04777100
H	-7.79070800	5.75058000	0.38856900
H	-2.81912500	-1.39929000	2.43847500
H	-4.28368500	-2.07432800	3.18608600
H	-3.02684400	-3.14964900	2.56071700
H	-5.46986700	-0.52737900	3.21808300
H	-6.94200600	0.41968500	2.99737100
H	-5.37639500	1.09664500	2.51343400
C	-0.99471600	-2.83265300	-0.70150000
H	-1.07556000	-3.89492800	-0.98417200
H	-1.19741100	-2.24441000	-1.61133900
C	0.36523900	-2.51043000	-0.11907000
H	0.38425200	-1.44751100	0.17437800
H	0.50576100	-3.09234400	0.80701500
C	1.49975900	-2.80193100	-1.09671900
H	1.33757900	-2.23295800	-2.02954400
H	1.47287700	-3.86781900	-1.38532900
C	2.87961700	-2.46650000	-0.53813800
H	2.90766500	-1.39928800	-0.25434500
H	3.04178300	-3.03088500	0.39748000
C	4.01826800	-2.76108600	-1.50994200
H	3.85073200	-2.20224600	-2.44820800
H	3.99276500	-3.82985800	-1.78885500
C	5.39858600	-2.41495500	-0.95883900
H	5.41224600	-1.34817100	-0.67645600
H	5.57113800	-2.97750600	-0.02327200
C	6.53225600	-2.70987500	-1.93904800
H	6.46181800	-3.76517400	-2.25432400
H	6.38311300	-2.11130300	-2.85593500
C	7.93616400	-2.45100400	-1.39114100
H	8.09780000	-3.07303900	-0.49217800
H	8.67622800	-2.79727400	-2.13299500
C	8.23038700	-0.99129200	-1.05070700
H	7.54921400	-0.64467800	-0.25447100
H	8.01013500	-0.36136200	-1.93182600
C	9.67045400	-0.75114700	-0.60629700
H	10.35918800	-1.08374000	-1.40371400
H	9.89337100	-1.38951700	0.26757800
C	9.96927500	0.70382300	-0.25704300
H	9.74085800	1.34228700	-1.12942400
H	9.28430800	1.03496000	0.54422400
C	11.41085500	0.94612400	0.18049500
H	11.63954700	0.30784100	1.05291200
H	12.09557700	0.61492100	-0.62091300
C	11.70946600	2.40149800	0.52861000
H	11.02562200	2.73219400	1.33103300
H	11.47888300	3.03948900	-0.34356600

C	13.15163300	2.64492200	0.96346700
H	13.38253500	2.00683900	1.83571400
H	13.83545300	2.31391300	0.16091400
C	13.45121000	4.10098800	1.31139000
H	13.21813300	4.73622300	0.43906700
H	12.76680300	4.42917900	2.11305600
C	14.89443400	4.33534200	1.74345600
H	15.07792600	5.39246500	1.98705000
H	15.59936700	4.04857400	0.94688000
H	15.14439600	3.73851100	2.63508400

IV

C	-4.38469500	-2.08318000	-1.52249800
C	-2.94384300	-2.56677100	-1.39875400
C	-2.06662800	-1.48909200	-0.77245900
C	-2.55786800	-1.08103500	0.63633300
C	-4.06909700	-0.68760900	0.55871000
C	-4.64118400	-0.27293900	1.91484800
C	-6.07998900	0.12831900	1.81759700
C	-6.85454400	-0.13403800	0.75216000
C	-6.32370900	-0.86509500	-0.46939700
C	-5.02371200	-1.67281800	-0.17974500
C	-7.45776200	-1.67187600	-1.11289000
C	-8.63281500	-0.77169500	-1.48229300
C	-9.09111800	0.06705100	-0.31399400
C	-8.24840000	0.31868300	0.70285200
C	-10.50976100	0.59774700	-0.31883600
C	-10.81864900	1.38421800	-1.59757800
C	-11.53404400	-0.52285600	-0.09707900
C	-1.78377700	0.17580800	1.05570900
C	-2.20696600	-2.17500100	1.65212300
C	-5.36463500	-2.92194100	0.64864200
O	-1.05965700	0.25575600	2.01504800
O	-1.99485000	1.20327000	0.22177500
H	-4.39745200	-1.20854500	-2.19741000
H	-4.99885500	-2.85850600	-2.00687000
H	-2.89870200	-3.49634100	-0.80844200
H	-2.55333300	-2.82343400	-2.39599200
H	-2.08015500	-0.60123000	-1.42382200
H	-1.01822700	-1.82383200	-0.70883900
H	-4.07769200	0.21798900	-0.06983400
H	-4.05385100	0.56390500	2.33121000
H	-4.53963600	-1.08869100	2.65294500
H	-6.51403200	0.66142500	2.67036300
H	-6.04723900	-0.08141500	-1.20205800
H	-7.81495300	-2.43918700	-0.40710900
H	-7.09645900	-2.19850400	-2.00868500
H	-9.46828600	-1.38318400	-1.86047500
H	-8.35003400	-0.10715400	-2.31962000
H	-8.59735200	0.91783400	1.55129000
H	-10.59538900	1.29458500	0.53184500
H	-11.82321100	1.83164200	-1.54612800
H	-10.79376400	0.73335700	-2.48628800
H	-10.09108000	2.19548400	-1.75136100
H	-12.55414300	-0.11181900	-0.04161500
H	-11.51938300	-1.25434100	-0.92084700
H	-11.33069300	-1.06342400	0.83909400
H	-2.57435300	-1.93856300	2.65895900

H	-2.61071700	-3.15003800	1.35715300
H	-1.11532800	-2.27121400	1.72760400
H	-4.47078800	-3.47126600	0.96532000
H	-5.97631700	-3.61990000	0.05848700
H	-5.93700600	-2.66557800	1.55230700
C	-1.30751300	2.42284300	0.48724800
H	-1.91107200	3.20329400	0.00251300
H	-1.29491800	2.60427500	1.57260400
C	0.10738100	2.40379900	-0.06553100
H	0.65444300	1.57947600	0.41688900
H	0.06671800	2.18132700	-1.14563200
C	0.83193800	3.72775000	0.16781400
H	0.91215200	3.91095900	1.25414500
H	0.21841000	4.55208800	-0.23554200
C	2.22494100	3.79900300	-0.45877300
H	2.13997800	3.66624000	-1.55239800
H	2.62816200	4.81516500	-0.30920700
C	3.22486600	2.78567100	0.09424600
H	2.87444400	1.75959400	-0.11104600
H	3.26357800	2.87569900	1.19493800
C	4.63034700	2.95045800	-0.47691300
H	4.58700600	2.87643900	-1.57855900
H	4.99658700	3.96993900	-0.25922700
C	5.63123600	1.93026200	0.05742800
H	5.67059700	2.00055200	1.15929000
H	5.26694300	0.91114600	-0.16477000
C	7.03818900	2.09720900	-0.50899800
H	6.99821500	2.02984300	-1.61115500
H	7.40361700	3.11537400	-0.28401000
C	8.03802100	1.07421500	0.02212300
H	7.67280100	0.05621500	-0.20387600
H	8.07718300	1.14063900	1.12430200
C	9.44544500	1.24169400	-0.54292800
H	9.81005900	2.26024300	-0.31816400
H	9.40645600	1.17392600	-1.64511800
C	10.44520100	0.21848500	-0.01143700
H	10.49386900	0.29285700	1.09026400
H	10.06703200	-0.79531900	-0.22869400
C	11.84927700	0.38647500	-0.58887300
H	12.17825700	1.42672200	-0.42215100
H	11.80806200	0.25620200	-1.68552500
C	12.89877200	-0.56153000	-0.00728900
H	12.96556600	-0.40550400	1.08472600
H	13.88854600	-0.28693500	-0.41118100
C	12.64918100	-2.04215300	-0.28736300
H	11.69584600	-2.35735000	0.17124900
H	12.52402800	-2.19035600	-1.37574100
C	13.76489600	-2.95240200	0.22078800
H	14.71948400	-2.64949300	-0.24385100
H	13.89502300	-2.79231200	1.30535800
C	13.51039700	-4.43085300	-0.05080000
H	14.33147700	-5.05924800	0.32538300
H	13.40872300	-4.62478900	-1.13052300
H	12.58095700	-4.76999500	0.43381000

V

C	-6.91860000	-0.96761700	1.58845300
C	-6.53038300	-2.29386200	2.23157000
C	-5.57300300	-3.06680400	1.33344900

C	-4.27646200	-2.28439900	1.03693300
C	-4.65021700	-0.86538800	0.48151800
C	-3.42333600	-0.00644000	0.16627700
C	-3.79825000	1.33720500	-0.37622200
C	-5.03969400	1.84522500	-0.31521100
C	-6.19060800	1.08051300	0.31511200
C	-5.71780800	-0.05257800	1.27356300
C	-7.18552000	2.07282100	0.92906300
C	-7.69190600	3.06249000	-0.11580600
C	-6.55943300	3.73752600	-0.85066900
C	-5.34964100	3.15339300	-0.90045900
C	-6.82247200	5.07551200	-1.51001200
C	-8.01856500	5.01307800	-2.46635200
C	-7.00036300	6.19204600	-0.47280700
C	-3.58930900	-2.98398300	-0.14385700
C	-3.36138300	-2.29852400	2.26937400
C	-5.16320900	0.56163100	2.56920500
O	-4.16264700	-3.36552800	-1.13255500
O	-2.26616200	-3.09828400	0.00539500
H	-7.44908900	-1.18493200	0.64425000
H	-7.63495800	-0.43427100	2.23287300
H	-6.08415100	-2.12320500	3.22525700
H	-7.43457100	-2.89721600	2.40780200
H	-6.07144000	-3.28240000	0.37587800
H	-5.31511600	-4.04020700	1.78245800
H	-5.13508100	-1.08576100	-0.48594300
H	-2.77533600	-0.52490900	-0.56015200
H	-2.79463100	0.12633500	1.06521000
H	-3.00534500	1.92942300	-0.84575200
H	-6.72367200	0.58216500	-0.51826100
H	-6.69467200	2.63905000	1.73723000
H	-8.03607400	1.53880100	1.37780800
H	-8.33534900	3.81758000	0.36455300
H	-8.34017000	2.54188200	-0.84472100
H	-4.53227000	3.65908800	-1.42654200
H	-5.92840200	5.32105700	-2.10746400
H	-8.14251000	5.96944500	-2.99738700
H	-8.95704800	4.81381200	-1.92456100
H	-7.88632200	4.21998000	-3.21775200
H	-7.12999200	7.16657800	-0.96892300
H	-7.88906900	6.02326100	0.15615300
H	-6.12468500	6.25810800	0.18965200
H	-2.46788300	-1.67551500	2.14114100
H	-3.89266400	-1.95720900	3.16575700
H	-3.01554600	-3.32356100	2.46528700
H	-4.71153800	-0.18875800	3.22810900
H	-5.96787100	1.04664700	3.14097400
H	-4.40009200	1.32607000	2.36101300
C	-1.54143400	-3.67171800	-1.07875700
H	-1.89027000	-4.70426000	-1.24331900
H	-1.76346300	-3.10923400	-2.00035400
C	-0.06702100	-3.62403400	-0.73755000
H	0.20797500	-2.57703300	-0.53283100
H	0.10385200	-4.18196800	0.19862000
C	0.80214900	-4.20033600	-1.85319300
H	0.65190300	-3.61053100	-2.77520100
H	0.45551600	-5.22143700	-2.08737000
C	2.29482400	-4.24931900	-1.52489600
H	2.45000500	-4.87004300	-0.62421400
H	2.81941500	-4.77029800	-2.34382500

C	2.94626300	-2.88416800	-1.31347800
H	2.48093800	-2.37300000	-0.45305200
H	2.74257200	-2.24433600	-2.19138900
C	4.45297900	-2.96130100	-1.08378700
H	4.65589700	-3.60944900	-0.21229700
H	4.92945500	-3.46076900	-1.94646300
C	5.10793400	-1.60085600	-0.86382400
H	4.90244300	-0.95247200	-1.73455400
H	4.63232300	-1.10237300	-0.00004500
C	6.61478900	-1.67486100	-0.63548000
H	6.82046300	-2.32168300	0.23635900
H	7.09054400	-2.17458000	-1.49843900
C	7.26860800	-0.31332800	-0.41844900
H	6.79207200	0.18682300	0.44385000
H	7.06307100	0.33290200	-1.29077400
C	8.77542500	-0.38572400	-0.18918900
H	9.25184300	-0.88739300	-1.05067300
H	8.98108000	-1.03031900	0.68431900
C	9.42870700	0.97654900	0.02483900
H	9.22329400	1.62066700	-0.84908500
H	8.95168800	1.47858000	0.88578600
C	10.93546900	0.90506200	0.25483400
H	11.14084900	0.26212500	1.12964000
H	11.41227300	0.40181900	-0.60553200
C	11.58849600	2.26784800	0.46659500
H	11.11102300	2.77120200	1.32655800
H	11.38306100	2.91029900	-0.40859200
C	13.09517100	2.19722700	0.69712000
H	13.30071600	1.55531300	1.57290300
H	13.57264100	1.69285600	-0.16248200
C	13.74918100	3.56064300	0.90758800
H	13.54166300	4.19991700	0.03176400
H	13.26974500	4.06276900	1.76596100
C	15.25429900	3.48088500	1.13725300
H	15.69512800	4.47809400	1.28472500
H	15.76318000	3.01399000	0.27891100
H	15.48906900	2.87650500	2.02793000

VI

C	-3.34065600	2.01446200	1.78445800
C	-2.56952900	3.24281200	2.25364400
C	-1.22474700	2.83900500	2.84437900
C	-0.33811600	2.08232700	1.83471300
C	-1.15050300	0.87789400	1.23895500
C	-0.35908200	0.08103100	0.20077500
C	-1.13905400	-1.07345600	-0.34388700
C	-2.46402500	-1.21827800	-0.18081600
C	-3.29320200	-0.20604900	0.59099200
C	-2.59181200	1.17757600	0.72806900
C	-4.70969700	-0.15694300	0.00567700
C	-5.36389200	-1.53504300	0.03062300
C	-4.49299900	-2.58647100	-0.61301600
C	-3.16624400	-2.39199300	-0.70864000
C	-5.15185000	-3.84090300	-1.14813300
C	-6.00291600	-4.53640400	-0.07998400
C	-5.97447500	-3.55290700	-2.41085500
C	0.79100200	1.41498300	2.63045100
C	0.24722800	3.06114000	0.80822400
C	-2.60453600	1.90306200	-0.62665400

O	0.63518200	0.85975600	3.68875300
O	1.97507000	1.46862800	2.00942000
H	-3.53734500	1.37425100	2.66291100
H	-4.32432100	2.31654300	1.39204900
H	-2.43461100	3.95381000	1.42161700
H	-3.15748400	3.77907900	3.01473600
H	-1.39433900	2.18461400	3.71306300
H	-0.67915100	3.72211800	3.21553000
H	-1.29905800	0.20959400	2.10564500
H	0.58308400	-0.28734400	0.63773600
H	-0.04973700	0.73497100	-0.63345000
H	-0.58617000	-1.83285800	-0.90744500
H	-3.39556300	-0.60775400	1.61822900
H	-4.66806000	0.19056000	-1.03938200
H	-5.33229900	0.55980900	0.56123500
H	-6.34311700	-1.49367600	-0.47367800
H	-5.58070800	-1.83034300	1.07387000
H	-2.54614000	-3.15681300	-1.18934700
H	-4.34184200	-4.53279400	-1.43408400
H	-6.40565000	-5.48723700	-0.46167300
H	-6.86036300	-3.91290500	0.22005600
H	-5.41044200	-4.75397000	0.82154000
H	-6.39606100	-4.48389900	-2.82092900
H	-6.81768500	-2.87594800	-2.19975400
H	-5.35246300	-3.08611900	-3.18882400
H	0.82886700	2.55440100	0.02972500
H	-0.53704600	3.65204900	0.31982500
H	0.92469500	3.76785100	1.30916600
H	-2.01933700	2.82897200	-0.60524900
H	-3.63077300	2.17964200	-0.90915100
H	-2.19930400	1.27271900	-1.43207400
C	3.06501000	0.77657800	2.61483100
H	3.96700900	1.24243200	2.19329800
H	3.04740100	0.94836600	3.70151700
C	3.02882500	-0.71052600	2.30479900
H	2.11856000	-1.14595800	2.74785400
H	2.94870000	-0.83251300	1.21160200
C	4.26507300	-1.43576300	2.83083600
H	4.38383000	-1.21682200	3.90556000
H	5.16369800	-1.02138700	2.34103600
C	4.22891300	-2.95596500	2.64614000
H	3.45231100	-3.37859900	3.30584200
H	5.18790200	-3.37166400	2.99953200
C	3.96893700	-3.43455600	1.21545800
H	2.93122400	-3.19029000	0.92635200
H	4.02857100	-4.53603100	1.19318700
C	4.91642100	-2.85225100	0.17078900
H	4.81595000	-1.75448100	0.17209200
H	5.96445800	-3.05639000	0.45625800
C	4.65581000	-3.37451700	-1.23967300
H	4.87053700	-4.45610000	-1.27433200
H	3.57794800	-3.28262300	-1.46008300
C	5.46246500	-2.66960500	-2.33432200
H	6.53486100	-2.88970300	-2.19500300
H	5.18718200	-3.10739800	-3.30918400
C	5.27822900	-1.14933300	-2.40071000
H	5.85322700	-0.76036100	-3.25828000
H	5.72806400	-0.67715700	-1.50980200
C	3.82683400	-0.69324200	-2.52454800
H	3.23889100	-1.07973800	-1.67337500

H	3.37652600	-1.14404000	-3.42755400
C	3.65793500	0.82223500	-2.57012500
H	4.06792800	1.25852600	-1.64115700
H	4.26279300	1.24187600	-3.39380000
C	2.20462400	1.25663600	-2.72977300
H	1.81431100	0.88459100	-3.69390800
H	1.59752000	0.76264000	-1.95126600
C	1.99081400	2.76380200	-2.64012800
H	2.62296400	3.27412400	-3.38872900
H	2.34627500	3.11998000	-1.65621300
C	0.53659300	3.18401600	-2.83265500
H	0.20553700	2.90594100	-3.84935600
H	-0.10511300	2.60734600	-2.14354600
C	0.29621700	4.67403400	-2.60739300
H	0.62806100	4.93955700	-1.58832600
H	0.93922200	5.25353500	-3.29255000
C	-1.15859300	5.08865500	-2.79547500
H	-1.29806400	6.16696800	-2.62755100
H	-1.81872500	4.55276900	-2.09513300
H	-1.50916600	4.85995900	-3.81440600

VII

C	-7.15366900	-1.76713200	-0.86206000
C	-6.92383600	-3.27384700	-0.83724700
C	-5.45196200	-3.59760300	-1.05999500
C	-4.53752900	-2.95922000	0.00693700
C	-4.83730300	-1.42107200	0.08308800
C	-3.97914300	-0.69918000	1.12439000
C	-4.26524100	0.76905500	1.17189200
C	-5.33833100	1.33751900	0.59882500
C	-6.36762000	0.52302500	-0.16550700
C	-6.33160400	-0.99338800	0.18878400
C	-7.74364200	1.18626700	-0.03021300
C	-7.71039600	2.62941400	-0.52407900
C	-6.60329400	3.42385100	0.12503200
C	-5.53995200	2.78901200	0.64805700
C	-6.73130200	4.93221700	0.17597300
C	-6.97154500	5.52978700	-1.21481800
C	-7.81835700	5.37489900	1.16411500
C	-3.10159300	-3.03592900	-0.52876800
C	-4.65774300	-3.72775700	1.32987100
C	-6.92066200	-1.21287800	1.59161900
O	-2.78749500	-2.74166400	-1.65478500
O	-2.21354600	-3.42979300	0.39064700
H	-6.87892400	-1.39340300	-1.86456400
H	-8.22537700	-1.54994500	-0.73121200
H	-7.27915400	-3.70126200	0.11494000
H	-7.52687600	-3.75198400	-1.62489000
H	-5.14511600	-3.21943800	-2.04729300
H	-5.28883900	-4.68793300	-1.07286000
H	-4.51354600	-1.04573900	-0.90361800
H	-2.90961700	-0.85427000	0.90543600
H	-4.13092800	-1.13325400	2.12911100
H	-3.55127500	1.40394700	1.70768500
H	-6.08998600	0.60006300	-1.23511100
H	-8.05364400	1.18633900	1.02742700
H	-8.50219400	0.61961800	-0.59018100
H	-8.68343000	3.11186200	-0.33593700
H	-7.57772200	2.64907800	-1.62163800

H	-4.74866800	3.37592100	1.12764300
H	-5.76964300	5.32432900	0.54756200
H	-6.97894400	6.62974600	-1.16982000
H	-7.94216400	5.21096200	-1.62739500
H	-6.18576600	5.22219200	-1.92124800
H	-7.85594600	6.47321200	1.23443200
H	-8.81677400	5.03202200	0.84872500
H	-7.62469600	4.97317100	2.16967100
H	-4.09780100	-3.25548300	2.14624900
H	-5.70425100	-3.82426400	1.64234800
H	-4.25768000	-4.74441000	1.20863200
H	-6.81407800	-2.24902500	1.93256200
H	-7.99616300	-0.98290400	1.59812200
H	-6.44049000	-0.56431100	2.33915400
C	-0.83884400	-3.44101500	0.01159700
H	-0.35804100	-4.15316200	0.69701400
H	-0.74835700	-3.81771700	-1.01814300
C	-0.21421600	-2.06121100	0.13237600
H	-0.75256700	-1.37090100	-0.53690000
H	-0.35789000	-1.69355000	1.16261200
C	1.27041300	-2.06314500	-0.21897300
H	1.40214700	-2.44139500	-1.24816200
H	1.80586900	-2.77343800	0.43658600
C	1.91803700	-0.68622900	-0.10360300
H	1.38070800	0.02322000	-0.75758800
H	1.78656400	-0.30619300	0.92531100
C	3.40164200	-0.67865900	-0.45925600
H	3.53189800	-1.06073400	-1.48767400
H	3.93766400	-1.38933200	0.19536000
C	4.05097600	0.69754500	-0.34740000
H	3.51417900	1.40828600	-1.00110200
H	3.92164400	1.07973700	0.68121500
C	5.53483900	0.70442200	-0.70364900
H	6.06073400	-0.01684600	-0.05457500
H	5.66469300	0.33199500	-1.73614300
C	6.18133400	2.08288900	-0.58188000
H	5.59954300	2.80135700	-1.18464800
H	6.09862400	2.43079200	0.46376600
C	7.64533800	2.14330500	-1.01927700
H	7.98198900	3.19396200	-0.99117200
H	7.72332300	1.83056500	-2.07630500
C	8.59948600	1.30011700	-0.17564700
H	8.32327000	0.23391700	-0.24668900
H	8.48067200	1.57242800	0.88899200
C	10.06351700	1.45751400	-0.57660400
H	10.17867500	1.19677700	-1.64413400
H	10.35276800	2.52078400	-0.49503400
C	11.02103000	0.60939400	0.25531000
H	10.90340800	0.86815100	1.32305700
H	10.73328100	-0.45409500	0.17119300
C	12.48546900	0.76783200	-0.14306500
H	12.77331600	1.83125100	-0.05854800
H	12.60277400	0.50948900	-1.21094400
C	13.44258800	-0.08084700	0.68874300
H	13.32506700	0.17744200	1.75681200
H	13.15448400	-1.14442000	0.60443000
C	14.90812400	0.07679200	0.29095600
H	15.02259900	-0.18099300	-0.77638900
H	15.19303600	1.14005400	0.37511500
C	15.85706600	-0.77454100	1.12716900

H	16.90373800	-0.63882400	0.81640500
H	15.61686800	-1.84568000	1.03402500
H	15.78888900	-0.51333500	2.19526600

VIII

C	-7.19202700	-2.02276900	-0.98077500
C	-6.54156500	-3.40063300	-1.02197500
C	-5.07286700	-3.29030000	-1.41113400
C	-4.26520200	-2.41247500	-0.43204000
C	-4.98000700	-1.02428200	-0.28023500
C	-4.25449700	-0.08393900	0.68482500
C	-4.94099900	1.24052000	0.80610900
C	-6.18792700	1.47619400	0.36706200
C	-7.01868800	0.39935600	-0.30920000
C	-6.51313100	-1.04182800	-0.00339600
C	-8.50380900	0.64034500	-0.01274600
C	-8.93658400	2.03206900	-0.46387700
C	-8.03959600	3.11103600	0.09237500
C	-6.78918300	2.80849200	0.48248200
C	-8.58728400	4.51874200	0.20385100
C	-9.13682900	5.02255300	-1.13527100
C	-9.64249000	4.62917700	1.31220500
C	-2.93489400	-2.07896600	-1.12111000
C	-4.01287500	-3.17879000	0.87377500
C	-6.85550900	-1.41425300	1.44834500
O	-2.83712000	-1.72538500	-2.26888500
O	-1.87717300	-2.17799600	-0.31033300
H	-7.14766500	-1.59054100	-1.99635500
H	-8.25988100	-2.12029400	-0.72970700
H	-6.65225200	-3.90730800	-0.04898300
H	-7.06713000	-4.03508500	-1.75266500
H	-4.99831900	-2.84436500	-2.41488900
H	-4.60826800	-4.28832200	-1.47226800
H	-4.88730200	-0.57467300	-1.28455900
H	-3.21532200	0.07315200	0.34999900
H	-4.16555800	-0.54082700	1.68696700
H	-4.38417400	2.05425800	1.28323700
H	-6.89159100	0.54995500	-1.39924100
H	-8.68578000	0.55258100	1.07068000
H	-9.12480800	-0.12036500	-0.50840500
H	-9.98102900	2.21524900	-0.16304200
H	-8.93252400	2.08836100	-1.56815200
H	-6.15153900	3.59789800	0.89579400
H	-7.74377200	5.17078100	0.48654800
H	-9.45378500	6.07379300	-1.05520700
H	-10.01418400	4.43796600	-1.45550900
H	-8.37676600	4.95446600	-1.92821400
H	-9.98554900	5.66999600	1.42016300
H	-10.52857900	4.01289700	1.09101000
H	-9.23456800	4.30031700	2.27941300
H	-3.52219300	-2.56393300	1.63813800
H	-4.94710900	-3.56934000	1.29454500
H	-3.35664200	-4.03937000	0.68056300
H	-6.41500700	-2.37152300	1.74983400
H	-7.94389200	-1.50823200	1.57578300
H	-6.50589200	-0.64782300	2.15577700
C	-0.61197600	-1.81549200	-0.85566200
H	-0.39342500	-2.45616300	-1.72532000
H	-0.66262800	-0.77909700	-1.22801400

C	0.43259100	-1.96888900	0.22961200
H	0.14759300	-1.34098500	1.09023300
H	0.42555000	-3.01166000	0.58828700
C	1.83043300	-1.59106600	-0.25119800
H	1.82513700	-0.54830000	-0.61561300
H	2.10172200	-2.21347000	-1.12250100
C	2.90041100	-1.74123600	0.82637700
H	2.62666000	-1.12350400	1.70026800
H	2.91020900	-2.78519300	1.18728900
C	4.29922200	-1.35625200	0.35423000
H	4.28796400	-0.31158000	-0.00522400
H	4.57133100	-1.97209900	-0.52175000
C	5.37135700	-1.50775700	1.42924800
H	5.09861900	-0.89273000	2.30568400
H	5.38327000	-2.55264600	1.78820900
C	6.76977400	-1.11907800	0.95779300
H	7.04840500	-1.74077500	0.08748000
H	6.74530100	-0.07889300	0.58989700
C	7.83658800	-1.26430400	2.04122100
H	7.79581100	-2.29156300	2.44268800
H	7.58569600	-0.60009600	2.88803700
C	9.26399400	-0.97256600	1.57688500
H	9.52760200	-1.66139800	0.75392500
H	9.96056800	-1.20749500	2.40007700
C	9.50826200	0.46564700	1.12422500
H	9.18783500	1.15805600	1.92402100
H	8.87432600	0.69930400	0.25147000
C	10.96425400	0.74779500	0.76360200
H	11.60558200	0.54194900	1.64001300
H	11.28196900	0.03465700	-0.01653900
C	11.20430600	2.17994700	0.29006800
H	10.81079100	2.87510500	1.05168400
H	10.60852600	2.36460100	-0.62221800
C	12.66800300	2.52698900	0.01537800
H	13.25697700	2.37973900	0.93881400
H	12.74020000	3.60317500	-0.21868900
C	13.31128900	1.73423100	-1.12068200
H	13.31637400	0.65828600	-0.87390200
H	12.69015700	1.83235100	-2.02994500
C	14.73921800	2.17434300	-1.43513500
H	14.73866900	3.24741000	-1.69471900
H	15.35292000	2.08701800	-0.52156000
C	15.38408700	1.37585000	-2.56246600
H	16.41012500	1.71730300	-2.76545300
H	14.80876100	1.47194000	-3.49707800
H	15.43173000	0.30367200	-2.31365300

IX

C	7.43185200	-0.74566000	1.09922200
C	7.61525300	-2.25880900	1.08400600
C	6.27173600	-2.96491000	1.21491900
C	5.29520800	-2.59336200	0.07891400
C	5.17739400	-1.03103500	-0.00340200
C	4.23200000	-0.56353800	-1.11223200
C	4.11721400	0.92782300	-1.16429200
C	4.95679600	1.76189700	-0.52981800
C	6.11096700	1.25130400	0.31530400
C	6.50640100	-0.21835400	-0.01612200
C	7.26558100	2.25900700	0.26227200

C	6.81352200	3.63942400	0.72883500
C	5.58217400	4.10975200	-0.00660100
C	4.76598500	3.21447500	-0.58939700
C	5.30613000	5.59748000	-0.07424200
C	5.28139600	6.23519100	1.31924900
C	6.30140200	6.31622700	-0.99453200
C	3.89876600	-3.05222600	0.51927400
C	5.70733700	-3.29951100	-1.21977800
C	7.22710900	-0.26814400	-1.37309500
O	3.43971800	-2.85065800	1.61555000
O	3.21481400	-3.67190200	-0.44888000
H	6.99973400	-0.46104400	2.07516300
H	8.41287700	-0.24924200	1.03584900
H	8.13519800	-2.57392500	0.16422000
H	8.26947000	-2.55953200	1.91719600
H	5.80847700	-2.68357500	2.17315100
H	6.40520600	-4.05914200	1.23578200
H	4.69761300	-0.75763400	0.95279200
H	3.23051200	-1.00048600	-0.96410700
H	4.56464300	-0.93828200	-2.09704600
H	3.29830300	1.34930500	-1.75728600
H	5.74947200	1.24860300	1.36237900
H	7.63708500	2.34440100	-0.77182200
H	8.10704100	1.91477300	0.88155400
H	7.63272400	4.36535200	0.59888800
H	6.60443900	3.62042400	1.81443200
H	3.88188900	3.56909000	-1.13090700
H	4.30282400	5.71886200	-0.51610300
H	4.99822900	7.29718800	1.25629000
H	6.27090300	6.18669400	1.80148300
H	4.55915700	5.72761800	1.97640000
H	6.04998900	7.38492600	-1.08010200
H	7.33091900	6.25131100	-0.60752400
H	6.29133200	5.87951100	-2.00409300
H	5.09677100	-2.99576400	-2.07885100
H	6.75964800	-3.10840000	-1.46144700
H	5.58970500	-4.38663000	-1.10717800
H	7.42518300	-1.29396500	-1.70424700
H	8.19972700	0.24164000	-1.31167900
H	6.64302600	0.23042400	-2.16070200
C	1.86882900	-4.04681600	-0.16329600
H	1.64350600	-4.86344600	-0.86361100
H	1.80805500	-4.43042100	0.86604000
C	0.91247800	-2.88215000	-0.35703600
H	1.20406900	-2.06966500	0.32795900
H	1.02386900	-2.49661500	-1.38473200
C	-0.53981700	-3.27265200	-0.10041700
H	-0.63839000	-3.65768600	0.92993400
H	-0.82265700	-4.10847100	-0.76553900
C	-1.51692700	-2.11779800	-0.30096000
H	-1.22844300	-1.27992600	0.35820200
H	-1.42227900	-1.73531500	-1.33301100
C	-2.97085800	-2.49470900	-0.03317300
H	-3.06495000	-2.87306100	1.00052600
H	-3.25671300	-3.33706200	-0.68859300
C	-3.94905100	-1.34186800	-0.23897800
H	-3.66104900	-0.49835900	0.41377900
H	-3.85682600	-0.96550600	-1.27368400
C	-5.40295200	-1.71637400	0.03341200
H	-5.68976500	-2.56251900	-0.61665900

H	-5.49547100	-2.08937500	1.06928100
C	-6.38214300	-0.56518600	-0.17699100
H	-6.09399800	0.28192800	0.47114000
H	-6.29119500	-0.19388100	-1.21365300
C	-7.83567700	-0.93877600	0.09871400
H	-7.92697200	-1.30731000	1.13631800
H	-8.12291600	-1.78791200	-0.54725200
C	-8.81559500	0.21107500	-0.11563500
H	-8.72555200	0.57821500	-1.15386300
H	-8.52734000	1.06099500	0.52880400
C	-10.26881600	-0.16187100	0.16261700
H	-10.55638000	-1.01330600	-0.48017000
H	-10.35914300	-0.52686800	1.20156700
C	-11.24917400	0.98703300	-0.05483600
H	-10.96072000	1.83901400	0.58681000
H	-11.15967700	1.35091000	-1.09426600
C	-12.70220700	0.61464700	0.22531300
H	-12.79160800	0.25215400	1.26525500
H	-12.98991800	-0.23845800	-0.41523900
C	-13.68303900	1.76270000	0.00582600
H	-13.39465600	2.61641900	0.64555400
H	-13.59414900	2.12467900	-1.03453700
C	-15.13692600	1.39130300	0.28722200
H	-15.42194400	0.53735600	-0.35177600
H	-15.22284500	1.02998300	1.32686200
C	-16.10989900	2.54377300	0.06439500
H	-17.14744800	2.24480600	0.27565300
H	-16.07046700	2.90160900	-0.97680600
H	-15.86909500	3.39889500	0.71591300

X

C	-7.52609500	-2.24593500	0.02834800
C	-6.79631400	-3.56618600	0.24645200
C	-5.41446700	-3.52890000	-0.39374100
C	-4.53024100	-2.39707300	0.17061600
C	-5.30764500	-1.03863600	0.06396800
C	-4.51065300	0.14812400	0.61041400
C	-5.26149600	1.43729000	0.48951700
C	-6.57435000	1.51221900	0.21780600
C	-7.42140000	0.27072100	-0.00149100
C	-6.77561400	-1.01929600	0.58559100
C	-8.85646600	0.54424100	0.46463000
C	-9.45045500	1.74883500	-0.25893600
C	-8.55677000	2.96154500	-0.16508300
C	-7.24297400	2.80946000	0.07598500
C	-9.17873900	4.33177400	-0.33747900
C	-9.96519200	4.43879200	-1.64860800
C	-10.05273900	4.71129000	0.86512800
C	-3.34501800	-2.23186500	-0.79058700
C	-4.03069300	-2.77111500	1.57309200
C	-6.86646300	-0.99206600	2.12004700
O	-3.44405500	-2.21311800	-1.99129800
O	-2.17478800	-2.06765000	-0.16636600
H	-7.66638400	-2.10537600	-1.05827800
H	-8.53418800	-2.29806900	0.46869000
H	-6.72365300	-3.79212200	1.32318200
H	-7.38152500	-4.38840000	-0.19417100
H	-5.52361100	-3.37233000	-1.47791200
H	-4.89679100	-4.49360500	-0.26339800

H	-5.40084400	-0.87859000	-1.02474400
H	-3.54878200	0.23494600	0.07768800
H	-4.23693000	-0.01565200	1.66834500
H	-4.69633000	2.36429100	0.63491400
H	-7.47427800	0.12228400	-1.09793900
H	-8.86279100	0.74987800	1.54737200
H	-9.49084700	-0.33898600	0.29874200
H	-10.44645900	1.97920900	0.15315400
H	-9.62031200	1.50291400	-1.32348500
H	-6.60656700	3.69801200	0.15373700
H	-8.34978700	5.05793200	-0.38378100
H	-10.33917500	5.46376600	-1.79547200
H	-10.83839300	3.76680100	-1.65009200
H	-9.33566000	4.17793400	-2.51274000
H	-10.44802700	5.73281600	0.75159500
H	-10.91630600	4.03490800	0.96814700
H	-9.47605800	4.66861300	1.80088800
H	-3.47701000	-1.95714800	2.05677600
H	-4.86046100	-3.05959900	2.22922900
H	-3.35020300	-3.63219500	1.50936400
H	-6.32366200	-1.82213700	2.58648100
H	-7.91420600	-1.07161100	2.44497300
H	-6.46384400	-0.05534400	2.53297900
C	-1.03251200	-1.84202200	-0.98718100
H	-0.88545100	-2.70916300	-1.65144300
H	-1.21942400	-0.97109600	-1.63646900
C	0.16217800	-1.62031900	-0.08379100
H	-0.04426400	-0.76112600	0.57607500
H	0.28071500	-2.49738000	0.57402000
C	1.44833100	-1.37872600	-0.86821700
H	1.31349400	-0.51252800	-1.54030400
H	1.64615800	-2.24328000	-1.52667900
C	2.66251000	-1.13782600	0.02415800
H	2.46789600	-0.26801800	0.67669500
H	2.79284400	-1.99957900	0.70283500
C	3.95499700	-0.90736400	-0.75315300
H	3.82150000	-0.05081400	-1.43807900
H	4.15235300	-1.78098900	-1.40005600
C	5.16768100	-0.65560500	0.13807700
H	4.97262200	0.22253800	0.77959600
H	5.29660200	-1.50856200	0.82840500
C	6.46302900	-0.43541100	-0.63774600
H	6.66108700	-1.31648200	-1.27432800
H	6.33240600	0.41340100	-1.33285500
C	7.67393900	-0.17498000	0.25343900
H	7.47779100	0.70969800	0.88563300
H	7.80093200	-1.02088500	0.95279500
C	8.97131700	0.03691600	-0.52131900
H	8.84310100	0.87959300	-1.22433200
H	9.16988600	-0.84999800	-1.14961000
C	10.18066900	0.30406200	0.37000900
H	10.30618700	-0.53642200	1.07615200
H	9.98352300	1.19363100	0.99500700
C	11.47955000	0.50980600	-0.40389200
H	11.67846400	-0.38136900	-1.02603200
H	11.35313700	1.34794200	-1.11265500
C	12.68768500	0.78184200	0.48762200
H	12.48957500	1.67475100	1.10754400
H	12.81220200	-0.05486000	1.19843000
C	13.98760900	0.98356100	-0.28565100

H	13.86220000	1.81874200	-0.99812500
H	14.18655600	0.08956300	-0.90377300
C	15.19510900	1.25874900	0.60567600
H	14.99653400	2.15366800	1.22291400
H	15.31955600	0.42430000	1.31946300
C	16.49664300	1.45867100	-0.16694400
H	16.69263000	0.56343600	-0.78252900
H	16.36911800	2.29143700	-0.88040300
C	17.69729900	1.73434000	0.73136400
H	18.61821800	1.87344200	0.14563500
H	17.86916800	0.90161600	1.43189000
H	17.54301600	2.64430700	1.33291700

XI

C	6.01124100	-1.87756700	1.75900500
C	5.37985600	-3.26128600	1.86846600
C	3.99813600	-3.27497300	1.22500200
C	4.04596900	-2.88707200	-0.27117000
C	4.79043500	-1.52003700	-0.42255700
C	4.88626400	-1.05846700	-1.87729000
C	5.57520100	0.26442300	-2.00173600
C	6.28920900	0.82668100	-1.01284200
C	6.45328200	0.15969400	0.34244800
C	6.15696000	-1.36923200	0.30971400
C	7.81349800	0.54328800	0.93736700
C	7.95737900	2.05788900	1.05175700
C	7.66466300	2.75367900	-0.25539600
C	6.91062100	2.14345600	-1.18618300
C	8.24745100	4.13266100	-0.48546200
C	7.87970500	5.10150100	0.64377600
C	9.76639500	4.07711600	-0.69476400
C	2.60466300	-2.66587200	-0.74972900
C	4.61180000	-4.04824400	-1.09760700
C	7.30715800	-2.10389800	-0.39739300
O	2.05925100	-3.28267000	-1.62834300
O	1.98829100	-1.68898200	-0.07287800
H	5.37738200	-1.16511000	2.31693400
H	6.99390900	-1.87474300	2.25631000
H	6.03173500	-4.02012700	1.40597000
H	5.29302400	-3.54461000	2.92915200
H	3.35458700	-2.55706100	1.75728100
H	3.52684000	-4.26603800	1.32876900
H	4.13029600	-0.79892000	0.08699500
H	3.87650600	-0.98961700	-2.31811700
H	5.41677500	-1.80532200	-2.49477300
H	5.48783600	0.79096400	-2.95827000
H	5.68780700	0.61170500	1.00358000
H	8.62184000	0.16402000	0.29139300
H	7.94573400	0.08121300	1.92695700
H	8.97262800	2.31254500	1.39721800
H	7.27435200	2.44370300	1.83107500
H	6.71657200	2.65360700	-2.13629100
H	7.80114700	4.51925900	-1.41710600
H	8.24094100	6.11677300	0.41858800
H	8.33340300	4.79565300	1.60014400
H	6.78958200	5.15090900	0.78650800
H	10.16466300	5.07864000	-0.92051000
H	10.28666000	3.71232700	0.20530600
H	10.02528100	3.40892400	-1.52932100

H	4.70553000	-3.79307500	-2.16095100
H	5.59348700	-4.36912300	-0.73131800
H	3.93193900	-4.90894400	-1.03694400
H	7.09761200	-3.16988000	-0.54138100
H	8.23003700	-2.03683700	0.19707600
H	7.51932900	-1.66886100	-1.38518600
C	0.64858300	-1.38658800	-0.44574800
H	0.61726300	-1.13743300	-1.51901700
H	0.02148400	-2.28266300	-0.30784000
C	0.16804300	-0.23265100	0.40867300
H	0.25609000	-0.51432100	1.47129300
H	0.84020700	0.62836400	0.25680500
C	-1.27133700	0.16374000	0.09359700
H	-1.92463400	-0.71506200	0.22922300
H	-1.35357900	0.44173700	-0.97266800
C	-1.77324500	1.32208200	0.95364000
H	-1.07438600	2.16945300	0.84867200
H	-1.73224900	1.02667200	2.01749200
C	-3.18591100	1.79979600	0.61607100
H	-3.21874100	2.12605800	-0.43915600
H	-3.40821300	2.69915300	1.21556400
C	-4.28435800	0.76600900	0.85676700
H	-4.12170500	-0.11500000	0.21209500
H	-4.21423800	0.39667900	1.89615300
C	-5.68998500	1.30510500	0.60668500
H	-5.86760100	2.17965300	1.25815900
H	-5.75574000	1.68440000	-0.42897100
C	-6.79017000	0.27314800	0.83657400
H	-6.61354300	-0.60005200	0.18301100
H	-6.72158900	-0.10824000	1.87130100
C	-8.19705600	0.80988600	0.59001100
H	-8.26597300	1.19087000	-0.44482800
H	-8.37434300	1.68302900	1.24347700
C	-9.29559500	-0.22358800	0.82103600
H	-9.22455200	-0.60633500	1.85513000
H	-9.11916700	-1.09582100	0.16609000
C	-10.70351500	0.31199400	0.57643200
H	-10.88578000	1.17785600	1.23882200
H	-10.76218600	0.70293300	-0.45398500
C	-11.79580900	-0.73169500	0.80170200
H	-11.67420700	-1.15843800	1.81225500
H	-11.64416900	-1.57201000	0.10018200
C	-13.22439700	-0.20570400	0.65751800
H	-13.38686800	0.61169000	1.38332400
H	-13.92829900	-1.00614000	0.94385600
C	-13.58680300	0.28743600	-0.74191900
H	-12.94079600	1.13780800	-1.02135600
H	-13.37005200	-0.51023700	-1.47600300
C	-15.04706000	0.71322200	-0.87595000
H	-15.69832300	-0.13706500	-0.60816200
H	-15.26238500	1.50148900	-0.13357000
C	-15.40609300	1.21280600	-2.27088400
H	-16.46334200	1.51056600	-2.33500400
H	-15.23185300	0.43300100	-3.02933600
H	-14.79536300	2.08621500	-2.54997900

XII

C	-7.52596600	-2.24610800	0.02837200
C	-6.79604100	-3.56628500	0.24645900

C	-5.41420300	-3.52885200	-0.39373600
C	-4.53008900	-2.39693400	0.17059400
C	-5.30761600	-1.03856900	0.06390100
C	-4.51075400	0.14831300	0.61027800
C	-5.26178700	1.43737100	0.48946100
C	-6.57465800	1.51213400	0.21778200
C	-7.42153000	0.27052500	-0.00155100
C	-6.77558500	-1.01938100	0.58555400
C	-8.85663500	0.54382000	0.46456400
C	-9.45077900	1.74836700	-0.25896000
C	-8.55726500	2.96120600	-0.16504600
C	-7.24345300	2.80928600	0.07602100
C	-9.17940200	4.33136000	-0.33745600
C	-9.96593000	4.43825000	-1.64854600
C	-10.05337700	4.71083800	0.86518600
C	-3.34487400	-2.23165900	-0.79060300
C	-4.03050000	-2.77088900	1.57309000
C	-6.86640800	-0.99210800	2.12000400
O	-3.44390900	-2.21292000	-1.99131500
O	-2.17466400	-2.06734100	-0.16635900
H	-7.66632800	-2.10558100	-1.05824900
H	-8.53403000	-2.29832500	0.46876800
H	-6.72334700	-3.79221700	1.32318600
H	-7.38117800	-4.38855500	-0.19415400
H	-5.52335800	-3.37230600	-1.47791000
H	-4.89642200	-4.49350400	-0.26340600
H	-5.40086100	-0.87857700	-1.02481600
H	-3.54894600	0.23528200	0.07746400
H	-4.23688600	-0.01543100	1.66817600
H	-4.69674300	2.36444200	0.63489100
H	-7.47437000	0.12206500	-1.09799800
H	-8.86299800	0.74939900	1.54731900
H	-9.49089100	-0.33949000	0.29862800
H	-10.44682500	1.97857500	0.15312000
H	-9.62058800	1.50246500	-1.32352000
H	-6.60715800	3.69791600	0.15380900
H	-8.35053100	5.05760600	-0.38382300
H	-10.34003200	5.46317800	-1.79542600
H	-10.83905800	3.76616400	-1.64996700
H	-9.33641600	4.17743300	-2.51270300
H	-10.44876700	5.73232100	0.75163700
H	-10.91687600	4.03438100	0.96827200
H	-9.47664200	4.66824800	1.80091600
H	-3.47686100	-1.95687100	2.05673300
H	-4.86024800	-3.05939000	2.22924600
H	-3.34996900	-3.63194000	1.50939100
H	-6.32360800	-1.82218600	2.58643300
H	-7.91414500	-1.07163600	2.44495700
H	-6.46376800	-0.05538800	2.53291700
C	-1.03239500	-1.84162900	-0.98716000
H	-0.88530400	-2.70872300	-1.65147600
H	-1.21933800	-0.97067500	-1.63640300
C	0.16228900	-1.61993900	-0.08376000
H	-0.04415700	-0.76076200	0.57612600
H	0.28081800	-2.49701500	0.57403200
C	1.44845000	-1.37834400	-0.86816900
H	1.31359700	-0.51220200	-1.54032400
H	1.64632600	-2.24293500	-1.52656700
C	2.66260100	-1.13734500	0.02421600
H	2.46799000	-0.26743900	0.67662300

H	2.79288300	-1.99900200	0.70302500
C	3.95512200	-0.90703900	-0.75308100
H	3.82165900	-0.05062600	-1.43818600
H	4.15250100	-1.78079000	-1.39980700
C	5.16777200	-0.65511500	0.13814700
H	4.97273500	0.22320800	0.77942600
H	5.29660200	-1.50789700	0.82870900
C	6.46317200	-0.43520800	-0.63766700
H	6.66123100	-1.31648000	-1.27397200
H	6.33262200	0.41339300	-1.33304800
C	7.67404600	-0.17455500	0.25350000
H	7.47793300	0.71036400	0.88536800
H	7.80092200	-1.02021900	0.95317100
C	8.97148800	0.03695400	-0.52125300
H	8.84337200	0.87936400	-1.22460400
H	9.17003800	-0.85021400	-1.14919100
C	10.18080300	0.30436500	0.37004400
H	10.30618900	-0.53582700	1.07655800
H	9.98370400	1.19421900	0.99464900
C	11.47975600	0.50965000	-0.40385700
H	11.67863400	-0.38181800	-1.02558800
H	11.35346400	1.34747700	-1.11300700
C	12.68785600	0.78198100	0.48761200
H	12.48980000	1.67520600	1.10709600
H	12.81222800	-0.05439300	1.19883400
C	13.98785600	0.98318800	-0.28566300
H	13.86258500	1.81803200	-0.99855600
H	14.18675300	0.08887100	-0.90333900
C	15.19532200	1.25868300	0.60561300
H	14.99680700	2.15393500	1.22238800
H	15.31961700	0.42458200	1.31983400
C	16.49693600	1.45806200	-0.16701100
H	16.69286500	0.56249300	-0.78212800
H	16.36956000	2.29047700	-0.88090600
C	17.69755600	1.73405000	0.73124300
H	18.61853800	1.87272400	0.14551400
H	17.86926200	0.90167300	1.43222200
H	17.54334400	2.64435100	1.33230600

XIII

C	6.68534300	-1.80027000	-0.45086400
C	6.24148200	-3.20376000	-0.84648700
C	4.93820200	-3.57180600	-0.14885100
C	3.79010800	-2.59718000	-0.48567300
C	4.26316800	-1.12949200	-0.19702400
C	3.19026300	-0.08486900	-0.51282500
C	3.65280300	1.30743200	-0.21629500
C	4.93568500	1.63398900	0.00872100
C	6.04440800	0.59603400	-0.01133200
C	5.64804600	-0.70478500	-0.77078700
C	7.34695000	1.25089900	-0.48679400
C	7.72145100	2.43696800	0.39679300
C	6.58307200	3.41783100	0.53884700
C	5.31831400	3.01218800	0.33120000
C	6.90667200	4.85094000	0.90708600
C	7.74661200	4.93346500	2.18646400
C	7.58696900	5.59291200	-0.25082200
C	2.67575700	-2.84459800	0.54042200
C	3.27475400	-2.86451100	-1.90665200

C	5.61190500	-0.43191500	-2.28335800
O	2.86183100	-2.97964800	1.72321700
O	1.45246900	-2.85764400	0.00235300
H	6.87623900	-1.79334000	0.63711200
H	7.64375800	-1.55961400	-0.93708400
H	6.13490400	-3.27909000	-1.94131500
H	7.02226400	-3.92939200	-0.56991700
H	5.09518900	-3.55521900	0.94063100
H	4.63136500	-4.59829500	-0.40921900
H	4.40303900	-1.11363100	0.89830000
H	2.27496000	-0.29614500	0.06514100
H	2.88013400	-0.14775400	-1.57155400
H	2.89192900	2.09483900	-0.18569400
H	6.21197900	0.30275000	1.04361700
H	7.22605800	1.61102500	-1.52139300
H	8.16746300	0.51825000	-0.49235900
H	8.60962700	2.94407400	-0.01416400
H	8.02192700	2.07994800	1.39921700
H	4.50154800	3.73645600	0.42564700
H	5.94664400	5.35731500	1.10337400
H	7.90124300	5.98220200	2.48350200
H	8.74151700	4.48179000	2.04446500
H	7.25361300	4.41148800	3.02048300
H	7.76000000	6.64822800	0.01189900
H	8.56603200	5.15102600	-0.49587100
H	6.96554000	5.56268800	-1.15799300
H	2.52048200	-2.13605000	-2.22823600
H	4.09291700	-2.85699700	-2.63659500
H	2.80535400	-3.85759100	-1.95246300
H	5.22316400	-1.28371700	-2.85306800
H	6.62408000	-0.22840700	-2.66253000
H	4.98861200	0.44230700	-2.52271500
C	0.35605100	-3.01383600	0.89842400
H	0.45068900	-3.97799200	1.42399500
H	0.40218500	-2.22558400	1.66756200
C	-0.92529600	-2.93741500	0.09542300
H	-0.95712500	-1.97034700	-0.43374100
H	-0.90706800	-3.71838400	-0.68288400
C	-2.16805900	-3.09662300	0.96606600
H	-2.16760800	-2.32268400	1.75423700
H	-2.12610500	-4.06546600	1.49493500
C	-3.47176600	-3.00958400	0.17788200
H	-3.51430600	-2.03960800	-0.34905800
H	-3.47249800	-3.78130100	-0.61246500
C	-4.71895800	-3.16992500	1.04179800
H	-4.71495800	-2.40027100	1.83453500
H	-4.67763300	-4.14128800	1.56653000
C	-6.02342900	-3.07400500	0.25599900
H	-6.04832500	-2.10637500	-0.27521000
H	-6.03606400	-3.84964100	-0.53126600
C	-7.26802000	-3.21779200	1.12818000
H	-7.19648600	-4.15732200	1.70267500
H	-7.27506500	-2.41146800	1.88240300
C	-8.58934200	-3.21923300	0.35505100
H	-8.62888500	-4.11855700	-0.28361400
H	-9.41835200	-3.32322300	1.07620400
C	-8.84349700	-1.98803800	-0.51862500
H	-9.82724600	-2.10092300	-1.00578600
H	-8.10941400	-1.95417200	-1.34280600
C	-8.81177000	-0.65939600	0.23252300

H	-7.80912000	-0.49816900	0.66610800
H	-9.50598000	-0.70739100	1.09124400
C	-9.16803700	0.54003700	-0.64093300
H	-8.48218200	0.57565300	-1.50659500
H	-10.17826900	0.39707500	-1.06485400
C	-9.11515400	1.87363600	0.09851800
H	-9.79908400	1.83728900	0.96553500
H	-8.10394500	2.01658200	0.52039700
C	-9.47080400	3.07378900	-0.77410200
H	-10.48290400	2.93215400	-1.19412900
H	-8.78810700	3.10824000	-1.64223700
C	-9.41386000	4.40758300	-0.03522100
H	-10.09643900	4.37316700	0.83320600
H	-8.40157200	4.54887800	0.38503100
C	-9.76902600	5.60920200	-0.90748400
H	-9.08688700	5.64022200	-1.77496900
H	-10.78045400	5.46559700	-1.32601400
C	-9.70819200	6.93745000	-0.16146300
H	-9.96929900	7.78227700	-0.81612500
H	-8.69828400	7.12335900	0.23756400
H	-10.40603300	6.94693200	0.69096300

XIV

C	5.92807300	-2.99653700	0.42694300
C	4.77316300	-3.99004200	0.47539300
C	3.54400800	-3.35413200	1.11212300
C	3.06239400	-2.10153800	0.34981700
C	4.26619700	-1.10991900	0.18165100
C	3.88688800	0.16384700	-0.57701000
C	5.04247700	1.10553700	-0.71244500
C	6.31752200	0.75889600	-0.47329500
C	6.69979800	-0.64233000	-0.02920000
C	5.60552400	-1.70355600	-0.34912500
C	8.10010400	-0.97911500	-0.55572200
C	9.12731100	0.04498200	-0.08277900
C	8.70096300	1.45896600	-0.39446000
C	7.40070700	1.74048400	-0.58782900
C	9.76767500	2.52996500	-0.49061500
C	10.64010700	2.57857200	0.76857300
C	10.62482800	2.36297200	-1.75215500
C	2.08741100	-1.36115000	1.27574000
C	2.35464800	-2.51630400	-0.94752400
C	5.58051900	-1.98357100	-1.86061600
O	2.28631200	-1.16021000	2.44705200
O	0.99417800	-0.91622000	0.64893500
H	6.19207600	-2.72452100	1.46448000
H	6.81896800	-3.47842100	-0.00542500
H	4.54250100	-4.36148900	-0.53676500
H	5.07152300	-4.87464700	1.05943900
H	3.78582200	-3.05703100	2.14418400
H	2.71684700	-4.08026200	1.17678100
H	4.49384400	-0.80152700	1.21730500
H	3.05652900	0.67687700	-0.06332000
H	3.49498500	-0.07860300	-1.58133900
H	4.82177800	2.13151400	-1.02633600
H	6.78431700	-0.60414500	1.07461900
H	8.09195500	-0.98192900	-1.65782900
H	8.40242600	-1.98580500	-0.23122100
H	10.10602200	-0.16588200	-0.54393600

H	9.28577400	-0.05589400	1.00686000
H	7.10517300	2.76844800	-0.82557700
H	9.24526200	3.49784600	-0.57376600
H	11.35367500	3.41522900	0.71548200
H	11.22646000	1.65338600	0.88783300
H	10.02678600	2.70979200	1.67285200
H	11.35449800	3.18327600	-1.83788700
H	11.19289600	1.41912900	-1.73434700
H	9.99931000	2.36430000	-2.65701500
H	2.07369600	-1.65779900	-1.56967100
H	2.98156600	-3.18567200	-1.54851700
H	1.42942000	-3.06059500	-0.71032500
H	4.74953500	-2.63622200	-2.15164800
H	6.50723900	-2.48588500	-2.17451900
H	5.49565200	-1.05502000	-2.44413900
C	0.06776400	-0.16282600	1.42584500
H	-0.30047200	-0.78582200	2.25711700
H	0.58925400	0.69650600	1.87825800
C	-1.05932200	0.28165400	0.51775800
H	-0.63730900	0.87513200	-0.31043600
H	-1.52165100	-0.60853600	0.05964900
C	-2.11605400	1.09618800	1.25781900
H	-1.64121700	1.97554500	1.72838800
H	-2.52904500	0.49615000	2.08821900
C	-3.25714200	1.56250400	0.35841800
H	-2.84494100	2.16689400	-0.46925700
H	-3.72884100	0.68391600	-0.11687200
C	-4.32174500	2.37149100	1.09307000
H	-3.84849000	3.24586000	1.57486000
H	-4.73799900	1.76382300	1.91665500
C	-5.45946800	2.84431000	0.19283300
H	-5.04771200	3.46650200	-0.62260100
H	-5.91271500	1.96584900	-0.29903600
C	-6.53388800	3.63261200	0.93766800
H	-6.98331800	2.98688700	1.71237300
H	-6.05424700	4.46096200	1.48691600
C	-7.63128400	4.21389700	0.04249400
H	-7.18429600	4.97160200	-0.62410200
H	-8.35212700	4.75701100	0.67775800
C	-8.39218600	3.19837100	-0.81379700
H	-7.71272400	2.75293100	-1.56154200
H	-9.15849000	3.73696000	-1.39737900
C	-9.06701300	2.08008100	-0.02349600
H	-9.70662100	2.52161400	0.76237000
H	-8.30480200	1.48474200	0.50929800
C	-9.90350300	1.14425000	-0.89126600
H	-10.67951900	1.72901800	-1.41717700
H	-9.26400500	0.71454900	-1.68337400
C	-10.56686200	0.01338400	-0.11076200
H	-9.79039200	-0.57238500	0.41356800
H	-11.20474100	0.44314900	0.68247300
C	-11.40337200	-0.92228100	-0.97857700
H	-10.76502800	-1.35174300	-1.77167200
H	-12.17958300	-0.33636800	-1.50302400
C	-12.06709200	-2.05323500	-0.19842900
H	-11.29089400	-2.63890600	0.32674700
H	-12.70584600	-1.62368600	0.59450500
C	-12.90353200	-2.99015700	-1.06641700
H	-13.67712900	-2.40251800	-1.59083000
H	-12.26323600	-3.41734800	-1.85783200

C	-13.56345100	-4.11666900	-0.27902900
H	-14.15782700	-4.77317300	-0.93188500
H	-14.23673400	-3.71814600	0.49657600
H	-12.81076000	-4.74159300	0.22755300

XV

C	6.82684800	-1.06386800	1.62036900
C	6.54581100	-2.52587400	1.94718800
C	5.05117100	-2.75889300	2.12759400
C	4.23824300	-2.38828300	0.86943800
C	4.59679000	-0.92191700	0.44234100
C	3.84274400	-0.46442700	-0.80837300
C	4.18464300	0.94151900	-1.19141400
C	5.23397700	1.61498700	-0.69305800
C	6.17525800	0.99664600	0.32596100
C	6.10935200	-0.55930400	0.35185600
C	7.58096000	1.58081100	0.14081900
C	7.56557100	3.10163900	0.26117300
C	6.53776500	3.73045800	-0.64789100
C	5.49191300	3.00524600	-1.08100100
C	6.72443200	5.17677900	-1.05704700
C	6.88513300	6.09559500	0.15913900
C	7.89558000	5.34194000	-2.03437400
C	2.76509300	-2.30884100	1.29272600
C	4.42499800	-3.46229200	-0.21129000
C	6.78773700	-1.12823600	-0.90495800
O	2.37643400	-1.76346800	2.29442800
O	1.93002100	-2.87265600	0.41477500
H	6.49534400	-0.44977200	2.47661900
H	7.91213600	-0.90381300	1.52355300
H	6.95204100	-3.18111000	1.15892200
H	7.07413500	-2.80532700	2.87208200
H	4.68861000	-2.14359400	2.96536900
H	4.84842600	-3.80919500	2.39477200
H	4.21695700	-0.30887000	1.27870300
H	2.75495700	-0.54332500	-0.64408300
H	4.05193000	-1.13551900	-1.66095700
H	3.53476400	1.43666200	-1.92104900
H	5.82465500	1.34052700	1.31887000
H	7.96590700	1.31351800	-0.85667500
H	8.27569800	1.15664500	0.88072900
H	8.56661300	3.50502200	0.03720300
H	7.35544700	3.39479700	1.30639600
H	4.75917400	3.46944100	-1.75047800
H	5.80720100	5.48170900	-1.58838700
H	6.93545200	7.14992700	-0.15365400
H	7.81130700	5.87222700	0.71258100
H	6.03983100	5.98592600	0.85521400
H	7.97826700	6.38785100	-2.36885200
H	8.85540900	5.07028100	-1.56660800
H	7.76054400	4.70737600	-2.92271100
H	3.94382400	-3.19670500	-1.16049500
H	5.48711700	-3.65292700	-0.40587700
H	3.97913400	-4.40979900	0.12345700
H	6.66509200	-2.21388400	-0.99153700
H	7.86912400	-0.92885000	-0.88277700
H	6.38768400	-0.67262700	-1.82279700
C	0.53700400	-2.77622600	0.69683200
H	0.32592900	-3.26830200	1.66015300

H	0.26477400	-1.71452000	0.81474600
C	-0.22176300	-3.42488200	-0.44155000
H	0.05111000	-2.92134900	-1.38405000
H	0.10807100	-4.47247400	-0.54088700
C	-1.73321600	-3.36791700	-0.24029200
H	-2.04077100	-2.31546900	-0.11673900
H	-2.00007200	-3.87530700	0.70434500
C	-2.51187600	-4.00530200	-1.38957000
H	-2.14339300	-5.03462000	-1.53881000
H	-2.28286100	-3.46396800	-2.32508700
C	-4.02678400	-4.04614300	-1.18726700
H	-4.25777500	-4.62047600	-0.27194500
H	-4.47952300	-4.61226800	-2.01924500
C	-4.69796800	-2.67657200	-1.10340800
H	-4.31544800	-2.12126100	-0.22959400
H	-4.41555300	-2.07835000	-1.98885400
C	-6.21931800	-2.75254300	-1.01010100
H	-6.61291600	-3.29272800	-1.88990600
H	-6.50038100	-3.36296200	-0.13308000
C	-6.89524000	-1.38813200	-0.91193600
H	-6.50491100	-0.85049900	-0.02909200
H	-6.60955900	-0.77592200	-1.78624300
C	-8.41676100	-1.46188000	-0.82455600
H	-8.70270800	-2.07396100	0.04976000
H	-8.80743100	-1.99938100	-1.70735300
C	-9.09117800	-0.09654100	-0.72473500
H	-8.81390200	0.51347300	-1.60375000
H	-8.68663700	0.43731200	0.15239200
C	-10.61347200	-0.17754900	-0.62955600
H	-10.99007400	-0.77290000	-1.47917700
H	-10.89012600	-0.74192100	0.27939800
C	-11.32695600	1.17494600	-0.62024200
H	-11.07879100	1.72450600	-1.54633500
H	-12.41647700	1.00248700	-0.65596900
C	-11.00972400	2.05677400	0.58596100
H	-9.93357700	2.30134400	0.60132500
H	-11.20320900	1.48715100	1.51323200
C	-11.81056000	3.35550700	0.61485900
H	-11.62553500	3.91831600	-0.31806900
H	-12.89025000	3.11969100	0.61310800
C	-11.49168900	4.24712200	1.81238700
H	-11.67376700	3.68124400	2.74267400
H	-10.41313200	4.48273400	1.81060500
C	-12.29747000	5.54121900	1.83519400
H	-12.04485800	6.16044500	2.70883900
H	-13.37910900	5.33544400	1.87271300
H	-12.10782600	6.14350400	0.93241700

XVI

C	-6.67668800	-2.32159600	-1.35009300
C	-5.77843500	-3.55265500	-1.32016100
C	-4.31136900	-3.14954900	-1.39749900
C	-3.88931100	-2.22440900	-0.23662600
C	-4.87275700	-1.00396100	-0.17118800
C	-4.53899800	-0.03087000	0.96174600
C	-5.47635800	1.13552500	0.99878500
C	-6.63835300	1.17865100	0.32724200
C	-7.10348700	0.02842800	-0.54892500
C	-6.39811700	-1.31876400	-0.21200800

C	-8.63598400	-0.02595900	-0.54677200
C	-9.23449800	1.29683300	-1.01533500
C	-8.68250700	2.47148400	-0.24481200
C	-7.49434800	2.36814600	0.37561000
C	-9.50064800	3.74477600	-0.18579800
C	-9.87337300	4.24674200	-1.58515400
C	-10.74749400	3.57572900	0.69219200
C	-2.54067900	-1.60318500	-0.62551000
C	-3.74882700	-3.03714500	1.05786600
C	-6.93358900	-1.86542500	1.12140600
O	-2.29462900	-1.14496500	-1.71245600
O	-1.65829800	-1.57862800	0.37785100
H	-6.52270200	-1.80632700	-2.31496000
H	-7.73397700	-2.62887000	-1.32597100
H	-5.97375500	-4.14916200	-0.41368000
H	-6.02301100	-4.20868300	-2.17013600
H	-4.13339700	-2.61693600	-2.34436400
H	-3.66101900	-4.03982900	-1.40736500
H	-4.67828800	-0.46444800	-1.11487600
H	-3.50447900	0.33588800	0.85275900
H	-4.55818400	-0.54472700	1.93978900
H	-5.18668600	1.99489700	1.61314100
H	-6.80377800	0.28742600	-1.58339500
H	-8.99921000	-0.23146100	0.47328500
H	-8.99407800	-0.84389000	-1.18926300
H	-10.33234000	1.26483700	-0.92232000
H	-9.03289100	1.44080200	-2.09293100
H	-7.10996100	3.22380300	0.94190400
H	-8.86652100	4.51302300	0.28778800
H	-10.39452300	5.21463800	-1.52534500
H	-10.54645700	3.54208400	-2.09937000
H	-8.97852000	4.37892100	-2.21194600
H	-11.29813600	4.52597400	0.77270200
H	-11.44062100	2.82932300	0.27229500
H	-10.47425400	3.25050300	1.70689200
H	-3.54122400	-2.40833100	1.93227700
H	-4.65323900	-3.62236400	1.26236000
H	-2.91461400	-3.74697000	0.96428700
H	-6.38469100	-2.75111200	1.46124000
H	-7.98802900	-2.16158500	1.02083400
H	-6.87809700	-1.11115500	1.92018100
C	-0.40351200	-0.95673700	0.11673000
H	0.09531800	-1.47972300	-0.71530300
H	-0.57611500	0.07991300	-0.21616600
C	0.42265500	-1.00660500	1.38454000
H	-0.13337100	-0.50362400	2.19329300
H	0.53967900	-2.05861400	1.69393400
C	1.79332400	-0.35916500	1.21036500
H	1.66775800	0.69451100	0.90211000
H	2.32414200	-0.85666900	0.38079300
C	2.64395200	-0.41280000	2.47804500
H	2.06691800	0.02540000	3.31024700
H	2.81642200	-1.46881800	2.75326800
C	3.98778900	0.30887300	2.37405600
H	4.47318800	0.29715000	3.36498200
H	3.81274400	1.37399700	2.13756000
C	4.95330900	-0.28089500	1.34784300
H	5.08176100	-1.36035900	1.54758500
H	4.51811100	-0.21089300	0.33595100
C	6.32012200	0.39793700	1.34310400

H	6.18890600	1.47906400	1.15673500
H	6.77067500	0.31964000	2.34890800
C	7.28607100	-0.17755500	0.31165600
H	7.41525100	-1.25919000	0.49656300
H	6.83617400	-0.09695700	-0.69425800
C	8.65341200	0.49965900	0.30729900
H	9.10344800	0.41944900	1.31319900
H	8.52405600	1.58121200	0.12205600
C	9.61915100	-0.07599600	-0.72427100
H	9.16669700	-0.00055800	-1.72947400
H	9.75242000	-1.15659300	-0.53604400
C	10.98474300	0.60522700	-0.73286200
H	10.85383000	1.68401700	-0.93476100
H	11.42282800	0.53855100	0.27793100
C	11.94952800	0.01524200	-1.75962600
H	11.46069300	0.02190300	-2.74913100
H	12.12804100	-1.04860000	-1.51954000
C	13.29145400	0.74024900	-1.86847900
H	13.11428700	1.79460200	-2.14822600
H	13.86608000	0.30070400	-2.70200100
C	14.14849300	0.69470400	-0.60499800
H	13.62265900	1.19669300	0.22571400
H	14.27620700	-0.35717500	-0.28966500
C	15.52161200	1.33933800	-0.77979100
H	16.05743700	0.83318500	-1.60170700
H	15.38977000	2.38547400	-1.10702000
C	16.37272000	1.30260800	0.48457300
H	17.35458600	1.77306500	0.32597300
H	16.54805400	0.26656300	0.81565200
H	15.87744200	1.83387300	1.31295100

XVII

C	-7.34977500	-1.06908900	-1.02945400
C	-7.42366600	-2.59139600	-1.00724300
C	-6.03517900	-3.19958800	-1.15958800
C	-5.06829200	-2.75428500	-0.04205800
C	-5.06166700	-1.18713200	0.03384400
C	-4.13260800	-0.64799900	1.12389200
C	-4.12560500	0.84792700	1.17042400
C	-5.03435400	1.61668100	0.54894000
C	-6.16319800	1.02080800	-0.27415700
C	-6.44539700	-0.47228100	0.06804000
C	-7.38663500	1.94253600	-0.20302000
C	-7.04404400	3.35036900	-0.68090000
C	-5.83719000	3.91119500	0.03132500
C	-4.94820100	3.07944200	0.60157300
C	-5.66813200	5.41521700	0.09070000
C	-5.71529300	6.04858600	-1.30418100
C	-6.69523400	6.06342400	1.02825900
C	-3.65005300	-3.11299100	-0.50526000
C	-5.40558300	-3.48353100	1.26548100
C	-7.13651500	-0.56853600	1.43785600
O	-3.22673600	-2.88721100	-1.61113200
O	-2.90490800	-3.67211900	0.45462200
H	-6.95621100	-0.75786800	-2.01354800
H	-8.36257100	-0.64382400	-0.95013700
H	-7.90405100	-2.93960200	-0.07792800
H	-8.06856600	-2.94143600	-1.82835900
H	-5.61013900	-2.88959500	-2.12663100

H	-6.08989800	-4.30065200	-1.17511900
H	-4.61997000	-0.88393900	-0.93168900
H	-3.10485600	-1.01120700	0.95780800
H	-4.41831800	-1.04252500	2.11569400
H	-3.32903600	1.32977000	1.74767600
H	-5.82102800	1.04031900	-1.32750900
H	-7.74512300	2.00482400	0.83730800
H	-8.21170700	1.53585200	-0.80630600
H	-7.91132200	4.01547700	-0.53811900
H	-6.85329000	3.34259500	-1.77000200
H	-4.08268400	3.49913200	1.12630000
H	-4.66814500	5.61002700	0.51342300
H	-5.50782300	7.12833000	-1.24847900
H	-6.70761300	5.92790600	-1.76754700
H	-4.97095800	5.59202300	-1.97397600
H	-6.51983400	7.14771700	1.10703800
H	-7.72448900	5.92339200	0.66097400
H	-6.63476900	5.63185000	2.03826700
H	-4.80412100	-3.13318200	2.11312900
H	-6.46468800	-3.36902800	1.52510800
H	-5.21077300	-4.55959600	1.15346500
H	-7.25118300	-1.60444800	1.77685000
H	-8.14540100	-0.13300600	1.39172100
H	-6.57742900	-0.02334300	2.21262300
C	-1.53930800	-3.94617600	0.14828700
H	-1.24085300	-4.73765400	0.85013000
H	-1.46673100	-4.33235400	-0.87931700
C	-0.67139800	-2.71060700	0.31786700
H	-1.03294200	-1.92921600	-0.36992800
H	-0.79779500	-2.32449700	1.34361000
C	0.80295900	-2.99166500	0.04432000
H	0.91647600	-3.38439500	-0.98158500
H	1.15958800	-3.79206100	0.71747700
C	1.68934900	-1.76085500	0.21203000
H	1.32893200	-0.96068400	-0.45848800
H	1.57647200	-1.36736400	1.23809100
C	3.16534100	-2.02790200	-0.06690200
H	3.27712300	-2.42226500	-1.09282500
H	3.52505600	-2.82888300	0.60383600
C	4.05064100	-0.79619400	0.09878100
H	3.69032200	0.00432300	-0.57197500
H	3.93815900	-0.40128600	1.12453000
C	5.52726800	-1.06078700	-0.18016900
H	5.88777900	-1.86112400	0.49089500
H	5.63957100	-1.45612400	-1.20574700
C	6.41210200	0.17141400	-0.01551900
H	6.05132500	0.97162200	-0.68650600
H	6.29974000	0.56684800	1.01005900
C	7.88881100	-0.09253900	-0.29469400
H	8.00117300	-0.48727400	-1.32054000
H	8.24927300	-0.89338900	0.37580300
C	8.77386900	1.13935000	-0.12934100
H	8.66429600	1.53201400	0.89764600
H	8.41112500	1.94149800	-0.79704400
C	10.25021600	0.87582600	-0.41209500
H	10.60001400	0.06361200	0.24819800
H	10.36273100	0.49584500	-1.44391100
C	11.13147300	2.11039800	-0.23225200
H	11.06483400	2.45345100	0.81614900
H	10.71924500	2.93153700	-0.84374600

C	12.60075800	1.90853700	-0.60481000
H	13.12119000	2.87947100	-0.53684200
H	12.66932000	1.60396200	-1.66492200
C	13.34616600	0.89177700	0.25747500
H	13.22743300	1.16177800	1.32293200
H	12.88672900	-0.10577800	0.14636500
C	14.83308500	0.78897200	-0.07435200
H	14.94735300	0.53214600	-1.14198700
H	15.30083300	1.78158900	0.04714700
C	15.57433000	-0.23363100	0.77957000
H	16.64178200	-0.28290100	0.51750600
H	15.15117600	-1.24239100	0.64889700
H	15.50416900	0.01696400	1.85009500

XVIII

C	-6.71443000	0.69061200	-0.18727700
C	-7.47384600	-0.59534900	0.11812600
C	-6.65891100	-1.81368500	-0.29749400
C	-5.29435800	-1.88517600	0.41923800
C	-4.53784400	-0.52512400	0.21554700
C	-3.16833300	-0.49701600	0.89822000
C	-2.45493900	0.80043500	0.68084100
C	-3.04984300	1.90749000	0.20817800
C	-4.52363600	1.93365100	-0.15809700
C	-5.33741300	0.78074500	0.50143300
C	-5.08340000	3.34042900	0.08486900
C	-4.30369300	4.38756500	-0.70461400
C	-2.81759800	4.29029000	-0.45903700
C	-2.28444600	3.13722900	-0.01921400
C	-1.96274500	5.51518300	-0.70997000
C	-2.16050900	6.06645900	-2.12632400
C	-2.20659500	6.60119400	0.34605800
C	-4.43642600	-2.90511400	-0.34086200
C	-5.48846600	-2.32877200	1.87564400
C	-5.51050000	1.06069400	2.00303400
O	-4.35068200	-2.95004700	-1.54238500
O	-3.74908800	-3.72214200	0.46503300
H	-6.56261200	0.74953000	-1.27983400
H	-7.32773500	1.56243200	0.08969000
H	-7.73245800	-0.64391400	1.18885100
H	-8.43293600	-0.59463900	-0.42295400
H	-6.47664400	-1.77278500	-1.38237400
H	-7.21936300	-2.74336800	-0.10484700
H	-4.34594700	-0.49676100	-0.87155400
H	-2.54227800	-1.32255500	0.52207800
H	-3.26502600	-0.68295100	1.98310000
H	-1.38767100	0.83487300	0.92545700
H	-4.57307500	1.76460700	-1.25172600
H	-5.01370100	3.58780500	1.15661800
H	-6.14774100	3.38487800	-0.18946900
H	-4.66500100	5.39645500	-0.44667500
H	-4.50267800	4.26956300	-1.78585700
H	-1.20606800	3.08283800	0.16726000
H	-0.91049800	5.19744500	-0.61807800
H	-1.47111900	6.90312000	-2.31799500
H	-3.18509700	6.44483300	-2.27148200
H	-1.97531000	5.29085900	-2.88477100
H	-1.53474900	7.45859300	0.18439700
H	-3.23979400	6.98159300	0.30512300

H	-2.02911600	6.21258800	1.35972200
H	-4.55788100	-2.29857600	2.45535600
H	-6.23229000	-1.70631000	2.38707200
H	-5.85428000	-3.36505600	1.90431400
H	-5.97800700	0.22316300	2.53327700
H	-6.15444300	1.93845600	2.15972100
H	-4.54639900	1.26819000	2.49042100
C	-2.82059600	-4.61654100	-0.14509600
H	-2.67479600	-5.42261900	0.58773300
H	-3.26564200	-5.03857400	-1.05849100
C	-1.50746900	-3.91899800	-0.45781900
H	-1.70110000	-3.11390600	-1.18558900
H	-1.14155000	-3.44215600	0.46594800
C	-0.45605400	-4.87424500	-1.01777200
H	-0.88960700	-5.42908200	-1.86720200
H	-0.20428700	-5.63201300	-0.25397900
C	0.82412400	-4.18117700	-1.48613900
H	1.49722600	-4.93778100	-1.92417300
H	0.57818700	-3.48365900	-2.30657000
C	1.57513100	-3.42315500	-0.39357800
H	1.76504100	-4.10163900	0.45821100
H	0.94185600	-2.61059700	0.00256800
C	2.89639000	-2.82642000	-0.86980500
H	3.54900400	-3.63443000	-1.24653000
H	2.70584400	-2.16645200	-1.73511400
C	3.63599400	-2.04101100	0.20918200
H	2.98270700	-1.23203300	0.58289000
H	3.82371400	-2.69955100	1.07634300
C	4.95716100	-1.44239900	-0.26439200
H	5.61235000	-2.25125400	-0.63490000
H	4.76945400	-0.78665800	-1.13360100
C	5.69258700	-0.65157700	0.81353300
H	5.88180300	-1.30762400	1.68227800
H	5.03610100	0.15575300	1.18521800
C	7.01315300	-0.05092300	0.34006800
H	6.82423000	0.61340100	-0.52285700
H	7.65814400	-0.86207600	-0.03949800
C	7.74621200	0.73327700	1.42667800
H	7.06011000	1.49354900	1.83820000
H	7.98206700	0.05485200	2.26655600
C	9.02812000	1.42558700	0.96226700
H	8.78687000	2.12964100	0.14535100
H	9.41678600	2.04568600	1.78841400
C	10.13365800	0.47928600	0.49801300
H	9.79015000	-0.10202600	-0.37504900
H	10.33558100	-0.26125900	1.29335700
C	11.43104900	1.19549700	0.13380200
H	11.22541100	1.94413500	-0.65269300
H	11.78948400	1.76980800	1.00733400
C	12.53816800	0.25763900	-0.34103700
H	12.73852600	-0.49162500	0.44463000
H	12.17849500	-0.31283200	-1.21513000
C	13.83158800	0.98077000	-0.69969200
H	14.60803200	0.27855800	-1.03816800
H	14.23283200	1.53079800	0.16643100
H	13.66734800	1.71216600	-1.50701900

XIX

C	4.62875100	-0.62909600	2.49119700
C	3.22008100	-0.92584500	2.99414100
C	2.17705900	-0.37962400	2.02612800
C	2.31834400	-0.98742100	0.61105800
C	3.78928300	-0.79692200	0.11519900
C	4.02118700	-1.37623400	-1.28086700
C	5.42519400	-1.15854000	-1.75204900
C	6.43139100	-0.78691300	-0.94376700
C	6.22362100	-0.54240500	0.54132400
C	4.92899000	-1.20946700	1.09384300
C	7.50830100	-0.89525000	1.30040500
C	8.69560300	-0.09384500	0.77538300
C	8.84000300	-0.21351000	-0.72241700
C	7.77882000	-0.55513000	-1.47382100
C	10.19838000	0.04776500	-1.33945100
C	10.75555900	1.41571300	-0.93037100
C	11.19254400	-1.07473600	-1.01428300
C	1.41829600	-0.18658100	-0.33920700
C	1.79549400	-2.42902300	0.60445800
C	5.12934500	-2.73089600	1.18298100
O	0.50626200	-0.64369000	-0.97987700
O	1.75680000	1.10902500	-0.38644300
H	4.75502800	0.46767000	2.44860600
H	5.37006000	-0.99944500	3.21657300
H	3.08381700	-2.00952100	3.14194500
H	3.07713700	-0.46608700	3.98461900
H	2.29836300	0.71266000	1.95720300
H	1.15731300	-0.56562600	2.40137500
H	3.89849600	0.29588200	0.01841800
H	3.31762000	-0.92137900	-1.99960800
H	3.79326200	-2.45704400	-1.30283500
H	5.63099400	-1.31843000	-2.81595100
H	6.08560500	0.55063500	0.65787300
H	7.72984200	-1.96780000	1.17763700
H	7.38263400	-0.71153900	2.37780000
H	9.62124700	-0.42638300	1.27292500
H	8.58053800	0.97194800	1.04657800
H	7.90003200	-0.65159100	-2.55853500
H	10.05859000	0.05652900	-2.43351500
H	11.70227900	1.62239200	-1.45283400
H	10.96059300	1.45954800	0.15133400
H	10.04801400	2.22269000	-1.17397500
H	12.15537400	-0.89984200	-1.51926500
H	11.39545200	-1.13589100	0.06691100
H	10.80583800	-2.05108500	-1.34167100
H	1.92022500	-2.91093100	-0.37363700
H	2.29370500	-3.04637900	1.36035900
H	0.71913600	-2.43150600	0.82399200
H	4.21114300	-3.25882400	1.46423100
H	5.88499100	-2.97569100	1.94369300
H	5.47638600	-3.15044600	0.22720800
C	0.98405500	1.96458500	-1.22391700
H	1.64223900	2.81601900	-1.44801200
H	0.74907100	1.43892900	-2.16186700
C	-0.29127100	2.42499200	-0.53876200
H	-0.89612100	1.53591800	-0.30419400
H	-0.02952400	2.90495400	0.41978600
C	-1.08970700	3.39525500	-1.40670700

H	-1.37561700	2.89081700	-2.34693100
H	-0.43933900	4.23799900	-1.69882900
C	-2.34489900	3.95147600	-0.73326800
H	-2.05690500	4.48765400	0.18891500
H	-2.79439800	4.71104400	-1.39546800
C	-3.40349800	2.90331700	-0.39681600
H	-2.99321500	2.16789100	0.31629600
H	-3.65112600	2.33136200	-1.30932200
C	-4.68054700	3.49865100	0.18910100
H	-4.43036000	4.07742600	1.09654200
H	-5.10524000	4.22804700	-0.52403200
C	-5.73938300	2.45426500	0.53088400
H	-5.97242600	1.87350400	-0.37805400
H	-5.31917100	1.72983600	1.25208200
C	-7.01948200	3.05392700	1.10933800
H	-6.75558700	3.68087500	1.97848700
H	-7.46576200	3.74058600	0.36729200
C	-8.06761100	2.02746600	1.54098800
H	-8.89785400	2.55513500	2.04131300
H	-7.63085800	1.36001400	2.30582300
C	-8.63876400	1.18131500	0.40479100
H	-7.83386400	0.59299800	-0.06844600
H	-9.02879700	1.84918500	-0.38484400
C	-9.74477400	0.23038000	0.85357800
H	-9.35590100	-0.43043800	1.64916700
H	-10.56091900	0.81179900	1.31916700
C	-10.31434500	-0.62259000	-0.27615300
H	-10.70298300	0.03854700	-1.07159700
H	-9.49785700	-1.20351800	-0.74160300
C	-11.41880400	-1.57575700	0.17111600
H	-12.23483800	-0.99520000	0.63795600
H	-11.02933100	-2.23773700	0.96545000
C	-11.98904600	-2.42704600	-0.95955200
H	-12.37974900	-1.76488100	-1.75339400
H	-11.17251600	-3.00616800	-1.42765800
C	-13.09245900	-3.38308300	-0.51304800
H	-12.69958700	-4.04321400	0.27983900
H	-13.90607200	-2.80240500	-0.04437600
C	-13.65675500	-4.22874100	-1.64926500
H	-14.44820400	-4.90634300	-1.29570100
H	-12.87104300	-4.84581300	-2.11354600
H	-14.08808700	-3.59474100	-2.44028500

XX

C	6.39210100	-1.55675200	2.18066500
C	5.98324000	-3.01503800	2.35297900
C	4.50898500	-3.20355200	2.01909000
C	4.17209100	-2.78369700	0.57255400
C	4.68719800	-1.32143400	0.33204900
C	4.40212700	-0.81580700	-1.08392900
C	4.88427100	0.58592500	-1.29038200
C	5.72297700	1.21608600	-0.45219800
C	6.25717800	0.54841200	0.80317100
C	6.15121700	-1.00506000	0.76068400
C	7.65686500	1.09282300	1.11298800
C	7.63591100	2.61068300	1.26611700
C	6.98448700	3.28897600	0.08551300
C	6.12665800	2.60519200	-0.69156700
C	7.32988900	4.73646000	-0.19670500

C	7.09932000	5.62594300	1.02996900
C	8.76244400	4.88357300	-0.72608800
C	2.64491800	-2.66344300	0.48465900
C	4.68457700	-3.84165500	-0.41396000
C	7.19722600	-1.57045400	-0.21363600
O	1.96695600	-2.09804000	1.30554100
O	2.13215300	-3.21960800	-0.61843400
H	5.80869000	-0.94890700	2.89504200
H	7.45101200	-1.42977300	2.45534900
H	6.61312300	-3.66766900	1.72613600
H	6.16621500	-3.32907400	3.39248200
H	3.90280400	-2.59225500	2.70525200
H	4.20377600	-4.25167600	2.17362100
H	4.06486700	-0.71356100	1.01207500
H	3.31991200	-0.86235400	-1.29057200
H	4.86593700	-1.47572100	-1.83908100
H	4.52552200	1.11578600	-2.17946400
H	5.60373900	0.88296100	1.63270100
H	8.34608800	0.83464700	0.29265500
H	8.05610200	0.63245800	2.02883400
H	8.66297400	2.98758100	1.40039400
H	7.09553900	2.88876000	2.18985500
H	5.66959700	3.10502700	-1.55287800
H	6.64827000	5.08048700	-0.99273100
H	7.27544800	6.68400900	0.78202900
H	7.78365800	5.36261500	1.85237900
H	6.06852500	5.52973200	1.40320800
H	8.97610600	5.93281800	-0.98318500
H	9.50615700	4.57173500	0.02458600
H	8.91459100	4.27171400	-1.62749300
H	4.54912900	-3.54518100	-1.46123300
H	5.74755800	-4.05657900	-0.25248500
H	4.13513100	-4.78285700	-0.27047800
H	7.09058500	-2.65132400	-0.36053000
H	8.21358300	-1.39840300	0.16985800
H	7.13224700	-1.09097000	-1.20148900
C	0.72996500	-3.07479300	-0.83405200
H	0.45458700	-3.90502000	-1.49963900
H	0.20048700	-3.19205800	0.12320300
C	0.39761100	-1.73445100	-1.46765700
H	0.73088600	-0.93215200	-0.78966300
H	0.97302300	-1.62942100	-2.40314300
C	-1.09387400	-1.57962600	-1.74953600
H	-1.65115700	-1.70674400	-0.80598700
H	-1.43281000	-2.39281700	-2.41710800
C	-1.44334300	-0.23356900	-2.38150000
H	-0.82943800	-0.09796400	-3.28836900
H	-1.14610200	0.57610900	-1.69136800
C	-2.91740600	-0.06800600	-2.75212600
H	-3.20471500	-0.85790000	-3.46964200
H	-3.04175400	0.88685500	-3.29108600
C	-3.88080600	-0.09147600	-1.56702700
H	-3.82463400	-1.06706200	-1.05391800
H	-3.55965000	0.65959300	-0.82264500
C	-5.33121300	0.17450800	-1.96006400
H	-5.39998900	1.15662200	-2.46159300
H	-5.64683700	-0.56953500	-2.71354500
C	-6.30031100	0.14101700	-0.78196500
H	-6.23279100	-0.84194400	-0.28191000
H	-5.98282400	0.88324100	-0.02763600

C	-7.75083800	0.40849200	-1.17269000
H	-8.06844100	-0.33390800	-1.92690600
H	-7.81878400	1.39150700	-1.67255600
C	-8.71894200	0.37485900	0.00629800
H	-8.40135700	1.11759400	0.76012400
H	-8.65030800	-0.60788800	0.50660300
C	-10.16992200	0.64141900	-0.38344200
H	-10.23851100	1.62384800	-0.88439800
H	-10.48785000	-0.10190100	-1.13660700
C	-11.13727500	0.60886000	0.79620400
H	-11.06808600	-0.37335700	1.29749000
H	-10.81933800	1.35252800	1.54899100
C	-12.58855800	0.87474300	0.40697700
H	-12.90652700	0.13047400	-0.34527100
H	-12.65734500	1.85662300	-0.09509700
C	-13.55563600	0.84342300	1.58682000
H	-13.48639000	-0.13835000	2.08942700
H	-13.23765800	1.58796600	2.33903500
C	-15.00806800	1.10883700	1.19839200
H	-15.07423400	2.08933300	0.69534600
H	-15.32303900	0.36404400	0.44679600
C	-15.96693200	1.07645200	2.38332500
H	-17.00389500	1.27055400	2.07097400
H	-15.69636600	1.83557400	3.13447700
H	-15.94670300	0.09549800	2.88435200

5. References

(1) Yadav, J. S.; Baishya, G.; Dash, U. Synthesis of (+)-amberketal and its analog from l-abietic acid. *Tetrahedron* **2007**, *63*, 9896-9902.

(2) Yang, L.; Chen, F.; Gao, C.; Chen, J.; Li, J.; Liu, S.; Zhang, Y.; Wang, Z.; Qian, S. Design and synthesis of tricyclic terpenoid derivatives as novel PTP1B inhibitors with improved pharmacological property and in vivo antihyperglycaemic efficacy. *J. Enzyme Inhib. Med. Chem.* **2020**, *35*, 152-164.

(3) Lee, H.-J.; Ravn, M. M.; Coates, R. M. Synthesis and characterization of abietadiene, levopimaradiene, palustradiene, and neoabietadiene: hydrocarbon precursors of the abietane diterpene resin acids. *Tetrahedron* **2001**, *57*, 6155-6167.
